# Chemical synthesis of human syndecan-4 glycopeptide bearing O-, N-sulfation and multiple aspartic acids for probing impacts of the glycan chain and the core peptide on biological functions†

**DOI:** 10.1039/d0sc01140a

**Published:** 2020-05-11

**Authors:** Weizhun Yang, Yigitcan Eken, Jicheng Zhang, Logan Emerson Cole, Sherif Ramadan, Yongmei Xu, Zeren Zhang, Jian Liu, Angela K. Wilson, Xuefei Huang

**Affiliations:** Department of Chemistry, Michigan State University 578 South Shaw Lane East Lansing MI 48824 USA; Chemistry Department, Faculty of Science, Benha University Benha Qaliobiya 13518 Egypt; Division of Chemical Biology and Medicinal Chemistry, Eshelman School of Pharmacy, University of North Carolina Chapel Hill NC 27599 USA; Department of Biomedical Engineering, Michigan State University East Lansing MI 48824 USA; Institute for Quantitative Health Science and Engineering, Michigan State University East Lansing MI 48824 USA huangxu2@msu.edu

## Abstract

Proteoglycans are a family of complex glycoproteins with glycosaminoglycan chains such as heparan sulfate (HS) attached to the core protein backbone. Due to the high structural heterogeneity of HS in nature, it is challenging to decipher the respective roles of the HS chain and the core protein on proteoglycan functions. While the sulfation patterns of HS dictate many activities, the core protein can potentially impact HS functions. In order to decipher this, homogeneous proteoglycan glycopeptides are needed. Herein, we report the first successful synthesis of proteoglycan glycopeptides bearing multiple aspartic acids in the core peptide and O- and N-sulfations in the glycan chain, as exemplified by the syndecan-4 glycopeptides. To overcome the high acid sensitivities of sulfates and base sensitivities of the glycopeptide during synthesis, a new synthetic approach has been developed to produce a sulfated glycan chain on a peptide sequence prone to the formation of aspartimide side products. The availability of the structurally well-defined synthetic glycopeptide enabled the investigation of their biological functions including cytokine, growth factor binding and heparanase inhibition. Interestingly, the glycopeptide exhibited context dependent enhancement or decrease of biological activities compared to the peptide or the glycan alone. The results presented herein suggest that besides varying the sulfation patterns of HS, linking the HS chain to core proteins as in proteoglycans may be an additional approach to modulate biological functions of HS in nature.

## Abstract 2

Attaching heparan sulfate glycan on a peptide backbone can modulate biological functions of the glycan.

## Graphical abstract

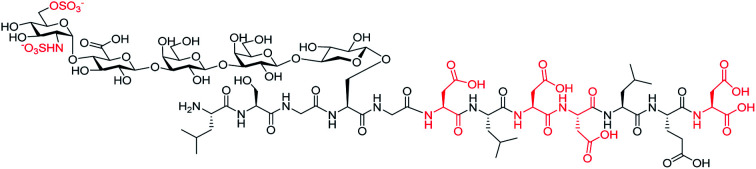

Heparan sulfate (HS) and its more highly sulfated derivative heparin are a class of highly sulfated polysaccharides.^1,2^ They are known to have multifaceted biological functions,^3–5^ which range from growth factor and chemokine binding, enzyme inhibition, to reducing blood clot formation. HS in nature is highly heterogenous varying both in terms of the number and the location of sulfates as well as the backbone uronic acid structures. Many outstanding studies have been carried out synthesizing diverse HS structures to decode the structure and activity relationship,^6–12^ which has led to novel therapeutics such as the synthetic anti-coagulant fondaparinux.^3^

In nature, HS is covalently linked to a core protein forming heparan sulfate proteoglycans (HSPGs).^13,14^ In contrast to the rich activities of HS,^3,15,16^ core proteins of HSPGs were previously thought to mainly serve as carriers of HS. However, there has been evidence suggesting that the core protein itself can be biologically active.^17–19^ For example, the core protein of a HSPG syndecan-1 can regulate invasive properties of cancer cells with the mutation of a 5 amino acid sequence in the core resulting in a loss of invasive migration abilities of these cells.^20^ Another HSPG, syndecan-4,^21^ can activate kinases^22^ and facilitate the assembly of focal adhesions.^23^ Interestingly, syndecan-4 mutants without any HS chains were found to be equally effective as the wild type glycoprotein in promoting focal adhesion suggesting that the core protein dictates the activity in this case.^23^ As both HS and the core protein can be biologically active, the core protein may potentially modulate HS activities, adding another dimension to the functional complexity of HS.

To decipher how the core protein impacts HS functions, HSPG glycopeptides bearing well-defined homogeneous glycans are critically needed, and total synthesis is an important strategy to access these complex molecules. While many innovative methods to produce HS and heparin oligosaccharides have been established,^6–12^ strategies for HS glycopeptide synthesis are underdeveloped.^24–26^ Currently, structural features common in naturally existing HSPGs, such as aspartic acids in the core peptide and glycan chains bearing both O- and N-sulfates, are not accessible with the existing synthetic methods.

Herein, we report the first successful synthesis of a human syndecan-4 ^21^ (amino acids 60–71) HS glycopeptides **1** and **2** bearing an N- and O-sulfated glycan chain and four aspartic acid (Asp) residues in the peptide backbone (Fig. 1). The availability of such structurally well-defined glycopeptides enabled the analysis of the roles of glycan and core peptide in their interactions with biological targets. Interestingly, the functions of the glycan chain can be modulated by the peptide, which indicates that besides varying HS structures, the attachment of HS onto protein may be another avenue to direct HS functions.

**Fig. 1:**
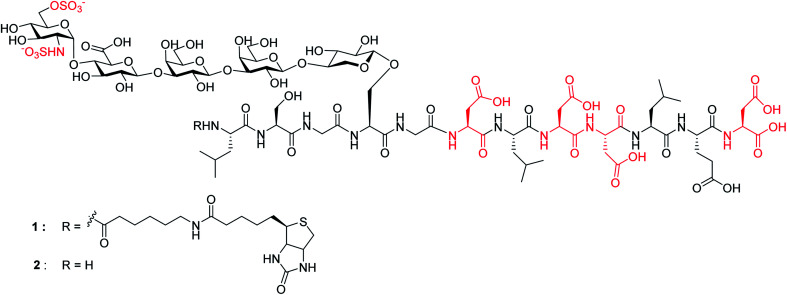
Synthetic targets **1** and **2**.

## Results and discussion

### Synthetic design of syndecan-4 glycopeptides

**Fig. 2:**
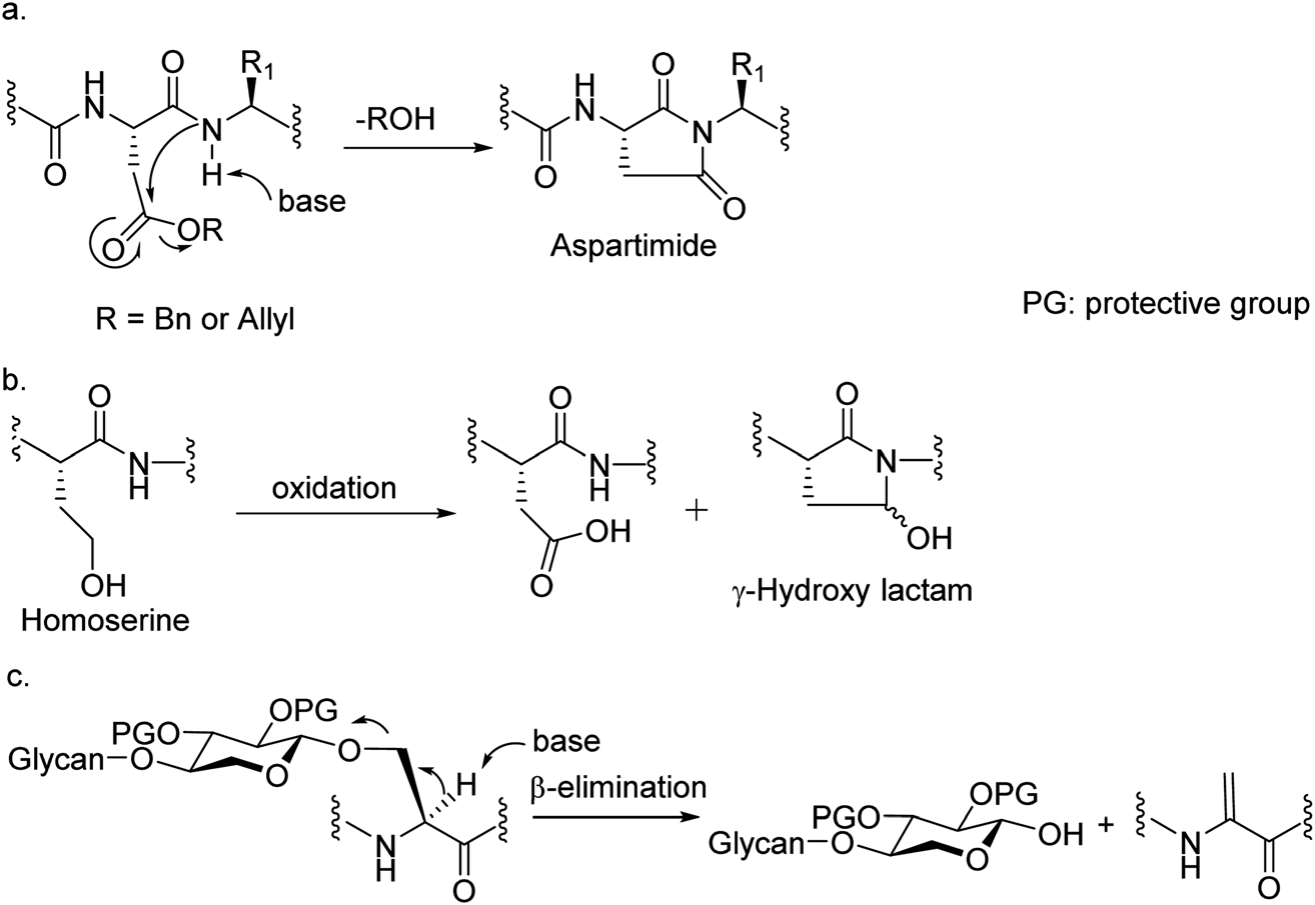
Obstacles encountered in HS glycopeptide synthesis. (a) The formation of aspartimide during peptide synthesis; (b) the formation of γ-hydroxy lactam during the conversion of homoserine to Asp; (c) the potential elimination of glycan chain under basic conditions.

Among many obstacles faced in HSPG synthesis, a major hurdle is that common amino acid side chain protective groups such as *t*-butyl (*t*Bu) ester and trityl requiring strong acids for deprotection cannot be applied in synthesis of glycopeptides bearing highly acid labile O- or N-sulfates. When alternative protective groups such as benzyl and allyl esters were used to protect the Asp side chain carboxylic acid, aspartimide formation is a major side reaction significantly lowering the yield of the desired peptide (Fig. 2a). Amide backbone protection by pseudoproline^27,28^ or 2,4-dimethoxybenzyl^29^ could effectively suppress the aspartimide formation during glycopeptide synthesis. However, the strong acidic conditions required to cleave the pseudoproline or 2,4-dimethoxybenzyl group suggests they are not suitable for HS glycopeptides with free O- or N-sulfates. Recently, the cyanosulfurylide moiety was developed as a novel carboxylic acid protecting group to prevent aspartimide formation during polypeptide synthesis,^30^ the applicability of which is yet to be demonstrated in sulfated glycopeptide synthesis. We reported a homoserine method where homoserine was used as the Asp surrogate, which could be converted to Asp after assembly of the full glycopeptide (Fig. 2b).^31^ While this strategy gave good yields of glycopeptides containing a single Asp, the yield was significantly lowered when the condition was extended to targets bearing more than one Asp due to the formation of γ-hydroxy lactam side products (Fig. 2b). As the core proteins of HSPGs can contain multiple aspartic acids flanking the serine linkage site of glycans,^32^ an effective strategy enabling access to these glycopeptides is needed.

Another significant hurdle in HSPG glycopeptide synthesis is N-sulfation. For the synthesis of HS oligosaccharides, N-sulfates are traditionally introduced by treating the free amines with a sulfation agent such as SO_3_.pyridine under strong basic conditions.^8,33–36^ However, when amine bearing HS glycopeptide precursors were subjected to sulfation under the traditional condition, β-elimination of the glycan chain from the peptide backbone was observed as a major side reaction presumably due to the high basicity of the reaction media needed to promote N-sulfation (Fig. 2c). Decreasing the basicity of reaction led to incomplete sulfation with low yields of the desired glycopeptides.

**Scheme 1:**
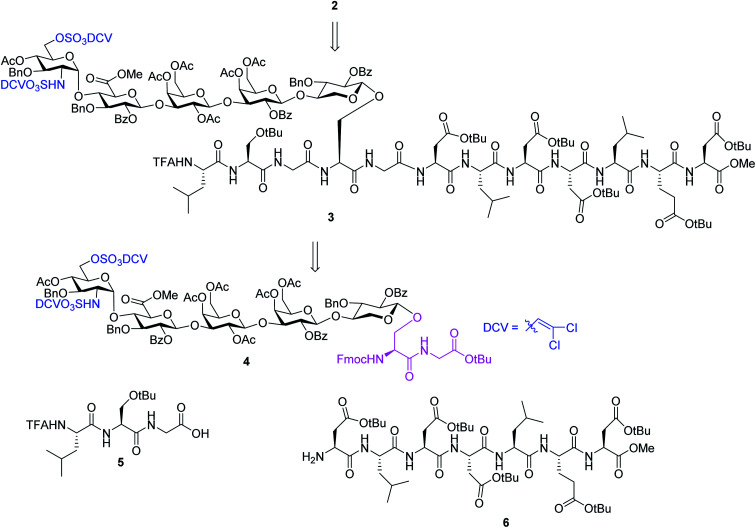
Retrosynthetic design of HS glycopeptide **2**.

We hypothesize that if acid stabilities of sulfates could be enhanced, the aforementioned difficulties could be potentially overcome. Innovative research by the Taylor,^37–39^ Linhardt^40,41^ and Widlanski^42^ groups and later extended by others^43–46^ demonstrated that sulfate esters can be attractive protective groups for sulfates. As the utility of sulfate ester chemistry has not been established in HS glycopeptide synthesis, we began to examine various sulfate esters. The dichlorovinyl (DCV) sulfate ester group^38^ was found to be attractive due to its high stability to acids such as trifluoroacetic acid (TFA).^47^ We envision that DCV sulfates can be installed first into protected glycopeptides followed by peptide elongation. Upon acidic cleavage of side chain protecting groups such as *t*Bu to free Asp, the DCV moieties can be removed under mild conditions to release the sulfate groups. With this consideration in mind, we designed the synthesis of syndecan-4 glycopeptide **2** from the fully protected precursor **3**. In turn, **3** can be generated using a cassette approach from glycosyl serine–glycine **4** building block followed by peptide chain elongation (Scheme 1). While previous synthesis utilized glycosyl serine as the cassette for peptide synthesis,^26,48^ we found that the glycosylated serine was prone to base promoted glycan elimination (Fig. 2c) during coupling with peptides more than five amino acids long. This was presumably due to the reduced nucleophilicity of peptide amine slowing down the amide formation reaction and resulting in a higher relative rate of elimination. To overcome this, we chose to explore the utility of glycosyl dipeptide **4** (glycan–serine–glycine) as the cassette, as in all HSPGs, the glycosylated serine is linked to a glycine residue at its C-terminus.

### Synthesis of the glycosyl dipeptide cassette **4**

**Scheme 2:**
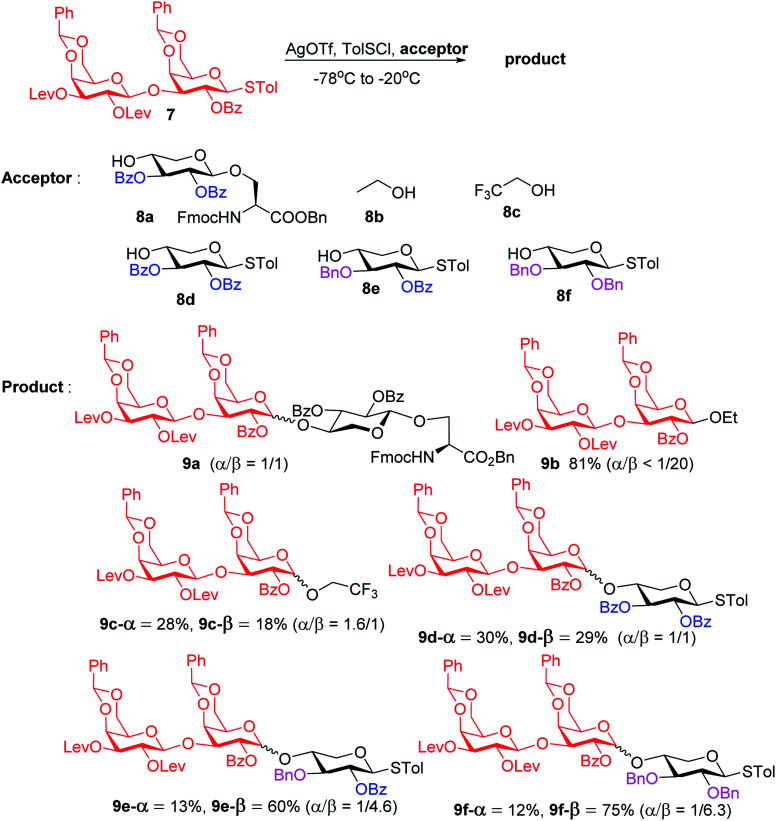
Stereochemical outcomes of glycosylation of disaccharide **7** with various acceptors.

To form the glycosyl dipeptide cassette **4**, the galactose (gal)–gal–xylose (xyl) trisaccharide at the reducing end is an important module. Previous synthesis of the trisaccharide utilized gal–gal disaccharide donor **7** to glycosylate xyloside acceptor **8a** bearing two benzyol (Bz) groups.^24^ However, this reaction gave no selectivity (α : β = 1 : 1) towards the desired β anomer **9a-β** (Scheme 2). The loss of 1,2-*trans* selectivity on galactosyl donors bearing 2-*O*-acyl groups has been reported by several groups.^49–52^ The lack of β-selectivity was presumably due to the presence of 4,6-*O*-benzylidene in the donor, conformationally reducing the propensity of neighboring group participation by the 2-*O* acyl group. This was supported by the reports that similar glycosylations using galactosyl donors without the 4,6-*O*-benzylidene moiety gave β-anomers only.^50,51^ However, in our hands, the replacement of the two 4,6-*O*-benzylidenes in **7** with four O-acetate groups did not lead to any improvements of β-selectivity in glycosylation of **8a**.

To address this stereoselectivity problem, we turned our attention to the influence of glycosyl acceptor reactivity, as acceptor reactivities can significantly impact stereoselectivities.^53–55^ Two nucleophiles, *i.e.*, ethanol **8b** and trifluoroethanol (TFE) **8c**, were tested (Scheme 2). Glycosylation between **7** and the more reactive acceptor ethanol provided **9b** with high yield and β-selectivity (α/β < 1:20), while less reactive acceptor TFE gave a slight preference for the α-anomer product (α/β = 1.6/1). Inspired by this result, three thioxyloside acceptors (**8d–8f**) with different patterns of Bz and benzyl (Bn) groups on O-2 and O-3 positions were subsequently probed. Pre-activation based glycosylations^56^ between donor **7** and the di-Bz acceptor **8d** gave product **9d** with no stereoselectivity (α/β = 1/1), which was consistent with the result of **9a**. A significant increase of β-selectivity was observed upon switching 3-*O*-Bz (**8d**) to 3-*O*-Bn (**8e**),^57^ while the di-*O*-Bn protected xylosyl acceptor **8f** further enhanced stereoselectivity towards the β-product (**9e-β** and **9f-β**). The results revealed that the β-selectivity of this 2 + 1 glycosylation reaction critically depends on the acceptor reactivity.

**Fig. 3:**
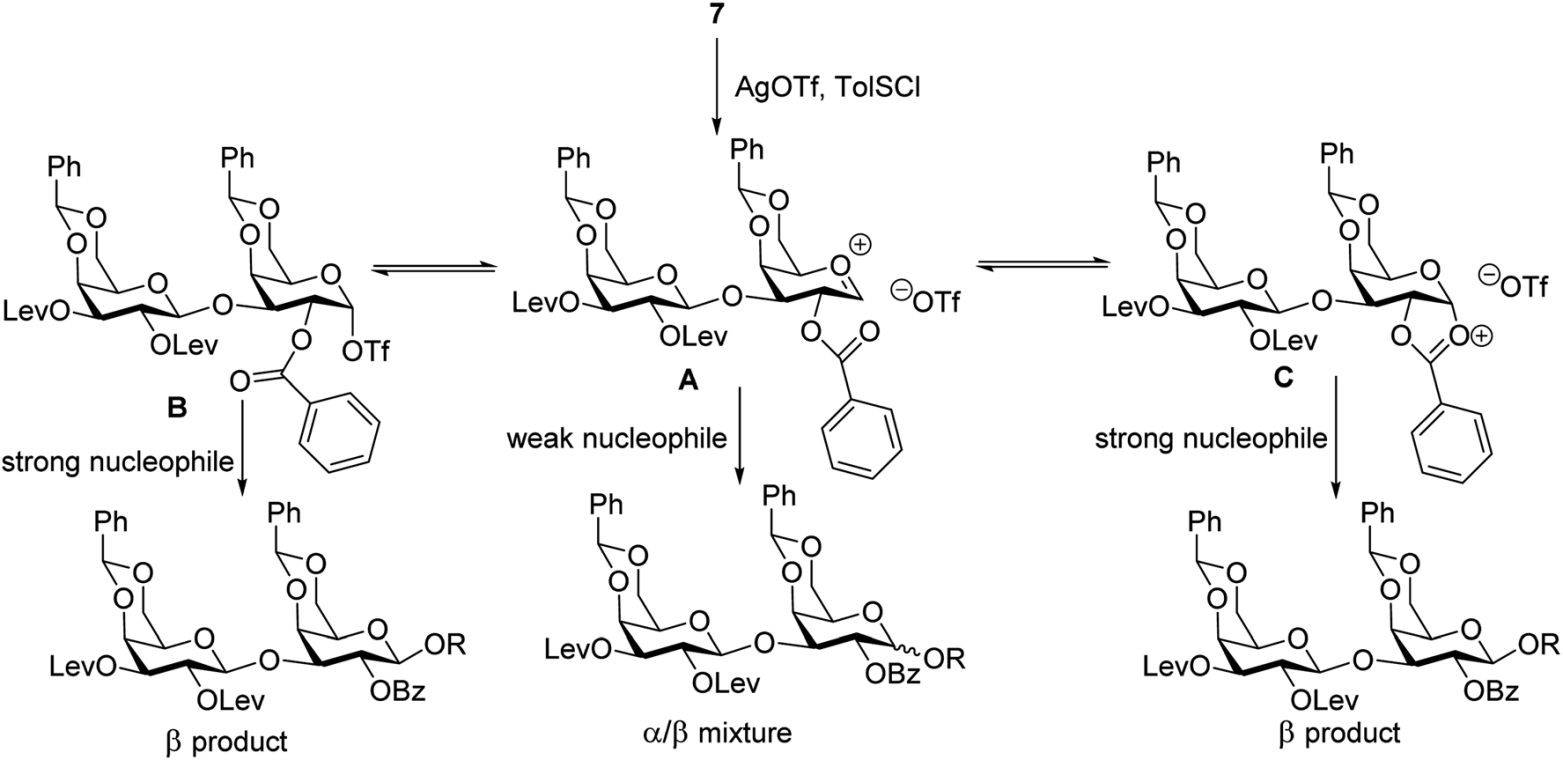
Proposed pathway for stereochemical outcome of disaccharide **7** glycosylation.

A plausible pathway to explain the outcome about the stereoselectivity is depicted in Fig. 3. Activation of **7** would lead to the oxocarbenium ion **A**, which can equilibrate with the glycosyl triflate **B**^58,59^ and/or dioxalenium ion **C**. The formation of the β-anomer can be explained through an S_N_2-like substitution on intermediate **B** or **C** by a reactive acceptor, while the less reactive acceptor requires more reactive oxocarbenium ion **A**^60,61^ leading to α,β-mixtures in S_N_1-like fashion.

**Scheme 3:**
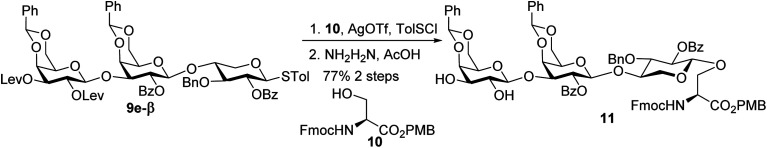
Synthesis of trisaccharide serine **11**.

With the 2-*O*-Bz moiety on xylose of trisaccharide **9e-β**, it is a suitable donor for formation of 1,2-*trans* xylosyl linkage (Scheme 3). The glycosylation between **9e-β** and serine **10** went smoothly followed by the removal of Lev groups to afford diol **11**. The PMB ester was selected as it could be selectively removed for peptide chain elongation.

**Scheme 4:**
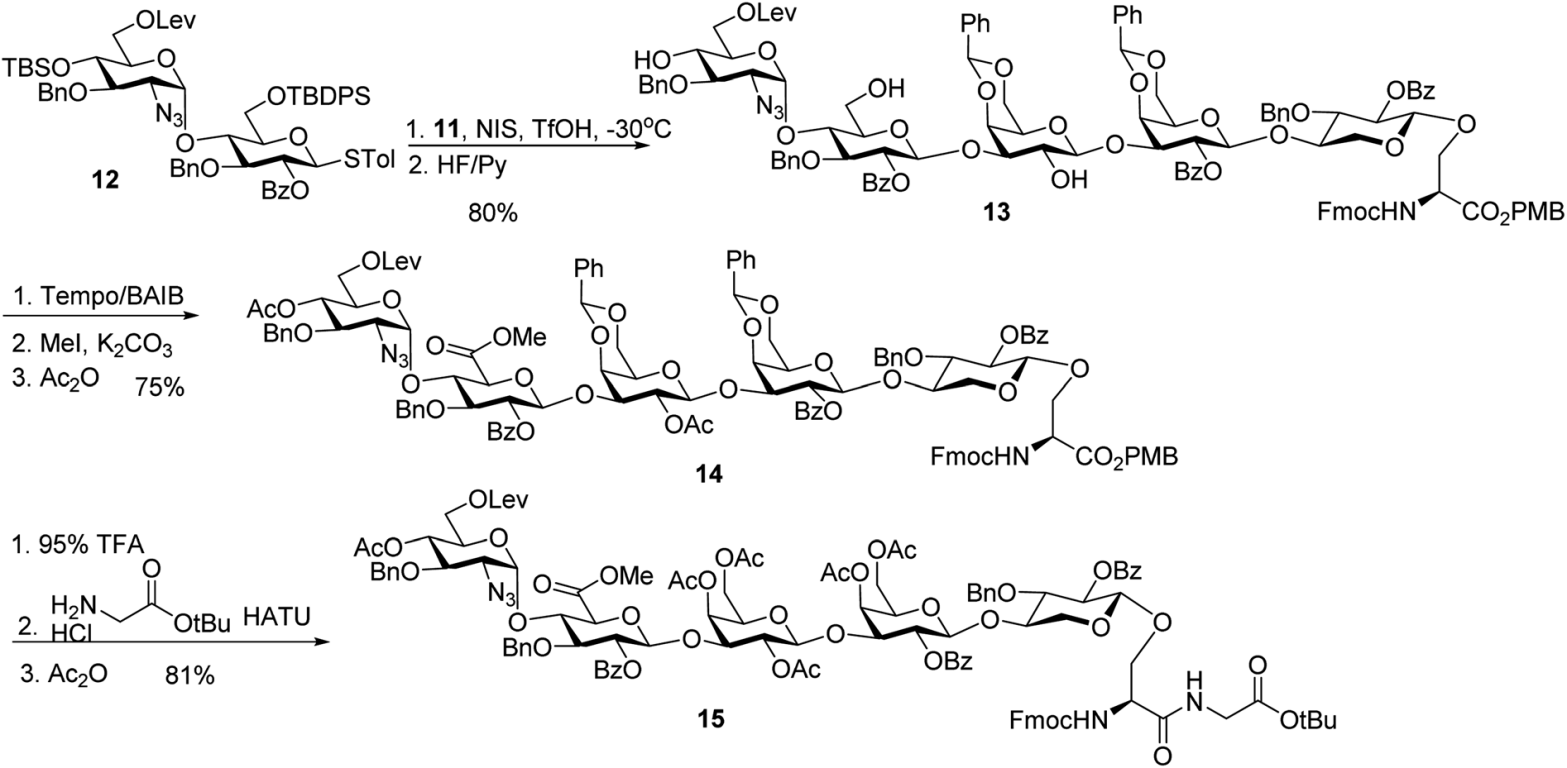
Synthesis of pentasaccharide – dipeptide module **15**.

With the trisaccharide serine **11** in hand, the 2 + 3 coupling between **12** and **11** produced the pentasaccharide followed by removal of silyl ether groups to afford compound **13** in 80% yield for the two steps (Scheme 4). The primary hydroxyl group of **13** was selectively oxidized to a carboxylic acid, and subsequent methylation and acetylation generated compound **14** in a good yield. **14** was treated with 95% TFA to cleave the two benzylidenes and PMB ester. The resulting carboxylic acid was coupled with glycine *t*Bu ester followed by acetylation to give glycopeptide **15**.

### Synthesis of sulfate ester bearing glycopeptides

**Scheme 5:**
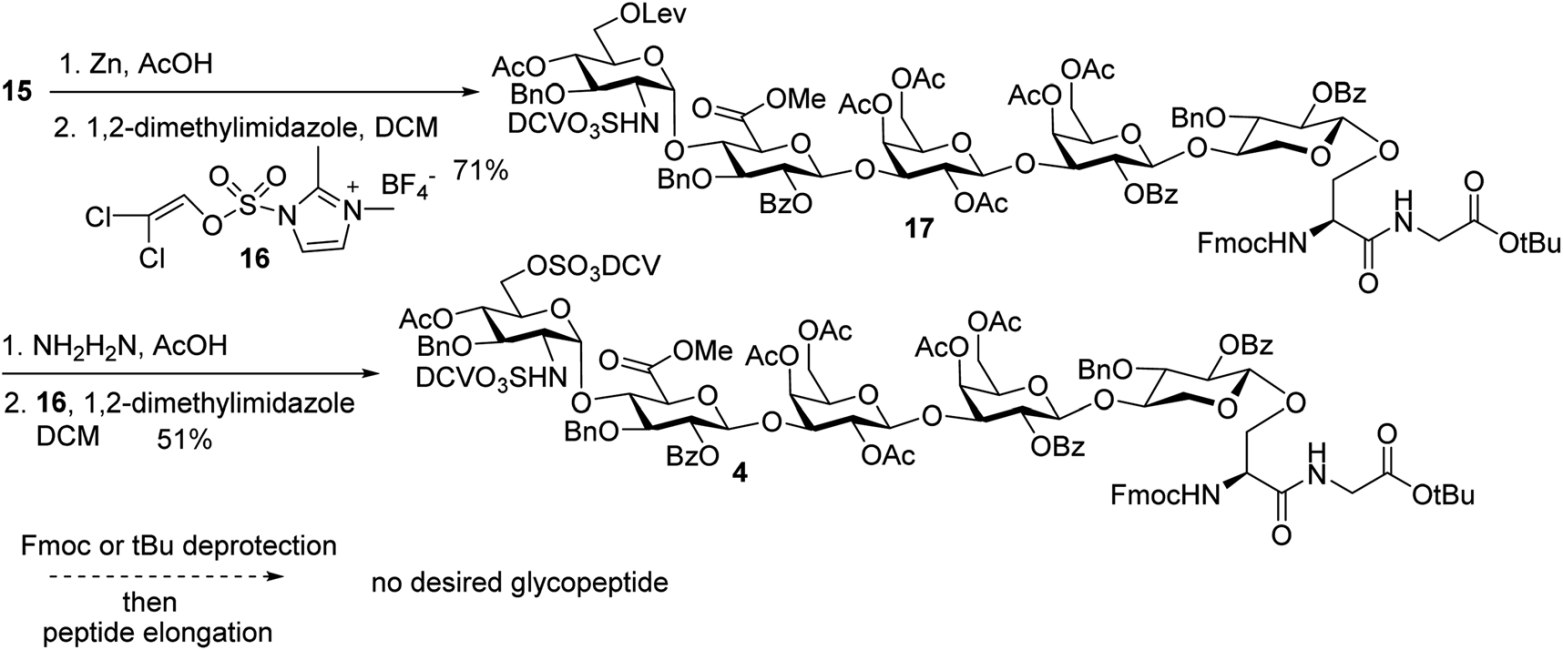
Failure in peptide elongation of glycopeptide **4**.

In order to introduce sulfates to the glycan chain, the azide group in **15** was converted to a free amine by Zn reduction (Scheme 5). The sulfation of amine with reagent **16** ^38^ in the presence of 1,2-dimethylimidazole as the base provided **17** in 71% yield for the two steps. With the mild reaction condition, no elimination products were observed. Upon removal of Lev from **17**, sulfation of the free 6-OH proceeded to produce di-DCV protected glycopeptide **4**. However, attempts to elongate the peptide chain following Fmoc or *t*Bu removal from **4** failed to give any desired glycopeptides with multiple un-identifiable side products.

**Scheme 6:**
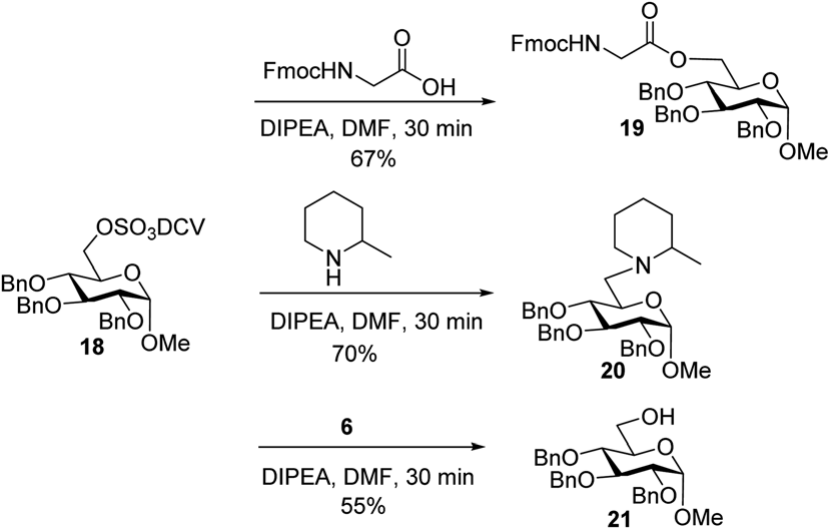
Stability test of 6-*O*-DCV sulfate in model compound **18**.

To better understand the failure to extend glycopeptide **4**, *O*-DCV sulfate **18** was tested as a model compound. When **18** was subjected to the typical condition (DIPEA, DMF, 30 min) for peptide coupling in the presence of Fmoc–glycine or 2-methylpiperidine, the substituted products **19** and **20** were produced. Mixing peptide **6** with **18** led to sugar **21** free of sulfates (Scheme 6). These model studies suggest that the failure of peptide elongation in glycopeptide **4** could result from the competitive substitution and hydrolysis reactions on the 6-*O* DCV sulfate ester.^45,62^

**Scheme 7:**
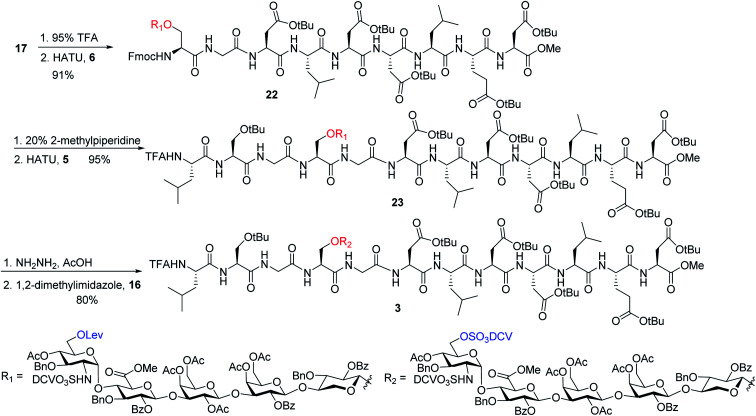
Synthesis of glycopeptide containing two DCV sulfate groups and full length core peptide.

To address this problem, we explored the alternative of installing the *O*-DCV sulfate group after peptide coupling. Compound **17** was treated with TFA to generate the carboxylic acid followed by coupling with heptapeptide **6** bearing four *t*Bu ester protected Asp residues (Scheme 7). The desired product **22** was obtained in 91% yield, with no β-elimination side product (Fig. 1c) observed. These results confirmed the hypothesis that the failure of **4** in glycopeptide synthesis was likely because of the instability of the 6-*O*-DCV sulfate ester under peptide coupling condition. Upon removal of Fmoc by 2-methylpiperidine and coupling with tripeptide **5**, compound **23** was formed in 95% yield. The Lev group on the glycan of **23** was cleaved by hydrazine acetate and the resulting primary OH was sulfated to afford the desired di-DCV protected glycopeptide **3**.

### Successful synthesis of glycopeptide **1** and the corresponding peptide and glycan fragments

**Scheme 8:**
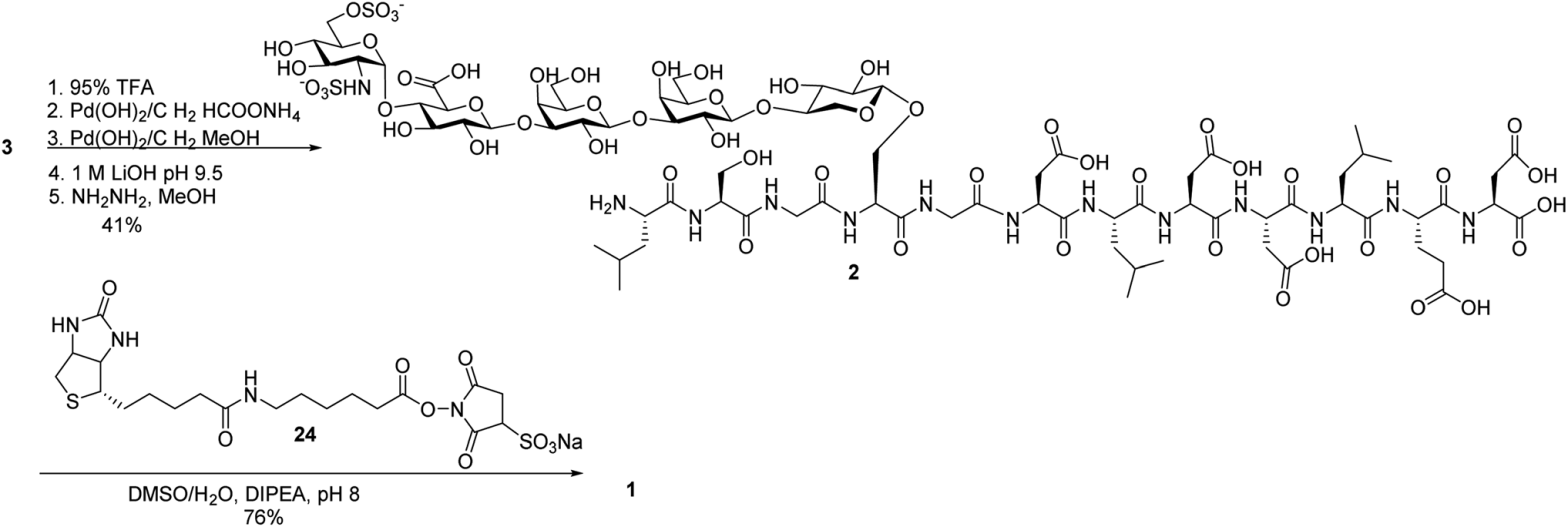
Global deprotection and biotinylation to form glycopeptide **1**.

Next, we focused on the global deprotection. *t*Bu groups on the peptide side chain of **3** were deprotected by TFA without affecting DCV groups (Scheme 8). Subsequently, the DCV esters were removed by hydrogenolysis in the presence of Pd(OH)_2_/C and ammonium formate to produce the free sulfates. Subsequent removal of the benzyl ethers, methyl esters and acyl groups by hydrogenolysis and a mild two step base treatment successfully produced the fully deprotected glycopeptide **2**. Finally, the free amine **2** was biotinylated to afford the target molecule **1**.

**Scheme 9:**
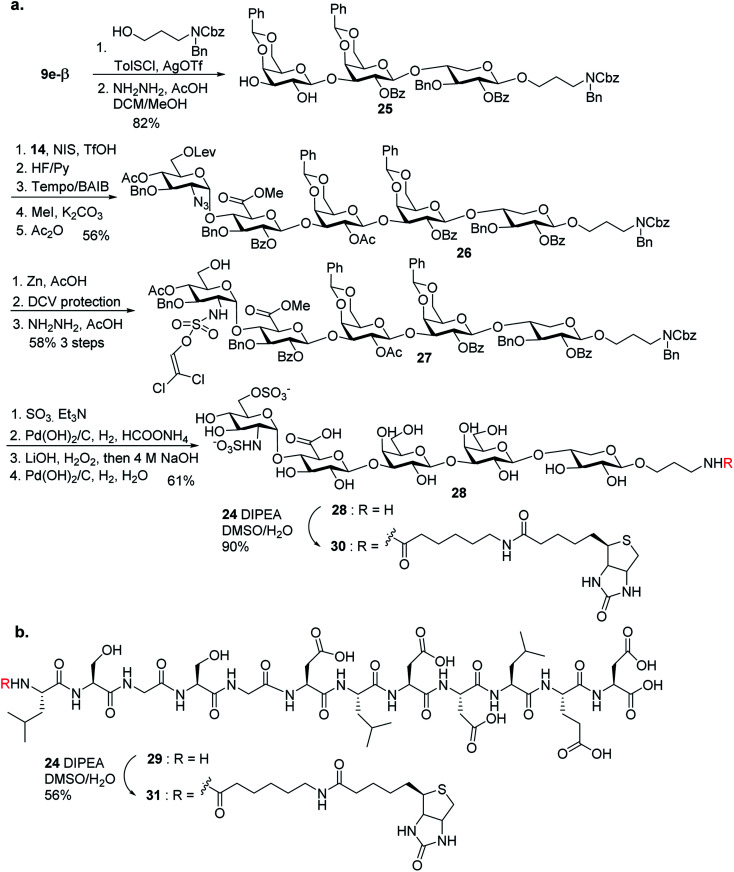
Synthesis of (a) compound **30**; and (b) compound **31**.

To aid in biological studies, pentasaccharide **30** bearing the same glycan sequence as glycopeptide **1** was synthesized from trisaccharide **9e-β** in an analogous manner as **2** followed by biotination (Scheme 9a). The corresponding biotinylated peptide **31** was also synthesized (Scheme 9b).

### Glycan **30** significantly inhibited the activities of heparanase while glycopeptide **1** had little effects

With the synthetic glycopeptide and the corresponding peptide and glycan in hand, we began to probe the impacts of glycan and peptide on biological functions of the glycopeptide. The endoglycosidase heparanase is an important enzyme for HS metabolism, which can cleave HS polysaccharide chains at the cell surface or in the extracellular matrix.^63^ Heparanase activities are important for a variety of biological processes, including tumor metastasis and cell proliferation.^64^ Some oligosaccharides can function as inhibitors of heparanase,^65^ which was the case for glycan **30** as it exhibited 32% and 61% of heparanase activities at 10 and 33 μM (Table 1), respectively, using the activity assay developed by Hammond and co-workers.^66^ Interestingly, neither peptide **31** nor glycopeptide **1** were able to inhibit heparanase at concentrations up to 100 μM. These results suggested that attaching the glycan to the peptide backbone blocked the inhibitory activities of the glycan.

**Table 1:** Inhibitory activities towards heparanase (5 nM) by glycopeptide **1**, **30** and peptide **31**. (NA denotes that no inhibitions were observed)

Compound #	% of inhibition at		
	3.3 μM	10 μM	33 μM
**1**	NA	NA	NA
**30**	NA	32%	61%
**31**	NA	NA	NA

### Glycan chain and the core peptide could be synergistic in growth factor binding by the glycopeptide

Cytokines and growth factors are important proteins modulating cellular functions, many of which are known to interact with HS.^1,8,34,67^ To probe the impacts of the glycan and the core peptide, the binding of glycopeptide **1**, pentasaccharide **30**, and peptide **31** to several representative growth factors and cytokines including fibroblast growth factor 2 (FGF-2), interleukin 8 (IL-8) and chemokine (C–C motif ligand 13 (CCL-13)) respectively was tested using the biolayer interferometry (BLI). The biotinylated glycopeptide **1**, pentasaccharide **30**, and peptide **31** were immobilized on streptavidin coated biosensors, and their dissociation constants (*K*_D_) with the proteins were measured (Table 2).

**Table 2:** BLI experiments determined dissociation constant of synthetic compounds **1**, **30** and **31** that bind to protein CCL-13, IL-8 and FGF-2. Inactive denotes a *K*_D_ value greater than 400 μM

Compound	*K*_D_ (nm)		
	CCL-13	IL-8	FGF2
**1**	498	28	5
**30**	Inactive	39	14.5
**31**	92	75	17

For CCL-13, glycan **30** did not exhibit much binding, while peptide **31** has a *K*_D_ of 92 nM. The biotin tag itself did not show any significant binding to the protein indicating the binding of **31** resulted from the peptide (data not shown). The glycopeptide **1** bound with CCL-13 with a *K*_D_ of 498 nM, suggesting the glycan interfered with peptide binding with CCL-13. For IL-8, glycopeptide **1** (*K*_D_ = 28 nM) exhibited a modest enhancement in binding compared to glycan **30** and peptide **31** alone (*K*_D_ = 39 and 75 nM respectively). The enhancement was more pronounced for FGF-2, as the binding is strongest for glycopeptide **1** (*K*_D_ = 5 nM), which is about 3-fold higher than pentasaccharide **30** (*K*_D_ = 14.5 nM) and peptide **31** (*K*_D_ = 17 nM). The FGF-2 binding results reveal that the glycan chain and the core peptide can be synergistic in enhancing glycopeptide-protein interactions, in contrast to heparanase inhibition and CCL-13 binding.

### Computation analyses of the interactions of glycopeptide **2** with FGF-2 and heparanase provide insights into the impacts of peptide on HS functions

To better understand the binding of glycopeptide with FGF-2, modeling studies were performed on the FGF-2 complexes with the glycopeptide, peptide and glycan respectively, using crystal structures of the FGF2 protein (PDB^68^ ID: 4OEE).^69^ The potential ligand binding sites on the protein were detected by the Site Finder program implemented in Molecular Operating Environment (MOE). The results showed three potential ligand binding sites on FGF-2 with a positive Propensity of Ligand Binding (PLB) score (Fig. S4†). Glycopeptide **2**, pentasaccharide **28** and peptide **29** structures were docked individually into each of these potential binding sites. Molecular dynamics (MD) simulations and binding free energy calculations were performed on the distinct binding poses with highest GBVI/WSA ΔG scores. The results showed that site 1 had highest affinities for not only glycan **28** also for glycopeptide **2** and peptide **29** (Table S1†). X-ray crystal structure of complexes of FGF-2 and a heparin oligosaccharide showed that the glycan resided in site 1,^69,70^ which is consistent with our computation results.

The average binding energies and their experimental *K*_D_ values for FGF2 are listed in Table S2†, and energies calculated from individual poses can be found in Table S3†. A strong correlation was observed between the experimental and the calculated binding free energy values (Table S2†).

**Fig. 4:**
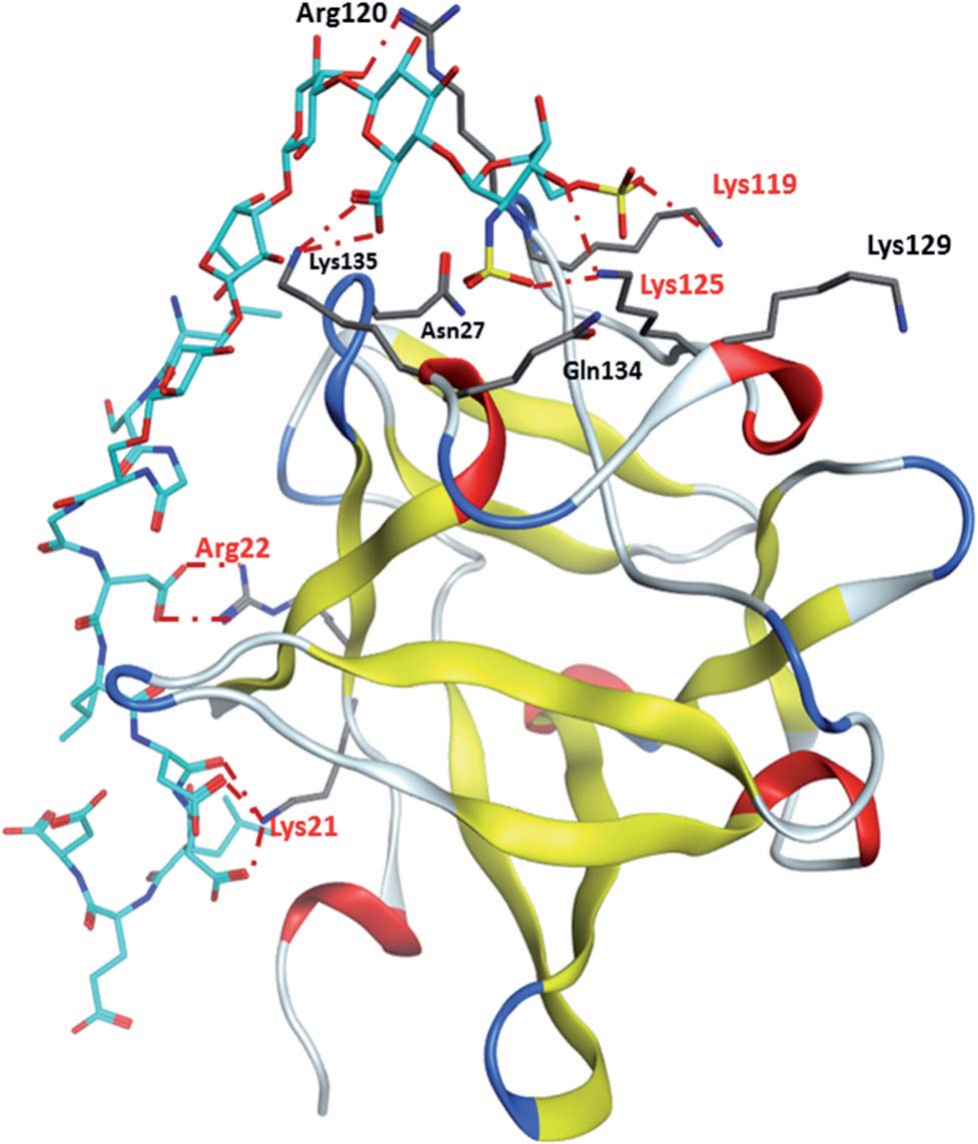
Representative binding poses of glycopeptide **2** with site 1 of FGF2.

Binding site 1 of FGF-2 is lined with many basic residues including Asn27, Arg44, Lys 119, Arg120, Lys125, Lys129, Gln134 and Lys135 (Fig. 4). MD simulations of FGF-2 complex with glycopeptide **2** showed that these residues formed hydrogen bonds with glycopeptide **2**. The distances between the side chains of Lys125 and Lys119 are within 5 Å from the sulfates on the glycan, indicating potential electrostatic interactions. In all glycopeptide poses, the glycan is located within binding site 1 while the peptide extends out of the pocket and towards the protein surface. Glycan **28** was found to reside in binding site 1 with an analogous conformation as that of glycopeptide **2** (Fig. S5†). The peptide portion of glycopeptide **2** extends out of site 1 towards the surface of FGF-2. This leads to the formation of additional salt bridges with the basic residues outside of binding site 1 including Arg22 and Lys21 (Fig. 4). These additional salt bridges are presumably responsible for improved binding to FGF-2 as observed in glycopeptide **2** as compared to glycan **28**.

The binding behavior of glycopeptide **2**, glycan **28** and peptide **29** on heparanase has also been investigated by computational studies. Glycan **28**, peptide **29**, glycopeptide **2**,and the biotin tag were docked into the heparin binding site of the heparanase (PDB ID: 5E9C)^71^ using MOE. The distinct poses with highest GBVI/WSA ΔG scores were further studied with molecular dynamics and binding free energy calculations (Fig. S6†). The average binding energies and energies calculated from individual poses can be found in Table S4.† The binding energy results show that glycan **28** has a higher affinity to heparanase than peptide **29** and glycopeptide **2**, respectively. The biotin tag gave little binding energy with heparanase indicating the major contributor of the binding energy of glycan **30** with heparanase should be from the interactions with the glycan.

**Fig. 5:**
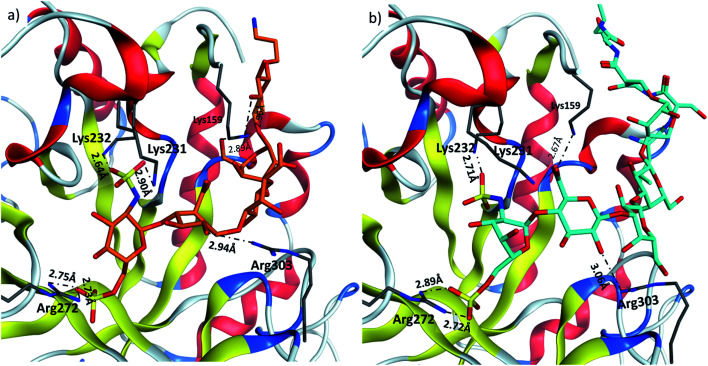
Comparison of (a) glycan **28** and (b) glycopeptide **2** in heparin binding site of heparanase.

Heparanase binding site consists of many basic residues including Lys159, Arg272, Lys231, Lys232, Arg303. Glycan **28** is oriented within the binding site by interacting with these basic residues through hydrogen bonds and ionic bonds (Fig. 5a). In glycopeptide **2** complex with heparanase, the glycan is situated within the binding site, while the peptide is extended toward the solvent (Fig. 5b). The comparison of glycan **28** and glycopeptide **2** shows that core H-bonds and ionic interactions in the binding pocket are weakened in the glycopeptide complex. For example, interaction between Lys231 and N-sulfate group observed in glycan **28**/heparanase is lost in the glycopeptide **2**/heparanase complex. Furthermore, in glycan **28**/heparanase complex *vs.* glycopeptide **2**/heparanase, the distance between Lys232 and N-sulfate group increased from 2.64 Å to 2.71 Å, the distance between Arg272 and O-sulfate group increased from 2.75 Å to 2.89 Å, and H-bond distance between Arg303 and a hydroxyl group increased from 2.94 Å to 3.06 Å (Fig. 5). This weakening of glycan/protein interactions can be explained by the peptide backbone of glycopeptide **2** not fitting in the pocket, thus disrupting the glycan interactions with heparanase, which presumably leads to reduced affinity and inhibitory activity of glycopeptide **2** on heparanase.

## Conclusion

For the first time, HSPG glycopeptides bearing multiple Asp residues in the peptide backbone and O- and N-sulfation on the glycan chain represented by syndecan-4 glycopeptides **1** and **2** have been successfully synthesized. Acceptor reactivity has been found to play a critical role in the synthesis of gal–gal–xyl trisaccharide module. A key factor for the glycopeptide synthesis is the judicious choice of reaction sequences to install O- and N-sulfates as DCV esters into the fully protected glycopeptides bearing multiple aspartic acids. With the significantly enhanced acid stabilities of DCV sulfate esters, *t*Bu esters could be utilized to protect the side chain carboxylic acids of the Asp, which enabled the successful synthesis of glycopeptide **1**.

The availability of well-defined HS glycopeptide such as **1** provided unique opportunities to analyze the roles of the glycan chain and the core peptide in biological functions of the HS glycopeptide. While the glycan **30** inhibited the activities of heparanase, the glycopeptide **1** did not have much an effect on heparanase suggesting the peptide backbone could be antagonistic to glycan functions. In contrast, with the same glycan attached to the same core peptide, the peptide backbone enhanced FGF-2 interactions with the glycan. Molecular modeling results have provided important structural insights on how the peptide backbone impacts HS functions.

While it is well known that HS backbone structure and sulfation pattern can be critical to its biological properties,^8,34,69^ our findings suggest that attaching HS to the core protein as in proteoglycans may be an additional approach to modulate functions. During some biological transformations, HS can be cleaved off HSPGs by enzymes releasing free HS, which may have very different biological properties from the parent HSPG molecules. Understanding the differences between free HS and HS in the context of HSPG can open up a new avenue of investigation into the multi-faceted biological roles of HS.

## Conflicts of interest

There are no conflicts to declare.

## Supplementary Material

SC-011-D0SC01140A-s001
