# Supplementary material for: Chemical synthesis of human syndecan-4 glycopeptide bearing O-, N-sulfation and multiple aspartic acids for probing impacts of the glycan chain and the core peptide on biological functions
Source: Chem Sci. 2020 May 11;11(25):6393–404. doi: 10.1039/d0sc01140a (PMC8159385; doi:10.1039/d0sc01140a)

Electronic Supplementary Material (ESI) for Chemical Science.
This journal is © The Royal Society of Chemistry 2020

Supporting Information

Chemical Synthesis of Syndecan-4 Glycopeptide Bearing *O*-, *N*-sulfation and Multiple Aspartic Acids for Probing Impacts of the Glycan Chain and the Core Peptide on Biological Functions

Weizhun Yang,[1] Yigitcan Eken,[1] Jicheng Zhang,[1] Logan Emerson Cole,[1] Sherif Ramadan,[1,2] Yongmei Xu,[3] Zeren Zhang,[1] Jian Liu,[3] Angela K. Wilson,[1] Xuefei Huang[1,4,5]*

[1]Department of Chemistry, Michigan State University, 578 South Shaw Lane, East Lansing, MI 48824, USA

[2]Chemistry Department, Faculty of Science, Benha University, Benha, Qaliobiya 13518, Egypt

[3]Division of Chemical Biology and Medicinal Chemistry, Eshelman School of Pharmacy, University of North Carolina, Chapel Hill, NC 27599, USA

[4]Department of Biomedical Engineering, Michigan State University, East Lansing, MI 48824, USA

[5]Institute for Quantitative Health Science and Engineering, Michigan State University, East Lansing, MI 48824, USA

*Email: huangxu2@msu.edu

Table of Contents

Experimental procedures and characterization data	S5
Figures S**1-4**	S31
Tables S**1-3**	S33
Figure S**5**	S35
Table S**4**	S37
Figure S**6**	S38
References	S40
^{1}H-NMR ($CDCl_3$, 500 MHz) of **8f**	S41
^{13}C-NMR ($CDCl_3$, 125 MHz) of **8f**	S42
^{1}H-NMR ($CDCl_3$, 500 MHz) of **9b**	S43
^{13}C-NMR ($CDCl_3$, 125 MHz) of **9b**	S44
coupled gHSQC ($CDCl_3$, 500 MHz) of **9b**	S45
^{1}H-NMR ($CDCl_3$, 500 MHz) of **9c-α**	S46
^{13}C-NMR ($CDCl_3$, 125 MHz) of **9c-α**	S47
coupled gHSQC ($CDCl_3$, 500 MHz) of **9c-α**	S48
^{1}H-NMR ($CDCl_3$, 500 MHz) of **9c-β**	S49
^{13}C-NMR ($CDCl_3$, 125 MHz) of **9c-β**	S50
coupled gHSQC ($CDCl_3$, 500 MHz) of **9c-β**	S51
^{1}H-NMR ($CDCl_3$, 500 MHz) of **9d-α**	S52
^{13}C-NMR ($CDCl_3$, 125 MHz) of **9d-α**	S53
coupled gHSQC ($CDCl_3$, 500 MHz) of **9d-α**	S54
^{1}H-NMR ($CDCl_3$, 500 MHz) of **9d-β**	S55
^{13}C-NMR ($CDCl_3$, 125 MHz) of **9d-β**	S56
coupled gHSQC ($CDCl_3$, 500 MHz) of **9d-β**	S57
^{1}H-NMR ($CDCl_3$, 500 MHz) of **9e-α**	S58
coupled gHSQC ($CDCl_3$, 500 MHz) of **9e-α**	S59
^{1}H-NMR ($CDCl_3$, 500 MHz) of **9e-β**	S60
^{13}C-NMR ($CDCl_3$, 125 MHz) of **9e-β**	S61
coupled gHSQC ($CDCl_3$, 500 MHz) of **9e-β**	S62
^{1}H-NMR ($CDCl_3$, 500 MHz) of **9f-α**	S63
coupled gHSQC ($CDCl_3$, 500 MHz) of **9f-α**	S64
^{1}H-NMR ($CDCl_3$, 500 MHz) of **9f-β**	S65
coupled gHSQC ($CDCl_3$, 500 MHz) of **9f-β**	S66
^{1}H-NMR ($CDCl_3$, 500 MHz) of **10**	S67
^{13}C-NMR ($CDCl_3$, 125 MHz) of **10**	S68

gCOSY ($CDCl_3$, 500 MHz) of **10** S69
^{1}H-NMR ($CDCl_3$, 500 MHz) of **11** S70
^{13}C-NMR ($CDCl_3$, 125 MHz) of **11** S71
coupled gHSQC ($CDCl_3$, 500 MHz) of **11** S72
^{1}H-NMR ($CDCl_3$, 500 MHz) of **12** S73
^{13}C-NMR ($CDCl_3$, 125 MHz) of **12** S74
gHSQC ($CDCl_3$, 500 MHz) of **12** S75
^{1}H-NMR ($CDCl_3$, 500 MHz) of **13** S76
^{13}C-NMR ($CDCl_3$, 125 MHz) of **13** S77
coupled gHSQC ($CDCl_3$, 500 MHz) of **13** S78
^{1}H-NMR ($CDCl_3$, 500 MHz) of **14** S79
^{13}C-NMR ($CDCl_3$, 125 MHz) of **14** S80
gHSQC ($CDCl_3$, 500 MHz) of **14** S81
^{1}H-NMR ($CDCl_3$, 500 MHz) of **15** S82
^{13}C-NMR ($CDCl_3$, 125 MHz) of **15** S83
gHSQC ($CDCl_3$, 500 MHz) of **15** S84
gCOSY ($CDCl_3$, 500 MHz) of **15** S85
^{1}H-NMR ($CDCl_3$, 500 MHz) of **17** S86
^{13}C-NMR ($CDCl_3$, 125 MHz) of **17** S87
gHSQC ($CDCl_3$, 500 MHz) of **17** S88
^{1}H-NMR ($CDCl_3$, 500 MHz) of **4** S89
^{13}C-NMR ($CDCl_3$, 125 MHz) of **4** S90
gHSQC ($CDCl_3$, 500 MHz) of **4** S91
^{1}H-NMR ($CDCl_3$, 500 MHz) of **18** S92
^{13}C-NMR ($CDCl_3$, 125 MHz) of **18** S93
^{1}H-NMR ($CDCl_3$, 500 MHz) of **19** S94
^{13}C-NMR ($CDCl_3$, 125 MHz) of **19** S95
^{1}H-NMR ($CDCl_3$, 500 MHz) of **20** S96
^{13}C-NMR ($CDCl_3$, 125 MHz) of **20** S97
^{1}H-NMR ($CDCl_3$, 500 MHz) of **6** S98
^{13}C-NMR ($CDCl_3$, 125 MHz) of **6** S99
^{1}H-NMR ($CDCl_3$, 500 MHz) of **5** S100
^{13}C-NMR ($CDCl_3$, 125 MHz) of **5** S101
^{1}H-NMR ($CDCl_3$, 500 MHz) of **23** S102
^{13}C-NMR ($CDCl_3$, 125 MHz) of **23** S103

gHSQC ($CDCl_3$, 500 MHz) of **23** S104
^{1}H-NMR ($CDCl_3$, 500 MHz) of **3** S105
^{13}C-NMR ($CDCl_3$, 125 MHz) of **3** S106
gHSQC ($CDCl_3$, 500 MHz) of **3** S107
^{1}H-NMR (D_2O, 500 MHz) of **2** S108
gHSQC (D_2O, 500 MHz) of **2** S109
HRMS of **2** S110
^{1}H-NMR (D_2O, 500 MHz) of **1** S111
MS of **1** S112
^{1}H-NMR ($CDCl_3$, 500 MHz) of **25** S113
^{13}C-NMR ($CDCl_3$, 125 MHz) of **25** S114
coupled gHSQC ($CDCl_3$, 500 MHz) of **25** S115
^{1}H-NMR ($CDCl_3$, 500 MHz) of **26** S116
^{13}C-NMR ($CDCl_3$, 125 MHz) of **26** S117
gHSQC ($CDCl_3$, 500 MHz) of **26** S118
^{1}H-NMR ($CDCl_3$, 500 MHz) of **27** S119
^{13}C-NMR ($CDCl_3$, 125 MHz) of **27** S120
gHSQC ($CDCl_3$, 500 MHz) of **27** S121
^{1}H-NMR (D_2O, 500 MHz) of **28** S122
gHSQC (D_2O, 500 MHz) of **28** S123
^{1}H-NMR (D_2O, 500 MHz) of **30** S124
HPLC and HRMS of **29** S125
HPLC of **31** S126

General experimental procedures. All chemical reactions were carried out under nitrogen with anhydrous solvents in flame-dried glassware, unless otherwise noted. Glycosylation reactions were performed in the presence of molecular sieves, which were flame–dried right before the reaction under high vacuum. Glycosylation solvents were dried using a solvent purification system and used directly without further drying. Chemicals used were reagent grade as supplied except where noted. Compounds were visualized by UV light (254 nm) and by staining with a yellow solution containing $Ce(NH_4)_2(NO_3)_6$ (0.5 g) and $(NH_4)_6Mo_7O_{24}\bullet 4H_2O$ (24.0 g) in 6% H_2SO_4 (500 mL). Flash column chromatography was performed on silica gel 60 (230-400 Mesh). NMR spectra were referenced using residual $CHCl_3$ (δ ^{1}H-NMR 7.26 ppm) and $CDCl_3$ (δ ^{13}C-NMR 77.0 ppm). Peak and coupling constants assignments are based on ^{1}H-NMR, ^{1}H-^{1}H gCOSY and/or ^{1}H-^{13}C gHSQC and ^{1}H-^{13}C gHMBC experiments.

Solid-phase peptide synthesis using the Fmoc-strategy:

All amino acids and resins were purchased from Chem-impex. Reaction vessels (10 mL, disposable) and the Domino Block Synthesizer were purchased from Torvig. The peptides were synthesized according to the Fmoc-chemistry based on solid phase peptide synthesis procedure. Resins with pre-loaded amino acid were loaded into a plastic syringe fitted with a filter and swelled in dichloromethane (DCM) for at least 1h. For the coupling reactions, the Fmoc-amino acid (5.0 equiv.) was activated by *O*-(1*H*-benzotriazol-1-yl)-*N*,*N*,*N’*,N’-tetramethyluronium hexafluorophosphate (HBTU) (4.9 equiv.), 1-hydroxybenzotriazole (HOBt) (4.9 equiv.), *N*,*N*-diisopropylethylamine (DIPEA) (10.0 equiv.) and anhydrous dimethylformamide (DMF) (5 mL) for 30 min. Then this mixture containing activated Fmoc amino acid was transferred to the syringe containing the resin, which was then rotated on a tube rotator to mix the solvent with the resins. After completion of coupling, the resin was washed with DCM (3 × 5 mL) and DMF (3 × 5 mL) for 1 minute, each time followed by cleavage of the Fmoc group by treatment of the resin with a solution of piperidine (20%) in DMF for at least 2 × 20 min at r.t.. After every coupling step, unreacted amino groups were capped by treatment with a mixture of Ac_2O (0.5 mL), and DIPEA (1 mL) in DMF (3.5 mL) (capping reagent) for 2 times and 15 min each. After completion of the peptide syntheses, the resin was washed and the peptides were cleaved from the resins by treatment with trifluoroacetic

acid (TFA)/triisopropylsilyl ether (TIPS)/H_2O (95%: 2.5%: 2.5%) solution for 2.5 h. After filtration, the resins were washed with trifluoroacetic acid (2 × 10 mL), and the volume of the combined filtrates was concentrated to 1 mL, then absolute Et_2O (15 mL) at 0 °C was added dropwise to the residues. The precipitates were separated from the mother liquor by centrifugation and washed with cold Et_2O (10 mL). The crude product was dissolved in a solution of piperidine (20%) in DMF. The mixture was stirred at room temperature for 15 min, then absolute Et_2O (15 mL) at 0 °C was added dropwise to the residues. The precipitates were separated from the mother liquor by centrifugation and washed with cold Et_2O (10 mL). The crude product was dissolved in H_2O and subjected to a SUPELCOSILTM LC-18 HPLC column (25 cm × 10 mm, 5 mm) or (25 cm × 4.6 mm, 5 mm) for purification. The solvent systems used were A (0.1% TFA in H_2O) and B (acetonitrile (MeCN) with detection at 220 nm and 254 nm. Mass spectra were obtained by ESI mass spectra (Water Xevo G2-S Q-TOF LC-MS instrument).

General procedure for peptide synthesis in solution phase.

Carboxylic acid (1.1 eq.) and the free amine (1 eq.) were dissolved in DMF (0.125 M). After addition of HATU (1.1 eq.) and DIPEA (3 eq.), the reaction was stirred at room temperature for 1 hour or until TLC indicated the reaction was complete. The reaction mixture was extracted by ethyl acetate and washed by 10% HCl, $NaHCO_3$ (sat) and brine. The organic phase was concentrated, and the mixture was purified by silica gel column chromatography (hexanes-ethyl acetate) to afford the desired product.

General procedure for preactivation based glycosylation.

A solution of donor (0.5 mmol) and freshly activated molecular sieve MS 4 Å (200 mg) in DCM (2 mL) was stirred at room temperature for 30 minutes, and cooled to −78 °C, which was followed by addition of AgOTf (1.5 mmol) dissolved in diethyl ether and DCM without touching the wall of the flask. After 5 minutes, orange colored *p*-TolSCl (0.5 mmol) was added to the solution through a microsyringe. After the donor was completely consumed according to TLC analysis (~ 5 minutes), a solution of acceptor (0.45 mmol) in DCM (0.4 mL) was slowly added dropwise via a syringe. The reaction mixture was warmed to -30 °C under stirring in 1 h. Then the mixture was quenched by Et_3N, diluted with DCM and filtered over Celite. The filtrate was concentrated by vacuo, followed by silica gel flash chromatography.

General procedure for pre-mixed glycosylation.

A solution of donor (0.5 mmol), acceptor (0.45 mmol) and freshly activated molecular sieve MS 4 Å (200 mg) in DCM (2 mL) was stirred at room temperature for 30 minutes, and cooled to −78 °C, which was followed by addition of AgOTf (1.5 mmol) dissolved in diethyl ether and DCM without touching the wall of the flask. After 5 minutes, orange colored *p*-TolSCl (0.5 mmol) was added to the solution through a microsyringe. The reaction mixture was warmed to -30 °C under stirring in 1 h. Then the mixture was quenched by Et_3N, diluted with DCM and filtered over Celite. The filtrate was concentrated by vacuo, followed by silica gel flash chromatography.

General procedure for Fmoc removal.

The Fmoc containing peptide (0.4 mmol) was dissolved in piperidine/DCM (1/4, 5 ml) and the reaction was stirred at room temperature for 15 min. TLC indicated the completion of reaction. Then the mixture was extracted by ethyl acetate and washed by water (twice) and brine. After removal of the organic solvent, the mixture was purified by silica gel column chromatography (MeOH-DCM) to afford the coupling product.

General procedure for Lev removal.

To a solution of a glycan (0.1 mmol) in DCM:CH_3OH (1/1, 4 ml) was added acetic acid (0.4 ml) and 51% hydrazine (0.1 ml). The reaction was stirred at room temperature for 3 h, after which it was diluted with ethyl acetate and washed with sat. $NaHCO_3$ and brine. The organic phase was dried (Na_2SO_4), filtered, and the filtrate was concentrated under reduced pressure. The residue was purified by flash column chromatography.

General procedure for silyl group removal.

To a solution of a glycan (0.1 mmol) in pyridine (3 mL) was added 70% HF•Py (1 mL) at 0 ^oC. The reaction was stirred at room temperature for 3 h, after which it was neutralized by sat. $NaHCO_3$. The mixture was extracted with ethyl acetate and washed with 10% HCl, sat. $NaHCO_3$ and brine. The organic phase was dried (Na_2SO_4), filtered, and the filtrate was concentrated under reduced pressure. The residue was purified by flash column chromatography.

General procedure for oxidation and methylation (from primary alcohol to methyl ester).

TEMPO (0.2 eq.) and BAIB (2.5 eq.) were added into the solution of the alcohol (0.05 M) in DCM/^{t}BuOH/H_2O (4/4/1). The reaction was stirred at room temperature until TLC indicated the

completion of reaction. The reaction mixture was extracted by DCM (3x), and the organic solvent was removed. The residue was dissolved in 1 ml DMF followed by addition of 5 equiv of iodomethane and 3 equiv of potassium bicarbonate. The mixture was stirred at room temperature until TLC indicated the completion of reaction. The mixture was extracted with ethyl acetate and washed with 10% HCl, sat. $NaHCO_3$ and brine. The organic phase was dried (Na_2SO_4), filtered, and the filtrate was concentrated under reduced pressure. The residue was purified by flash column chromatography.

General procedure for Ac protection

To a solution of starting material in pyridine (0.8 ml) was added Ac_2O (0.4 ml). The reaction was stirred at room temperature for 6 hours, then the mixture was extracted with ethyl acetate and washed with 10% HCl, sat. $NaHCO_3$ and brine. The organic phase was dried (Na_2SO_4), filtered, and the filtrate was concentrated under reduced pressure. The residue was purified by flash column chromatography.

General procedure for DCV protection

A solution of 1,2-dimethylimidazole in DCM (10 mg/ml, 8 equiv to 1 OH or NH_2) was added in carbohydrate with free OH or NH_2, then the DCV reagent **16** (6 equiv to 1 OH or NH_2) was added to this mixture at 0^oC. The reaction was stirred for 12 h at room temperature and TLC indicated the completion of reaction. The mixture was directly loaded onto silica gel column for purification.

General procedure for testing DCV sulfate side reactions

A solution of compound **18** (32 mg, 0.05 mmol) in DMF (1 ml) was added the nucleophile (0.1 mmol) and DIPEA (0.03 ml). The reaction was stirred at rt for 6 hours, then the mixture was extracted with ethyl acetate and washed with 10% HCl, sat. $NaHCO_3$ and brine. The organic phase was dried (Na_2SO_4), filtered, and the filtrate was concentrated under reduced pressure. The residue was purified by flash column chromatography

General procedure for DCV deprotection

To a solution of oligosaccharide (5 mg) in DCM/MeOH (1/1, 1.5 ml) was added ammonium formate (3 mg) and $Pd(OH)_2/C$ (5 mg). The resulting mixture was stirred under H_2 atmosphere for 3 h. After filtration and concentration, the residue was purified by a LH-20 column.

General procedure for *O*-sulfation

To a solution of pentasaccharide (15 mg) in DMF (1 ml) was added in $SO_3.Et_3N$ complex (15 mg). The reaction was stirred for 5 h at 40^oC till TLC indicating the completion of reaction. The mixture was directly loaded onto a Sephadex LH-20 column for purification.

General procedure for biotinylation

To a solution of free amine compounds in DMSO/H_2O (1/1, 0.5 ml) was added sulfo-NHS-LC-Biotin (5 equiv) and DIPEA (pH ~ 8.5). The reaction was stirred at room temperature for 5 hours. Then the mixture was loaded onto a G-15 Sephadex column for purification.

General procedure for saponification

To a solution of pentasaccharide (6 mg) in THF/H_2O (2/1, 0.75 ml) at 0^oC were added LiOH (1 M, 0.2 ml) and 30% H_2O_2 (0.1 ml). After stirring for 12 h at rt, the mixture was cooled to 0^oC and MeOH (0.6 ml) and NaOH (4 M, 0.15 ml) were added. The reaction mixture was stirred at rt for 12 h and neutralized with 1 M AcOH. The solution was concentrated *in vacuo* to 1 ml volume, then the residue was purified by a Sephadex G-15 column.

General procedure for global debenzylation

The starting material was dissolved in H_2O (1 ml for 5 mg), to which $Pd(OH)_2/C$ (10 mg for 1 ml) was added. The mixture was placed under a hydrogen balloon and stirred at rt for 16 h. After completion of the reaction, the mixture was filtered through a PTFE syringe filter (0.2 mm, 13 mm). The filtrate was concentrated and the desired product was purified by a Sephadex G-15 column.

General procedure for heparanase assays

Fondaparinux colorimetric heparanase assays were carried out in 96 well microplates (NEST Scientific 96 Well Cell Culture Plate, 701002) pre-treated with a solution of 4% BSA in phosphate-buffered saline containing 0.05% Tween 20 (PBST) for 2 h at 37 °C. Assay solutions (100 ul) contained sodium acetate buffer (40 mM, pH 5.0) and fondaparinux (100 mM, MedChemExpress), heparanase (5 nM) and varying concentrations of compounds of interests or heparin. The mixture was kept at 37 °C for 20 h, which was followed by addition of a solution of WST-1 (100 μL of 1.69 mM WST-1 in 0.1 M NaOH) and incubation at 60 °C for 1 h after which the absorbance at 584 nm was measured (Fluostar Optima plate reader, BMG Labtech).

General procedure for BLI binding assay

The binding assay was performed on the Octet K2 System (Pall ForteBio). The biotinylated

compounds were absorbed to streptavidin (SA) sensor for 2 min. The sensor was then balanced in the assay buffer (PBS containing 0.005% P20) and dipped into protein solution in assay buffer at different concentration. After 5 min of association, the sensor was brought back to the previous assay buffer for a 5 min dissociation step. At the end of the assay, the sensor was regenerated in 2 M NaCl to remove the bound protein. Each measurement was repeated 3 times on the same sensor. The control assay was done with another sensor loaded with saturated biotin solution.

Computational modeling of FGF2 complexes with compounds 2, 28 and 29.

Initial coordinates of FGF2 were obtained from the Protein Data Bank (PDB ID: 4OEE). The disaccharide and solvent structures were eliminated from the initial structure. The N-terminus and the C-terminus of FGF2 were capped and the structure was protonated using the Protonate3D module of MOE. MD trajectories were generated from the protein system without any ligand to relax the structure and minimize the pocket restrictions caused by the present ligand in the structure.

The geometries of compounds **2**, **28** and **29** were optimized using the Gaussian 16 program package. The optimizations were performed using the AM1 method.[1] The obtained Mulliken charges were used with the *antechamber* module of Amber 16 in the generation of parameters. The systems were prepared using the Leap module of AmberTools16[2] under the AMBER ff14SB and GAFF force fields. Each enzyme complex was solvated in a 14 Å cube of TIP4P-Ew water beyond the solute and 100 mM sodium chloride. The systems were relaxed under NVT conditions over six minimization procedures with decreasing restraints on the protein of 500.0, 200.0, 20.0, 10.0, 5.0 kcal/(mol Å^2) to no restraints. The systems were then heated to 300 K over 30 ps. Atomistic molecular dynamics simulations were performed for 30 ns at 300 K and 1 atm using AMBER 16. The SHAKE algorithm constrained bonds involving hydrogen.[3] The trajectories were produced using Langevin dynamics and the pressure of the system was regulated with isotropic position scaling. Long-range electrostatic effects were modeled using the particle-mesh Ewald method with a 10 Å cutoff.

The produced trajectories were analyzed using AMBER 16 and visualized with MOE and the UCSF Chimera package. Free energy of binding was calculated for every picosecond using the Poisson Boltzmann model form the MMPBSA.py module of AmberTools and AMBER 16. The relative free energy trends between models were compared so solute entropy was neglected.

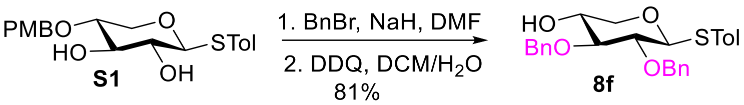

p-Tolyl 2, 3-di-O-benzyl-1-thio-β-D-xylopyranoside (8f)

To a solution of **S1**[4] (376 mg, 1 mmol) in DMF (2 ml) was added NaH (5 mmol, 200 mg) and BnBr (0.35 ml, 3 mmol) in ice bath. The reaction was stirred at rt for 2 hours, then the mixture was extracted with ethyl acetate and washed with 10% HCl, sat. $NaHCO_3$ and brine. The organic phase was dried (Na_2SO_4), filtered, and the filtrate was concentrated under reduced pressure. The residue was purified by flash column chromatography. The resulting compound was dissolved in DCM/H_2O (10:1, 4.4 mL), followed by addition of DDQ (272 mg, 1.2 mmol). The resulting mixture was stirred under room temperature for 4h. After the reaction was complete, it was diluted with DCM, washed with sat. $NaHCO_3$ solution and brine, dried over Na_2SO_4. Silica gel column purification afforded the desired compound **8f** (353 mg, 81% for 2 steps). $[\alpha]_D^{20}$ = -265.5 (*c* 2.0, CH_2Cl_2); ^{1}H NMR (500 MHz, $CDCl_3$): δ. 7.51 (d, J = 8.0 Hz, 2 H), 7.43-7.37 (m, 10 H), 7.20 (d, J = 8.0 Hz, 2 H), 5.17 (d, J = 5.0 Hz, 1 H), 4.88-4.83 (m, 2 H), 4.72-4.68 (m, 2 H), 4.45 (dd, J = 3.0, 11.5 Hz, 1 H), 3.78-3.76 (m, 2 H), 3.70-3.67 (m, 1 H), 3.56 (dd, J = 5.5, 12.0 Hz, 1 H), 3.22 (bs, 1 H), 2.40 (s, 3 H). ^{13}C-NMR (125 MHz, $CDCl_3$): δ.138.0, 137.5, 137.4, 131.9 (2 C), 129.8, 128.7, 128.2, 128.1, 128.0, 127.9, 87.3, 78.9, 78.1, 73.7, 73.6, 68.3, 65.2, 21.2. HRMS: $C_{26}H_{28}O_4S$ $[M + Na]^+$ calcd: 459.1600, obsd: 459.1609.

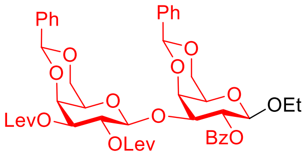

Ethyl 2,3-di-O-levulinoyl-4,6-O-benzylidene-β-D-galactopyranosyl-(1→3)-2-O-benzoyl-4,6-O-benzylidene-β-D-galactopyranoside (9b)

Following the general procedure for pre-mixed glycosylation reaction, compound **9b** (30 mg) was obtained using compound **7** (40 mg, 0.04 mmol) and EtOH in 81% yield. $[\alpha]_D^{20}$ = +100 (*c* 2.6, CH_2Cl_2); ^{1}H NMR (500 MHz, $CDCl_3$): δ. 8.06 (d, J = 8.5 Hz, 2 H), 7.62-7.58 (m, 3 H), 7.50-7.47 (m, 4 H), 7.39-7.32 (m, 6 H), 5.61-5.58 (m, 2 H), 5.46 (s, 1 H), 5.35 (dd, J = 8.0, 9.5 Hz, 1 H), 4.77-4.73 (m, 2 H), 4.60 (d, J = 8.0 Hz, 1 H), 4.47 (d, J = 3.5 Hz, 1 H), 4.36 (d, J = 12.0 Hz, 1 H), 4.26-4.23 (m, 2 H), 4.17 (dd, J = 3.0, 10.0 Hz, 1 H), 4.11 (d, J = 12.0 Hz, 1 H), 4.03 (d, J = 11.5 Hz, 1 H), 3.95-3.90

(m, 1 H), 3.60-3.54 (m, 1 H), 3.51 (s, 1 H), 3.42 (s, 1 H), 2.73-2.55 (m, 5 H), 2.44-2.38 (m, 1 H), 2.33-2.27 (m, 1 H), 2.24-2.18 (m, 1 H), 2.06 (s, 3 H), 1.99 (s, 3 H), 1.10 (t, J = 7.0 Hz, 3 H). ^{13}C-NMR (125 MHz, $CDCl_3$): δ.206.8, 206.7, 172.2, 171.3, 165.0, 137.8, 137.5, 133.2, 130.1, 129.6, 129.1, 128.7, 128.6, 128.2, 128.1, 126.4 (2 C), 100.9 (2 C), 100.8, 100.7, 76.0, 75.9, 73.2, 71.7, 70.9, 68.9, 68.8, 68.3, 66.8, 66.4, 64.5, 37.8, 37.7, 29.7, 29.6, 28.2, 27.5, 15.0. HRMS: $C_{45}H_{50}O_{16}$ [M + Na]$^+$ calcd: 864.3437, obsd: 864.3436.

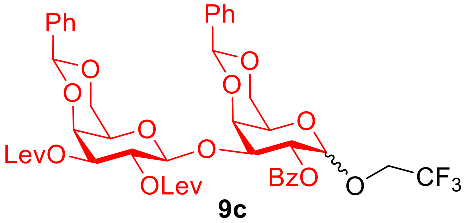

9c

Trifluoroethyl 2,3-di-*O*-levulinoyl-4,6-*O*-benzylidene-β-D-galactopyranosyl-(1→3)-2-*O*-benzoyl-4,6-*O*-benzylidene-α-D-galactopyranoside (9c-α) and Trifluoroethyl 2,3-di-*O*-levulinoyl-4,6-*O*-benzylidene-β-D-galactopyranosyl-(1→3)-2-*O*-benzoyl-4,6-*O*-benzylidene-β-D-galactopyranoside (9c-β)

Following the general procedure for pre-mixed glycosylation reaction, compounds **9c-α** (12 mg) and **9c-β** (7.4 mg) were obtained in 28% and 18% yields respectively using compound **7** (43 mg, 0.05 mmol) and TFE. α isomer: $[\alpha]_D^{20}$ = +86.1 (*c* 0.8, CH_2Cl_2); ^{1}H NMR (500 MHz, $CDCl_3$): δ. 8.07 (d, J = 7.0 Hz, 2 H), 7.64-7.57 (m, 3 H), 7.51-7.49 (m, 4 H), 7.39-7.33 (m, 6 H), 5.65 (s, 1 H), 5.61 (dd, J = 4.0, 11.0 Hz, 1 H), 5.50 (s, 1 H), 5.38-5.34 (m, 2 H), 4.88-4.83 (m, 2 H), 4.58 (d, J = 2.5 Hz, 1 H), 4.44 (dd, J = 3.5, 11.0 Hz, 1 H), 4.35-4.29 (m, 3 H), 4.12 (t, J = 11.5 Hz, 2 H), 4.05-4.01 (m, 1 H), 3.96-3.90 (m, 1 H), 3.82 (s, 1 H), 3.57 (s, 1 H), 2.74-2.63 (m, 2 H), 2.60-2.52 (m, 3 H), 2.30-2.24 (m, 1 H), 2.21-2.15 (m, 1 H), 2.06-1.93 (m, 7 H). ^{13}C-NMR (125 MHz, $CDCl_3$): δ.206.7, 206.4, 172.2, 171.0, 165.7, 137.6, 137.5, 133.6, 129.7, 129.4 (q, J = 42.2 Hz, CF_3), 128.6, 128.2, 128.1, 126.4, 126.2, 101.5, 101.0, 100.5, 98.2, 76.2, 73.3, 72.4, 71.7, 69.9, 68.9, 68.8, 68.5, 66.4, 65.9 (q, J = 32.6 Hz, CH_2CF_3), 37.7, 37.6, 29.6 (2 C), 28.2, 27.4. HRMS: $C_{46}H_{51}F_3O_{16}$ [M + Na]$^+$ calcd: 923.2708, obsd: 923.2695. β isomer: $[\alpha]_D^{20}$ = +19.0 (*c* 0.5, CH_2Cl_2); ^{1}H NMR (500 MHz, $CDCl_3$): δ. 8.05 (d, J = 8.0 Hz, 2 H), 7.63-7.57 (m, 3 H), 7.50-7.47 (m, 4 H), 7.40-7.33 (m, 6 H), 5.67-5.62 (m, 2 H), 5.47

(s, 1 H), 5.36-5.32 (m, 1 H), 4.78-4.74 (m, 3 H), 4.50 (d, J = 2.5 Hz, 1 H), 4.37 (d, J = 12.0 Hz, 1 H), 4.26-4.23 (m, 2 H), 4.17 (dd, J = 1.5, 10.0 Hz, 1 H), 4.14-4.01 (m, 4 H), 3.56 (s, 1 H), 3.44 (s, 1 H), 2.74-2.56 (m, 5 H), 2.44-2.37 (m, 1 H), 2.34-2.28 (m, 1 H), 2.24-2.18 (m, 1 H), 2.07 (s, 3 H), 1.99 (s, 3 H). ^{13}C-NMR (125 MHz, $CDCl_3$): δ.206.7 (2 C), 172.2, 171.3, 165.1, 137.6, 137.4, 133.4, 129.6 (2 C), 129.4 (q, J = 36.7 Hz, CF_3), 128.6, 128.2 (2 C), 126.3 (2 C), 100.9 (2 C), 100.7 (2 C), 75.6 (2 C), 73.2, 71.6, 70.1, 68.8, 68.7, 68.3, 67.3, 66.5, 64.7 (q, J = 37.0 Hz, CH_2CF_3), 37.7 (2 C), 29.7, 29.6, 28.1, 27.4. HRMS: $C_{46}H_{51}F_3O_{16}$ $[M + Na]^+$ calcd: 923.2708, obsd: 923.2703.

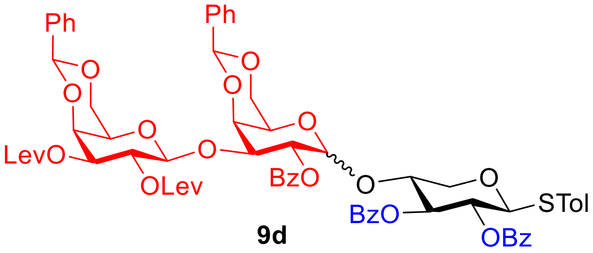

9d

***p*-Tolyl (2,3-di-*O*-levulinoyl-4,6-*O*-benzylidene-β-D-galactopyranosyl)-(1→3)-(2-*O*-benzoyl-4,6-*O*-benzylidene)-(1→4)-2,3-di-*O*-benzoyl-1-thio-α-D-xylopyranoside (9d-α) and *p*-tolyl (2,3-di-*O*-levulinoyl-4,6-*O*-benzylidene-β-D-galactopyranosyl)-(1→3)-(2-*O*-benzoyl-4,6-*O*-benzylidene)-(1→4)-2,3-di-*O*-benzoyl-1-thio-β-D-xylopyranoside (9d-β)**

Following the general procedure for preactivation based glycosylation reaction, compounds **9d-α** (25 mg) and **9d-β** (24 mg) were obtained in 30% and 30% yields respectively using compound **7** (62 mg, 0.07 mmol) and **8d**. α isomer : $[\alpha]_D^{20}$ = +124.0 (*c* 1.8, CH_2Cl_2); ^{1}H NMR (500 MHz, $CDCl_3$): δ. 7.90 (d, J = 7.5 Hz, 2 H), 7.65 (d, J = 7.5 Hz, 2 H), 7.55-7.47 (m, 7 H), 7.43-7.31 (m, 12 H), 7.20-7.11 (m, 6 H), 5.65-5.62 (m, 2 H), 5.49-5.46 (m, 2 H), 5.42 (d, J = 3.5 Hz, 1 H), 5.30 (dd, J = 8.0, 9.5 Hz, 1 H), 5.21 (t, J = 9.0 Hz, 1 H), 4.87 (d, J = 9.0 Hz, 1 H), 4.73 (d, J = 8.0 Hz, 1 H), 4.71 (dd, J = 3.0, 10.5 Hz, 1 H), 4.52 (s, 1 H), 4.40 (dd, J = 5.0, 12.0 Hz, 1 H), 4.34-4.25 (m, 3 H), 4.14-4.03 (m, 3 H), 3.79 (s, 1 H), 3.60 (t, J = 10.5 Hz, 1 H), 3.48 (s, 1 H), 3.36 (s, 1 H), 2.71-2.42 (m, 5 H), 2.35 (s, 3 H), 2.16-2.10 (m, 1 H), 2.01-1.95 (m, 7 H), 1.74-1.69 (m, 1 H). ^{13}C-NMR (125 MHz, $CDCl_3$): δ.206.8, 206.4, 172.1, 171.0, 165.5, 165.3, 165.1, 137.6, 137.5 (2 C), 133.7, 133.2 (2 C), 133.1, 129.8 (2 C), 129.5 (2 C), 129.1, 128.8, 128.4, 128.2 (3 C), 128.1, 126.3, 126.2, 101.4, 100.9, 100.5, 98.7, 87.0, 76.2, 75.3, 74.6, 73.3, 72.2, 71.7, 70.9, 70.1, 68.9, 68.2, 66.4, 63.8, 37.7, 37.6, 29.6, 28.1, 27.1, 21.2. HRMS: $C_{69}H_{68}O_{21}S$ $[M + Na]^+$ calcd: 1282.4312, obsd: 1282.4281. β isomer : $[\alpha]_D^{20}$ = +70.7 (*c* 1.7,

CH_2Cl_2); ^{1}H NMR (500 MHz, $CDCl_3$): δ. 8.01-7.98 (m, 6 H), 7.62 (t, J = 7.5 Hz, 1 H), 7.47-7.27 (m, 20 H), 7.07 (d, J = 8.0 Hz, 2 H), 5.65 (t, J = 7.5 Hz, 1 H), 5.54 (t, J = 9.0 Hz, 1 H), 5.45 (s, 1 H), 5.44 (s, 1 H), 5.31-5.24 (m, 2 H), 4.91 (d, J = 8.0 Hz, 1 H), 4.73-4.69 (m, 2 H), 4.31 (d, J = 3.0 Hz, 1 H), 4.22-4.19 (m, 2 H), 4.11-4.08 (m, 2 H), 3.78-3.71 (m, 2 H), 3.43-3.37 (m, 2 H), 3.16 (s, 1 H), 2.73-2.56 (m, 5 H), 2.42-2.35 (m, 1 H), 2.31-2.25 (m, 4 H), 2.21-2.15 (m, 1 H), 2.04 (s, 3 H), 1.99 (s, 3 H). ^{13}C-NMR (125 MHz, $CDCl_3$): δ.206.7 (2 C), 172.2, 171.3, 165.5, 165.1, 164.7, 138.2, 137.7, 137.4, 133.8, 133.4, 133.2, 133.1, 132.9, 133.0, 129.8, 129.7 (2 C), 129.6, 129.5, 129.3, 129.1, 129.0, 128.7, 128.6, 128.4, 128.2, 128.0, 127.9, 126.6, 126.4, 126.3, 102.3, 100.9, 100.7, 100.6, 86.9, 76.0, 75.6, 73.3, 73.2, 71.6, 71.2, 70.6, 68.7, 68.3, 68.2, 67.1, 66.4, 66.1, 37.7, 29.7, 29.6, 28.1, 27.5, 21.2. HRMS: $C_{69}H_{68}O_{21}S$ $[M + Na]^+$ calcd: 1282.4312, obsd: 1282.4280.

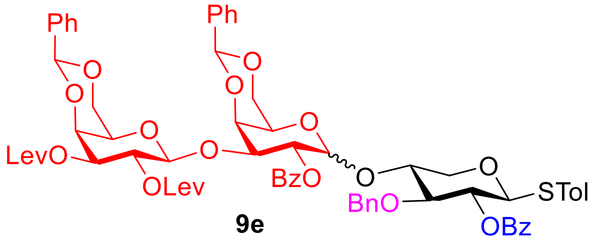

9e

***p*-Tolyl (2,3-di-*O*-levulinoyl-4,6-*O*-benzylidene-β-D-galactopyranosyl)-(1→3)-(2-*O*-benzoyl-4,6-*O*-benzylidene)-(1→4)-2-*O*-benzoyl-3-*O*-benzyl-1-thio-α-D-xylopyranoside (9e-α) and *p*-tolyl (2,3-di-*O*-levulinoyl-4,6-*O*-benzylidene-β-D-galactopyranosyl)-(1→3)-(2-*O*-benzoyl-4,6-*O*-benzylidene)-(1→4)-2-*O*-benzoyl-3-*O*-benzyl-1-thio-β-D-xylopyranoside (9e-β)**

Following the general procedure for preactivation based glycosylation reaction, compounds **9e-α** (48 mg) and **9e-β** (224 mg) were obtained in 13% and 60% yields respectively using compound **7** (200 mg, 0.22 mmol) and **8e**. α isomer: ^{1}H NMR (500 MHz, $CDCl_3$): δ. 7.96-7.94 (m, 4 H), 7.60-7.28 (m, 18 H), 7.09-7.04 (m, 3 H), 6.99 (t, J = 7.5 Hz, 2 H), 6.89 (d, J = 7.0 Hz, 2 H), 5.62-5.59 (m, 2 H), 5.47 (d, J = 4.0 Hz, 1 H), 5.45 (s, 1 H), 5.35 (dd, J = 8.0, 10.5 Hz, 1 H), 5.12-5.11 (m, 1 H), 4.83-4.77 (m, 3 H), 4.56-4.52 (m, 2 H), 4.48 (d, J = 11.5 Hz, 1 H), 4.41-4.36 (m, 2 H), 4.29 (d, J = 12.5 Hz, 1 H), 4.23-4.19 (m, 2 H), 4.10 (d, J = 11.5 Hz, 1 H), 4.00 (d, J = 12.0 Hz, 1 H), 3.85-3.81 (m, 2 H), 3.75 (t, J = 7.5 Hz, 1 H), 3.50 (dd, J = 8.5, 12.0 Hz, 1 H), 3.39 (s, 1 H), 2.72-2.48 (m, 5 H), 2.32 (s, 3 H), 2.26-2.11 (m, 2 H), 2.00 (s, 3 H), 1.98 (s, 3 H). HRMS: $C_{69}H_{70}O_{20}S$ $[M + Na]^+$ calcd: 1273.4073, obsd: 1273.4033. β isomer: $[\alpha]_D^{20}$ = +20.5 (*c* 0.5, CH_2Cl_2); ^{1}H NMR (500 MHz, $CDCl_3$): δ.

8.04-8.01 (m, 4 H), 7.64-7.61 (m, 1 H), 7.57-7.54 (m, 2 H), 7.51-7.47 (m, 4 H), 7.45-7.41 (m, 2 H), 7.40-7.30 (m, 6 H), 7.21-7.20 (m, 2 H), 7.11-7.07 (m, 2 H), 7.03 (d, J = 7.5 Hz, 1 H), 5.64-5.57 (m, 2 H), 5.46 (s, 1 H), 5.34 (dd, J = 8.0, 10.0 Hz, 1 H), 5,16-5.12 (m, 1 H), 4.97 (d, J = 11.5 Hz, 1 H), 4.75-4.73 (m, 2 H), 4.66-4.63 (m, 3 H), 4.25-4.16 (m, 3 H), 4.12 (dd, J = 3.5, 10.5 Hz, 1 H), 4.02 (d, J = 12.0 Hz, 1 H), 3.97-3.93 (m, 2 H), 3.91-3.87 (m, 1 H), 3.75 (t, J = 8.0 Hz, 1 H), 3.41 (s, 1 H), 3.38 (s, 1 H), 3.13 (dd, J = 10.0, 11.5 Hz, 1 H), 2.72-2.54 (m, 6 H), 2.42-2.36 (m, 1 H), 2.28 (s, 3 H), 2.21-2.16 (m, 1 H), 2.04 (s, 3 H), 1.99 (s, 3 H). ^{13}C-NMR (125 MHz, $CDCl_3$): δ. 206.7, 206.6, 172.2 (2 C), 165.1, 164.8, 138.1 (2 C), 138.0, 137.4, 133.5, 133.2, 133.1, 129.9, 129.7, 129.6, 129.5, 128.8, 128.7 (2 C), 128.4, 128.2 (2 C), 128.0 (2 C), 127.4, 126.4, 126.3, 101.8, 100.9, 100.8, 100.6, 86.8, 80.5, 77.9, 77.3, 75.9, 75.8, 74.7, 73.2, 71.6, 71.4, 71.2, 68.8, 68.6, 68.3, 67.0, 66.5, 66.4, 37.7, 29.7, 29.6, 28.1, 27.4, 21.1. HRMS: $C_{69}H_{70}O_{20}S$ $[M + Na]^+$ calcd: 1273.4073, obsd: 1273.4012.

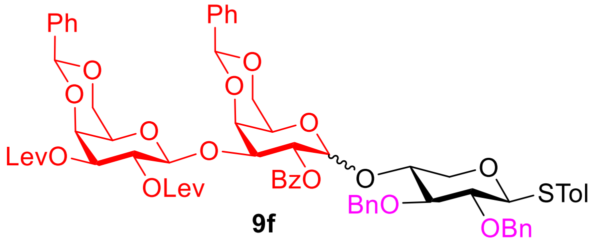

9f

***p*-Tolyl (2,3-di-*O*-levulinoyl-4,6-*O*-benzylidene-β-D-galactopyranosyl)-(1→3)-(2-*O*-benzoyl-4,6-*O*-benzylidene)-(1→4)-2,3-di-*O*-benzyl-1-thio-α-D-xylopyranoside (9f-α) and *p*-tolyl (2,3-di-*O*-levulinoyl-4,6-*O*-benzylidene-β-D-galactopyranosyl)-(1→3)-(2-*O*-benzoyl-4,6-*O*-benzylidene)-(1→4)-2,3-di-*O*-benzyl-1-thio-β-D-xylopyranoside (9f-β)**

Following the general procedure for preactivation based glycosylation reaction, compounds **9f-α** (9 mg) and **9f-β** (58 mg) were obtained in 12% and 75% yields respectively using compound **7** (50 mg, 0.07 mmol) and **8f**. α isomer: ^{1}H NMR (500 MHz, $CDCl_3$): δ. 7.90 (d, J = 8.0 Hz, 2 H), 7.57 (d, J = 6.5 Hz, 2 H), 7.49-7.26 (m, 16 H), 7.22-7.10 (m, 7 H), 6.96 (d, J = 7.0 Hz, 2 H), 5.64-5.61 (m, 2 H), 5.54 (d, J = 3.5 Hz, 1 H), 5.45 (s, 1 H), 5.34 (dd, J = 8.0, 10.5 Hz, 1 H), 4.84 (d, J = 8.0 Hz, 1 H), 4.78 (dd, J = 3.5, 10.5 Hz, 1 H), 4.70 (d, J = 10.5 Hz, 1 H), 4.64 (d, J = 9.0 Hz, 1 H), 4.58 (s, 2 H), 4.54-4.51 (m, 2 H), 4.42 (dd, J = 3.0, 10.5 Hz, 1 H), 4.27-4.20 (m, 4 H), 4.09 (d, J = 12.5 Hz, 1 H), 4.00 (d, J = 12.0 Hz, 1 H), 3.85-3.81 (m, 1 H), 3.76 (s, 1 H), 3.59 (t, J = 8.0 Hz, 1 H), 3.39-3.31 (m, 4 H), 2.71-2.48 (m, 5 H), 2.33 (s, 3 H), 2.26-2.20 (m, 1 H), 2.13-2.07 (m, 1 H), 2.00 (s, 3 H), 1.97 (s, 3 H), 1.92-1.86 (m, 1 H). β isomer: ^{1}H NMR (500 MHz, $CDCl_3$): δ. 8.03 (d, J = 7.5 Hz, 2 H),

7.64-7.57 (m, 3 H), 7.51-7.45 (m, 6 H), 7.40-7.21 (m, 16 H), 7.06 (d, J = 8.0 Hz, 2 H), 5.61 (s, 1 H), 5.60 (dd, J = 8.0, 10.0 Hz, 1 H), 5.46 (s, 1 H), 5.35 (dd, J = 8.0, 10.5 Hz, 1 H), 5.12 (d, J = 10.0 Hz, 1 H), 4.79-4.65 (m, 6 H), 4.46 (d, J = 3.0 Hz, 1 H), 4.43 (d, J = 9.5 Hz, 1 H), 4.26-4.21 (m, 3 H), 4.14 (dd, J = 3.0, 10.0 Hz, 1 H), 4.02 (s, 1 H), 3.99 (s, 1 H), 3.85-3.80 (m, 2 H), 3.60 (t, J = 9.5 Hz, 1 H), 3.41-3.31 (m, 3 H), 2.96 (t, J = 12.5 Hz, 1 H), 2.73-2.67 (m, 1 H), 2.64-2.54 (m, 4 H), 2.43-2.37 (m, 1 H), 2.32-2.26 (m, 4 H), 2.23-2.18 (m, 1 H), 2.04 (s, 3 H), 1.99 (s, 3 H).

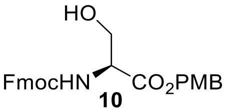

10

N-Fluorenylmethyloxycarbonyl-L-serine *p*-methoxybenzyl ester (10)

To a solution of Fmoc-L-serine (655 mg, 2 mmol) in DMF (2.5 ml) was added K_2CO_3 (3 mmol, 414 mg) and PMBCl (0.4 ml, 3 mmol). The reaction was stirred at rt for 5 hours, then the mixture was extracted with ethyl acetate and washed with 10% HCl, sat. $NaHCO_3$ and brine. The organic phase was dried (Na_2SO_4), filtered, and the filtrate was concentrated under reduced pressure. The residue was purified by flash column chromatography (hexanes : ethyl acetate = 2 : 1) to afford **10** in 74% yield. $[\alpha]_D^{20}$ = -14.0 (*c* 0.3, CH_2Cl_2); ^{1}H NMR (500 MHz, $CDCl_3$): δ. 7.77 (d, J = 7.5 Hz, 2 H), 7.60 (d, J = 7.0 Hz, 2 H), 7.42 (t, J = 7.5 Hz, 2 H), 7.32-7.27 (m, 4 H), 6.87 (d, J = 8.0 Hz, 2 H), 5.81 (d, J = 7.5 Hz, 1 H), 5.15 (s, 2 H), 4.47-4.36 (m, 3 H), 4.22 (t, J = 6.5 Hz, 1 H), 4.01 (d, J = 9.5 Hz, 1 H), 3.92 (d, J = 10.0 Hz, 1 H), 3.78 (s, 3 H), 2.32 (bs, 1 H). ^{13}C-NMR (125 MHz, $CDCl_3$): δ. 170.4, 159.8, 156.2, 143.8, 143.6, 141.3 (2 C), 130.1, 127.7, 127.2, 127.1 (2 C), 125.1, 120.0 (2 C), 114.0, 67.5, 67.2, 63.3, 56.2, 55.3, 47.1. HRMS: $C_{26}H_{25}NO_6$ $[M + Na]^+$ calcd: 470.1574, obsd: 470.1582.

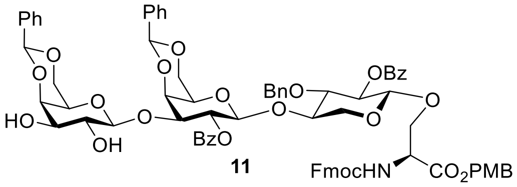

11

N-Fluorenylmethyloxycarbonyl-*O*-[4,6-*O*-benzylidene-β-D-galactopyranosyl-(1→3)-2-*O*-benzoyl-4,6-*O*-benzylidene-β-D-galactopyranosyl-(1→4)-2-*O*-benzoyl-3-*O*-benzyl-β-D-xylopyranosyl]-L-serine *p*-methoxybenzyl ester (11)

A solution of donor **9e-β**(200 mg, 0.16 mmol), acceptor **10** (65 mg, 0.15 mmol) and freshly activated

molecular sieve MS 4 Å (100 mg) in DCM (1.5 mL) was stirred at room temperature for 30 minutes, and cooled to −78 °C, which was followed by addition of AgOTf (123 mg, 0.48 mmol) dissolved in acetonitrile (0.1 mL) without touching the wall of the flask. After 5 minutes, orange colored *p*-TolSCl (23 µL, 0.16 mmol) was added to the solution through a microsyringe. The reaction mixture was warmed to -30 °C under stirring in 1 h. Then the mixture was quenched by DIPEA, diluted with DCM and filtered over Celite. The filtrate was concentrated *in vacuo*, followed by silica gel flash chromatography (toluene : acetone = 4 : 1) to afford trisaccharide serine in 85% yield. After that, following the general procedure for Lev removal, trisaccharide **11** was obtained in 75% yield. $[\alpha]_D^{20}$ = -1.2 (*c* 0.3, CH_2Cl_2); ^{1}H NMR (500 MHz, $CDCl_3$): *δ*. 8.07 (d, J = 7.0 Hz, 2 H), 7.99 (d, J = 7.0 Hz, 2 H), 7.78-7.76 (m, 2 H), 7.58-7.55 (m, 5 H), 7.50-7.45 (m, 5 H), 7.41-7.31 (m, 10 H), 7.19-7.12 (m, 5 H), 6.83 (d, J = 7.0 Hz, 2 H), 5.70 (t, J = 8.0 Hz, 1 H), 5.61 (s, 1 H), 5.57 (d, J = 8.0 Hz, 1 H), 5.51 (s, 1 H), 5.17 (t, J = 7.0 Hz, 1 H), 5.03-4.99 (m, 3 H), 4.79-4.75 (m, 2 H), 4.46-4.41 (m, 2 H), 4.35-4.32 (m, 3 H), 4.27-4.17 (m, 4 H), 4.13-3.97 (m, 4 H), 3.88 (d, J = 12.0 Hz, 1 H), 3.77-3.71 (m, 5 H), 3.46 (s, 1 H), 3.41 (d, J = 9.5 Hz, 1 H), 3.35 (s, 1 H), 3.14 (t, J = 10.0 Hz, 1 H), . ^{13}C-NMR (125 MHz, $CDCl_3$): *δ*. 169.5, 165.8, 165.2, 159.6, 155.9, 143.9, 143.7, 141.2 (2 C), 138.2, 137.9, 137.5, 133.5, 133.2, 130.1, 129.8, 129.6 (2 C), 129.3, 129.0, 128.7, 128.5, 128.3, 128.2 (2 C), 128.1, 127.8, 127.7, 127,4 (2 C), 127.1, 126.6, 126.3, 125.2 (2 C), 120.0, 119.9, 133.9, 104.3, 101.6, 101.2, 79.2, 77.8 (2 C), 77.3, 76.0, 75.0, 74.4, 72.3, 72.0, 71.6, 71.0, 69.1, 69.0, 68.7, 67.2, 67.0, 66.9, 66.8, 62.9, 55.3, 54.4, 47.0. HRMS: $C_{78}H_{75}NO_{22}$ $[M + NH_4]^+$ calcd: 1395.5119, obsd: 1395.5065.

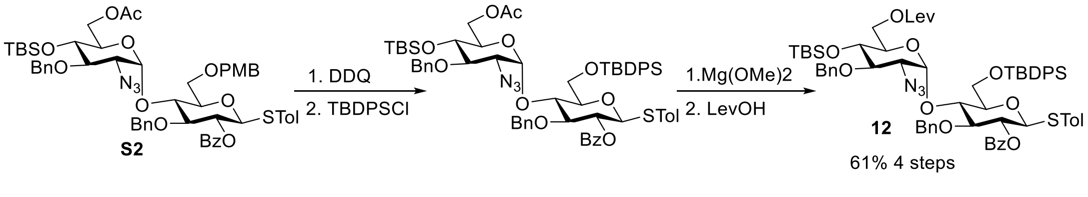

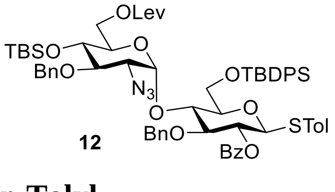

12

***p*-Tolyl 2-azido-3-*O*-benzyl-4-*O*-tert-butyl-dimethylsilyl-6-*O*-levulinoyl-2-deoxy-α-D-glucopyranosyl-(1 →4)-2-*O*-benzoyl-3-*O*-benzyl-6-*O*-*tert*-butyldiphenylsilyl-1-thio-β-D-glucopyranoside (12)**

Compound **S2**[5] (470 mg, 0.45 mmol) was dissolved in DCM/H_2O (10:1, 7.7 mL), followed by addition of DDQ (114 mg, 0.5 mmol). The resulting mixture was stirred under room temperature for 4h. After the reaction was complete, it was diluted with DCM, washed with sat. $NaHCO_3$ solution and brine, dried over Na_2SO_4. Silica gel column purification afforded the desired compound (378 mg, 91%). The resulting compound was dissolved in DCM (3.5 mL), followed by addition of imidazole (79 mg, 1.2 mmol) and TBDPSCl (0.16 ml, 0.6 mmol). The resulting mixture was stirred under room temperature overnight and directly purified by column (93% yield). The resulting compound (410 mg) was dissolved in dry DCM (8 mL) and cooled down to 0 ºC. $Mg(OMe)_2$ solution (8%) (3.5 mL) was added to the reaction mixture. The resulting mixture was left at room temperature and the reaction was monitored by TLC. After the reaction was complete, it was extracted with ethyl acetate and washed with 10% HCl, sat. $NaHCO_3$ and brine. The organic phase was dried (Na_2SO_4), filtered, and the filtrate was concentrated under reduced pressure. The residue was purified by flash column chromatography (89% yield). The resulting compound (500 mg), LevOH (2 equiv.), EDC-HCl (3 equiv) and DMAP (1 equiv.) were dissolved in DCM (3 mL). After overnight stirring under room temperature, the reaction mixture was extracted with ethyl acetate and washed with 10% HCl, sat. $NaHCO_3$ and brine. The organic phase was dried (Na_2SO_4), filtered, and the filtrate was concentrated under reduced pressure. The residue was purified by flash column chromatography to afford disaccharide **12** (81% yield). $[\alpha]_D^{20}$ = +1.6 (*c* 0.2, CH_2Cl_2); ^1H NMR (500 MHz, $CDCl_3$): δ. 8.10 (d, J = 7.5 Hz, 2 H), 7.77-7.75 (m, 4 H), 7.61-7.58 (m, 1 H), 7.49-7.35 (m, 15 H), 7.32-7.29 (m, 1 H), 7.22-7.14 (m, 5 H), 6.98 (d, J = 8.0 Hz, 2 H), 5.61 (d, J = 4.0 Hz, 1 H), 5.39 (t, J = 9.0 Hz, 1 H), 4.89-4.70 (m, 6 H), 4.12-3.95 (m, 4 H), 3.90-3.84 (m, 2 H), 3.73-3.70 (m, 1 H), 3.56-3.50 (m, 3 H), 3.18-3.15 (m, 1 H), 2.64-2.61 (m, 2 H), 2.40-2.37 (m, 2 H), 2.29 (s, 3 H), 2.16 (s, 3 H), 1.13 (s, 9 H), 0.87 (s, 9 H), -0.01 (s, 3 H), -0.03 (s, 3 H). ^{13}C-NMR (125 MHz, $CDCl_3$): δ. 206.2, 172.3, 165.3, 137.9, 137.5, 137.3, 135.7, 135.6, 133.4, 133.2, 131.9, 130.4, 129.9, 129.8 (2 C), 129.7, 128.5, 128.3 (2 C), 127.9, 127.8 (2 C), 127.7, 127.6, 127.2, 97.7, 87.3, 84.8, 80.0, 79.9, 75.0, 74.5, 74.1, 73.0, 71.3, 71.2, 64.4, 63.5, 62.9, 37.8, 29.9, 27.6, 27.0, 26.0, 21.1, 19.4, 18.0. HRMS: $C_{67}H_{81}N_3O_{12}SSi_2$ $[M + NH_4]^+$ calcd: 1225.5418, obsd: 1225.5365.

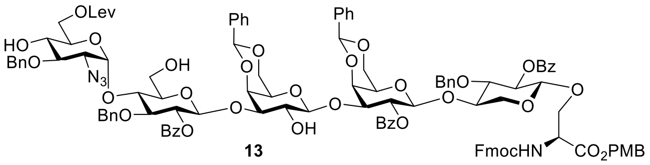

13

N-Fluorenylmethyloxycarbonyl-*O*-[2-azido-3-*O*-benzyl-6-*O*-levulinoyl-2-deoxy-α-D-glucopyranosyl-(1→4)-2-*O*-benzoyl-3-*O*-benzyl-β-D-glucopyranosyl-(1→3)-4,6-*O*-benzylidene-β-D-galactopyranosyl-(1→3)-2-*O*-benzoyl-4,6-*O*-benzylidene-β-D-galactopyranosyl-(1→4)-2-*O*-benzoyl-3-*O*-benzyl-β-D-xylopyranosyl]-L-serine *p*-methoxybenzyl ester (13)

A mixture of acceptor **11** (200 mg, 0.145 mmol), donor **12** (210 mg, 0.174 mmol), and 4 Å molecular sieves in DCM (3 ml) was stirred at room temperature for 30 min. Then it was cooled to -40 ^{o}C followed by the addition of *N*-iodosuccinimide (46 mg, 0.2 mmol) and TfOH (0.02 mmol). The reaction was stirred at -20 ^{o}C for 1 hours, after which it was quenched by DIPEA and filtered through celite, and the filtrate was concentrated under reduced pressure. The residue was purified by flash column chromatography (toluene : acetone = 10 : 1) to afford pentasaccharide. Following the general procedure of silyl group removal, compound **13** was obtained in 80% yield for two steps. $[\alpha]_D^{20}$ = +3.3 (*c* 0.3, CH_2Cl_2); ^{1}H NMR (500 MHz, $CDCl_3$): *δ*. 8.08 (d, J = 7.5 Hz, 2 H), 8.00-7.98 (m, 4 H), 7.79 (t, J = 7.0 Hz, 1 H), 7.63 (t, J = 7.5 Hz, 1 H), 7.58-7.49 (m, 7 H), 7.45-7.38 (m, 9 H), 7.36-7.28 (m, 9 H), 7.27-7.25 (m, 2 H), 7.22-7.18 (m, 5 H), 7.14-7.11 (m, 2 H), 6.84 (d, J = 8.5 Hz, 2 H), 5.67 (dd, J = 8.5, 10.5 Hz, 1 H), 5.62 (d, J = 4.0 Hz, 1 H), 5.52 (s, 1 H), 5.44 (s, 1 H), 5.32 (t, J = 8.0 Hz, 1 H), 5.16-5.13 (m, 1 H), 5.03-4.99 (m, 2 H), 4.95-4.90 (m, 2 H), 4.76-4.74 (m, 3 H), 4.54 (dd, J = 4.5, 12.0 Hz, 1 H), 4.41-4.31 (m, 5 H), 4.25-4.17 (m, 5 H), 41.13-4.11 (m, 2 H), 4.08-3.93 (m, 6 H), 3.86-3.71 (m, 10 H), 3.55-3.48 (m, 2 H), 3.44-3.42 (m, 2 H), 3.33-3.30 (m, 1 H), 3.24 (dd, J = 4.5, 10.5 Hz, 1 H), 3.14-3.08 (m, 2 H), 2.76-2.72 (m, 2 H), 2.61-2.57 (m, 2 H), 2.16 (s, 3 H). ^{13}C-NMR 125 MHz, $CDCl_3$): *δ*. 207.1, 173.6, 169.4, 165.6, 165.2 (2 C), 159.6, 155.9, 143.9, 143.7, 141.3, 141.2, 138.2, 138.0, 137,8, 137.6, 137.5, 133.4, 133.2, 130.1, 129.9 (2 C), 129.8 (2 C), 129.7, 129.6, 129.0, 128.7, 128.6 (2 C), 128.5, 128.4, 128.3, 128.2, 128.1 (4 C), 127.8 (2 C), 127.7, 127.4 (2 C), 127.2, 127.1 (2 C), 126.6, 126.0, 125.2 (2 C), 120.0, 119.9, 113.9, 104.1, 101.6, 101.3, 101.2, 100.3, 97.7, 83.3, 79.2 (2 C), 77.8, 77.3, 75.9, 75.5, 75.2, 74.7, 74.4, 74.3, 74.0, 72.3, 71.6, 71.0, 79.7, 69.9, 69.0, 68.9, 68.6, 67.2, 67.0, 66.9 (2 C), 63.3, 62.9, 62.6, 61.7, 55.3, 54.4, 47.1, 37.9, 29.8, 27.8. HRMS: $C_{116}H_{116}N_4O_{34}$ $[M + 2 NH_4]^{2+}$ calcd: 1072.4074, obsd: 1072.4041.

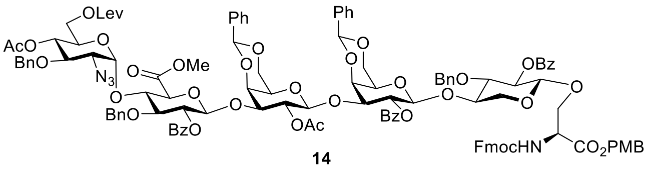

14

N-Fluorenylmethyloxycarbonyl-*O*-[2-azido-3-*O*-benzyl-4-acetyl-6-*O*-levulinoyl-2-deoxy-α-D-glucopyranosyl-(1→4)-methyl 2-*O*-benzoyl-3-*O*-benzyl-β-D-glucopyranosyluronate-(1→3)-4,6-*O*-benzylidene-β-D-galactopyranosyl-(1→3)-2-*O*-benzoyl-4,6-*O*-benzylidene-β-D-galactopyranosyl-(1→4)-2-*O*-benzoyl-3-*O*-benzyl-β-D-xylopyranosyl]-L-serine *p*-methoxybenzyl ester (14)

Following the general procedures for oxidation, methylation and Ac protection, compound **14** was obtained in 75% yield. $[\alpha]_D^{20}$ = +3.2 (*c* 0.2, CH_2Cl_2); ^{1}H NMR (500 MHz, $CDCl_3$): *δ*. 8.09 (d, J = 7.5 Hz, 2 H), 7.98-7.95 (m, 4 H), 7.78-7.75 (m, 2 H), 7.61-7.31 (m, 27 H), 7.23-7.10 (m, 11 H), 6.83 (d, J = 8.5 Hz, 2 H), 5.63 (dd, J = 8.0, 10.0 Hz, 1 H), 5.54-5.51 (m, 2 H), 5.44 (d, J = 3.5 Hz, 1 H), 5.40 (s, 1 H), 5.31-5.26 (m, 2 H), 5.12-5.09 (m, 1 H), 5.01-4.94 (m, 4 H), 4.83 (d, J = 11.0 Hz, 1 H), 4.72-4.61 (m, 6 H), 4.39-4.31 (m, 4 H), 4.27-4.08 (m, 9 H), 4.00-3.91 (m, 5 H), 3.77-3.67 (m, 8 H), 3.60-3.58 (m, 1 H), 3.38 (s, 1 H), 3.33-3.30 (m, 2 H), 2.78-2.71 (m, 2 H), 2.67-2.58 (m, 2 H), 2.17 (s, 3 H), 1.96 (s, 3 H), 1.58 (s, 3 H). ^{13}C-NMR (125 MHz, $CDCl_3$): *δ*. 206.6, 172.5, 169.3, 169.1, 169.0, 165.2, 164.7, 159.6, 155.8, 143.9, 143.7, 141.2, 138.2, 138.0, 137.5, 137.3, 137.1, 133.5, 133.4, 133.1, 133.0, 130.1, 129.8, 129.7, 129.6, 129.3, 128.8, 128.7, 128.6, 128.5 (2 C), 128.4 (3 C), 128.2, 128.1 (3 C), 128.0 (2 C), 127.8 (3 C), 127.7, 127.4, 127.3, 127.1 (2 C), 126.4, 126.1, 125.2 (2 C), 120.0, 119.9, 113.9, 101.6, 100.9, 100.6, 100.5, 99.8, 97.3, 82.5, 79.4, 77.9, 77.3, 75.6, 75.5, 74.7, 74.5, 73.8 (2 C), 72.9, 72.4, 71.4, 69.6, 69.2, 69.1, 68.8, 68.7, 68.6, 67.2, 67.0, 66.9, 66.7, 63.0, 62.7, 61.7, 55.3, 52.6, 47.1, 37.9, 29.8, 27.9, 20.8, 20.2. HRMS: $C_{121}H_{120}N_4O_{37}$ $[M + 2\ NH_4]^{2+}$ calcd: 1128.4154, obsd: 1128.4132.

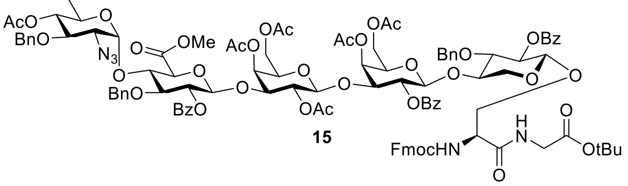

15

Compound **14** (30 mg) was dissolved in TFA/H_2O/TIPS (95/2.5/2.5, 2 ml), and the solution was

stirred at rt for 1 hour. The reaction was concentrated followed by purification by Sephadex LH 20 column (MeOH/DCM = 1/1). The resulting carboxylic acid was coupled with glycine *t*Bu ester followed by acetylation following the general procedures for peptide synthesis in solution phase and Ac protection to afford compound **15** in 81% yield for three steps. $[\alpha]_D^{20}$ = +3.5 (*c* 0.2, CH_2Cl_2); ^1H NMR (500 MHz, $CDCl_3$): δ. 8.01-7.93 (m, 6 H), 7.77-7.75 (m, 2 H), 7.63-7.54 (m, 5 H), 7.51-7.47 (m, 2 H), 7.45-7.28 (m, 14 H), 6.79 (bs, 1 H), 5.72 (bs, 1 H), 5.41-5.38 (m, 3 H), 5.17 (t, J = 6.5 Hz, 1 H), 5.12-5.09 (m, 1 H), 5.05-4.98 (m, 3 H), 4.85 (d, J = 11.5 Hz, 1 H), 4.81 (d, J =11.5 Hz, 1 H), 4.71-4.60 (m, 6 H), 4.42 (d, J = 8.0 Hz, 1 H), 4.33-4.27 (m, 3 H), 4.22-4.19 (m, 1 H), 4.17-4.07 (m, 5 H), 4.03-3.99 (m, 2 H), 3.96-3.83 (m, 8 H), 3.80 (s, 3 H), 3.74-3.63 (m, 6 H), 3.38 (dd, J = 3.5, 11.0 Hz, 1 H), 2.78-2.72 (m,2 H), 2.64-2.59 (m, 2 H), 2.17 (s, 3 H), 2.09 (s, 3 H), 2.08 (s, 3 H), 2.02 (s, 3 H), 2.00 (s, 3 H), 1.95 (s, 3 H), 1.44 (s, 3 H), 1.42 (s, 3 H). ^{13}C-NMR (125 MHz, $CDCl_3$): δ. 206.5, 172.5, 170.6, 170.2, 169.8, 169.3, 168.6, 168.4, 168.3, 165.5, 164.6, 164.5, 143.7, 141.2, 138.0, 137.3, 137.0, 133.5 (2 C), 133.3, 133.0, 129.8 (2 C), 129.7, 129.5, 129.4, 129.2, 128.8, 128.5 (2 C), 128.4 (2 C), 128.1 (2 C), 128.0 (2 C), 127.9, 127.8, 127.7, 127.6, 127.1, 125.1, 120.0, 101.2, 100.9, 100.8, 99.8, 97.5, 82.1, 77.5, 77.3, 76.9, 76.0, 75.9, 74.8, 74.7, 73.9 (2 C), 73.6, 72.7, 71.6 (2 C), 71.4, 70.1, 69.5, 69.2, 69.0, 68.7, 68.2, 67.2, 64.0, 62.8, 62.4, 62.1, 62.0, 61.6, 53.6, 52.7, 47.0, 42.1, 37.9, 29.8, 28.1, 28.0, 27.9, 19.8. HRMS: $C_{113}H_{123}N_5O_{41}$ $[M + 2\ Na]^{2+}$ calcd: 1125.8739, obsd: 1125.8712.

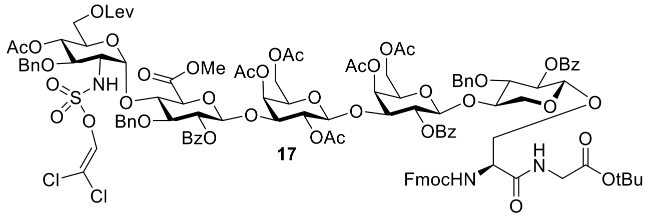

Compound **15** (22 mg, 0.01 mmol) was dissolved in THF/AcOH (3:1, 1.2 mL), followed by addition of Zn (60 mg). The resulting mixture was stirred under room temperature for 1 hour. After filtration and concentration by vacuum, the mixture was loaded onto sephadex LH-20 column (MeOH/DCM, 1/1) for purification. The resulting amine was protected with DCV following the general procedure for DCV protection to produce compound **17** (71%, 2 steps). $[\alpha]_D^{20}$ = +0.3 (*c* 0.1, CH_2Cl_2); ^1H NMR (500 MHz, $CDCl_3$): δ. 8.01-7.93 (m, 6 H), 7.77-7.74 (m, 2 H), 7.62-7.60 (m, 2 H), 7.57-7.54 (m, 3

H), 7.50-7.39 (m, 10 H), 7.34-7.27 (m, 8 H), 7.19-7.14 (m, 6 H), 6.91 (s, 1 H), 6.79 (bs, 1 H), 6.21 (bs, 1 H), 5.74 (bs, 1 H), 5.43-5.38 (m, 3 H), 5.28 (d, J = 3.5 Hz, 1 H), 5.23 (t, J = 6.0 Hz, 1 H), 5.13-5.08 (m, 2 H), 5.04-4.99 (m, 2 H), 4.85 (d, J = 11.5 Hz, 1 H), 4.76-4.72 (m, 2 H), 4.67 (d, J = 11.5 Hz, 1 H), 4.62 (d, J = 7.5 Hz, 1 H), 4.57 (d, J = 12.0 Hz, 1 H), 4.52-4.50 (m, 1 H), 4.45-4.27 (m, 8 H), 4.16-4.03 (m, 8 H), 3.96-3.60 (m, 20 H), 3.54-3.50 (m, 2 H), 3.36 (s, 1 H), 3.14 (bs,1 H), 2.78-2.60 (m, 4 H), 2.18 (3 H), 2.09 (s, 6 H), 2.08 (s, 3 H), 2.02 (s, 3 H), 1.89 (s, 3 H), 1.49 (s, 3 H), 1.42 (s, 9 H). ^{13}C-NMR (125 MHz, $CDCl_3$): *δ*. 206.6, 172.5, 170.6 (2 C), 170.3, 169.8, 169.4, 169.2, 168.7, 168.3, 167.7, 165.5, 164.6, 164.4, 143.7, 141.2, 138.0, 137.5, 135.7, 133.8, 133.7, 133.6, 133.3, 133.0, 129.8 (2 C), 129.7 (2 C), 129.5, 129.4, 128.9, 128.8, 128.7 (2 C), 128.7, 128.5, 128.4, 128.3, 128.1, 128.0, 127.9, 127.7 (2 C), 127.6, 127.1, 125.1, 120.0, 115.4, 114.1, 101.2, 100.9, 100.8, 99.6, 99.3, 82.1, 79.3, 78.0, 77.7, 76.9, 75.9, 75.3, 74.9, 74.8, 73.9, 72.9, 72.6, 72.1, 71.6, 71.3, 70.3, 69.6, 69.3, 69.2, 69.0, 68.2, 67.2, 64.0, 62.4, 62.1, 61.9, 61.5, 58.5, 56.0, 53.6, 53.0, 47.0, 42.1, 37.9, 29.8, 28.1, 28.0, 27.8, 20.8 (2 C), 20.7, 20.6 (2 C), 19.9. HRMS: $C_{115}H_{125}Cl_2N_3O_{44}S$ $[M + 2 Na]^{2+}$ calcd: 1199.8259, obsd: 1199.8269.

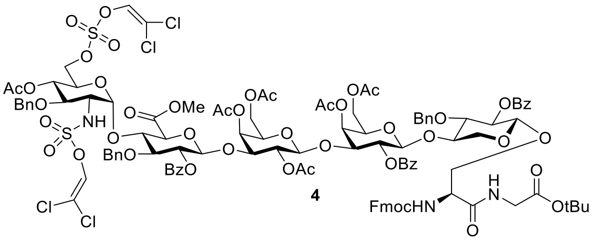

4

Following general procedure for Lev removal and DCV protection, compound **4** was obtained from **17** in 51% yield. $[\alpha]_D^{20}$ = +5.7 (*c* 0.3, CH_2Cl_2); ^{1}H NMR (500 MHz, $CDCl_3$): *δ*. 8.04-7.99 (m, 6 H), 7.94-7.93 (m, 3 H), 7.76-7.74 (m, 2 H), 7.63-7.59 (m, 2 H), 7.56-7.53 (m, 4 H), 7.49-7.38 (m, 11 H), 7.34-7.29 (m, 4 H), 7.25-7.22 (m, 4 H), 7.18-7.14 (m, 4 H), 7.08 (s, 1 H), 6.89 (s, 1 H), 6.78 (bs, 1 H), 5.73 (d, J = 6.5 Hz, 1 H), 5.46 (d, J = 3.5 Hz, 1 H), 5.43-5.40 (m, 2 H), 5.33 (d, J = 3.5 Hz, 1 H), 5.21 (t, J = 4.5 Hz, 1 H), 5.12-5.97 (m, 6 H), 4.94 (d, J = 9.5 Hz, 1 H), 5.84 (d, J = 11.5 Hz, 1 H), 4.76-4.73 (m, 2 H), 4.68-4.60 (m, 3 H), 4.55-4.48 (m, 5 H), 4.45-4.41 (m, 2 H), 4.40 (d, J = 8.0 Hz, 1 H), 4.23 (dd, J = 4.0, 11.5 Hz, 1 H), 4.15-4.04 (m, 5 H), 3.98-3.88 (m, 8 H), 3.86-3.78 (m, 5 H), 3.73-3.64 (m, 5 H), 3.62 (dd, J = 3.5, 10.5 Hz, 1 H), 3.55-3.51 (m, 2 H), 3.14-3.10 (m, 2 H), 2.10 (s, 3 H), 2.08 (s, 3 H), 2.05 (s, 3 H), 2.02 (s, 3 H), 1.54 (s, 3 H), 1.41 (s, 9 H). ^{13}C-NMR (125 MHz,

$CDCl_3$): δ.172.4, 170.6, 170.5, 169.8, 169.1, 168.3, 166.5, 141.2, 137.3, 135.6, 133.6, 133.5, 133.0, 130.0, 129.8 (3 C), 129.6 (2 C), 129.5, 129.4, 128.8 (2 C), 128.7, 128.6 (2 C), 128.5 (3 C), 128.4, 128.3 (2 C), 128.1, 128.0, 127.9, 127.7, 127.6, 127.1, 125.1, 120.0, 101.2, 100.9, 100.7, 99.2, 99.1, 75.0, 74.5, 72.6, 72.2, 71.7, 71.6, 71.3, 68.6, 47.0, 46.1, 42.1, 28.0, 20.8, 20.7, 20.6, 20.5, 20.0. HRMS: $C_{112}H_{119}Cl_4N_3O_{45}S_2$ $[M + 2\ NH_4]^{2+}$ calcd: 1232.7994, obsd: 1232.7911.

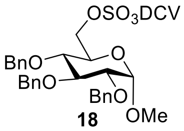

18

Methyl 2,3,4-tri-*O*-benzyl-6-*O*-dichlorovinylsulfo-α-D-glucoside (18)

Following the general procedure for DCV protection, compound **18** was obtained from primary alcohol **21** in 81% yield. $[\alpha]_D^{20}$ = +33.5 (*c* 1.0, CH_2Cl_2); ^{1}H NMR (500 MHz, $CDCl_3$): δ. 7.37-7.27 (m,15 H), 7.01 (s, 1 H), 5.04 (d, J = 11.0 Hz, 1 H), 4.94 (d, J = 11.0 Hz, 1 H), 4.85-4.80 (m, 2 H), 4.68 (d, J = 12.0 Hz, 1 H), 4.61-4.58 (m, 2 H), 4.50-4.44 (m, 2 H), 4.04 (t, J = 9.0 Hz, 1 H), 3.88-3.86 (m, 1 H), 3.55-3.53 (m, 1 H), 3.47 (t, J = 9.0 Hz, 1 H), 3.39 (s, 3 H). ^{13}C-NMR (125 MHz, $CDCl_3$): δ.138.4, 137.9, 137.5, 133.4, 128.6 (2 C), 128.5, 128.1 (2 C), 127.9, 127.8, 117.2, 98.2, 81.8, 79.7, 76.5, 75.8, 75.1, 73.5 (2 C), 68.1, 55.6, . HRMS: $C_{30}H_{32}Cl_2O_9S$ $[M + Na]^+$ calcd: 661.1036, obsd: 661.1033.

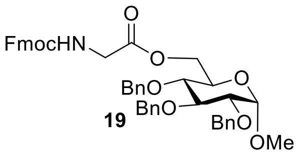

19

Methyl 2,3,4-tri-*O*-benzyl-6-*O*-(N-Fluorenylmethyloxycarbonyl-glycinyl)-α-D-glucoside (19)

Following the general procedure for testing DCV sulfate side reactions, compound **19** was obtained from Fmoc-glycine and compound **18** in 67% yield. $[\alpha]_D^{20}$ = +31.2 (*c* 1.0, CH_2Cl_2); ^{1}H NMR (500 MHz, $CDCl_3$): δ.7.78 (d, J = 7.5 Hz, 2 H), 7.60 (d, J = 7.0 Hz, 2 H), 7.42-7.26 (m, 19 H), 5.21 (bs, 1 H), 5.02 (d, J = 11.0 Hz, 1 H), 4.90 (d, J = 11.0 Hz, 1 H), 4.84 (d, J = 11.5 Hz, 1 H), 4.80 (d, J = 12.5 Hz, 1 H), 4.66 (d, J = 12.0 Hz, 1 H), 4.59-4.57 (m, 2 H), 4.40-4.29 (m, 4 H), 4.24-4.21 (m, 1 H), 4.03-3.89 (m, 3 H), 3.83-3.81 (m, 1 H), 3.53 (dd, J = 3.0, 9.5 Hz, 1 H), 3.46 (t, J = 9.5 Hz, 1 H), 3.36 (s, 3 H). ^{13}C-NMR (125 MHz, $CDCl_3$): δ.169.8, 156.1, 143.8, 141.3, 138.5, 138.0, 137.8, 128.5,

128.4, 128.2, 128.1, 128.0 (2 C), 127.7 (2 C), 127.1, 125.1, 120.0, 98.0, 73.4, 68.4, 67.2, 63.9, 55.3, 47.1, 42.6. HRMS: $C_{45}H_{45}NO_9$ $[M + NH_4]^+$ calcd: 761.3433, obsd: 761.3426.

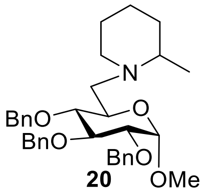

20

Methyl 2,3,4-tri-*O*-benzyl-6-(2-methyl-piperidinyl)-6-deoxy-α-D-glucoside (20)

Following the general procedure for testing DCV sulfate side reactions, compound **20** was obtained from 2-methyl piperidine and compound **18** in 70% yield. ^{1}H NMR (500 MHz, $CDCl_3$): δ.7.34-7.25 (m, 15 H), 5.00 (bs, 1 H), 4.90 (bs, 1 H), 4.81-4.78 (m, 2 H), 4.67-4.54 (m, 3 H), 4.01-3.97 (m, 2 H), 3.49-3.45 (m, 4 H), 3.19-2.51 (m, 6 H), 1.68 (bs, 4 H), 1.30-1.13 (m, 5 H). ^{13}C-NMR (125 MHz, $CDCl_3$): δ.138.5, 138.0, 128.5 (2 C), 128.4, 128.2, 128.1, 128.0 (3 C), 127.7, 98.3, 81.8, 79.7, 75.8, 75.3, 75.1, 73.5, 73.4, 56.4, 54.3, 31.9, 23.3, 22.7, 18.2. HRMS: $C_{34}H_{43}NO_5$ $[M + H]^+$ calcd: 546.3214, obsd: 546.3220.

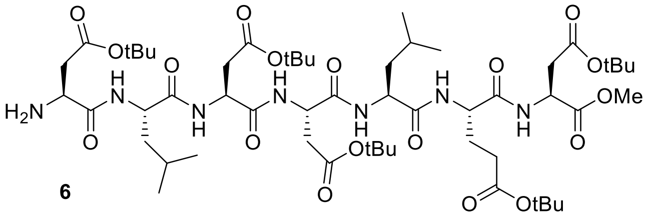

6

Peptide **6** was obtained following Fmoc based solid-phase peptide synthesis (75% yield based on resin loading). The resin was washed by hexafluoroisopropanol (HFIP)/DCM (1/4) for three times (3*5mins). The resulting solution was concentrated followed by the methylation and Fmoc removal procedures. $[\alpha]_D^{20}$ = -28.4 (*c* 0.8, MeOH); ^{1}H NMR (500 MHz, $CDCl_3$): δ. 4.76 (t, J = 6.5 Hz, 1 H), 4.64-4.59 (m, 2 H), 4.40-4.34 (m, 2 H), 4.28-4.25 (m, 1 H), 3.72 (s, 3 H), 3.68 (t, J = 6.0 Hz, 1 H), 2.89-2.59 (m, 8 H), 2.36-2.32 (m, 2 H), 2.16-2.09 (m, 1 H), 1.96-1.89 (m, 1 H), 1.78-1.71 (m, 3 H), 1.68-1.62 (m, 3 H), 1.47 (s, 9 H), 1.45-1.44 (m, 36 H). ^{13}C-NMR (125 MHz, $CDCl_3$): δ.173.9, 173.1, 172.3, 171.8, 171.7, 171.6, 171.3, 171.0, 170.0, 169.6, 81.3, 81.2, 81.1, 80.3, 52.9, 52.4, 52.3, 51.6, 51.3, 50.9, 50.8, 49.0, 40.2, 39.9, 39.8, 36.8, 36.2, 36.0, 31.1, 27.0 (5 C), 26.8, 24.4, 24.3, 22.3, 22.0, 20.7, 20.3. HRMS: $C_{54}H_{93}N_7O_{18}$ $[M + Na]^+$ calcd: 1150.6469, obsd: 1150.6448.

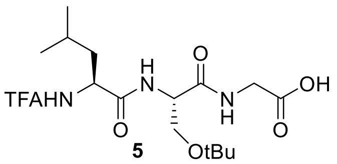

5

Following the general procedures of peptide synthesis in solution phase, Fmoc removal and Bn hydrogenolysis, compound **5** was obtained from sequential reactions of NH2-Gly-OBn, Fmoc-Ser-OH, and TFA-Leu-OH. $[\alpha]_D^{20}$ = +0.2 (*c* 0.1, CH_2Cl_2); ^1H NMR (500 MHz, $CDCl_3$): *δ*. 4.61-4.58 (m, 1 H), 4.52-4.50 (m, 1 H), 3.99-3.90 (m, 2 H), 3.69-3.61 (m, 2 H), 1.76-1.65 (m, 3 H), 1.20 (s, 9 H), 0.98 (d, J = 6.0 Hz, 3 H), 0.95 (d, J = 6.0 Hz, 3 H). ^{13}C-NMR (125 MHz, $CDCl_3$): *δ*.171.8, 171.2, 171.0, 158.0 (q, J = 37.1 Hz, TFA), 119.4 (q, J = 285.3, CF_3), 73.4, 61.3, 53.7, 52.1, 40.5, 39.7, 26.2, 24.5, 22.0, 20.3. HRMS: $C_{17}H_{28}F_3N_3O_6$ $[M + Na]^+$ calcd: 450.1822, obsd: 450.1827.

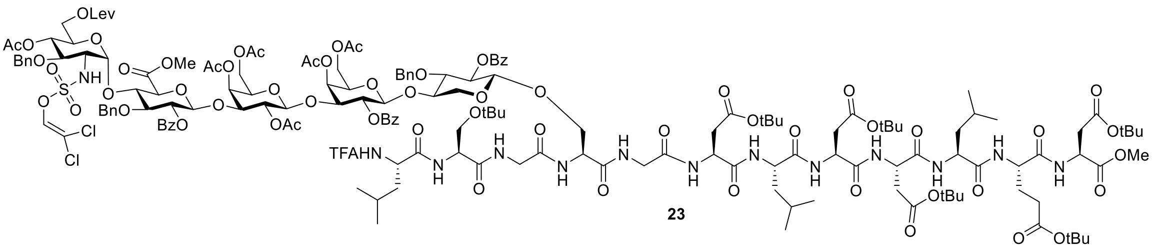

23

Compound **17** (10 mg) was dissolved in TFA/H_2O/TIPS (95/2.5/2.5, 2 ml), and the solution was stirred at rt for 1 hour. The reaction was concentrated followed by purification with Sephadex LH 20 column (MeOH/DCM = 1/1). The resulting carboxylic acid was treated with the general procedures for peptide synthesis in solution phase with compound **6**, Fmoc removal and peptide synthesis with compound **5** to afford glycopeptide **23** in 86% for four steps. $[\alpha]_D^{20}$ = -0.2 (*c* 0.1, CH_2Cl_2); ^1H NMR (500 MHz, $CDCl_3$): *δ*. 8.00-7.93 (m, 6 H), 7.63-7.56 (m, 4 H), 7.51-7.43 (m, 6 H), 7.34-7.31 (m, 2 H), 7.24-7.14 (m, 11 H), 6.90 (s, 1 H), 6.18 (d, J = 9.0 Hz, 1 H), 5.43-5.36 (m, 4 H), 5.28 (d, J = 2.0 Hz, 1 H), 5.23 (t, J = 5.5 Hz, 1 H), 5.13 (s, 1 H), 5.10-5.07 (m, 1 H), 5.04 (d, J = 9.0 Hz, 1 H), 5.00 (d, J = 9.5 Hz, 1 H), 4.82 (d, J = 11.5 Hz, 1 H), 4.76-4.72 (m, 2 H), 4.67-4.64 (m, 2 H), 4.56-4.52 (m, 2 H), 4.45-4.35 (m, 4 H), 4.14-4.05 (m, 5 H), 3.97-3.93 (m, 5 H), 3.87-3.81 (m, 6 H), 3.73-3.62 (m, 7 H), 3.53 (t, J = 9.0 Hz, 1 H), 3.26 (bs, 1 H), 2.82-2.57 (m, 12 H), 2.35-2.29 (m, 4 H), 2.19-2.00 (m, 16 H), 1.89 (s, 3 H), 1.44-1.33 (m, 47 H), 0.93-0.86 (m, 18 H). ^{13}C-NMR (125 MHz, $CDCl_3$): *δ*.206.6, 172.5, 171.1, 170.6 (2 C), 170.3, 169.8, 169.6 (2 C), 169.2, 168.7, 167.7, 165.1, 164.4, 138.0, 137.5, 135.7, 133.8, 129.9, 129.8, 129.5, 128.9, 128.7 (2 C), 128.6, 128.5, 128.4, 128.1 (2 C), 128.0, 127.9, 127.6, 127.3, 100.3, 100.9, 100.8, 99.6, 99.2, 81.2, 79.4, 77.7, 76.1, 74.9, 74.8, 74.0,

73.5, 72.9, 72.6, 71.6, 71.5, 71.3, 70.3, 69.5, 69.3 (2 C), 68.3, 61.9, 61.5, 58.5, 53.8, 52.9, 52.7, 37.9, 31.9, 29.8, 29.4, 28.1, 28.0 (3 C), 27.9, 27.8, 27.3, 24.7, 24.4, 23.2, 23.0, 22.9, 22.7, 22.3, 21.9, 21.7, 21.5, 20.8, 20.7 (2 C), 20.6, 19.8, 19.2. HRMS: $C_{167}H_{224}Cl_2F_3N_{13}O_{64}S$ [M + H + 2 NH_4]$^{3+}$ calcd: 1210.4824, obsd: 1210.4790.

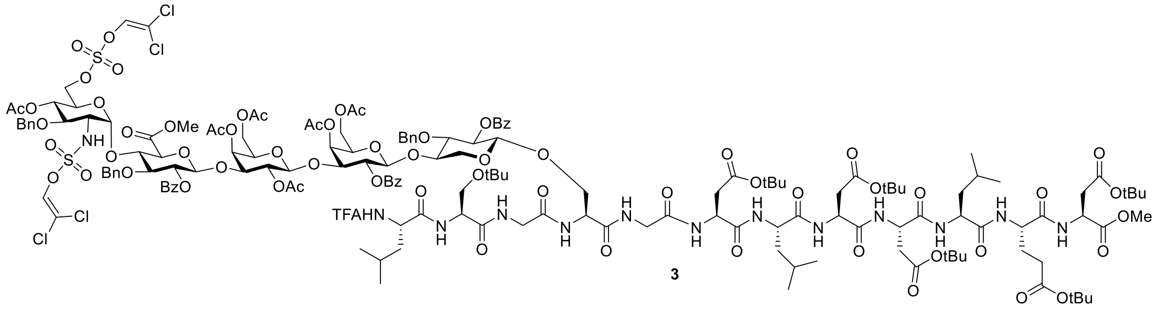

3

Following the general procedures for Lev removal and DCV protection, compound **3** was obtained from **23** in 80% yield. $[\alpha]_D^{20}$ = -0.2 (c 0.2, CH_2Cl_2); ^{1}H NMR (500 MHz, $CDCl_3$): δ. 8.05-7.94 (m, 6 H), 7.61-7.33 (m, 14 H), 7.25-7.09 (m, 8 H), 6.89 (s, 1 H), 6.18 (bs, 1 H), 5.47-5.34 (m, 4 H), 5.29-5.20 (m, 2 H), 5.16-5.01 (m, 4 H), 4.83-4.68 (m, 6 H), 4.56-3.37 (m, 44 H), 3.17-3.10 (m, 2 H), 2.96-2.65 (m, 10 H), 2.09-1.90 (m, 16 H), 0.91-0.86 (m, 18 H). ^{13}C-NMR (125 MHz, $CDCl_3$): δ.172.5, 172.3, 170.7, 170.1, 169.9, 169.8, 169.3, 168.5, 168.3, 167.9, 165.1, 164.9, 164.4, 138.1, 137.8, 137.2, 136.1, 133.6, 129.9, 129.7, 128.7 (2 C), 128.6, 128.4 (2 C), 128.3, 128.2, 128.1 (2 C), 127.8, 127.7 (2 C), 127.5, 101.3, 101.2, 100.3, 99.8, 99.7, 81.6, 81.4, 81.3, 74.9, 74.3, 74.0, 73.5, 72.1, 71.3, 68.9, 62.1, 61.8, 55.2, 53.6, 53.0, 52.8, 40.0, 38.9, 36.0, 31.5, 28.1, 28.0, 27.9, 27.8, 27.3, 27.2, 24.7, 24.4, 22.8, 22.7, 22.4, 22.2, 22.1, 21.8, 21.7, 20.9, 20,7 (2 C), 20.6, 19.8. HRMS: $C_{164}H_{218}Cl_4F_3N_{13}O_{65}S_2$ [M + 2 NH_4]$^{2+}$ calcd: 1853.1488, obsd: 1853.1398.

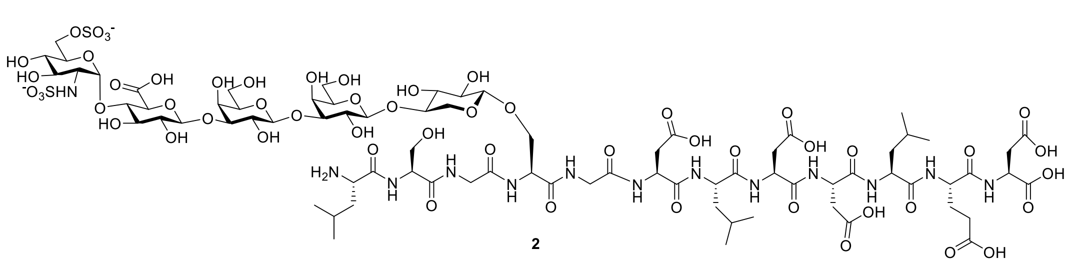

2

Compound **3** (5 mg) was dissolved in TFA/H_2O/TIPS (95/2.5/2.5, 1 ml), and the solution was stirred at rt for 1.5 hour. The reaction was concentrated followed by purification with Sephadex LH 20 column (MeOH/DCM = 1/1). The resulting compound was treated with the general procedures for

DCV deprotection to produce two free sulfates with partially Bn deprotected compound. The mixture was dissolved in THF- H_2O (2:1, 0.4 mL), to which 0.25 M LiOH was added to maintain pH around 9.0 under 0°C. When MS analysis showed the complete disappearance of the starting material (1 hour at 0°C), the mixture was neutralized by 1 M HOAc solution to adjust pH to around 7. The solution was loaded onto a LH-20 column (MeOH) directly to remove the Li salt. Glycopeptide containing fractions were combined and concentrated. The collected compound was dissolved in MeOH (0.4 mL), followed by addition of hydrazine hydrate (0.1 mL). The resulting mixture was stirred under room temperature overnight and it was neutralized by acetone under 0°C for 30 min. The solution was concentrated and loaded onto a Sephadex G-15 column to afford glycopeptide **2** in 41% for four steps. ^{1}H NMR (500 MHz, D_2O): δ. 5.53 (d, J = 4.0 Hz, 1 H), 4.57-4.53 (m, 3 H), 4.49-4.40 (m, 8 H), 4.32-4.15 (m, 2 H), 4.26-4.19 (m, 7 H), 4.07-4.03 (m, 4 H), 3.93 (bs, 2 H), 3.87-3.84 (m, 3 H), 3.80-3.78 (m, 2 H), 3.75-3.53 (m, 19 H), 3.48-3.46 (m, 3 H), 3.36-3.34 (m, 2 H), 3.28-3.27 (m, 1 H), 3.23 (bs, 1 H), 3.16-3.13 (m, 2 H), 2.64-2.42 (m, 18 H), 1.58-1.51 (10 H), 0.85-0.75 (m, 18 H). HRMS: $C_{78}H_{123}N_{13}O_{55}S_2^{2-}$ $[M]^{2-}$ calcd: 1092.8340, obsd: 1092.8276, $[M - H]^{3-}$ calcd: 728.2202, obsd: 728.2206.

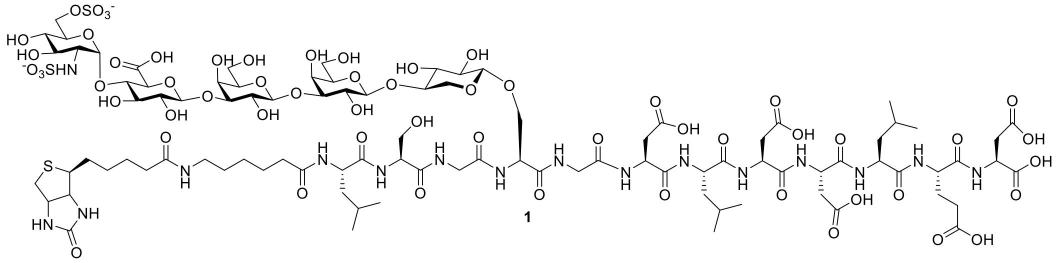

1

Following the general procedure for biotinylation, glycopeptide **1** was obtained from **2** in 76% yield. The overall yield from known disaccharide **7** is 3.4%. ^{1}H NMR (500 MHz, D_2O): δ. 5.49 (bs, 1 H), 4.53-4.50 (m, 3 H), 4.46-4.44 (m, 4 H), 4.37-4.36 (m, 2 H), 4.27-4.18 (m, 8 H), 4.04-4.01 (m, 6 H), 3.88-3.43 (m, 37 H), 3.18-3.17 (m, 3 H), 3.03-2.99 (m, 4 H), 2.93-2.88 (m, 6 H), 2.85 (d, J = 5.0 Hz, 1 H), 2.83 (d, J = 5.0 Hz, 1 H), 2.73-2.42 (m, 21 H), 1.49-1.35 (m, 22 H), 0.78-0.72 (m, 18 H). MS: $C_{94}H_{148}N_{16}O_{58}S_3^{2-}$ $[M]^{2-}$ calcd: 1262.9165, obsd: 1262.9006

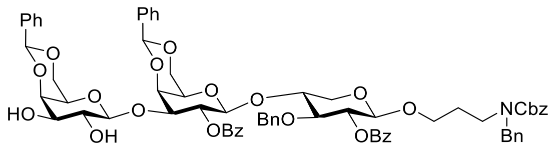

25

A solution of donor **9e** (200 mg, 0.16 mmol), linker acceptor 3-*N*-Cbz-*N*-Bn amino-1-propanol (45 mg, 0.15 mmol) and freshly activated molecular sieve MS 4 Å (100 mg) in DCM (1.5 mL) was stirred at room temperature for 30 minutes, and cooled to −78 °C, which was followed by addition of AgOTf (123 mg, 0.48 mmol) dissolved in acetonitrile (0.1 mL) without touching the wall of the flask. After 5 minutes, orange colored *p*-TolSCl (23 μL, 0.16 mmol) was added to the solution through a microsyringe. The reaction mixture was warmed to -30 °C under stirring in 1 h. Then the mixture was quenched by DIPEA, diluted with DCM and filtered over Celite. The filtrate was concentrated *in vacuo*, followed by silica gel flash chromatography to afford trisaccharide serine. After that, following the general procedure for Lev removal, trisaccharide **25** was obtained in 84% for two steps. $[\alpha]_D^{20}$ = -1.6 (*c* 0.3, CH_2Cl_2); ^{1}H NMR (500 MHz, $CDCl_3$): *δ*. 8.06 (d, J = 8.0 Hz, 2 H), 7.96 (d, J = 7.0 Hz, 2 H), 7.60-7.53 (m, 4 H), 7.49-7.46 (m, 4 H), 7.41-7.28 (m, 11 H), 7.23-7.20 (m, 5 H), 7.14-7.03 (m, 5 H), 5.67 (t, J = 7.0 Hz, 1 H), 5.60 (s, 1 H), 5.50 (s, 1 H), 5.15-5.06 (m, 3 H), 5.01 (d, J = 11.0 Hz, 1 H), 4.78 (d, J = 8.0 Hz, 1 H), 4.73 (d, J = 11.0 Hz, 1 H), 4.44-4.22 (m, 7 H), 4.08-3.95 (m, 5 H), 3.87 (bs, 1 H), 3.74-3.66 (m, 3 H), 3.44 (s, 1 H), 3.39-3.43 (m, 2 H), 3.18-3.07 (m, 3 H), 2.61 (s, 1 H), 2.47 (d, J = 7.5 Hz, 1 H), 1.71 (bs, 1 H), 1.62 (bs, 1 H). ^{13}C-NMR (125 MHz, $CDCl_3$): *δ*. 165.8, 165.1, 156.6, 156.0, 138.2, 137.9, 137.5, 133.4, 133.1, 129.8, 129.7 (2 C), 129.6, 129.3, 129.0, 128.7, 128.4 (2 C), 128.3, 128.2, 128.1, 127.8, 127.4, 127.3, 126.6, 126.3, 104.2, 101.5, 101.3, 101.2 (2 C), 79.7, 79.6, 78.4, 77.9, 76.0, 75.0, 74.5, 72.7, 72.0, 71.6, 71.0, 69.1, 68.7, 67.1, 66.9, 66.8, 63.3, 51.0, 50.8, 44.7, 43.5, 28.4, 28.0. HRMS: $C_{70}H_{71}NO_{19}$ $[M + NH_4]^+$ calcd: 1247.4959, obsd: 1247.4906.

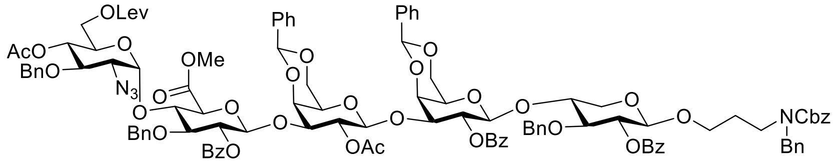

26

Following the procedure about the synthesis of **13** and general procedures for oxidation, methylation and Ac protection, compound **26** was obtained in 56% yield from **25** for five steps. $[\alpha]_D^{20}$ = +3.1 (*c*

0.3, CH_2Cl_2); ^{1}H NMR (500 MHz, $CDCl_3$): δ. 8.09 (d, J = 6.5 Hz, 2 H), 7.95-7.94 (m, 4 H), 7.64-7.61 (m, 1 H), 7.59-7.48 (m, 8 H), 7.42-7.29 (m, 15 H), 7.23-7.17 (m, 9 H), 7.14-7.03 (m, 5 H), 5.61 (t, J = 8.5 Hz, 1 H), 5.52 (s, 1 H), 5.43 (d, J = 3.0 Hz, 1 H), 5.39 (s, 1 H), 5.30-5.25 (m, 2 H), 5.14-5.06 (m, 3 H), 4.99-4.96 (m, 3 H), 4.82 (d, J = 11.0 Hz, 1 H), 4.69-4.61 (m, 6 H), 4.40-4.25 (m, 6 H), 4.19-4.07 (m, 6 H), 3.99-3.92 (m, 5 H), 3.87 (t, J = 10.0 Hz, 1 H), 3.76-3.57 (m, 9 H), 3.37-3.25 (m, 5 H), 3.18-2.95 (m, 4 H), 2.78-2.54 (m, 5 H), 2.17 (s, 3 H), 1.95 (s, 3 H), 1.69-1.57 (m, 5 H). ^{13}C-NMR (125 MHz, $CDCl_3$): δ. 206.5, 172.5, 169.4, 169.0, 168.9, 165.1, 164.7 (2 C), 138.3, 138.0, 137.5, 137.3, 137.1, 133.5, 133.3, 133.0, 130.0, 129.9, 129.8, 129.7, 129.6, 129.3, 128.8, 128.7, 128.5 (2 C), 128.4 (2 C), 128.2, 128.1, 128.0 (3 C), 127.9, 127.8, 127.3, 126.4, 126.1, 101.8, 101.3, 101.1, 100.8, 100.6, 100.5, 99.7, 97.3, 82.5, 79.8, 78.5, 75.6, 75.5, 75.2, 74.7, 74.6, 74.5, 73.8 (2 C), 72.9, 72.8, 71.5, 69.6, 69.1, 68.8, 68.7, 68.6, 67.1, 66.9, 66.7, 63.3, 62.7, 61.7, 52.6, 51.0, 50.8, 44.7, 43.5, 37.9, 29.8, 28.4, 27.9, 20.8, 20.2. HRMS: $C_{113}H_{116}N_4O_{34}$ [M + 2 NH_4]$^{2+}$ calcd: 1054.4073, obsd: 1054.4053.

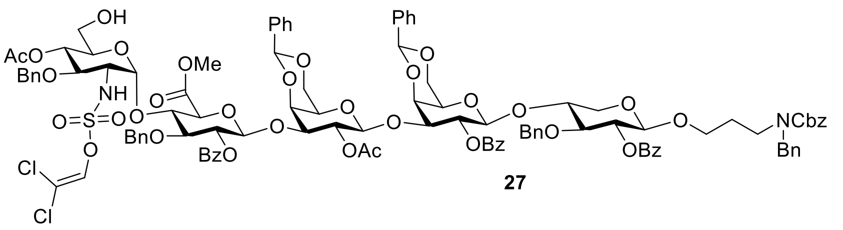

27

Compound **26** (21 mg, 0.01 mmol) was dissolved in THF/ AcOH (3:1, 1.2 mL), followed by addition of Zn (60 mg). The resulting mixture was stirred under room temperature for 1 hour. After filtration and concentration by vacuum, the mixture was loaded onto a silica gel column for purification. The resulting compound was treated with the general procedures for DCV protection and Lev removal to afford **27** in 58% yield. $[\alpha]_D^{20}$ = +0.2 (*c* 0.2, CH_2Cl_2); ^{1}H NMR (500 MHz, $CDCl_3$): δ. 8.08 (d, J = 8.0 Hz, 2 H), 7.95-7.93 (m, 4 H), 7.64 (t, J = 7.5 Hz, 1 H), 7.60-7.47 (m, 8 H), 7.43-7.28 (m, 15 H), 7.25-7.19 (m, 10 H), 7.14-7.03 (m, 5 H), 6.9 (s, 1 H), 5.95 (d, J = 9.0 Hz, 1 H), 5.58 (t, J = 9.0 Hz, 1 H), 5.50 (s, 1 H), 5.36-5.31 (m, 2 H), 5.22-5.17 (m, 2 H), 5.14-5.06 (m, 3 H), 4.98 (d, J = 11.0 Hz, 1 H), 4.89-4.85 (m, 2 H), 4.75 (d, J = 11.5 Hz, 1 H), 4.66-4.57 (m, 5 H), 4.51 (d, J = 12.0 Hz, 1 H), 4.39-4.28 (m, 4 H), 4.23-4.11 (m, 6 H), 3.97-3.91 (m, 5 H), 3.75-3.44 (m, 13 H), 3.33 (s, 1 H), 3.28 (s, 3 H), 3.18-3.11 (m, 2 H), 3.03-2.94 (m,1 H), 2.56 (bs, 1 H), 1.96 (s, 3 H), 1.69 (bs, 1 H), 1.60

(bs,1 H), 1.45 (s, 3 H). ^{13}C-NMR (125 MHz, $CDCl_3$): δ. 170.4, 168.9, 168.2, 165.1, 164.7, 164.6, 138.2, 138.0, 137.5, 136.0, 133.7 (2 C), 133.4, 133.0, 130.0, 129.9, 129.8, 129.7, 129.6, 129.0, 128.9, 128.7, 128.6 (2 C), 128.5, 128.4 (2 C), 128.2 (2 C), 128.0 (2 C), 127.9 (2 C), 127.6, 127.3, 126.4, 126.0, 115.4, 101.8, 101.1, 100.6 (3 C), 100.2, 99.0, 79.9, 79.8, 78.4, 78.2, 77.4, 76.9, 75.5, 75.0, 74.9, 74.6, 73.7, 73.4, 72.1, 71.5, 70.2, 69.3, 69.2, 68.7, 68.5, 67.1, 66.9, 66.7, 64.0, 63.3, 60.9, 58.3, 53.0, 51.0, 28.3, 27.9, 20.7, 20.1. HRMS: $C_{110}H_{112}Cl_2N_2O_{35}S$ $[M + 2\ NH_4]^{2+}$ calcd: 1079.3410, obsd: 1079.3387.

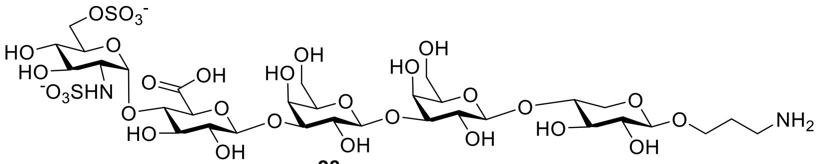

28

Following the general procedures for *O*-sulfation, DCV deprotection, saponification and global debenzylation, compound **28** was obtained from **27** in 61% yield. ^{1}H NMR (500 MHz, D_2O): δ. 5.50 (d, J = 3.5 Hz, 1 H), 4.53-4.50 (m, 2 H), 4.38 (d, J = 8.0 Hz, 1 H), 4.30 (d, J = 8.0 Hz, 1 H), 4.20 (dd, J = 3.5, 11.0 Hz, 1 H), 4.03-3.94 (m, 4 H), 3.86-3.82 (m, 1 H), 3.72-3.50 (m, 14 H), 3.46-3.40 (m, 3 H), 3.32-3.23 (m, 2 H), 3.17-3.10 (m, 2 H), 2.99 (t, J = 7.0 Hz, 2 H), 1.87-1.81 (m, 2 H). HRMS: $C_{32}H_{54}N_2O_{31}S_2^{2-}$ $[M]^{2-}$ calcd: 513.1082, obsd: 513.1091, $[M + H]^-$ calcd: 1027.2236, obsd: 1027.2178.

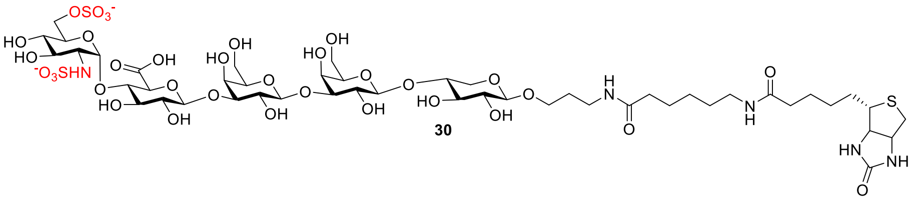

30

Following the general procedure for biotinylation, pentasaccharide **30** was obtained from **28** in 90% yield. The overall yield from known disaccharide **7** is 8.8%. ^{1}H NMR (500 MHz, D_2O): δ. 5.50 (d, J = 3.5 Hz, 1 H), 4.53-4.50 (m, 2 H), 4.46-4.44 (m, 1 H), 4.37 (d, J = 8.0 Hz, 1 H), 4.28-4.25 (m, 2 H), 4.20-4.17 (m,1 H), 4.03-3.99 (m, 3 H), 3.95 (dd, J = 5.0, 12.0 Hz, 1 H), 3.77-3.50 (m, 15 H), 3.45-3.42 (m, 2 H), 3.32 (t, J = 8.5 Hz, 1 H), 3.26 (t, J = 11.0 Hz, 1 H), 3.20-3.11 (m, 4 H), 3.04-3.00 (m, 2 H), 2.85 (dd, J = 4.5, 13.0 Hz, 1 H), 2.64 (d, J = 13.0 Hz, 1 H), 2.10 (t, J = 6.5 Hz, 3 H), 1.69-1.63 (m, 2 H), 1.60-1.33 (m, 9 H), 1.28-1.12 (m, 4 H). HRMS: $C_{48}H_{79}N_5O_{34}S_3^{2-}$ $[M + H]^-$ calcd:

1366.3852, obsd: 1366.3785, $[M]^{2-}$ calcd: 682.6890, obsd: 682.6887.

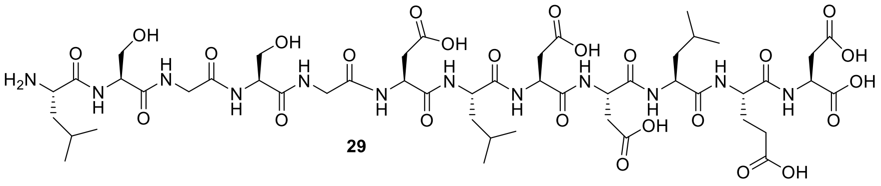

29

Following the general procedure for solid-phase peptide synthesis, peptide **29** was obtained in 52% yield based on resin loading. HRMS: $C_{49}H_{78}N_{12}O_{25}$ $[M + H]^+$ calcd: 1235.5274, obsd: 1235.5214. RP-HPLC retention time, t_R = 24.4 min.

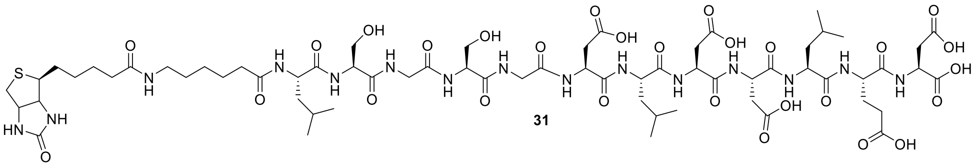

31

Following the general procedure for biotinylation, peptide **31** was obtained from 12-mer peptide **29** in 56% yield. The overall yield based on resin loading is 29%. HRMS: $C_{65}H_{103}N_{15}O_{28}S$ $[M - 2H]^{2-}$ calcd: 785.8336, obsd: 785.8295. RP-HPLC retention time, t_R = 29.8 min.

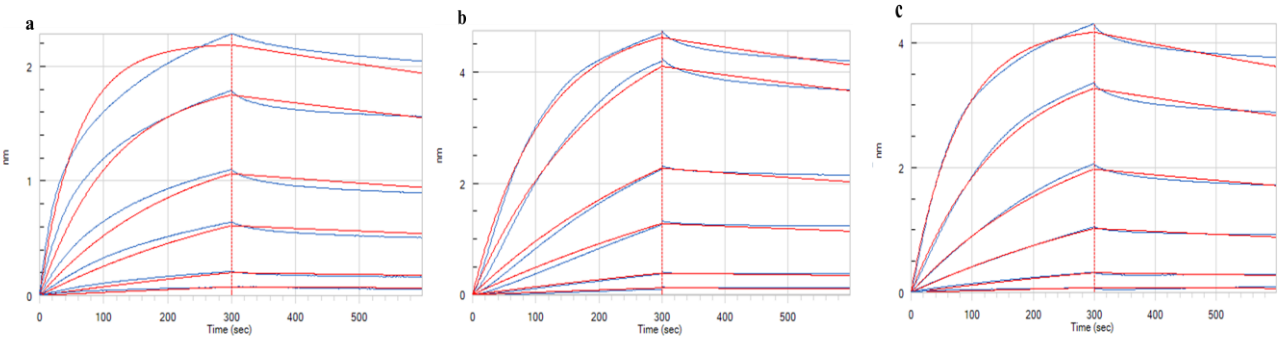

Figure S1: Binding of synthetic compounds **1**, **31** and **30** to protein FGF2 as measured by BLI (**a-c** respectively). The biotinylated compounds (2 μM) were immobilized on streptavidin coated biosensors, and FGF2 was captured on biosensors with six concentrations at 291 nM, 145 nM, 77 nM, 39 nM, 20 nM, 10 nM. Fitting curves were shown in red lines.

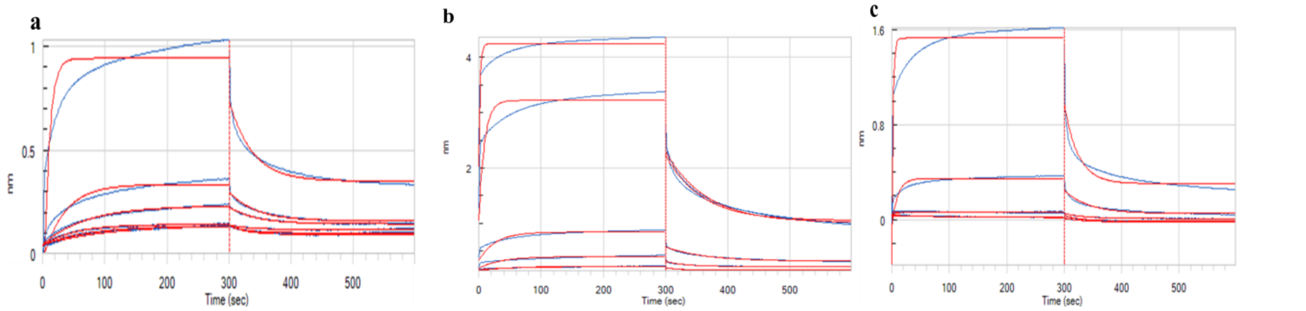

Figure S2: Binding of synthetic compounds **1**, **31** and **30** to protein IL8 as measured by BLI (**a-c** respectively). The biotinylated compounds (60 μM) were immobilized on streptavidin coated biosensors, and IL8 was captured on biosensors with six concentrations at 18.6 μM, 9.3 μM, 4.7 μM, 2.4 μM, 1.2 μM, 0.6 μM. Fitting curves were shown in red lines.

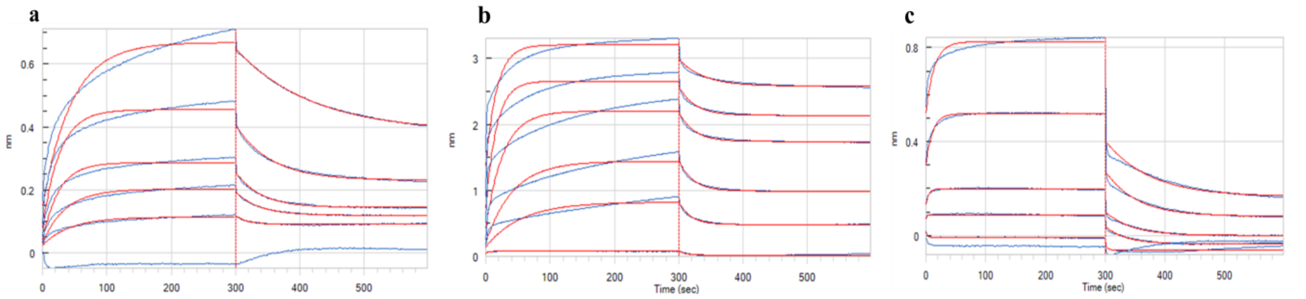

Figure S3: Binding of synthetic compounds **1**, **31** and **30** to protein CCL13 by BLI (**a-c** respectively). The biotinylated compounds (60 μM) were immobilized on streptavidin coated biosensors, and CCL13 was captured on biosensors with six concentrations at 23.2 μM, 11.6 μM, 5.8 μM, 2.9 μM, 1.5 μM, 0.7 μM. Fitting curves were shown in red lines.

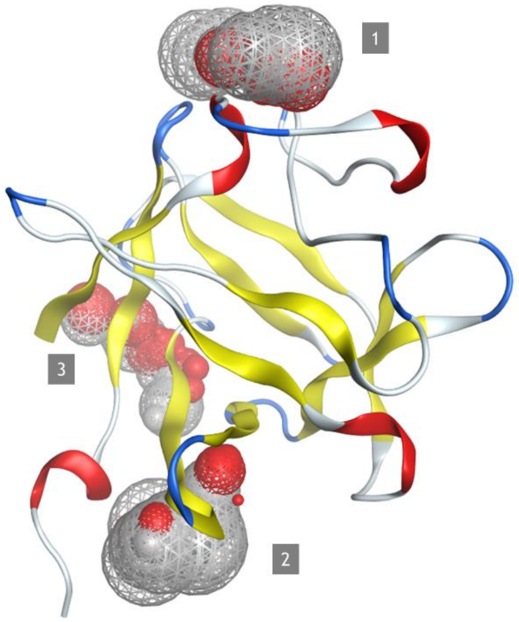

Figure S4: Potential Binding Sites on the FGF2 structure

Compound	Site	Average Binding Energies (kcal/mol)	
**Glycan 28**	1	-35.09	± 8.01
	2	-26.75	± 10.55
	3	-20.04	± 7.17
**Peptide 29**	1	-30.40	± 10.55
	2	-25.59	± 10.24
	3	-26.26	± 11.07
**Glycopeptide 2**	1	-60.04	± 13.65
	2	-37.40	± 13.88
	3	-41.48	± 12.03

Table S1: Average binding free energies and standard deviations calculated for glycan **28**, peptide **29** and glycopeptide **2** on 3 potential binding sites.

Binding Affinities			
**Compound**	**Average Binding Energies on Site 1 (kcal/mol)**		**Experimental K_D (nM)**
**2**	-60.04	± 13.65	5
**28**	-35.09	± 8.01	14.5
**29**	-30.40	± 10.55	17

Table S2: Binding affinities for compounds **2**, **28** and **29** on FGF-2 protein. Calculated results show average binding free energy results and experimental results show the K_D values from BLI measurements.

FGF-2 Site 1 Glycan Binding Comparison (kcal/mol)					
**Compound**	**Pose**	**ΔG Binding**	**STD**	**Average ΔG**	
**28**	1	-34.37	± 8.60	-35.09	± 8.01
	2	-35.25	± 6.59		
	3	-36.40	± 8.96		
	4	-31.72	± 6.45		
	5	-37.74	± 9.45		
**29**	1	-30.92	± 10.78	-30.40	± 10.55
	2	-25.55	± 8.97		
	3	-26.84	± 7.88		
	4	-35.85	± 10.16		
	5	-32.86	± 14.94		
**2**	1	-53.51	± 14.79	-60.04	± 13.65
	2	-77.67	± 16.44		
	3	-69.51	± 17.63		
	4	-47.82	± 12.29		
	5	-53.81	± 5.36		
	6	-51.88	± 18.17		
	7	-66.07	± 10.85		

Table S3: Binding free energy for compound **28**, **29** and **2** with FGF-2 calculated for various poses.

(a)

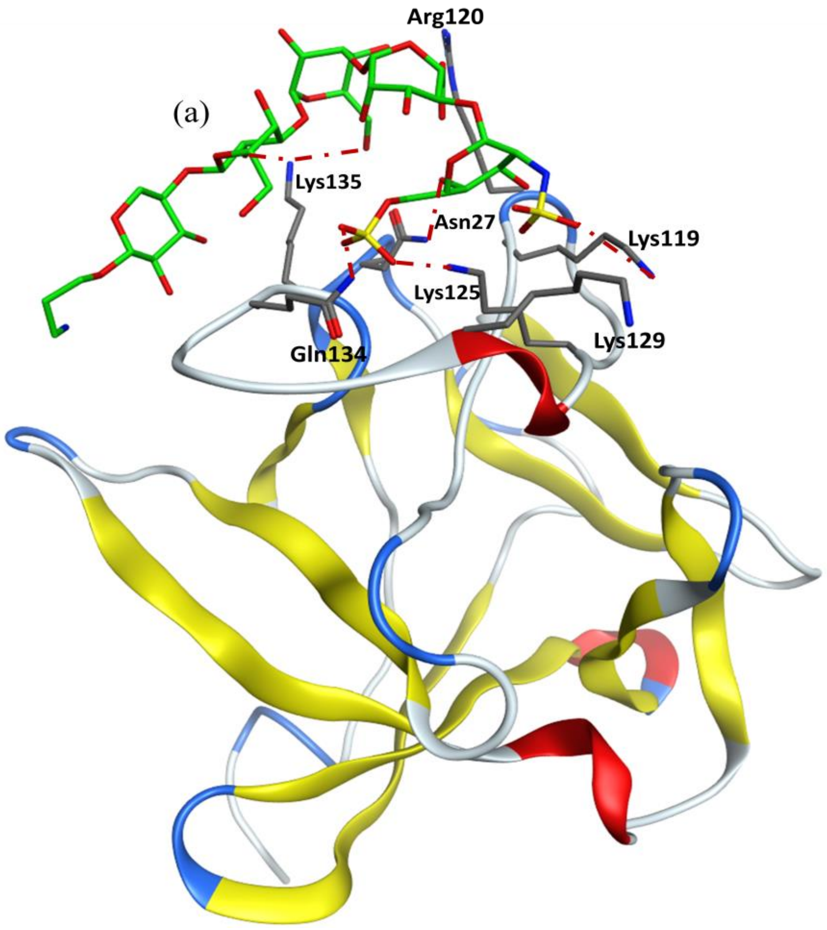

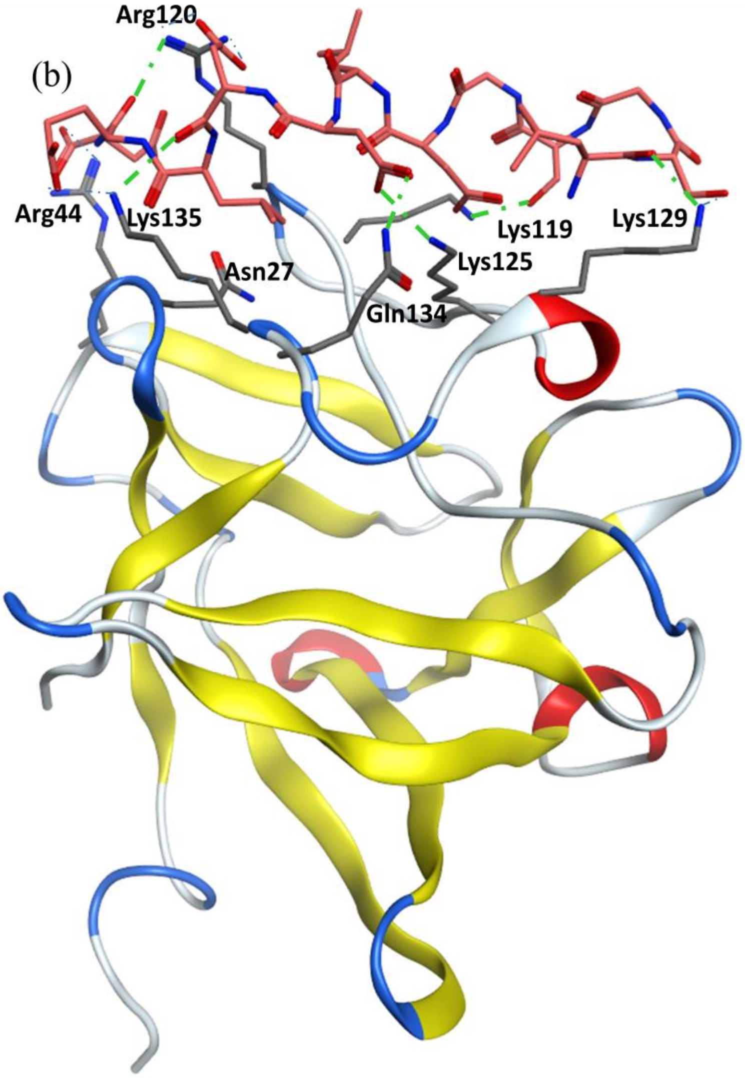

Figure S5: Representative binding pose of (a) glycan **28** and (b) peptide **29** in site 1 of FGF2.

Heparanase Binding (kcal/mol)					
**Glycan**	**Pose**	**ΔG Binding**	**STD**	**AVG ΔG**	**AVG STD**
**28**	1	-59.97	14.44	-57.36	12.19
	2	-53.89	10.00		
	3	-49.84	12.50		
	4	-72.58	10.83		
	5	-50.52	13.20		
**29**	1	-34.62	12.47	-43.14	14.45
	2	-61.90	20.10		
	3	-32.25	10.36		
	4	-35.28	12.51		
	5	-51.66	16.80		
**2**	1	-45.65	13.18	-50.55	15.67
	2	-55.65	15.23		
	3	-49.41	15.53		
	4	-46.77	15.42		
	5	-55.26	18.99		
**biotin**	1	-10.68	4.32	-15.04	4.46
	2	-13.82	4.13		
	3	-11.75	5.13		
	4	-16.61	4.09		
	5	-22.33	4.63		

Table S4: Binding free energy results for compounds **28**, **29, 2** and biotin with heparanase calculated for various binding poses.

Structure of biotin calculated:

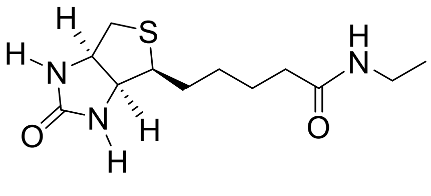

a

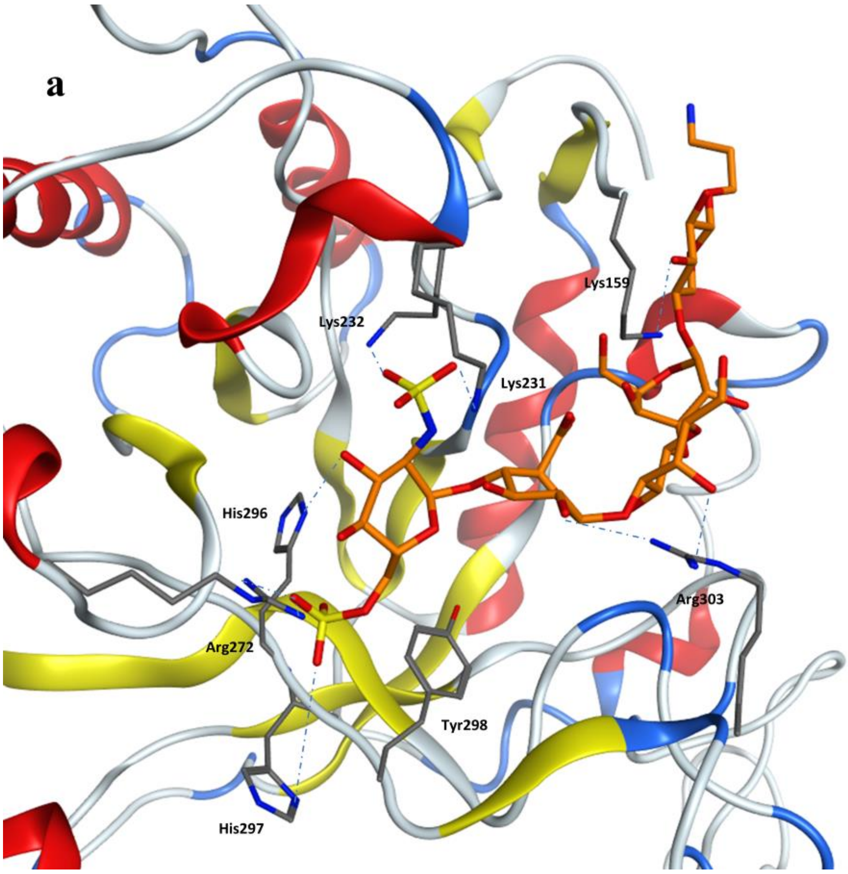
Lys159
Lys232
Lys231
His296
Arg303
Arg272
Tyr298
His297

b

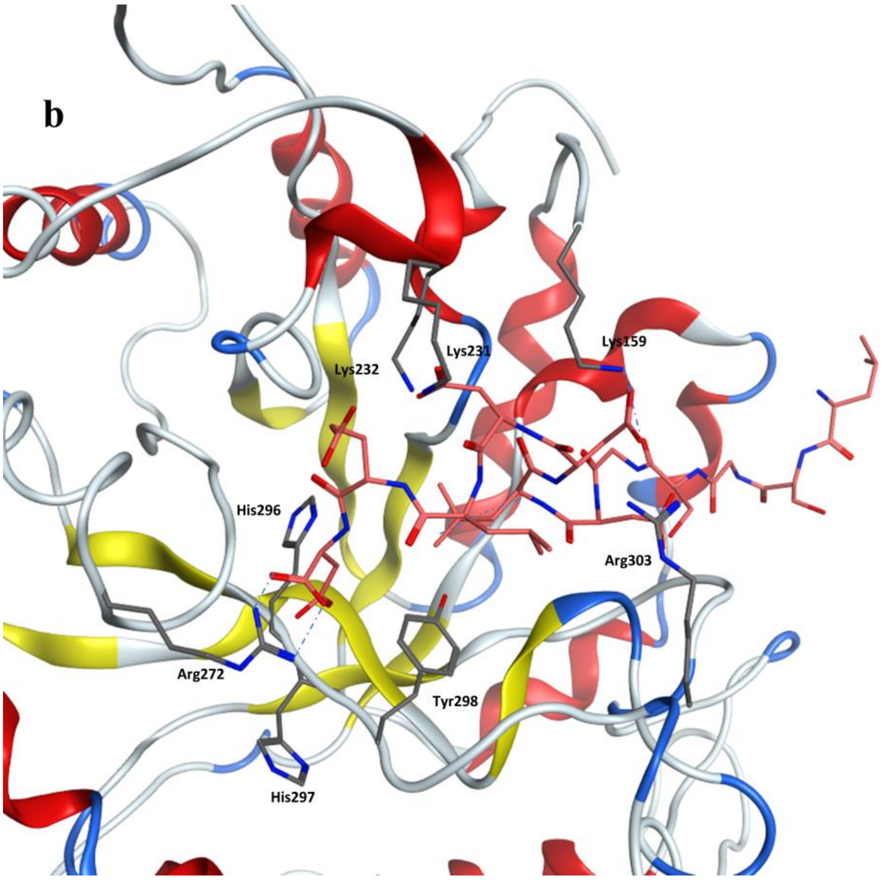
Lys159
Lys232
Lys231
His296
Arg303
Arg272
Tyr298
His297

c

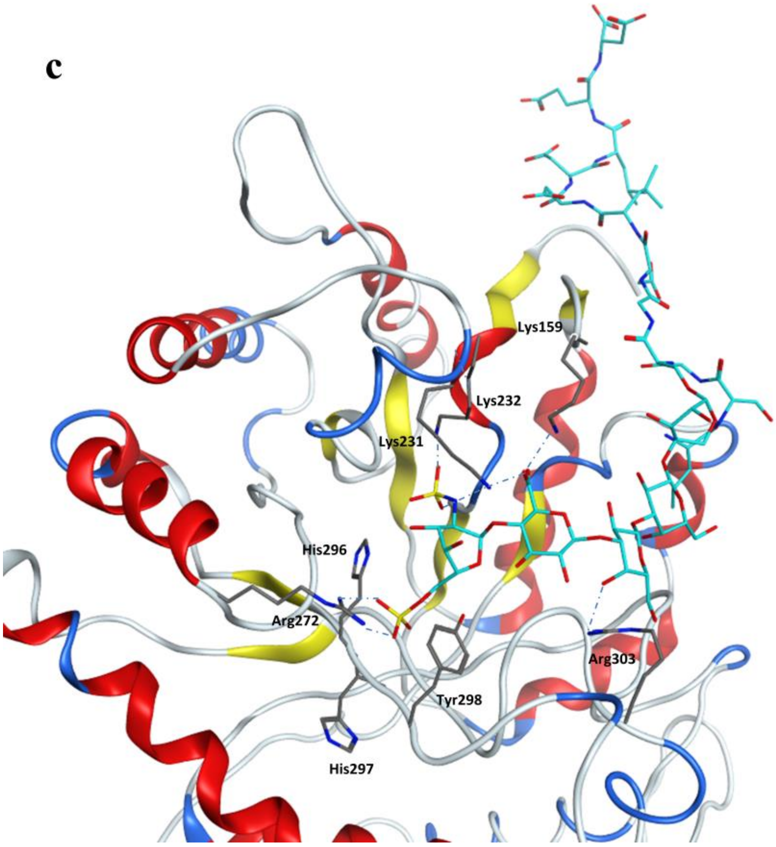

Figure S6. Representative binding poses of (a) glycan **28**, (b) peptide **29**, and (c) glycopeptide **2** in the heparin binding site of heparanase.

References:

1. Dewar, M. J. S.; Zoebisch, E. G.; Healy, E. F.; Stewart, J. J. P., Development and use of quantum mechanical molecular models. 76. AM1: a new general purpose quantum mechanical molecular model. *J. Am. Chem. Soc.* **1985,** *107*, 3902-3909.
2. Case, D. A.; Cerutti, D. S.; Cheatham, T. E.; Darden, T.; Duke, R. E.; Giese, T. J.; Gohlke, H.; Goetz, A. W.; Greene, D.; Homeyer, N.; Izadi, S.; Kovalenko, A. V.; Lee, T. S.; LeGrand, S.; Li, P.; Lin, C.; Liu, J.; Luchko, T.; Luo, R.; Mermelstein, D.; Merz, K. M.; Monard, G.; Nguyen, H.; Omelyan, I.; Onufriev, A.; Pan, F.; Qi, R.; Roe, D. R.; Roitberg, A.; Sagui, C.; Simmerling, C.; Botello-Smith, W. M.; Swails, J.; Walker, R. C.; Wang, J.; Wolf, R. M.; Wu, X.; Xiao, L.; York, D. M.; Kollman, P. A., *AMBER 2017, University of California, San Francisco.* **2017**.
3. Ryckaert, J. P.; Ciccotti, G.; Berendsen, H. J. C., Numerical integration of the Cartesian equations of motion of a system with constraints: molecular dynamics of n-alkanes. *J. Comput. Phys.* **1977,** *23*, 327-341.
4. Yang, B.; Yoshida, K.; Yin, Z.; Dai, H.; Kavunja, H.; El-Dakdouki, M. H.; Sungsuwan, S.; Dulaney, S. B.; Huang, X., Chemical Synthesis of a Heparan Sulfate Glycopeptide: Syndecan-1. *Angew. Chem. Int. Ed.* **2012,** *51*, 10185-10189.
5. Yoshida, K.; Yang, B.; Yang, W.; Zhang, Z.; Zhang, J.; Huang, X., Chemical Synthesis of Syndecan-3 Glycopeptides Bearing Two Heparan Sulfate Glycan Chains. *Angew. Chem. Int. Ed.* **2014,** *53*, 9051-9058.

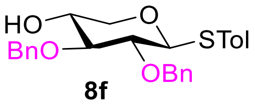

8f

^{1}H-NMR ($CDCl_3$, 500 MHz) of **8f**

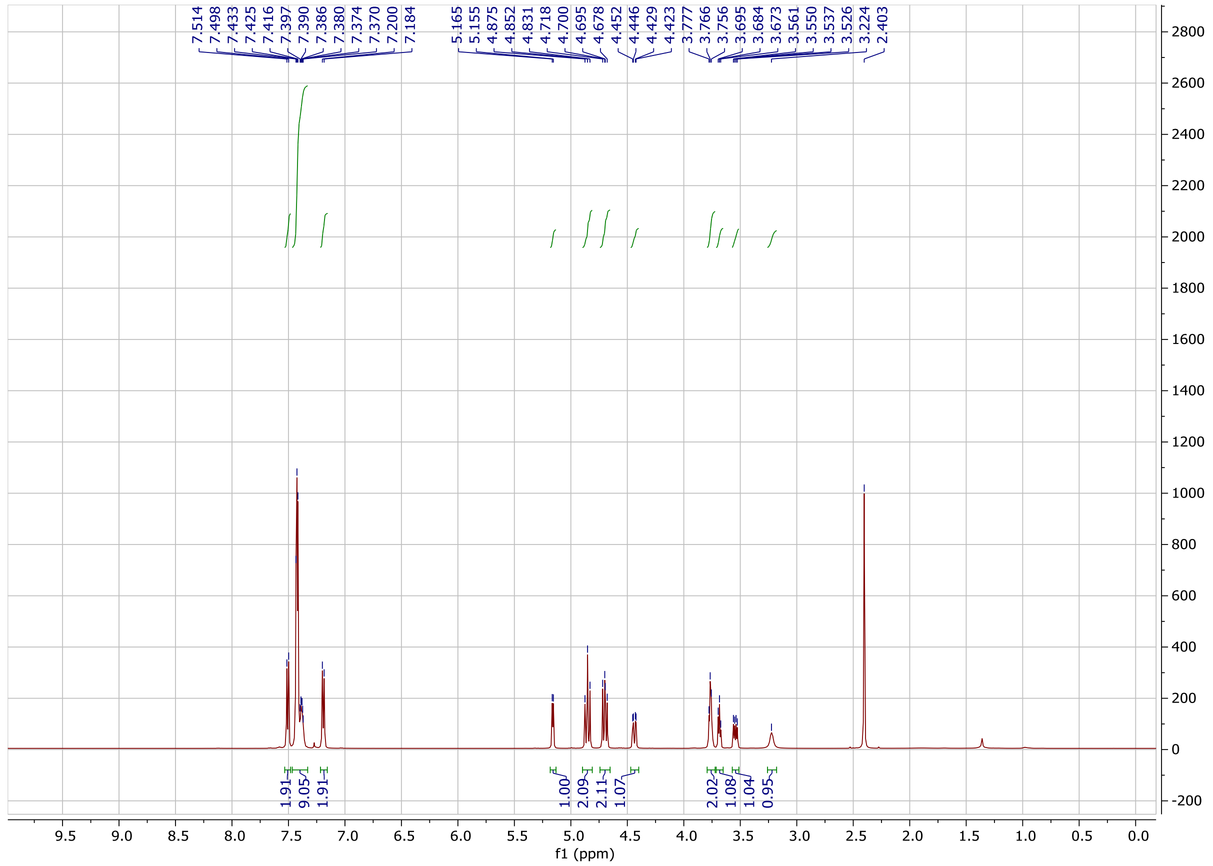

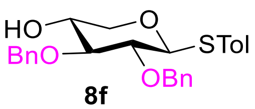

8f

^{13}C-NMR ($CDCl_3$, 125 MHz) of **8f**

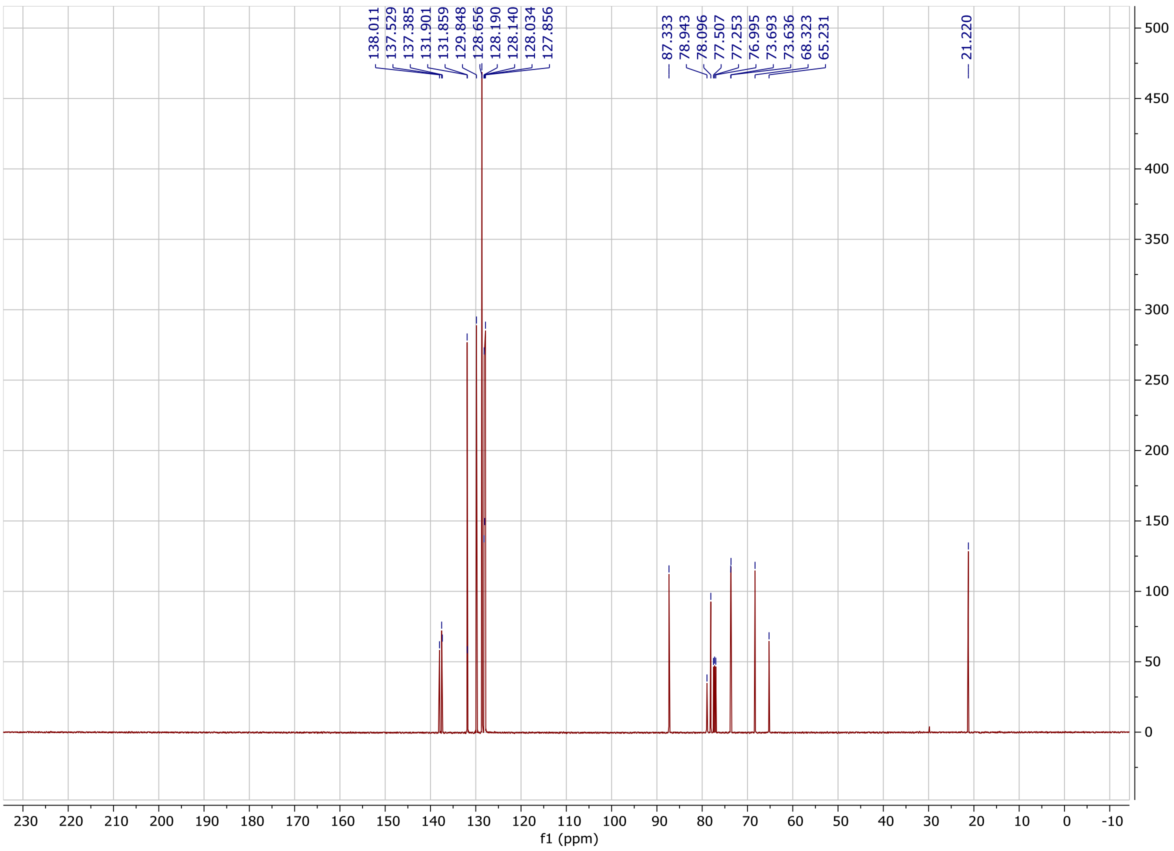

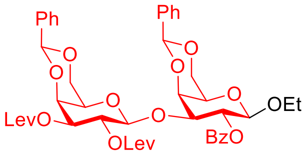

9b

^{1}H-NMR (CDCl$_3$, 500 MHz) of **9b**

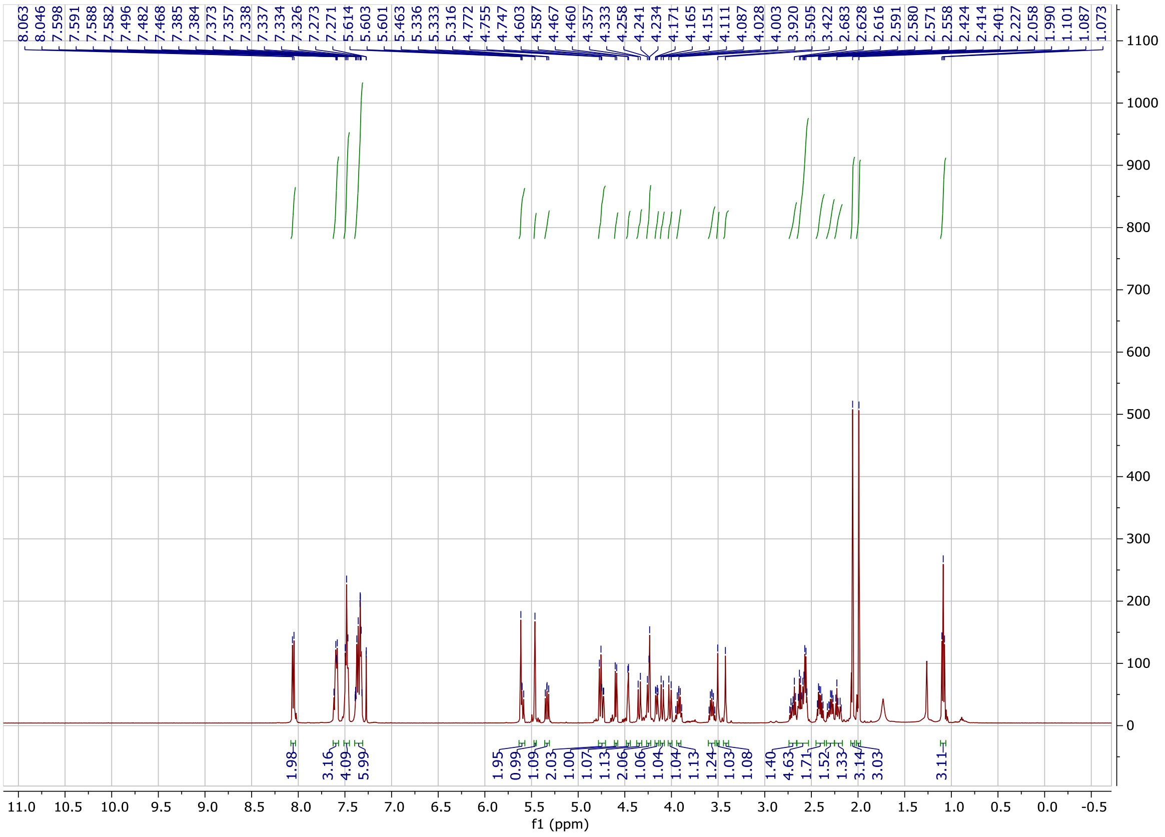

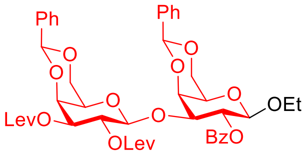

9b

^{13}C-NMR ($CDCl_3$, 125 MHz) of **9b**

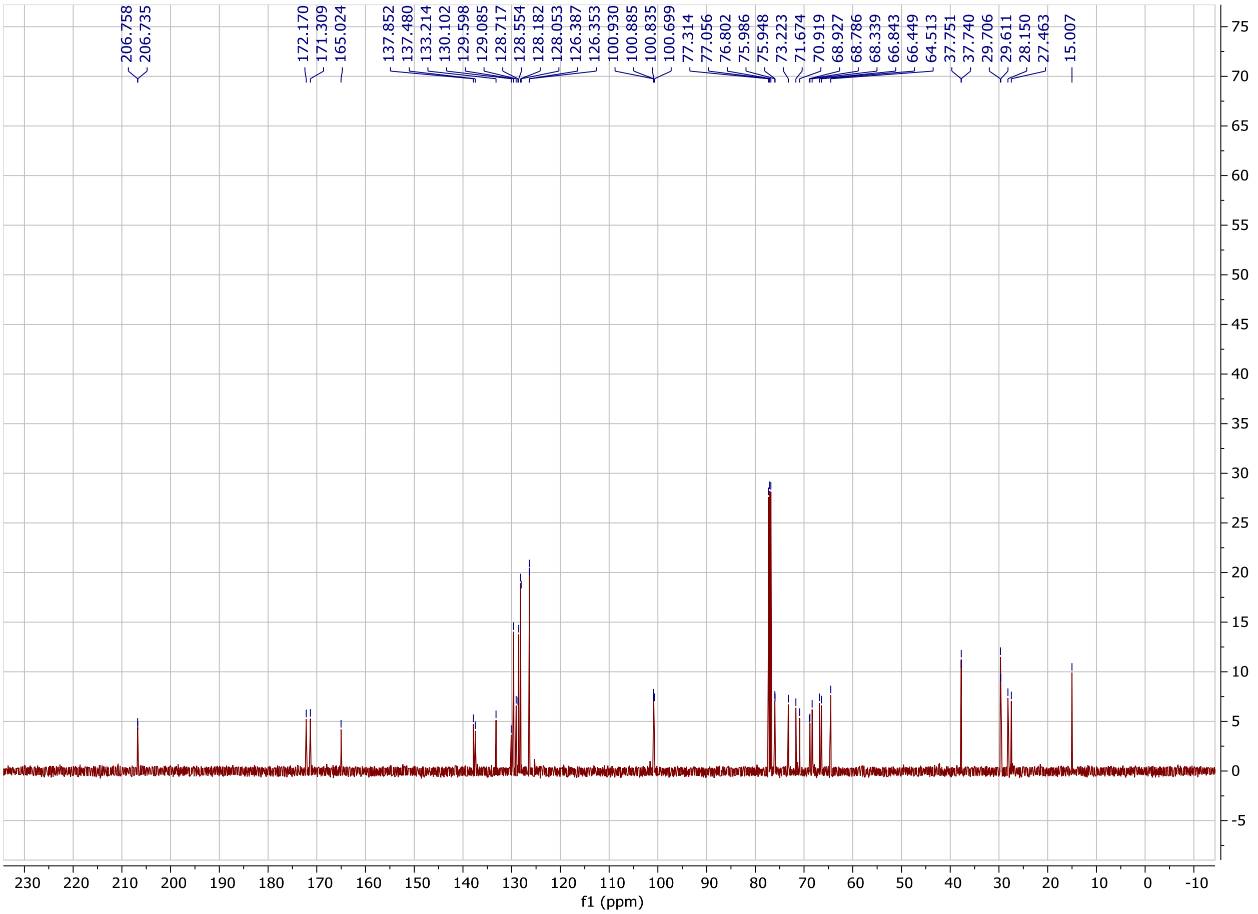

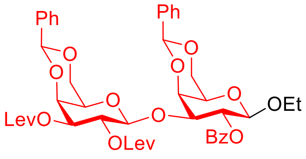

9b

coupled gHSQC ($CDCl_3$, 500 MHz) of **9b**

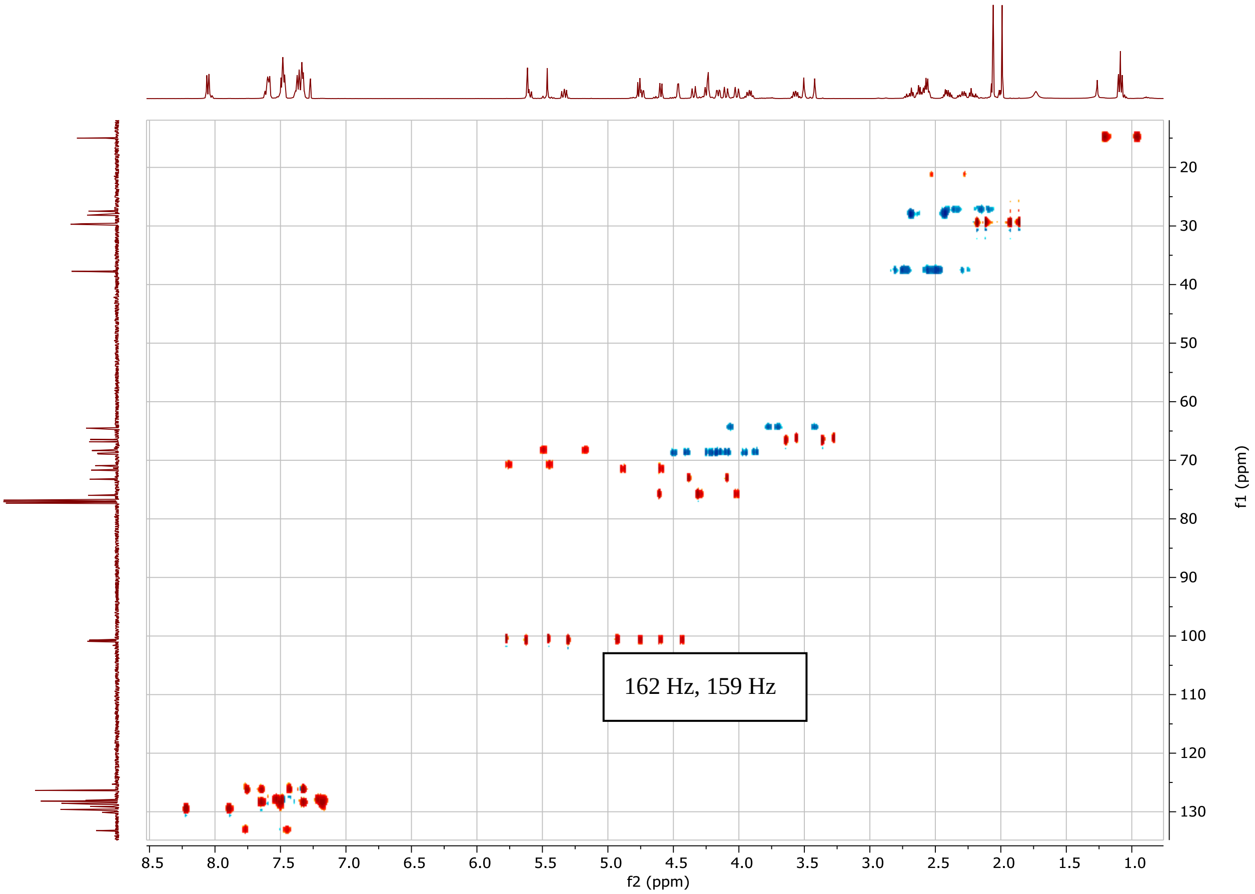

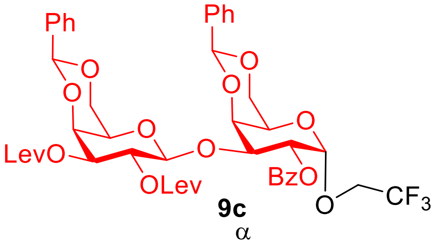

9c
α

^{1}H-NMR ($CDCl_3$, 500 MHz) of **9c-α**

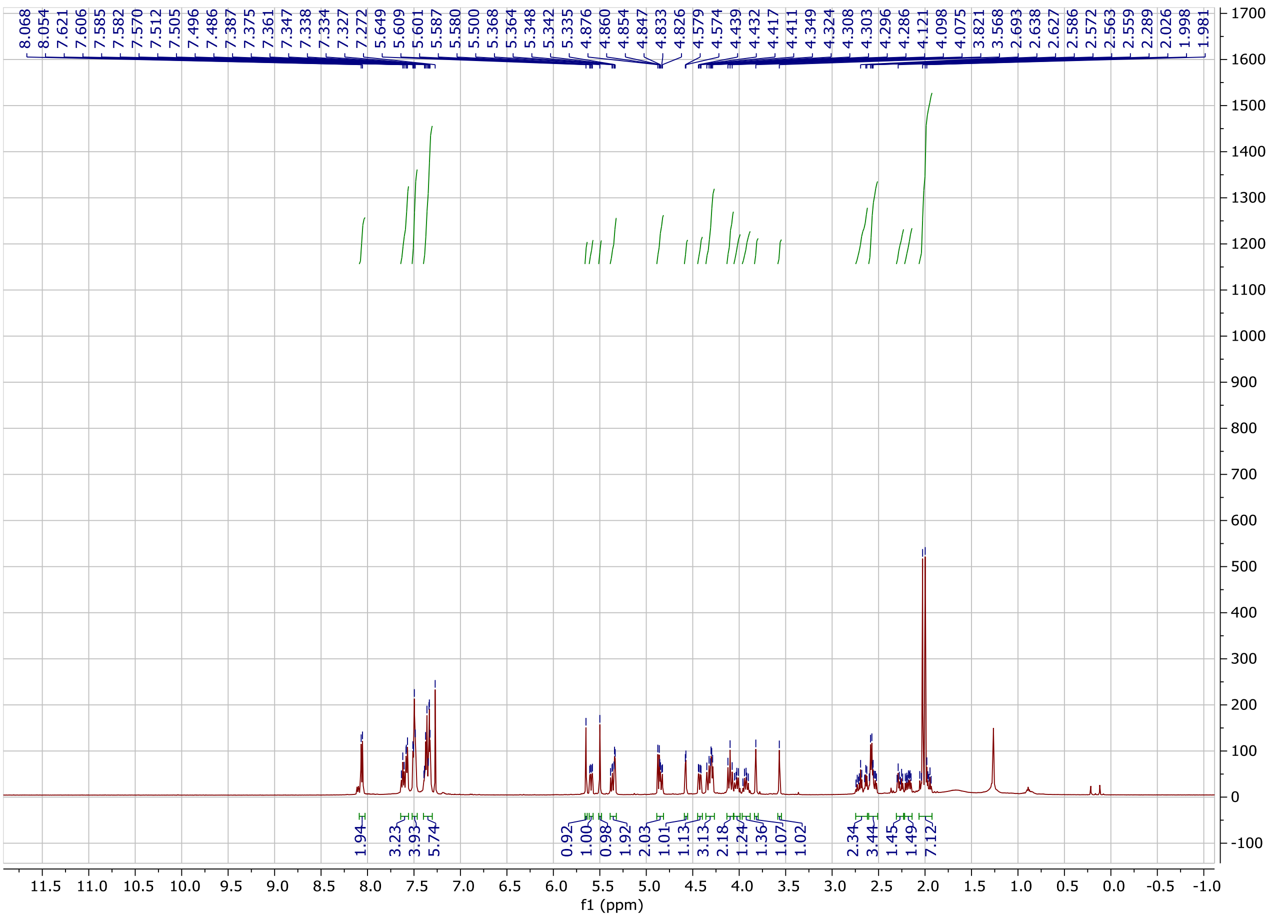

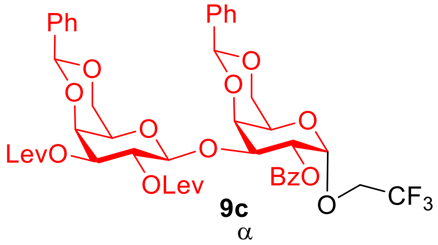

^{13}C-NMR ($CDCl_3$, 125 MHz) of **9c-α**

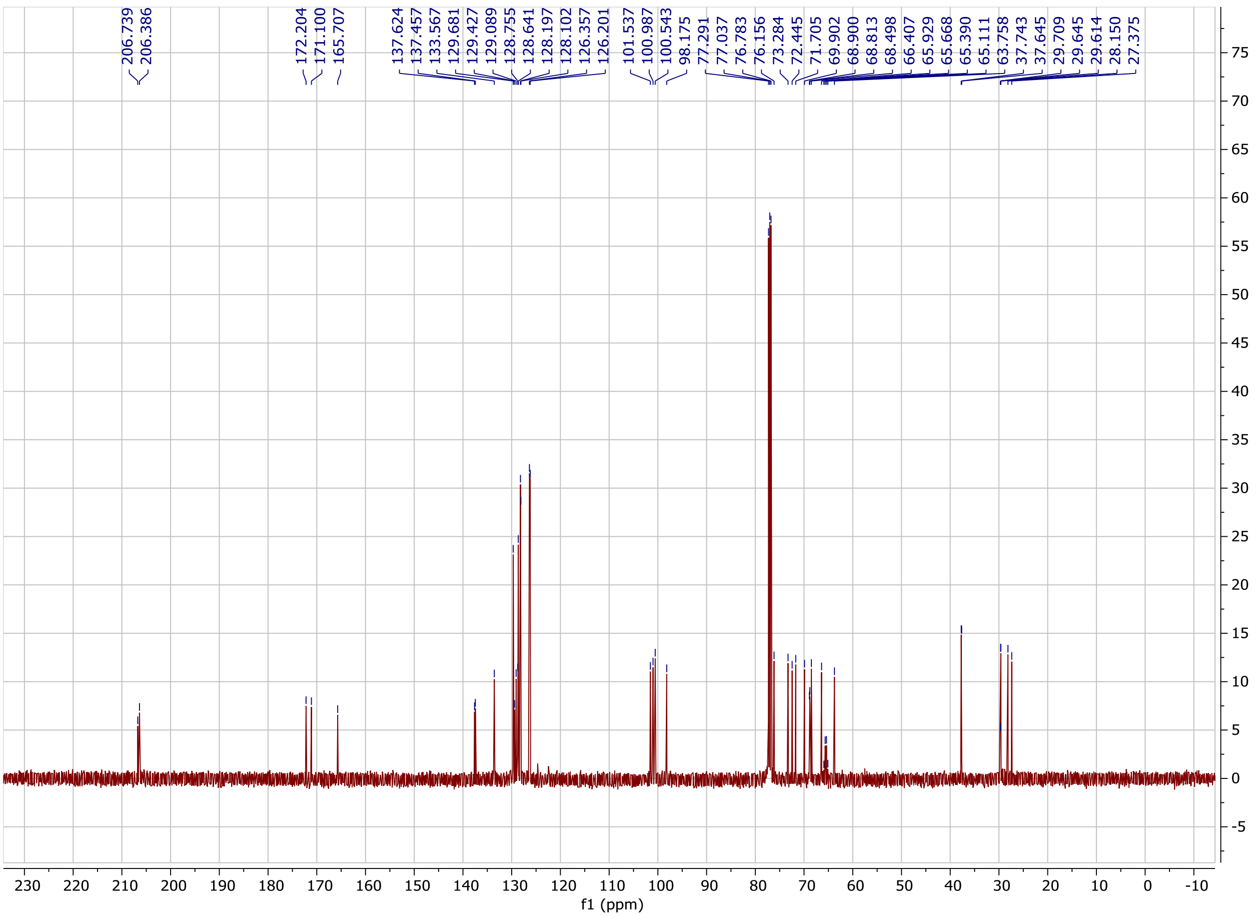

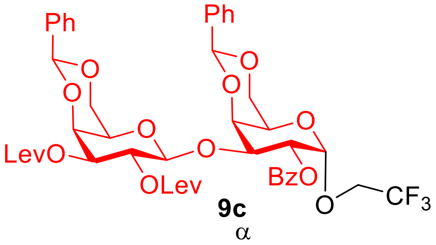

9c
α

coupled gHSQC (CDCl$_3$, 500 MHz) of **9c-α**

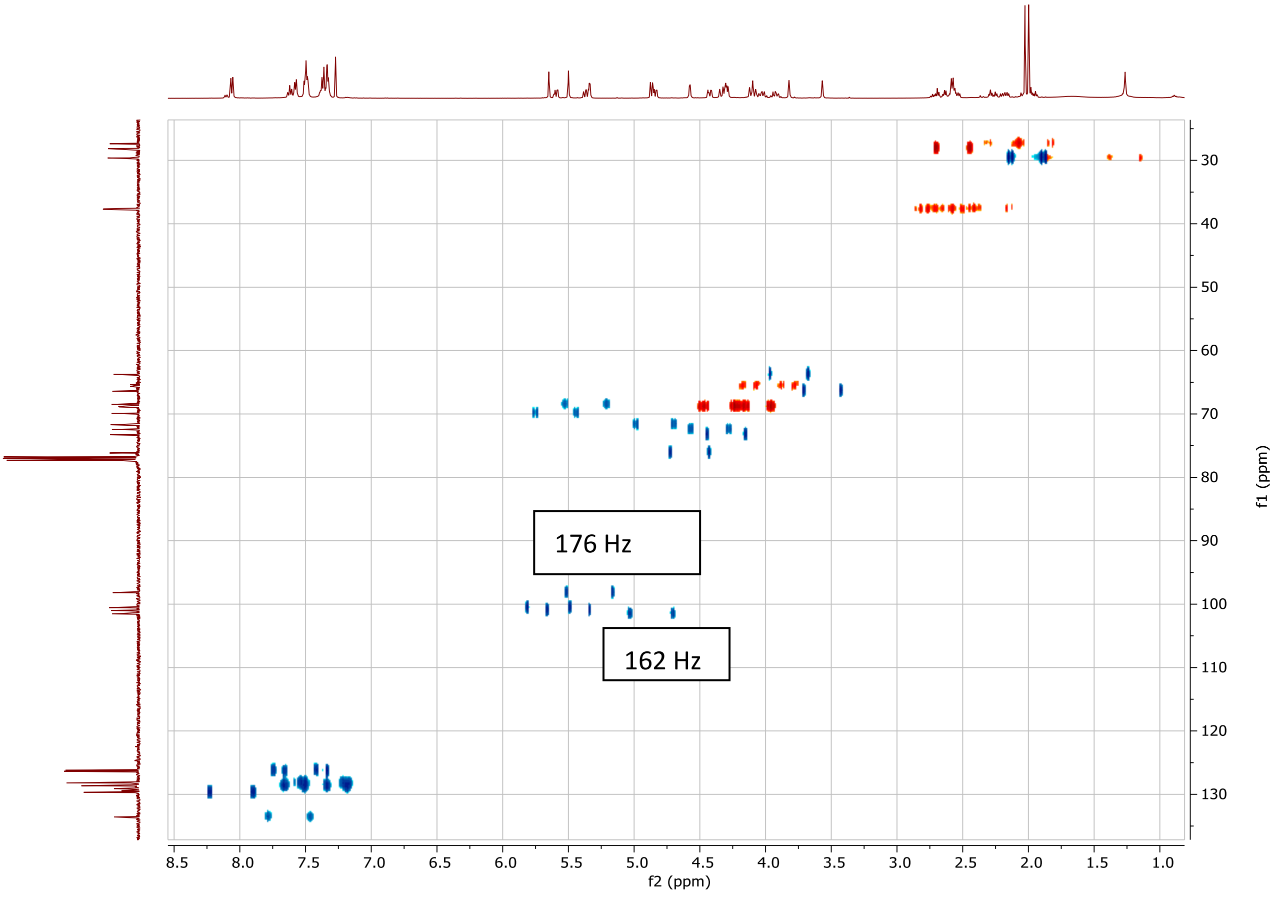

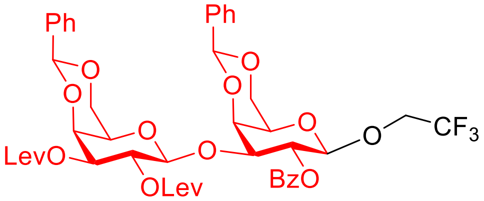

9c
β

^{1}H-NMR (CDCl$_3$, 500 MHz) of **9c-β**

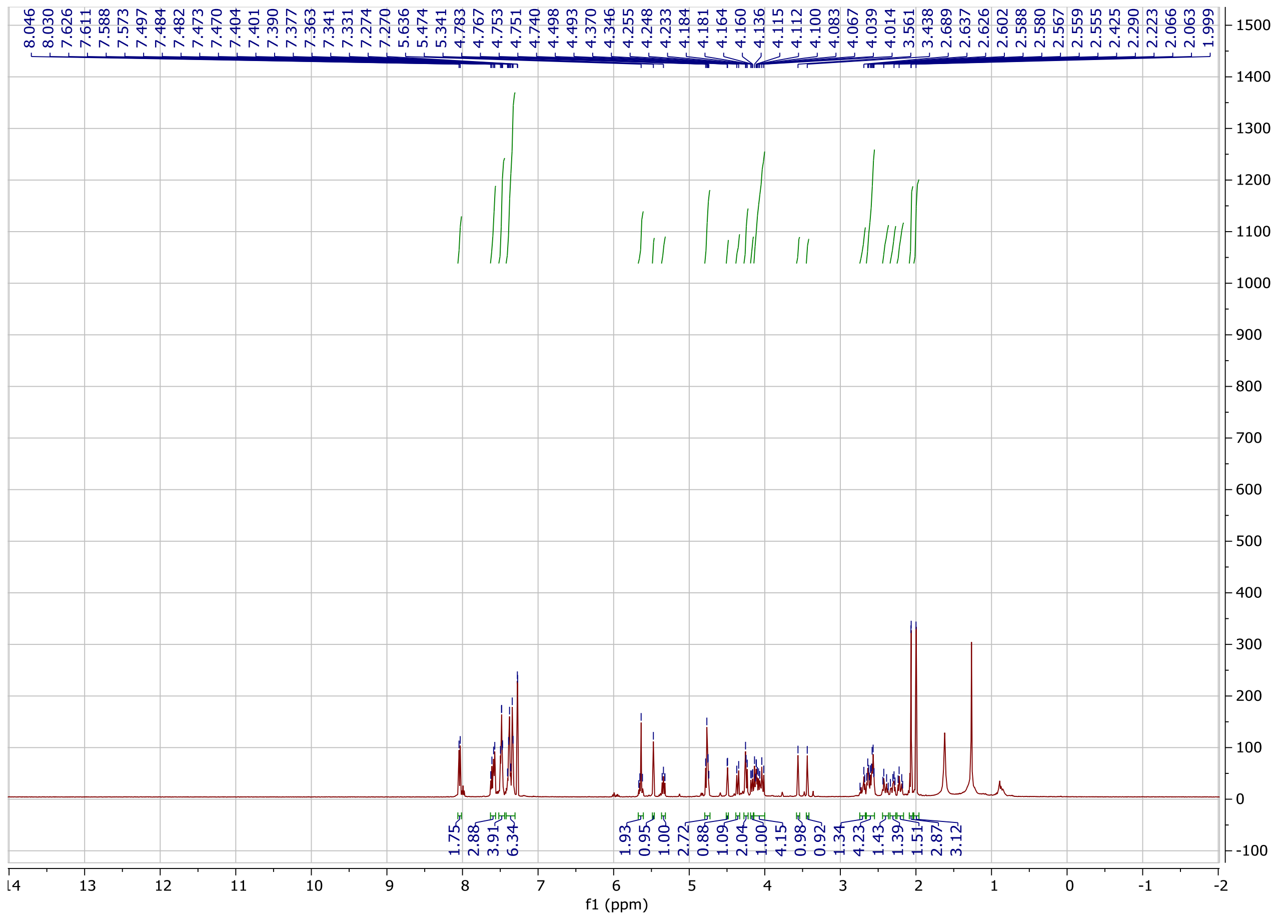

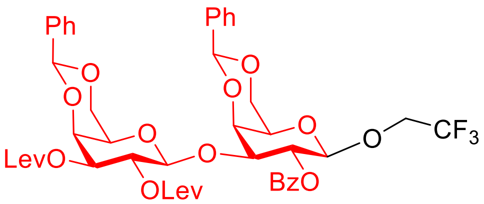

9c

β

^{13}C-NMR ($CDCl_3$, 125 MHz) of **9c-β**

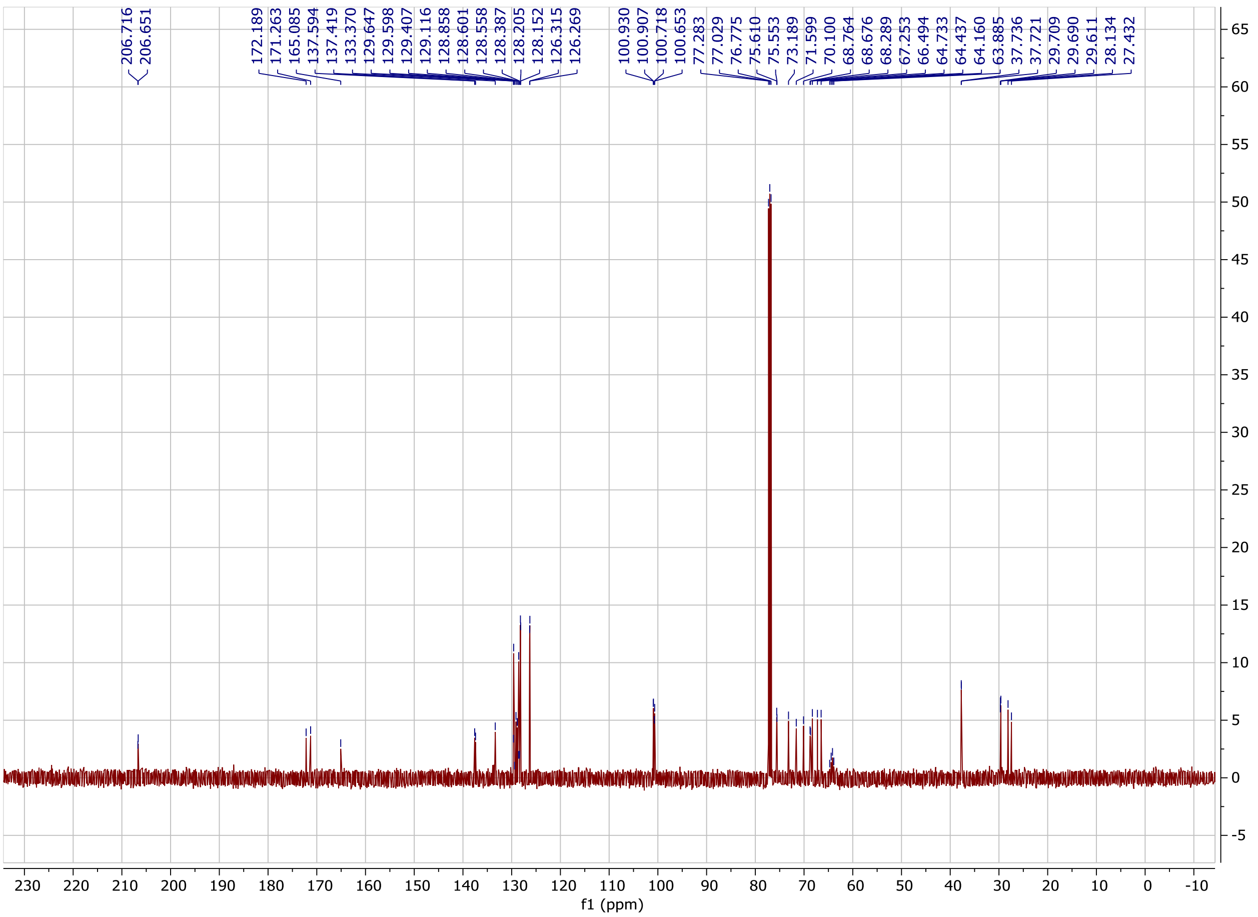

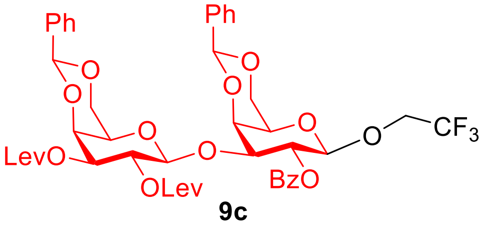

9c
β

coupled gHSQC ($CDCl_3$, 500 MHz) of **9c-β**

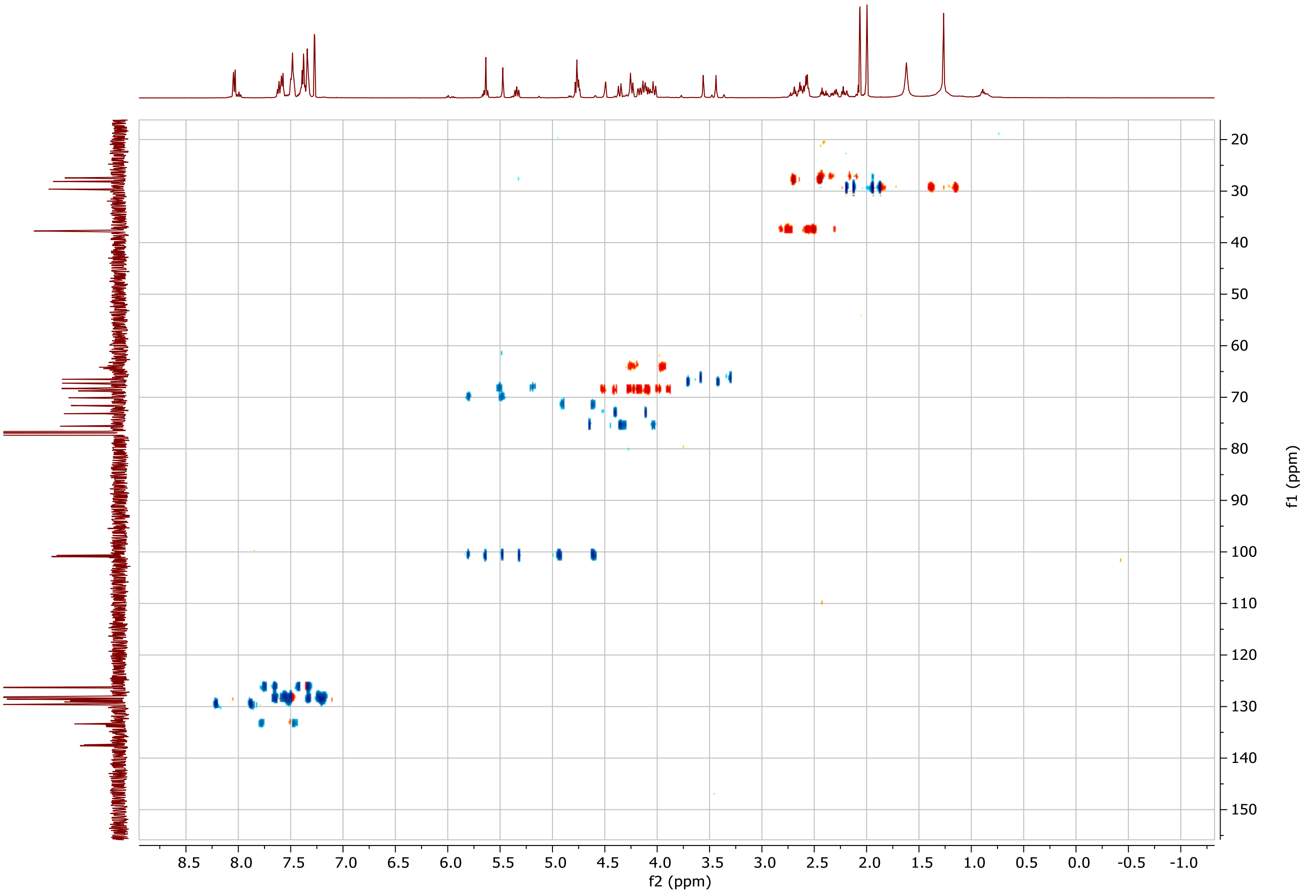

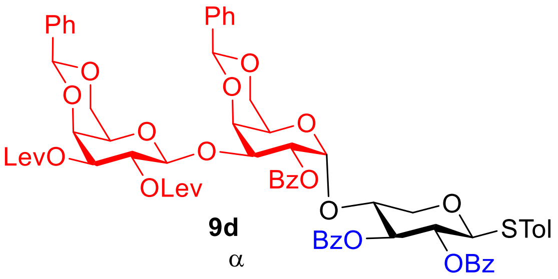

^{1}H-NMR (CDCl$_3$, 500 MHz) of **9d-α**

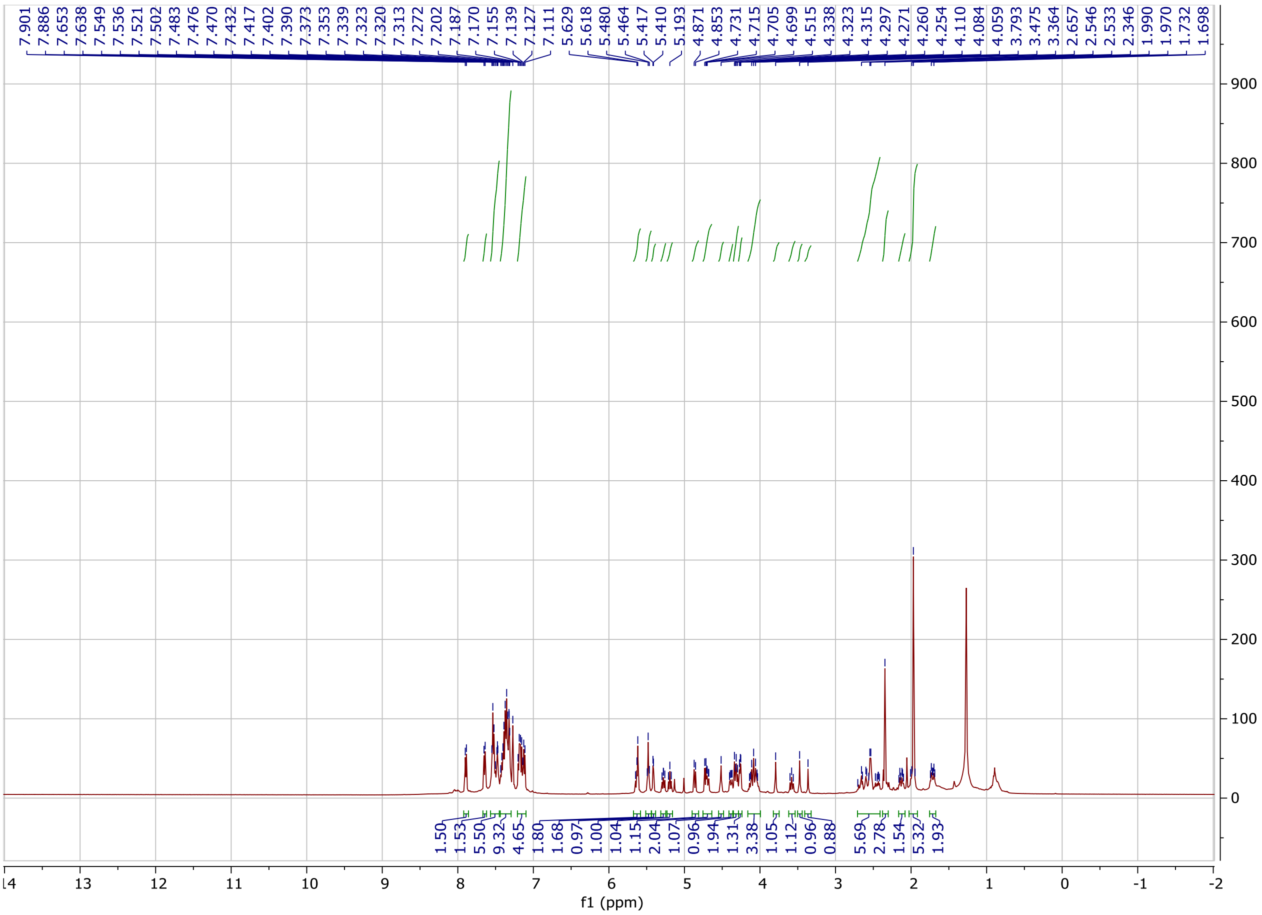

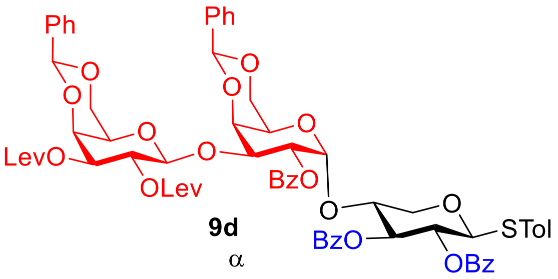

^{13}C-NMR ($CDCl_3$, 125 MHz) of **9d-α**

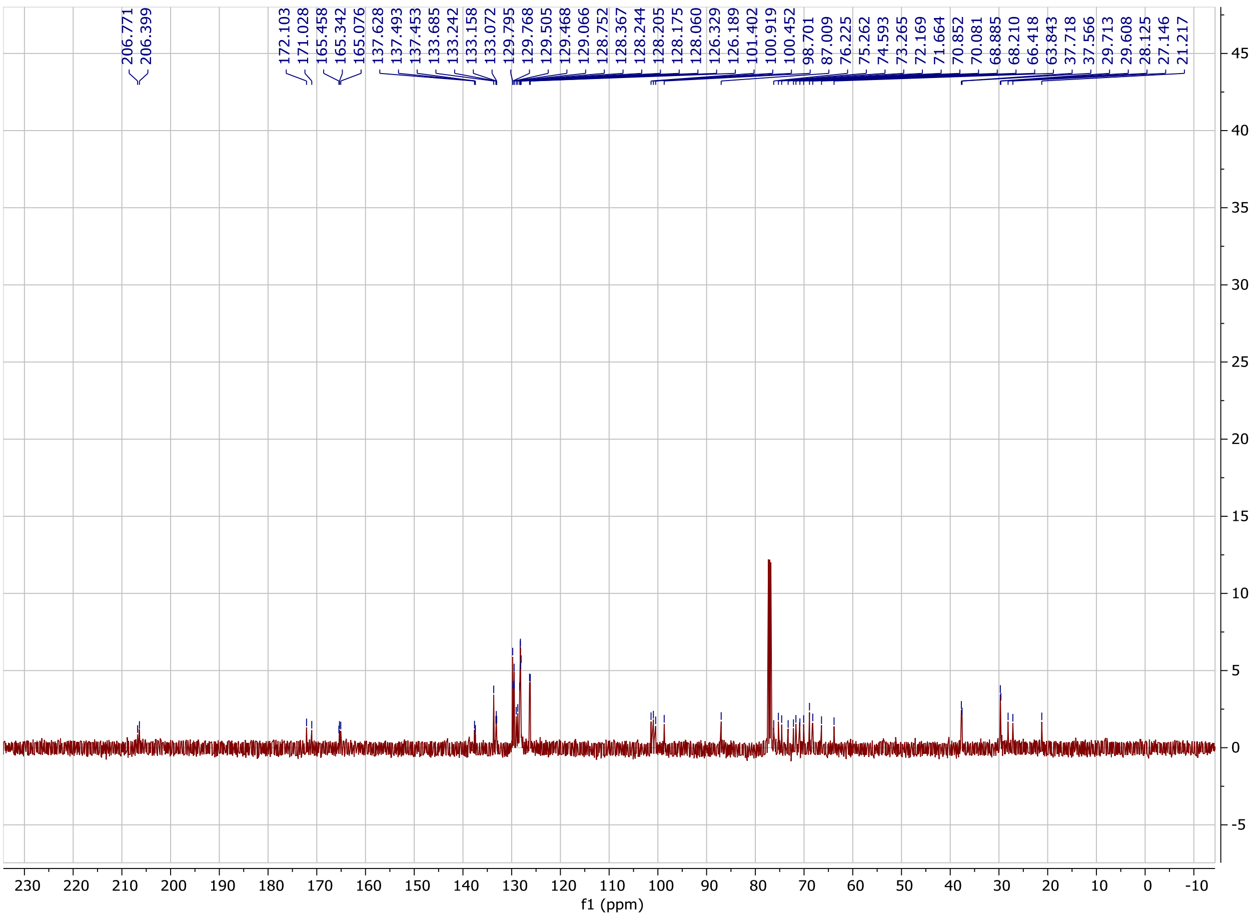

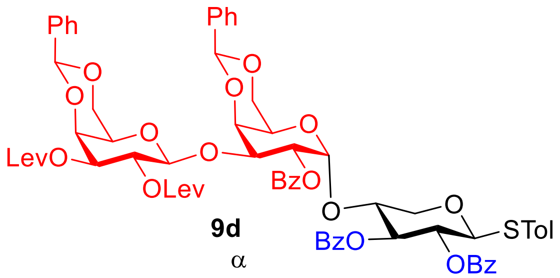

9d
α

coupled gHSQC ($CDCl_3$, 500 MHz) of **9d-α**

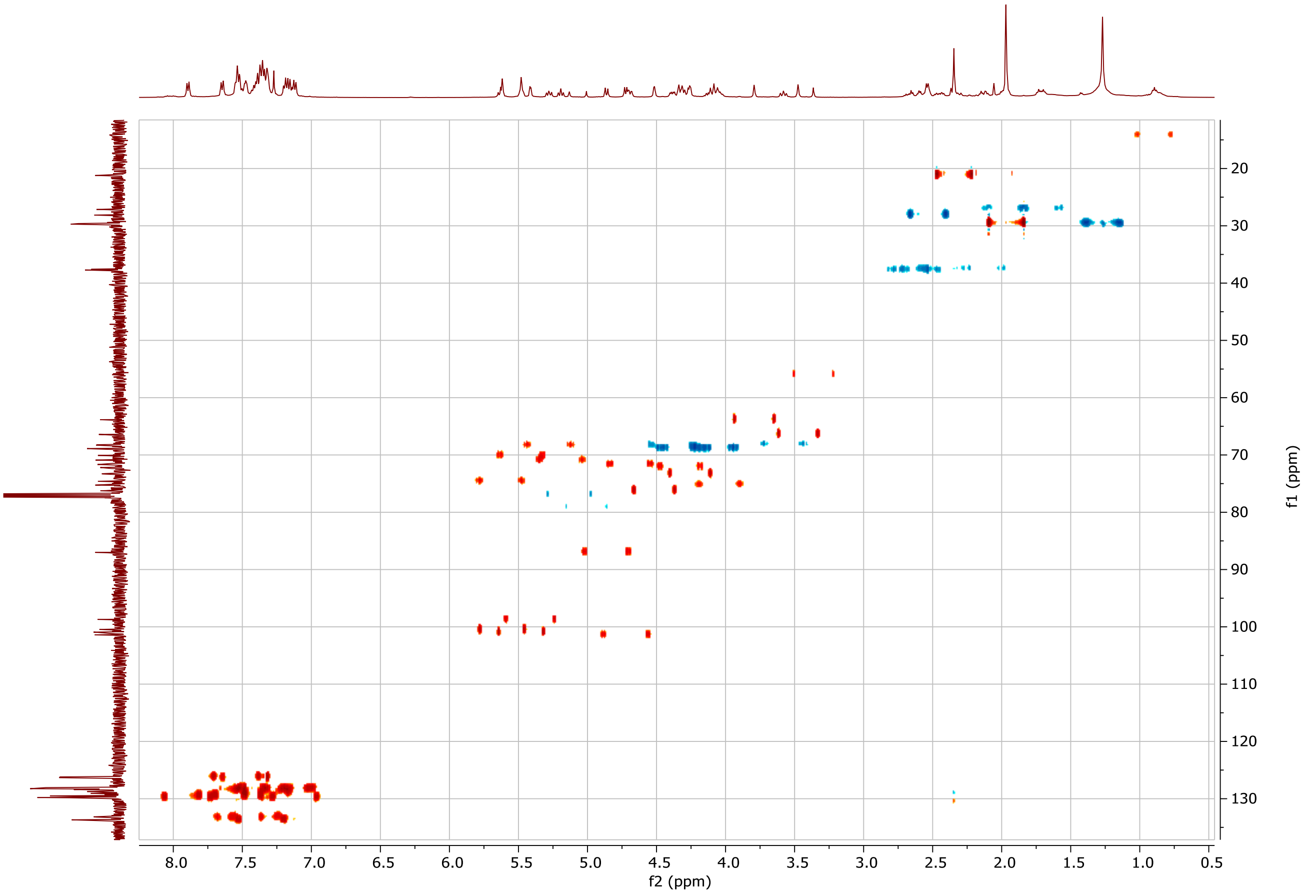

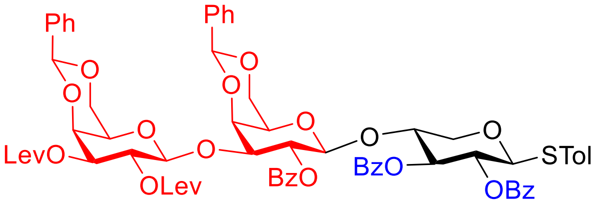

9d β

^{1}H-NMR ($CDCl_3$, 500 MHz) of **9d-β**

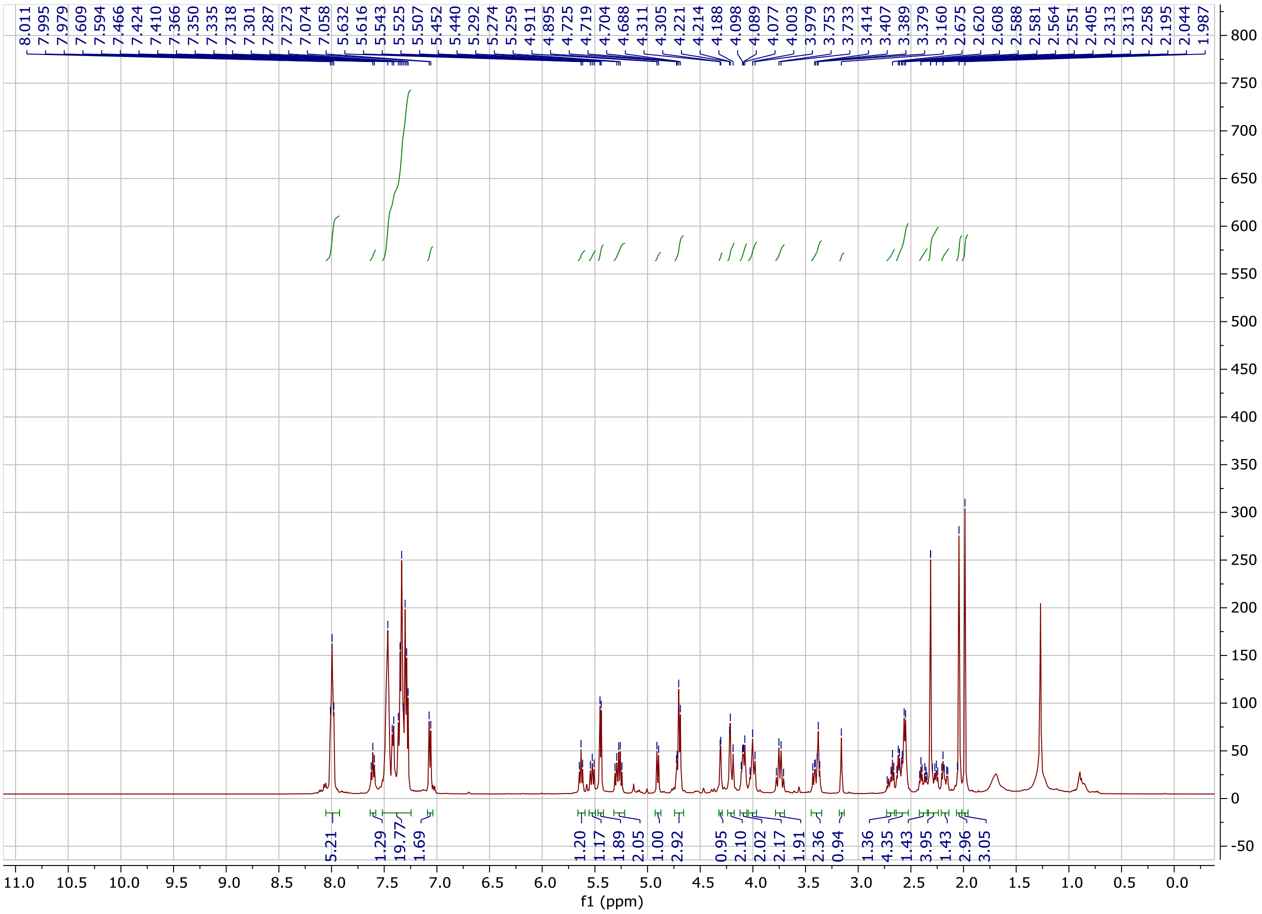

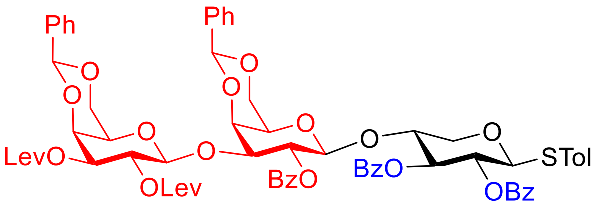

9d β

^{13}C-NMR ($CDCl_3$, 125 MHz) of **9d-β**

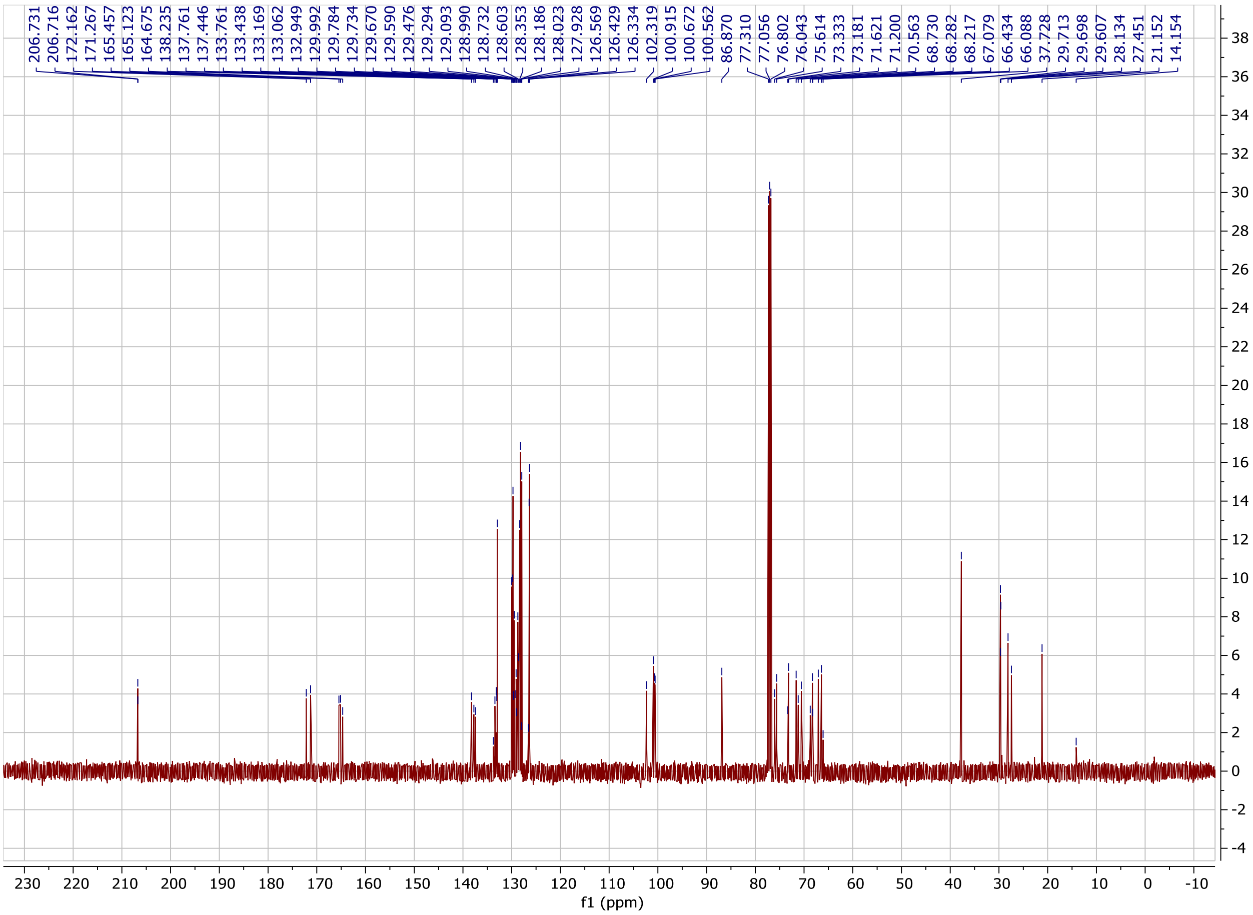

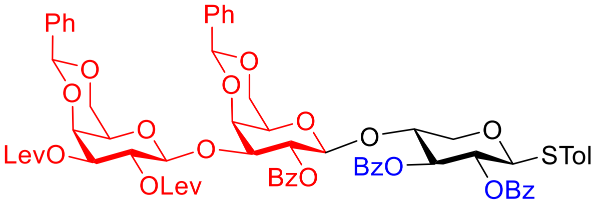

9d β

coupled gHSQC ($CDCl_3$, 500 MHz) of **9d-β**

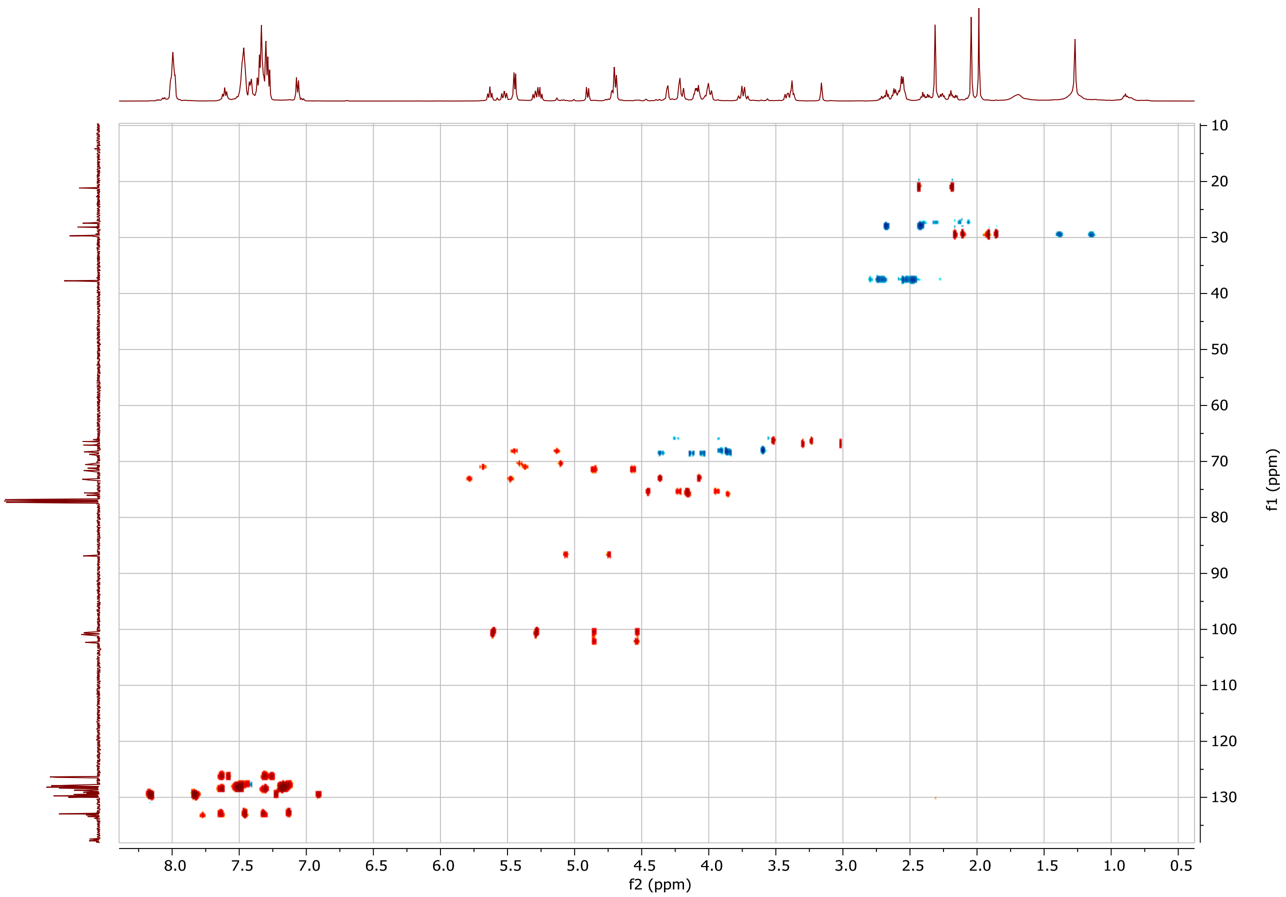

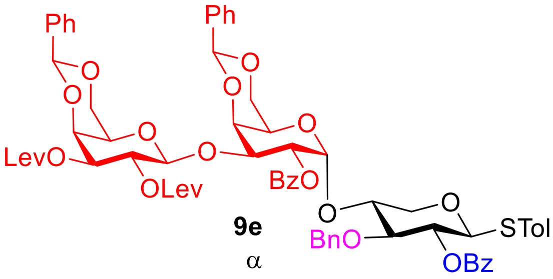

9e
α

^{1}H-NMR ($CDCl_3$, 500 MHz) of **9e-α**

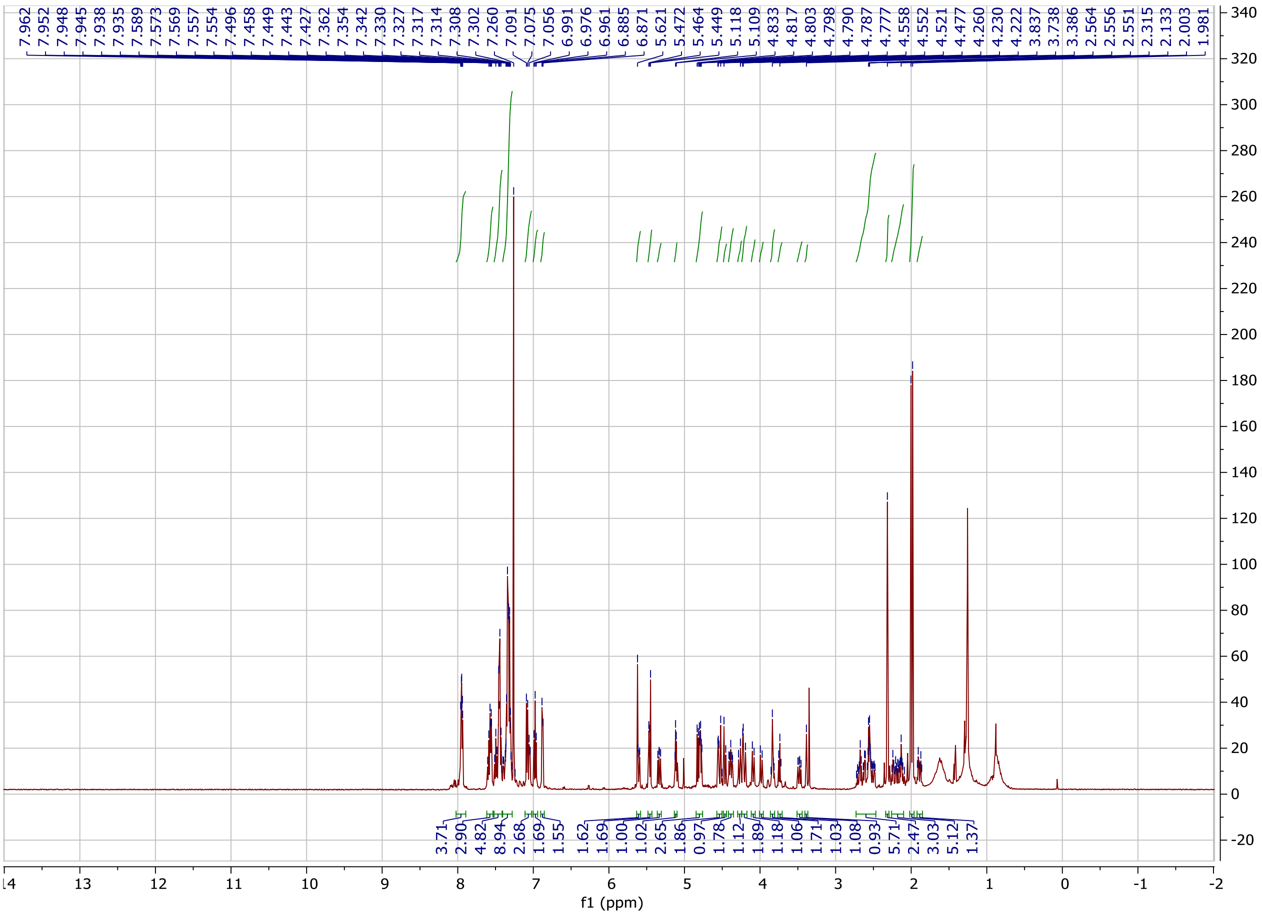

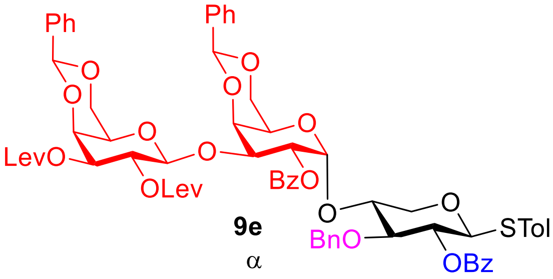

coupled gHSQC (CDCl$_3$, 500 MHz) of **9e-α**

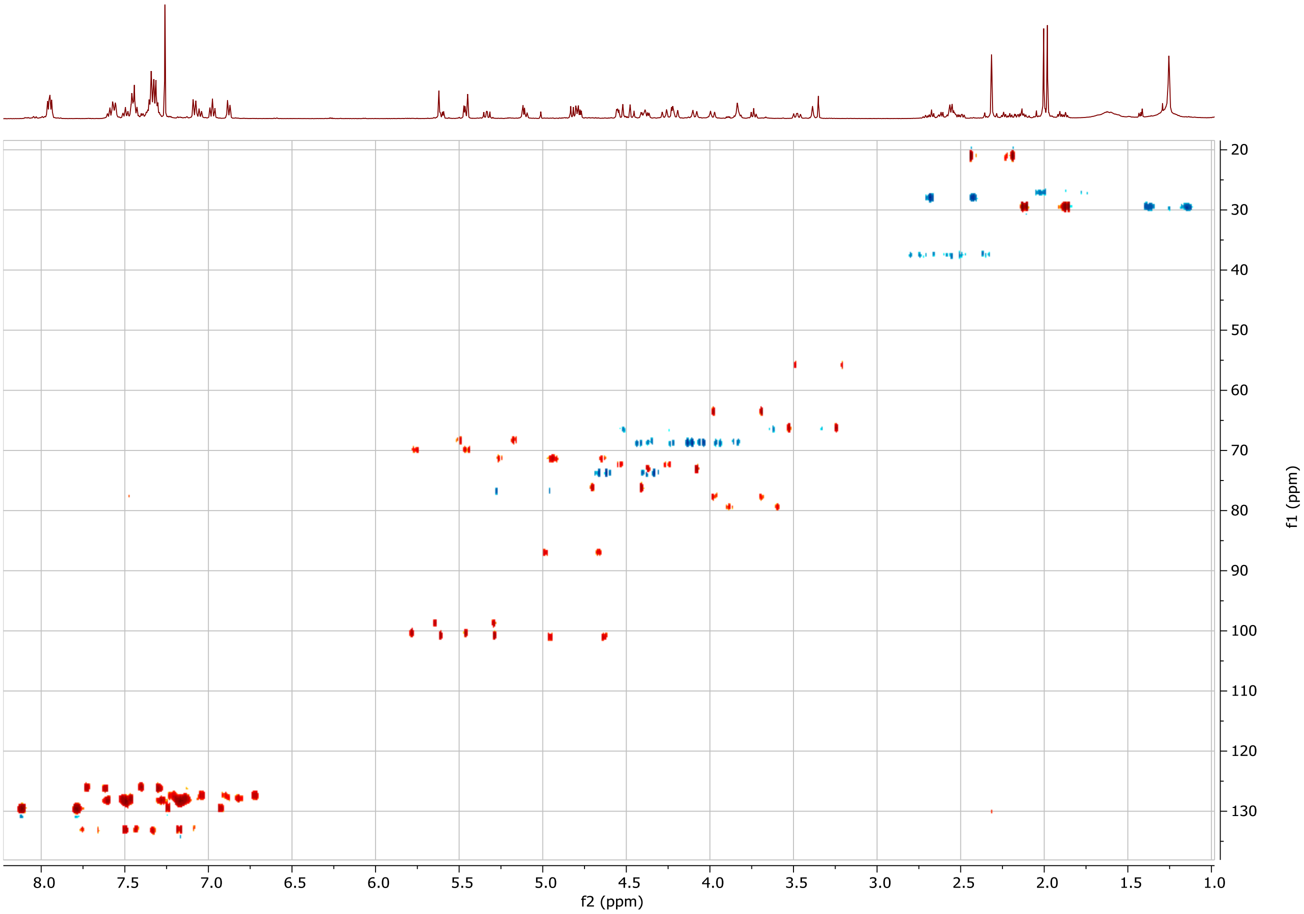

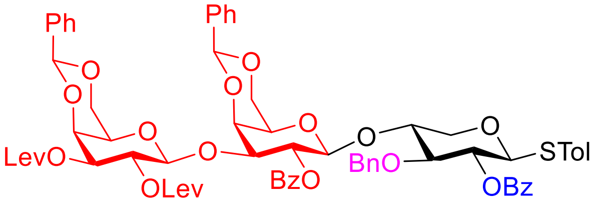

9e

β

^{1}H-NMR ($CDCl_3$, 500 MHz) of **9e-β**

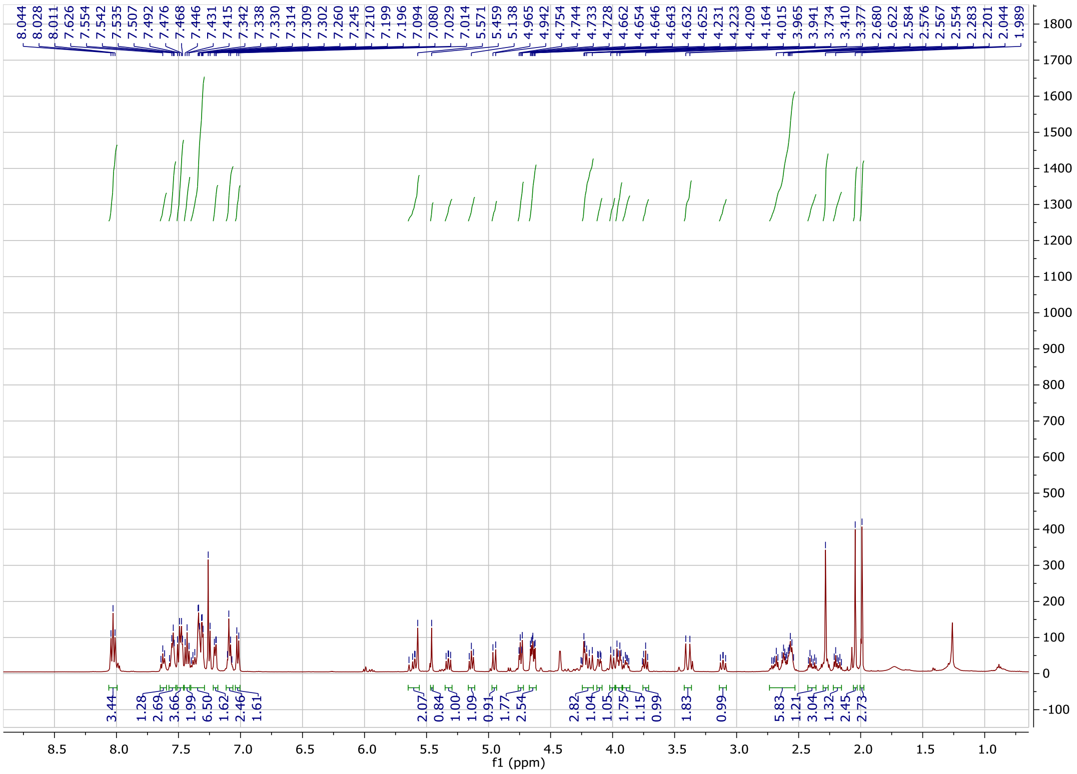

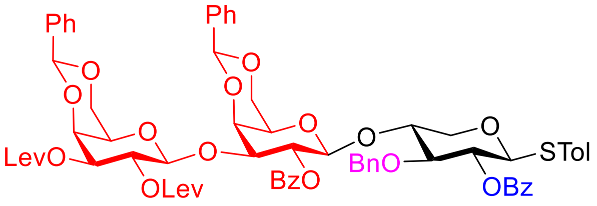

9e

β

^{13}C-NMR ($CDCl_3$, 125 MHz) of **9e-β**

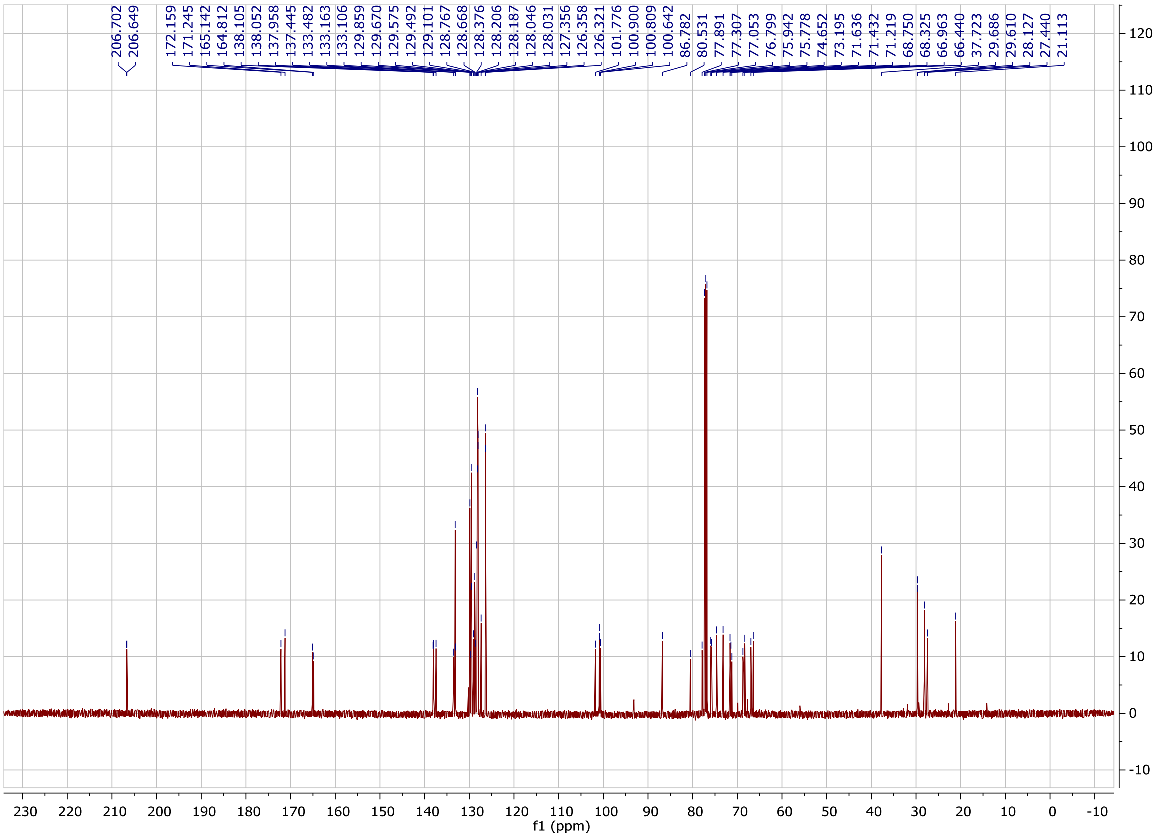

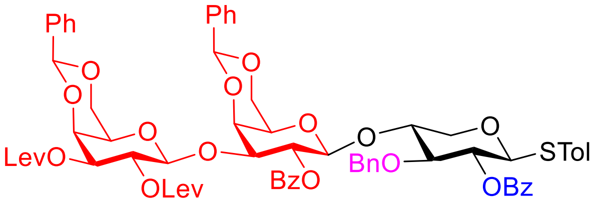

9e

β

coupled gHSQC ($CDCl_3$, 500 MHz) of **9e-β**

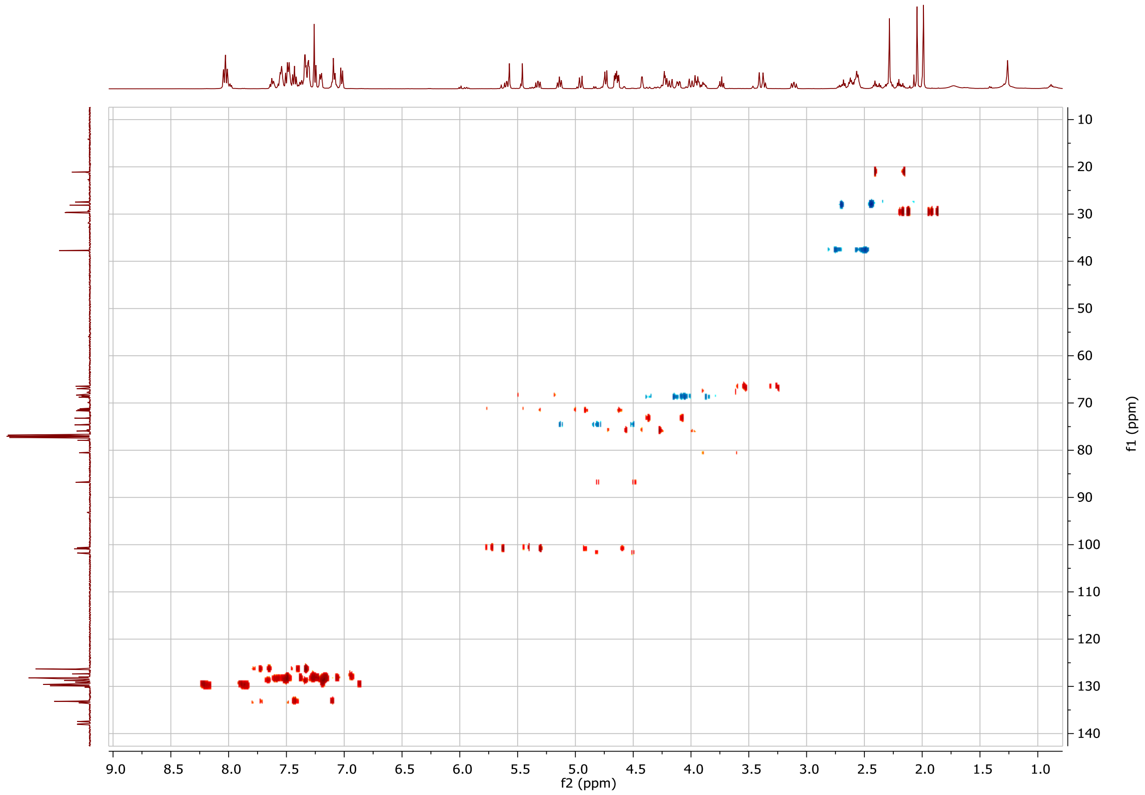

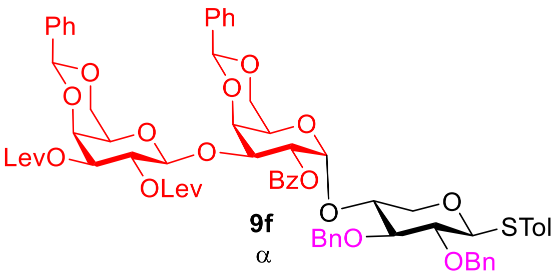

^{1}H-NMR (CDCl$_3$, 500 MHz) of **9f-α**

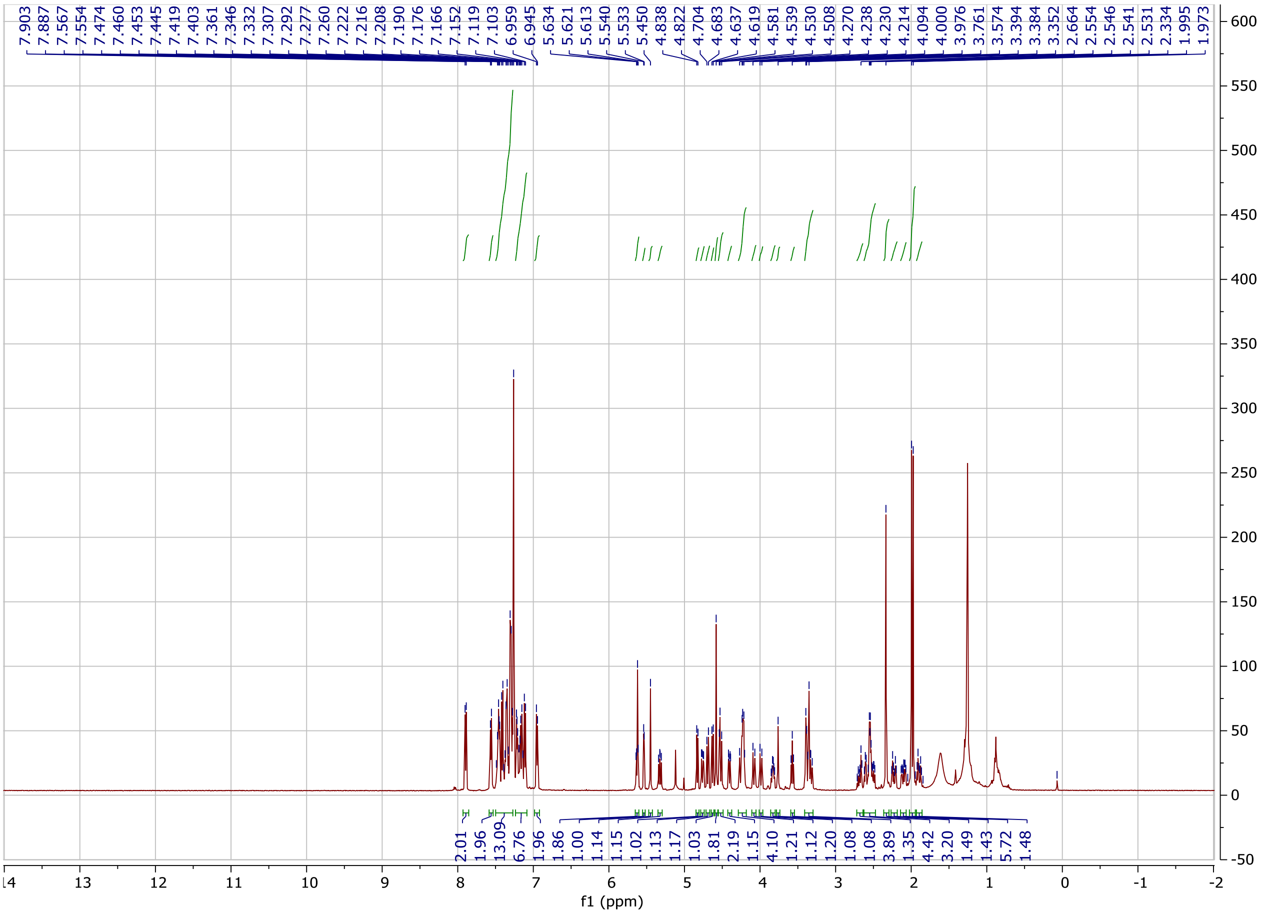

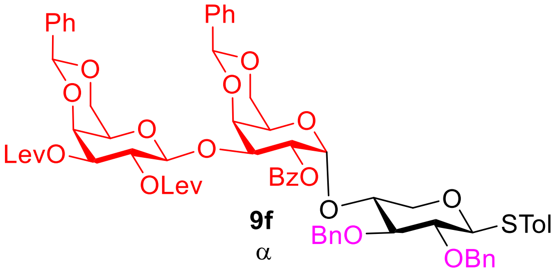

9f
α

coupled gHSQC ($CDCl_3$, 500 MHz) of **9f-α**

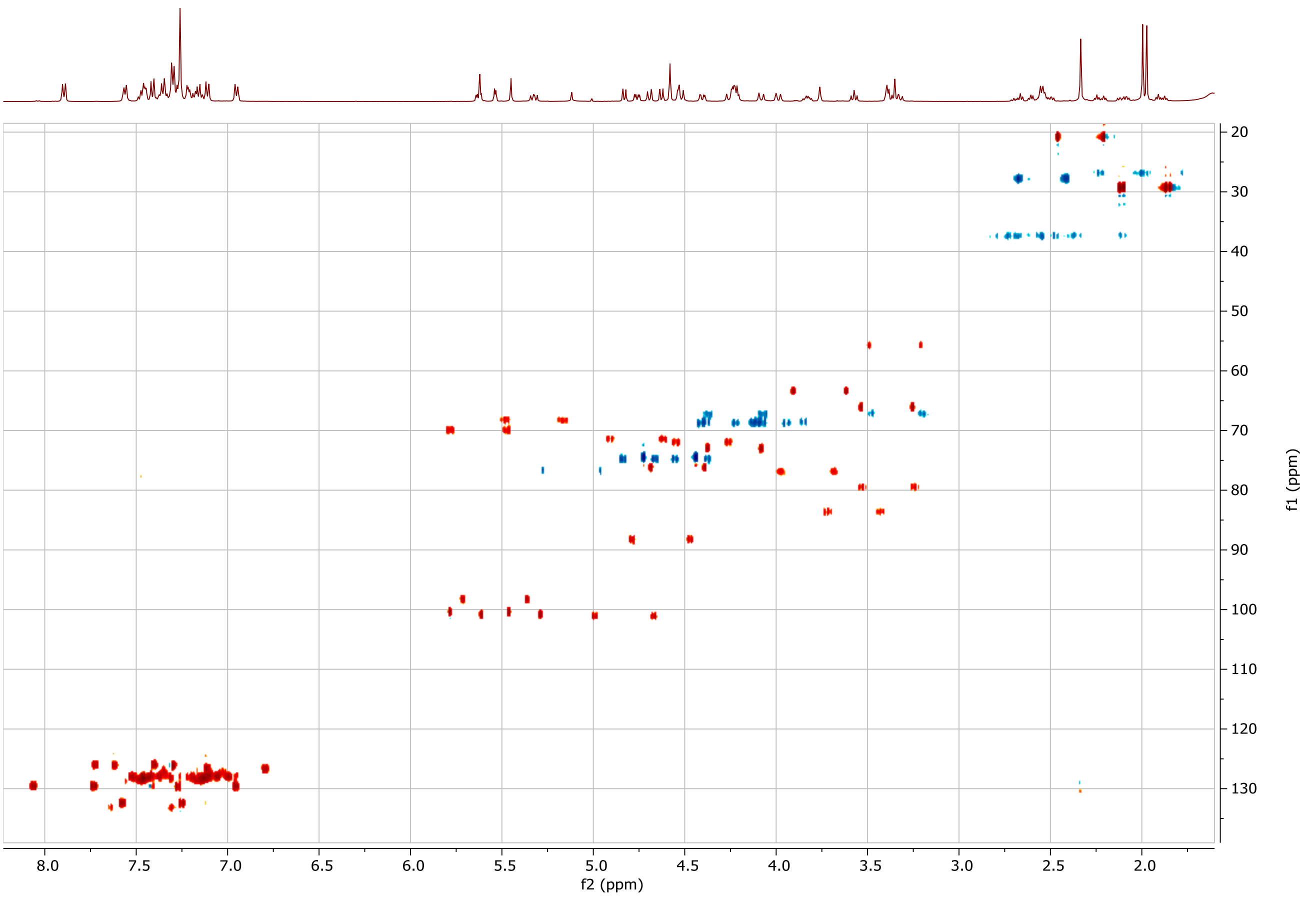

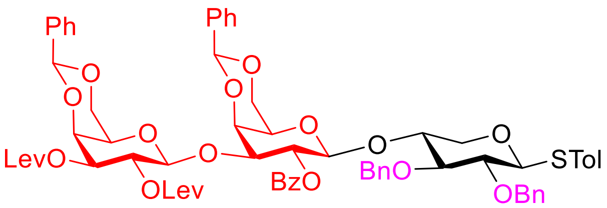

9f
β

^{1}H-NMR ($CDCl_3$, 500 MHz) of **9f-β**

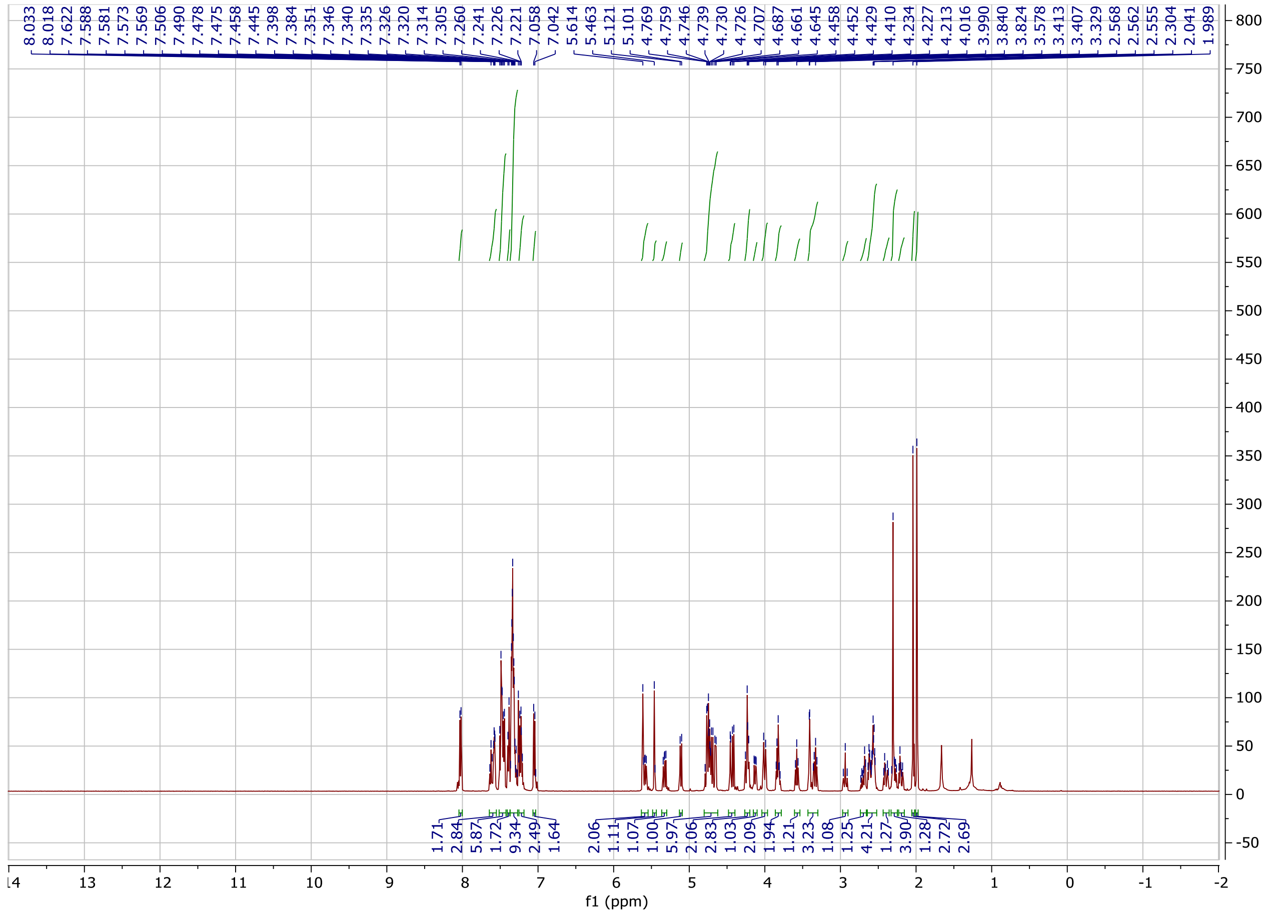

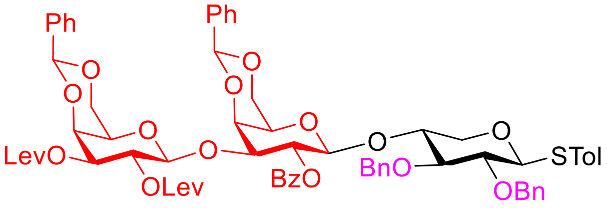

9f
β

coupled gHSQC ($CDCl_3$, 500 MHz) of **9f-β**

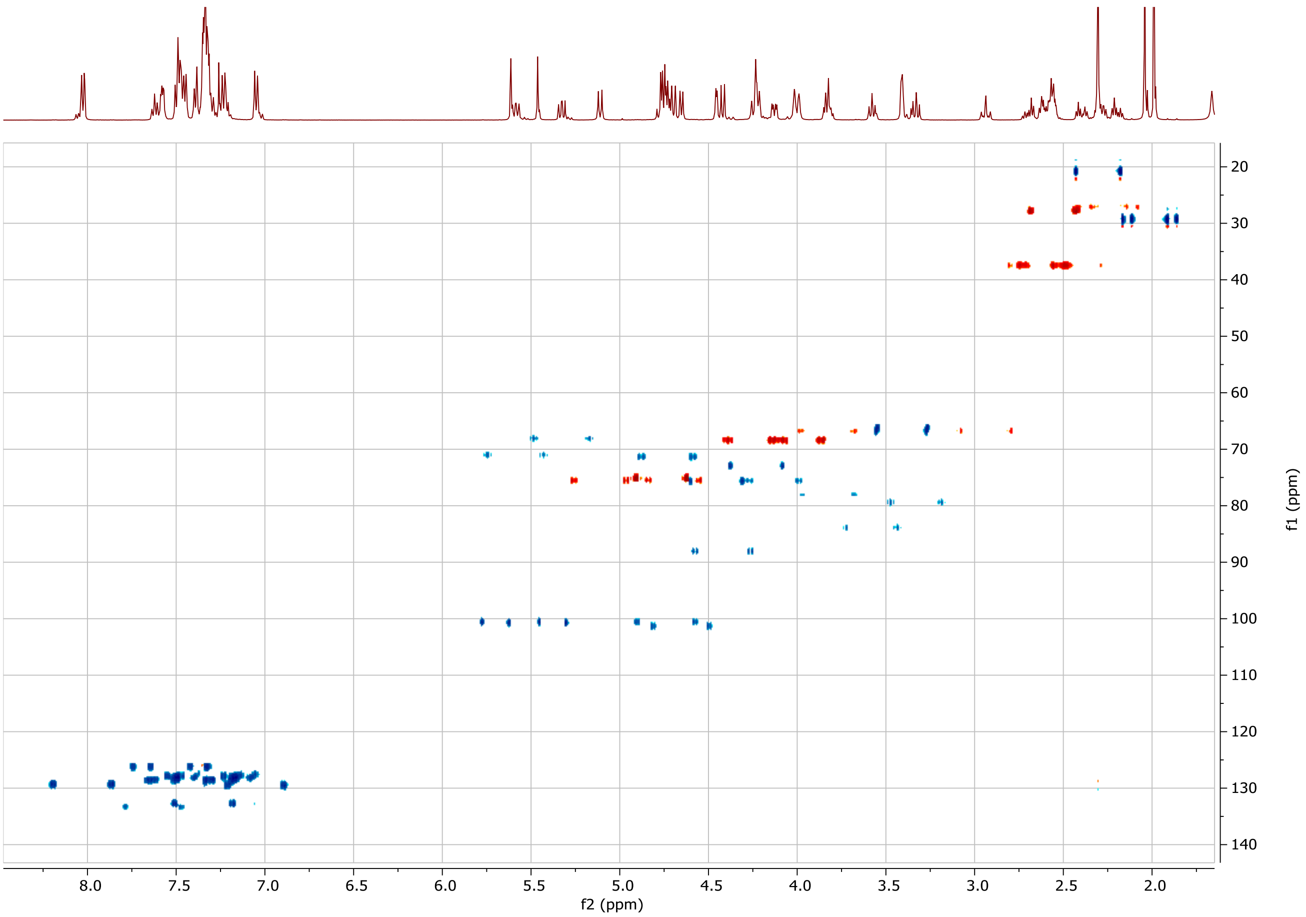

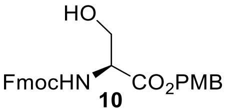

10

^{1}H-NMR ($CDCl_3$, 500 MHz) of **10**

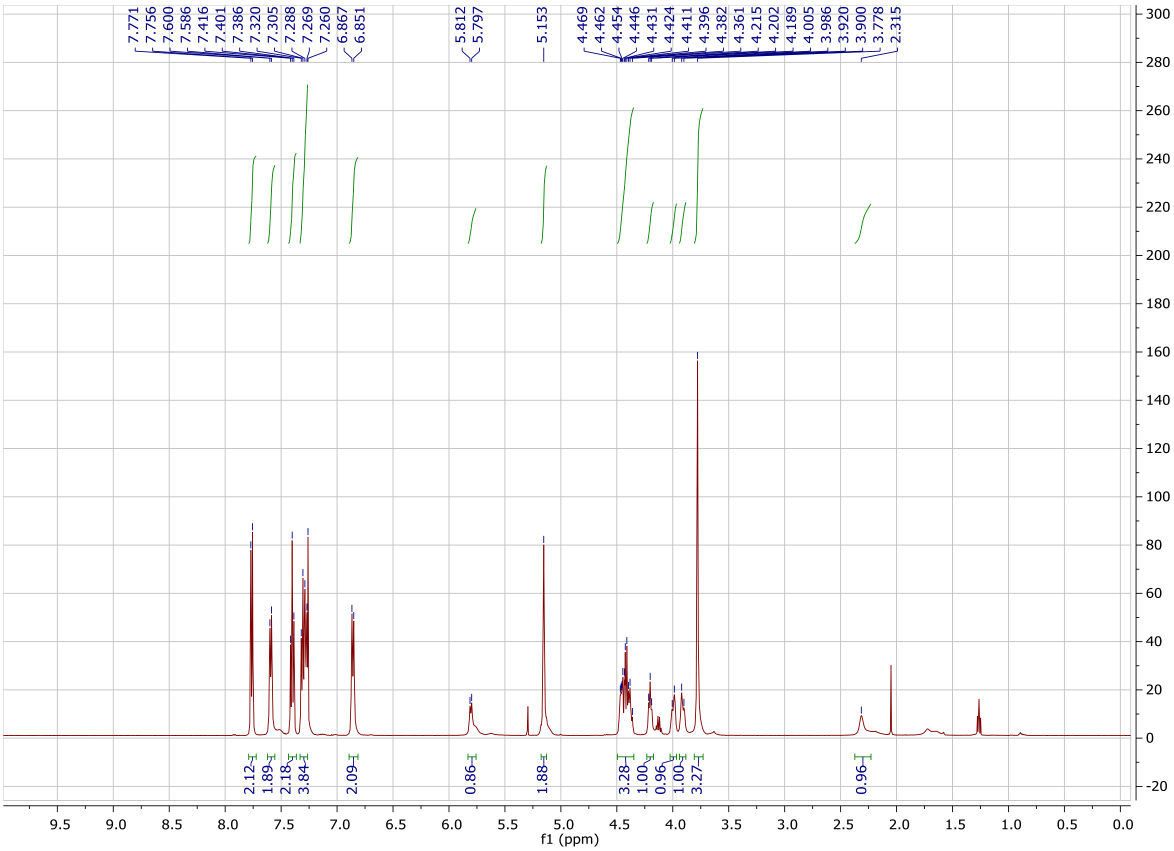

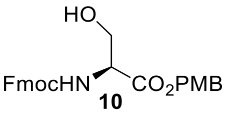

10

^{13}C-NMR ($CDCl_3$, 125 MHz) of **10**

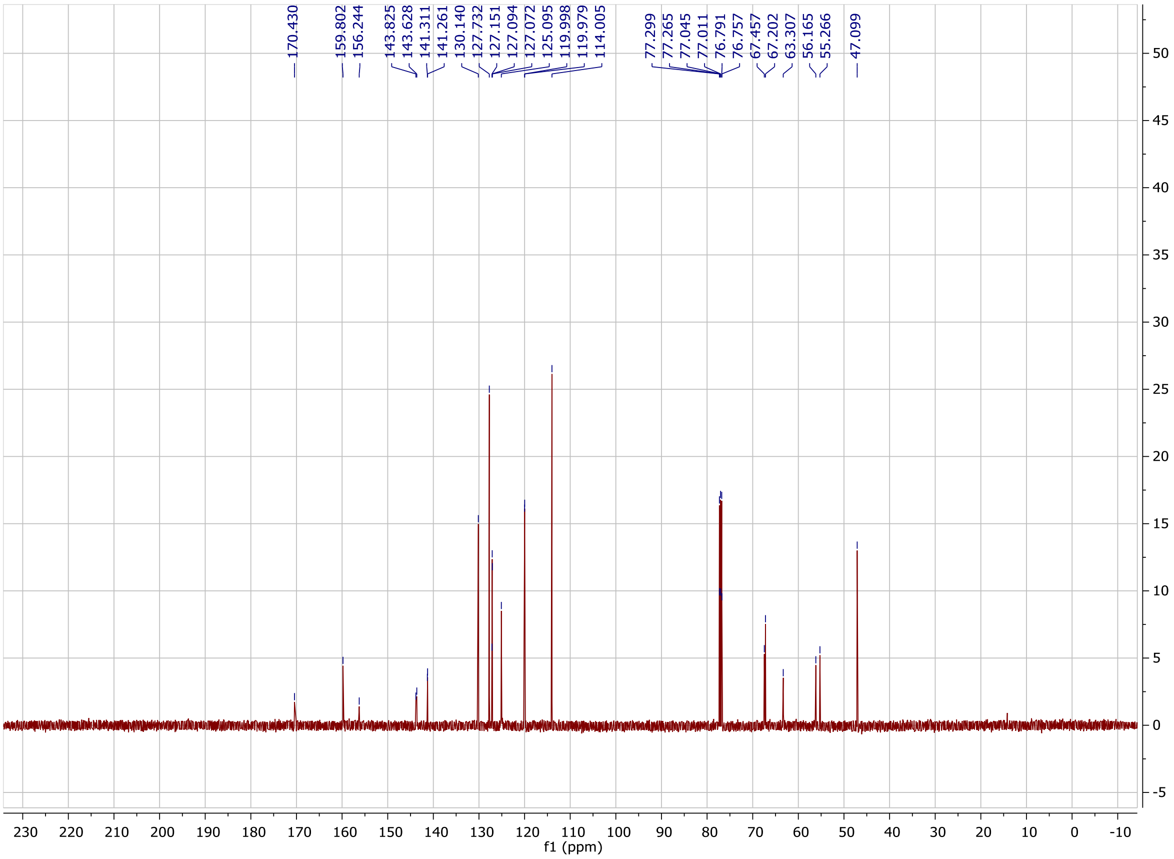

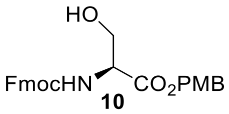

gCOSY ($CDCl_3$, 500 MHz) of **10**

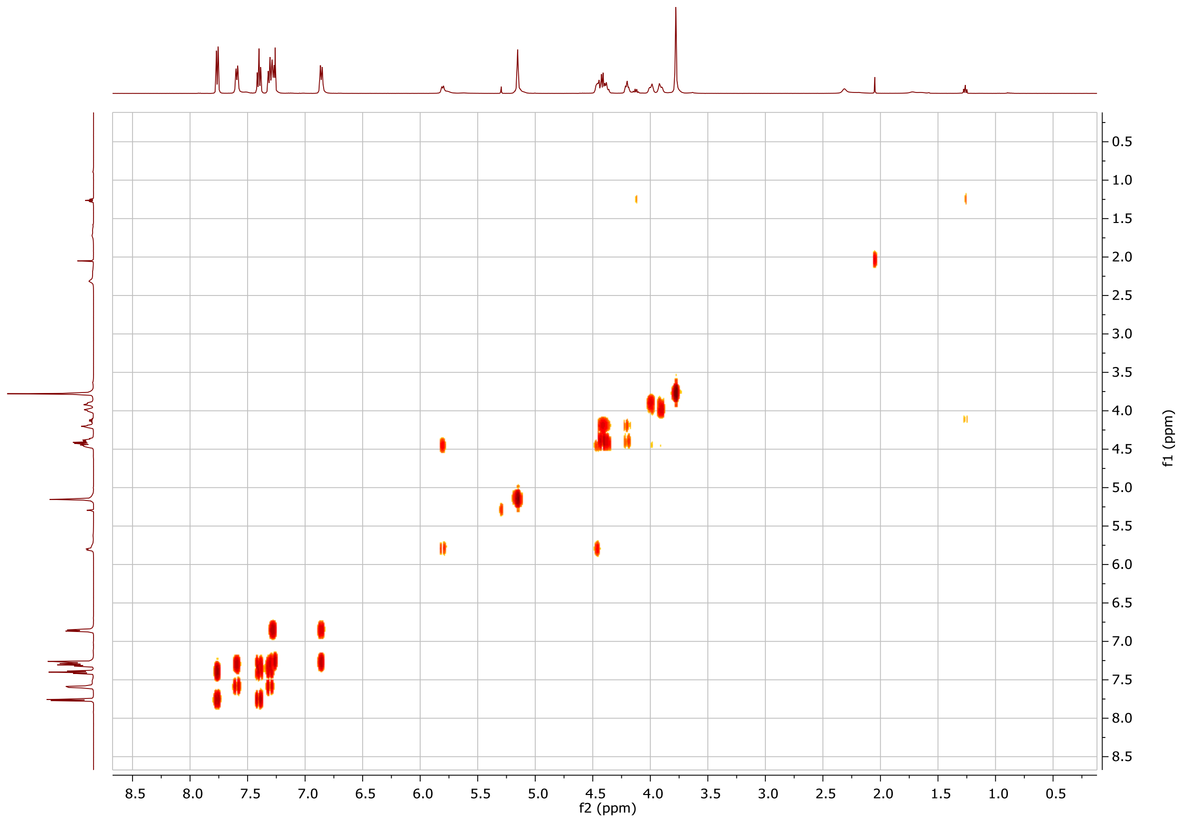

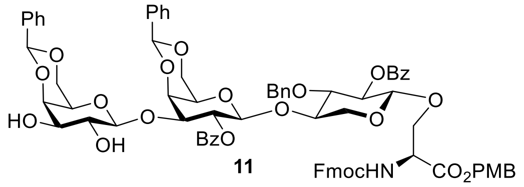

11

^{1}H-NMR ($CDCl_3$, 500 MHz) of **11**

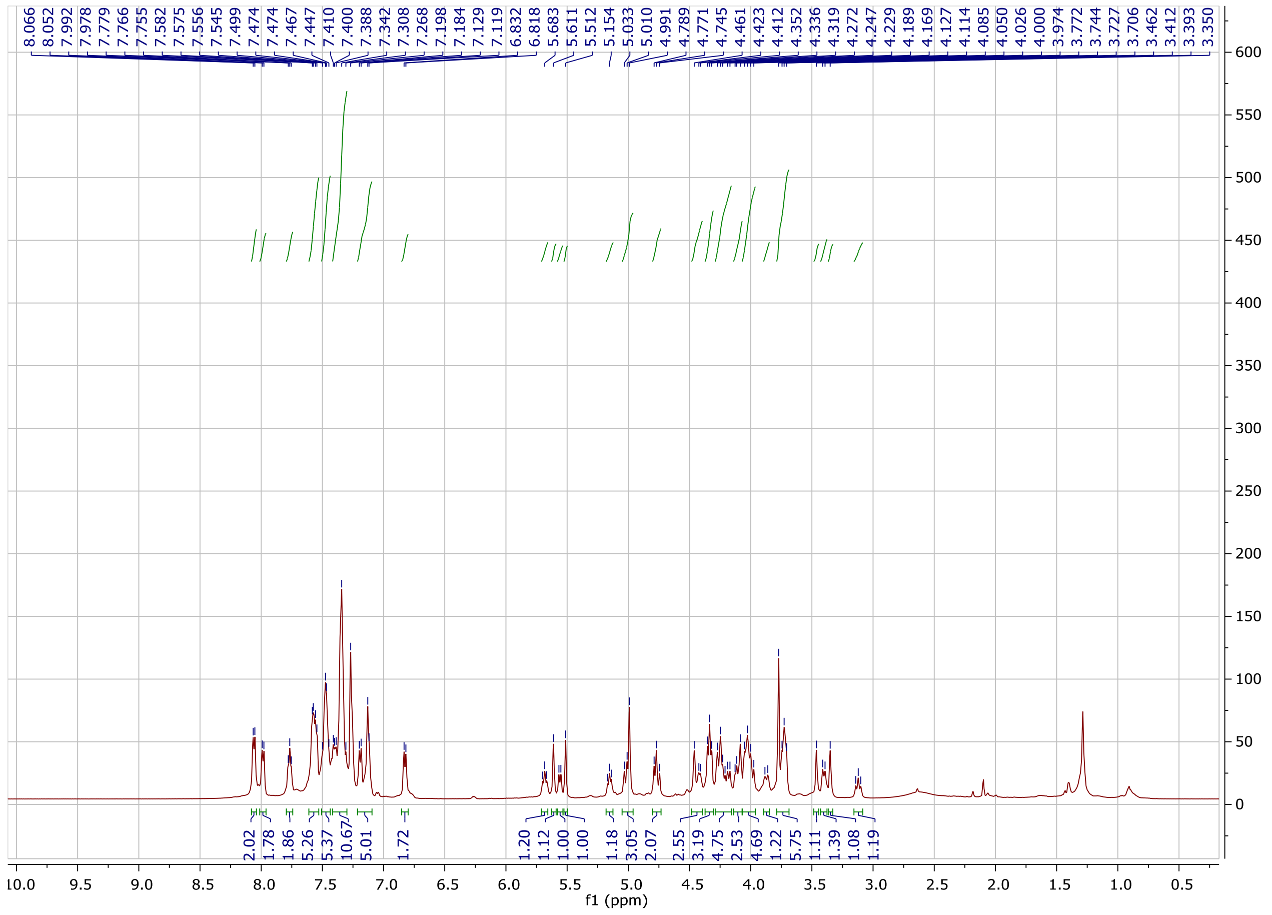

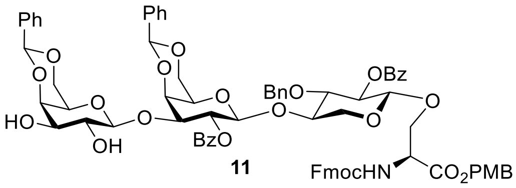

11

^{13}C-NMR ($CDCl_3$, 125 MHz) of **11**

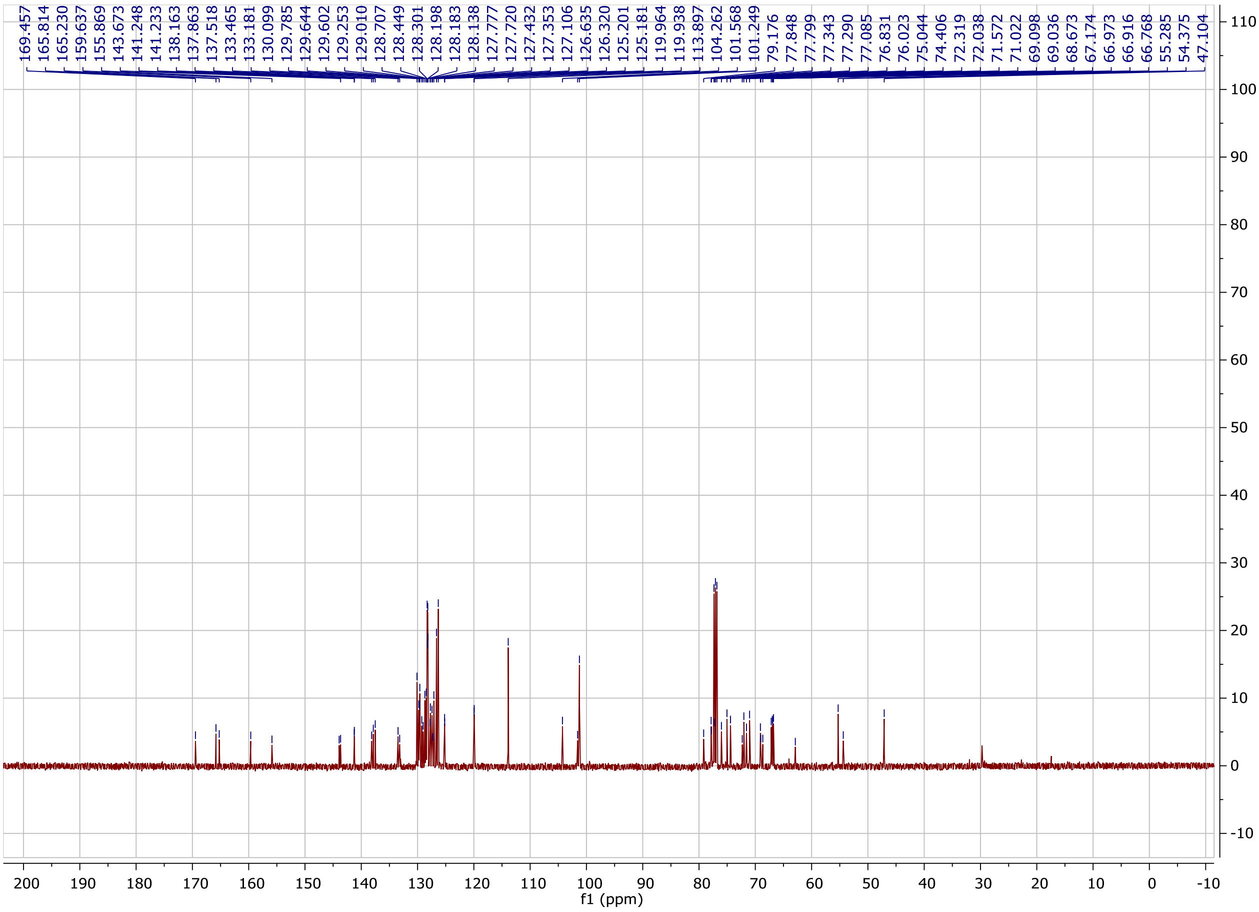

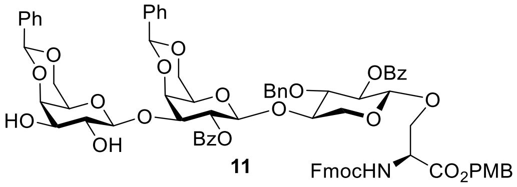

coupled gHSQC ($CDCl_3$, 500 MHz) of **11**

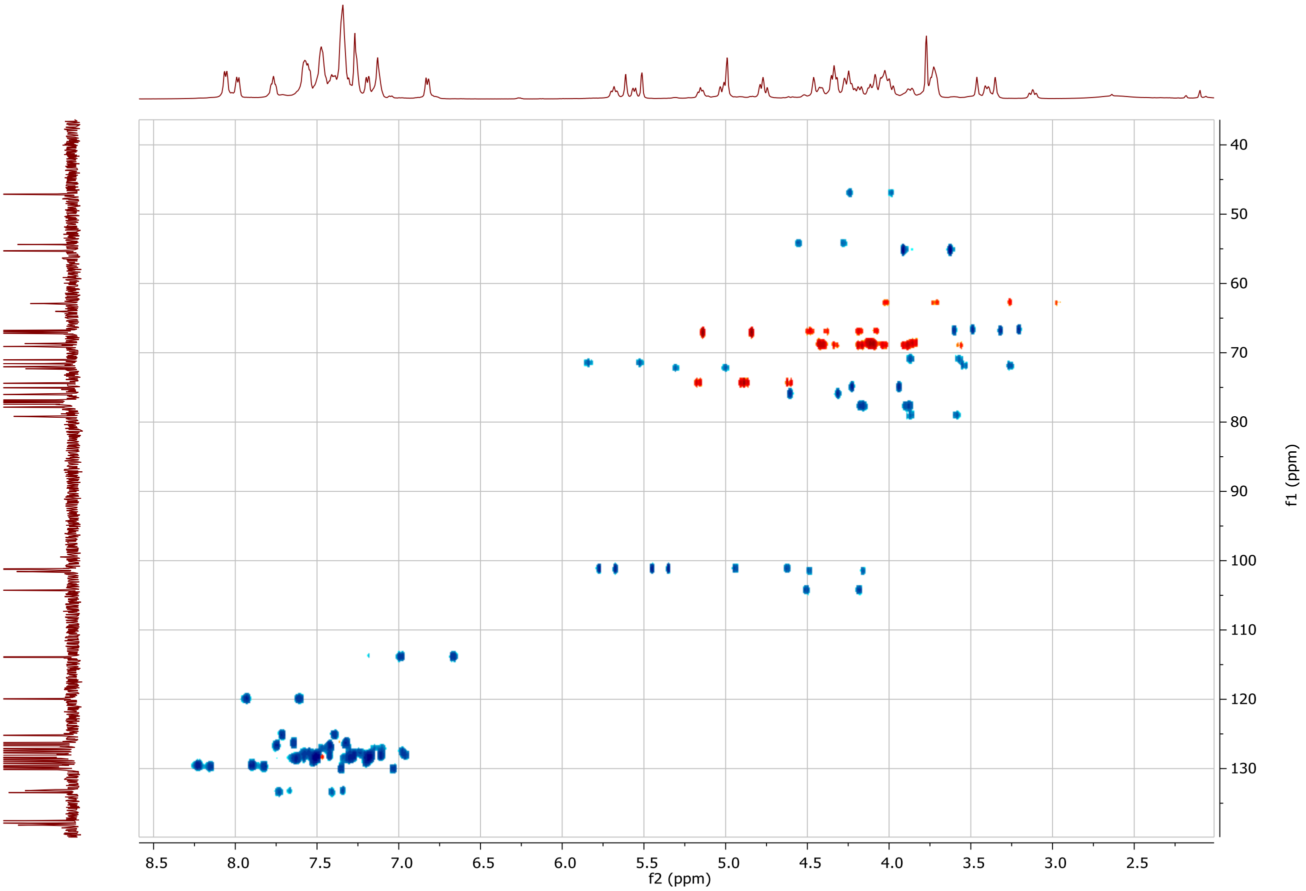

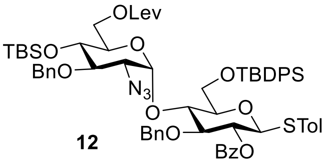

12

^{1}H-NMR ($CDCl_3$, 500 MHz) of **12**

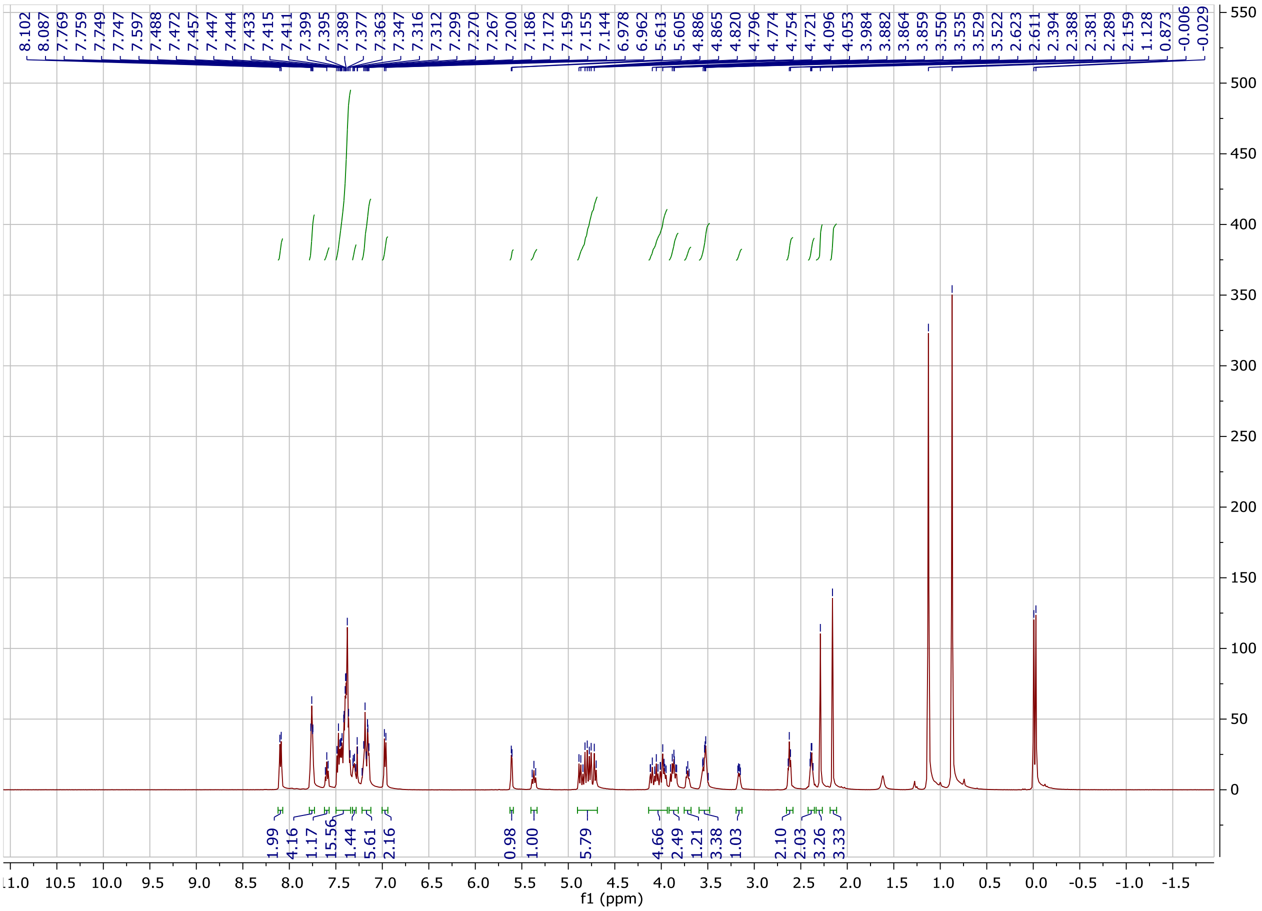

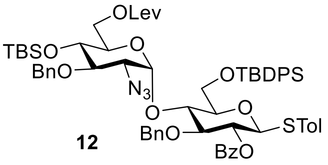

12

^{13}C-NMR ($CDCl_3$, 125 MHz) of **12**

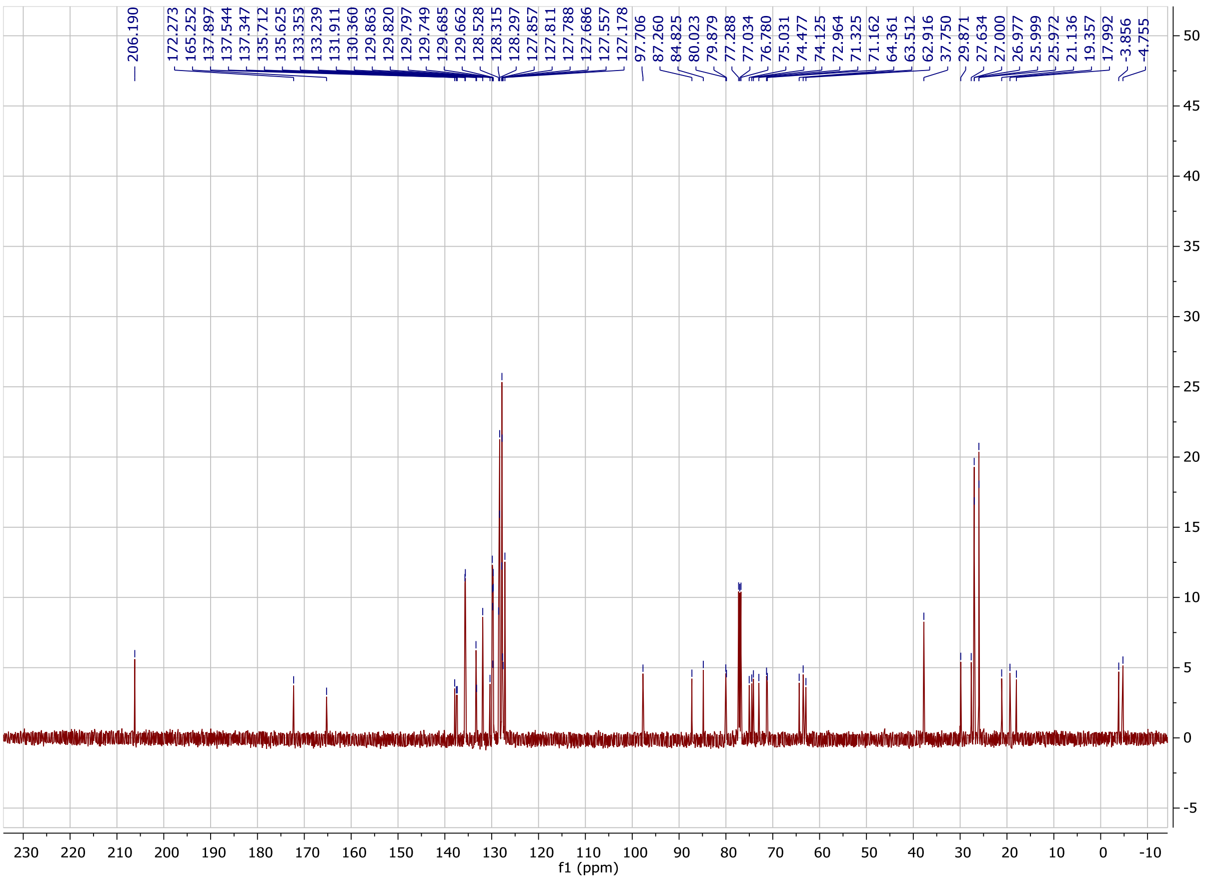

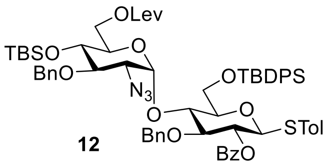

12

gHSQC ($CDCl_3$, 500 MHz) of **12**

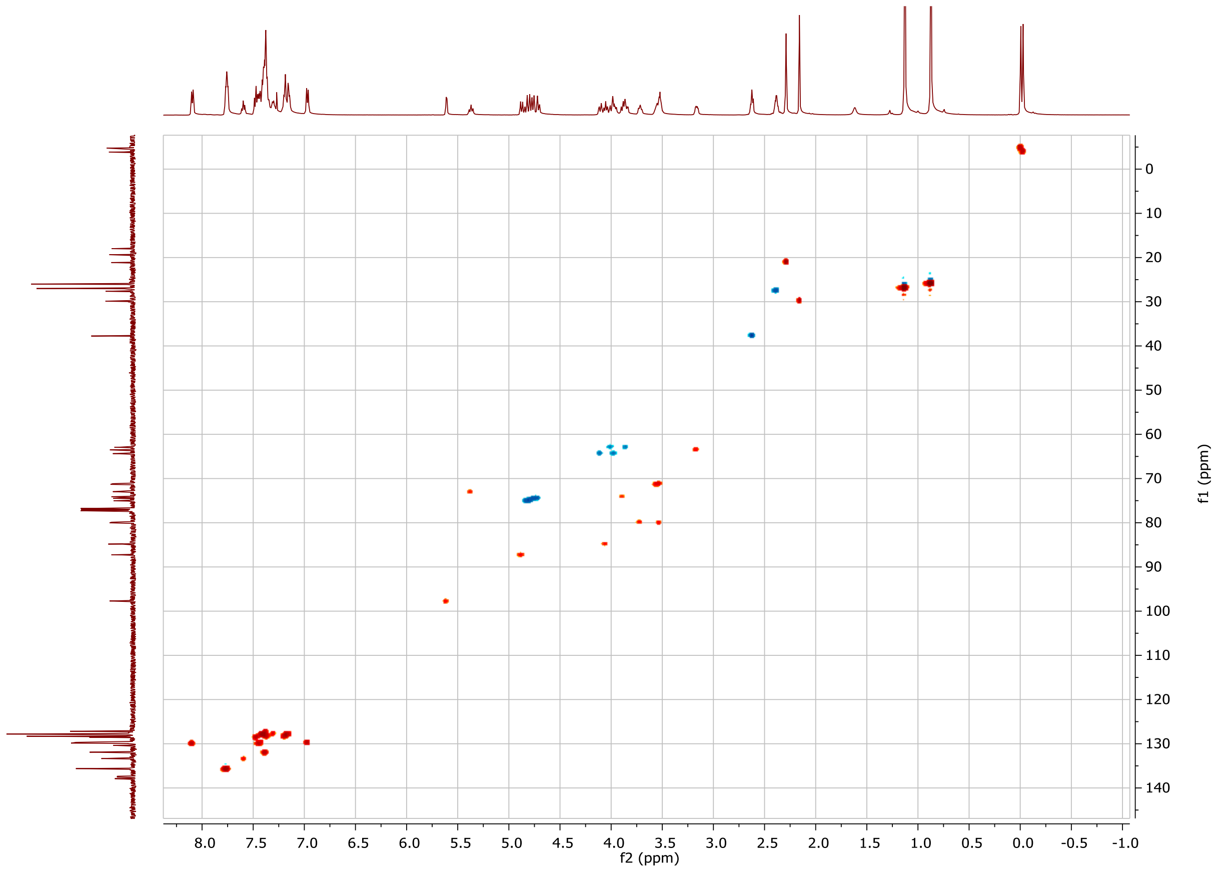

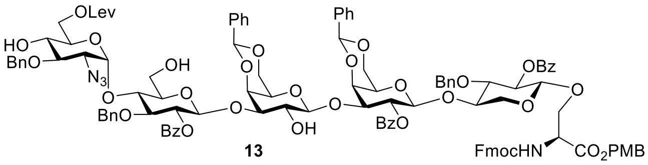

13

^{1}H-NMR ($CDCl_3$, 500 MHz) of **13**

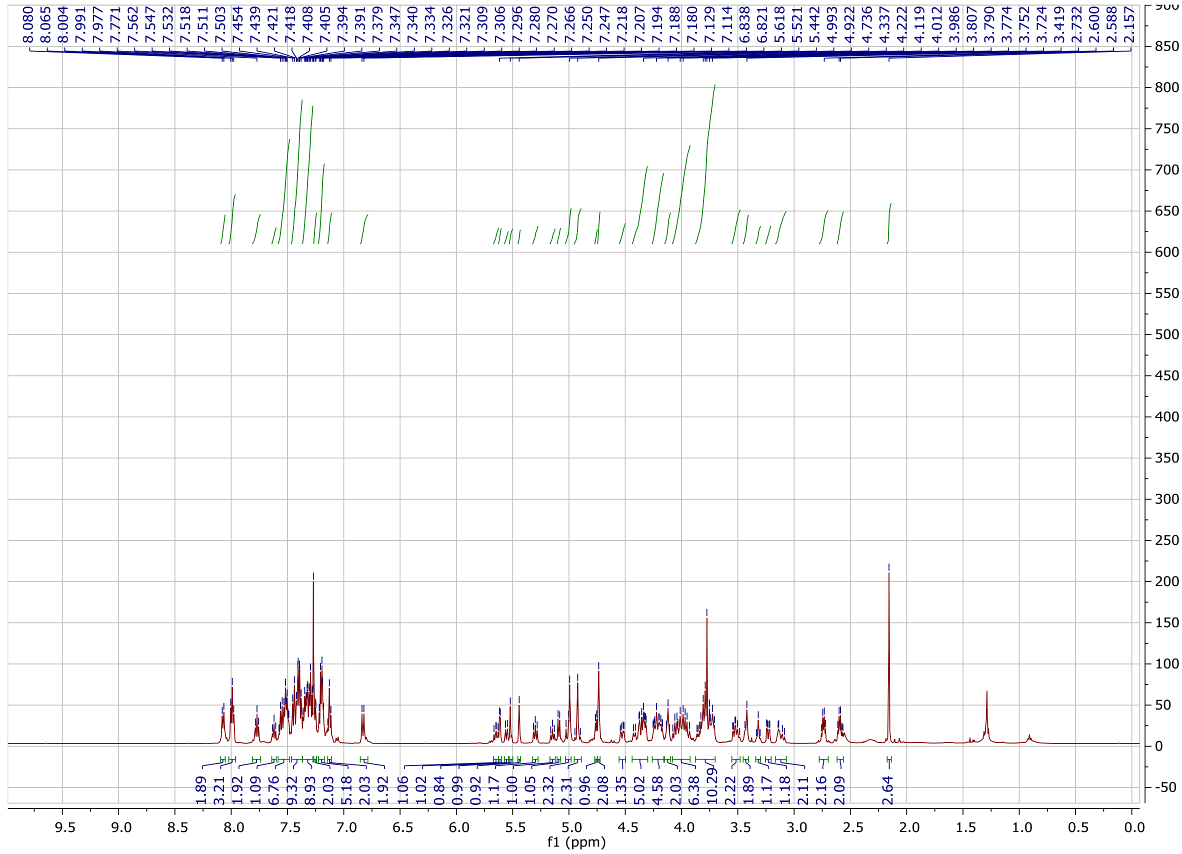

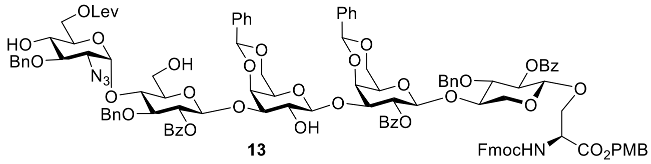

13

^{13}C-NMR ($CDCl_3$, 125 MHz) of **13**

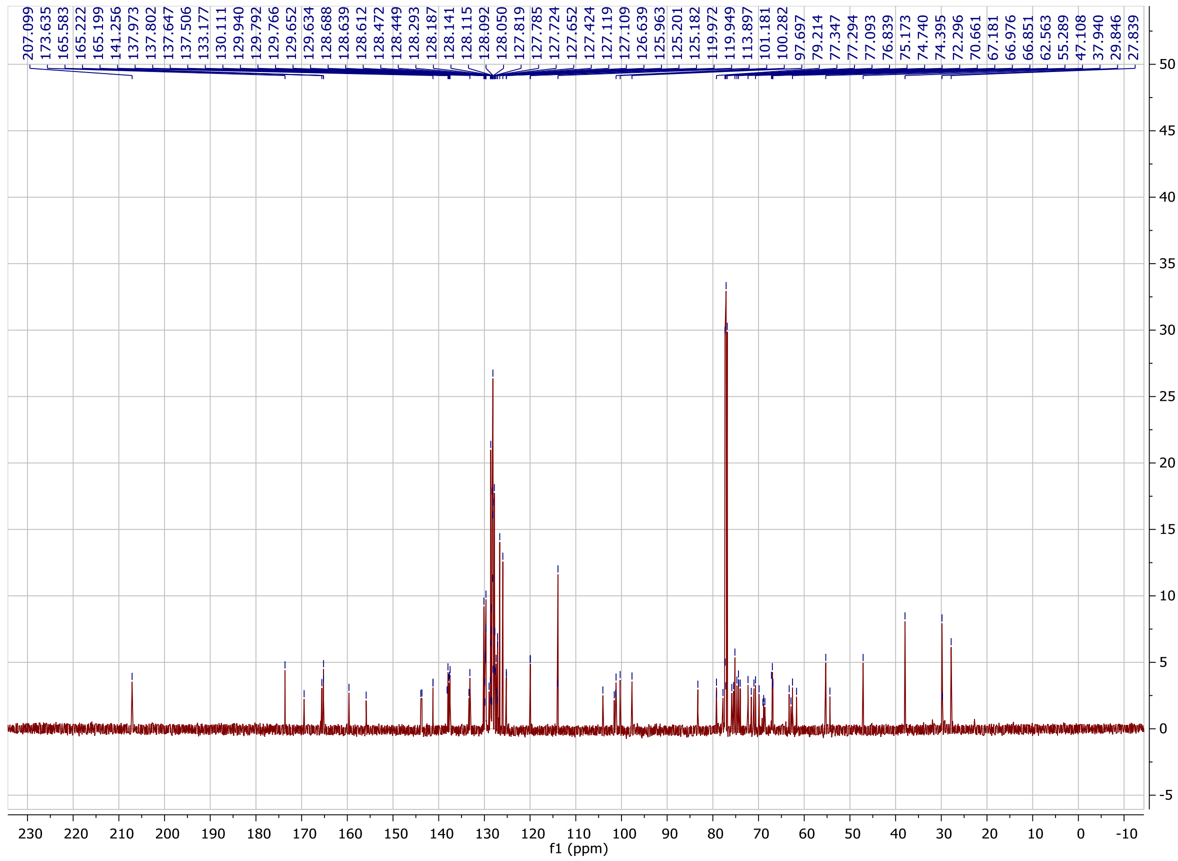

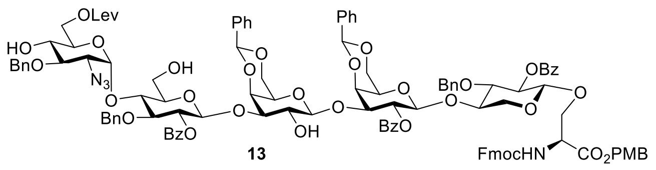

coupled gHSQC ($CDCl_3$, 500 MHz) of **13**

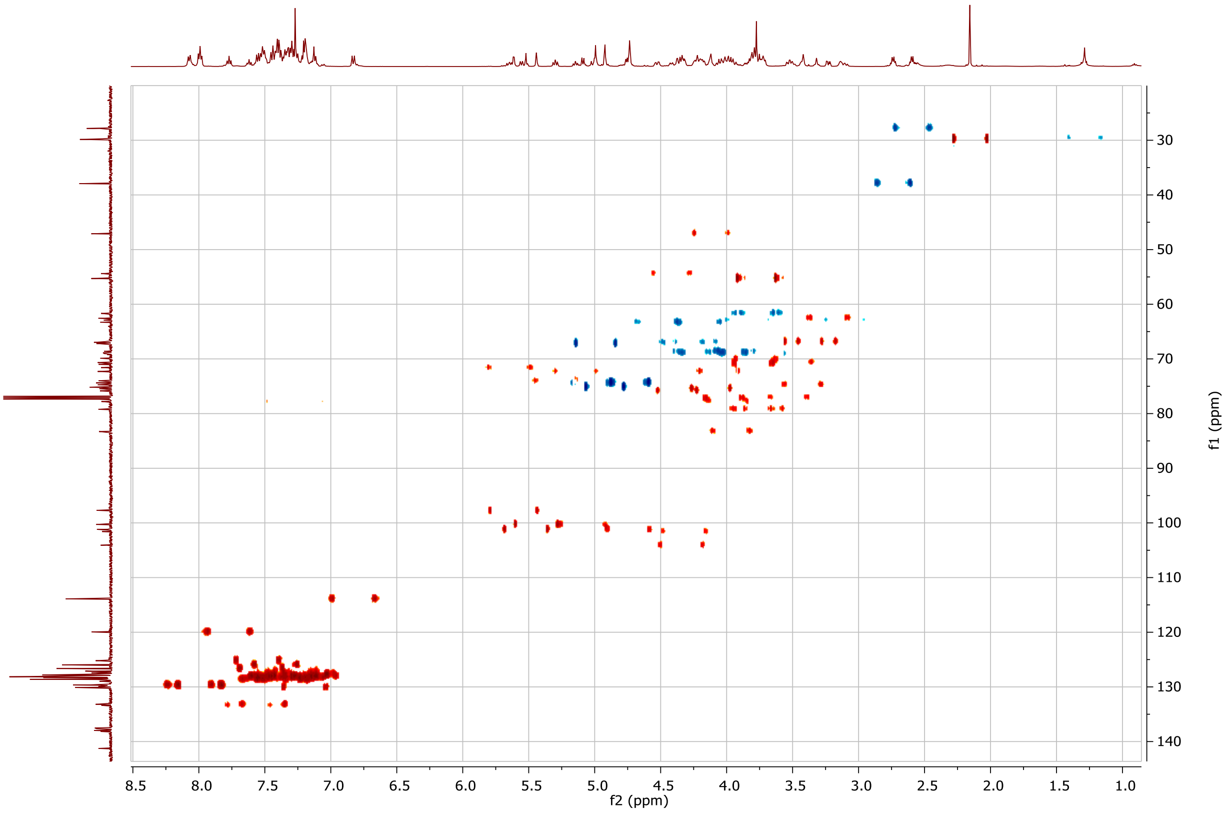

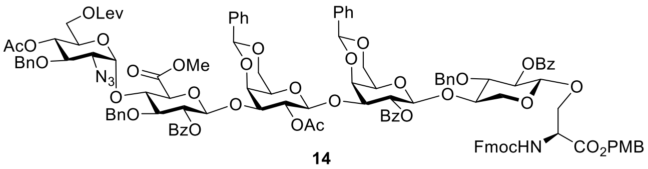

14

^{1}H-NMR ($CDCl_3$, 500 MHz) of **14**

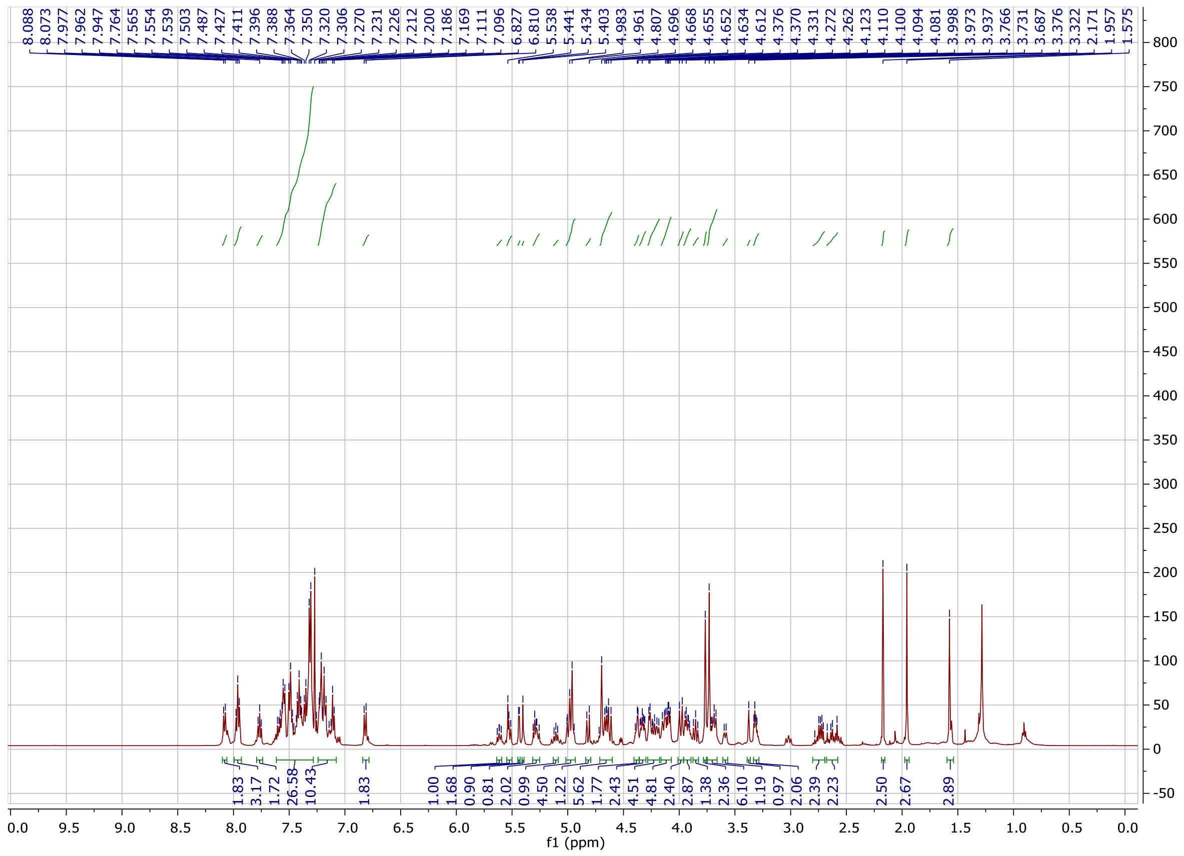

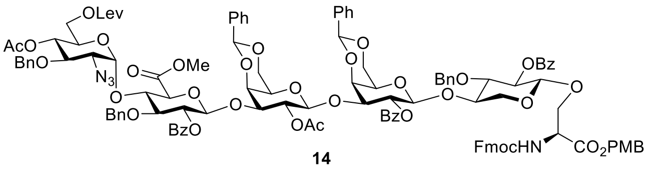

14

^{13}C-NMR ($CDCl_3$, 125 MHz) of **14**

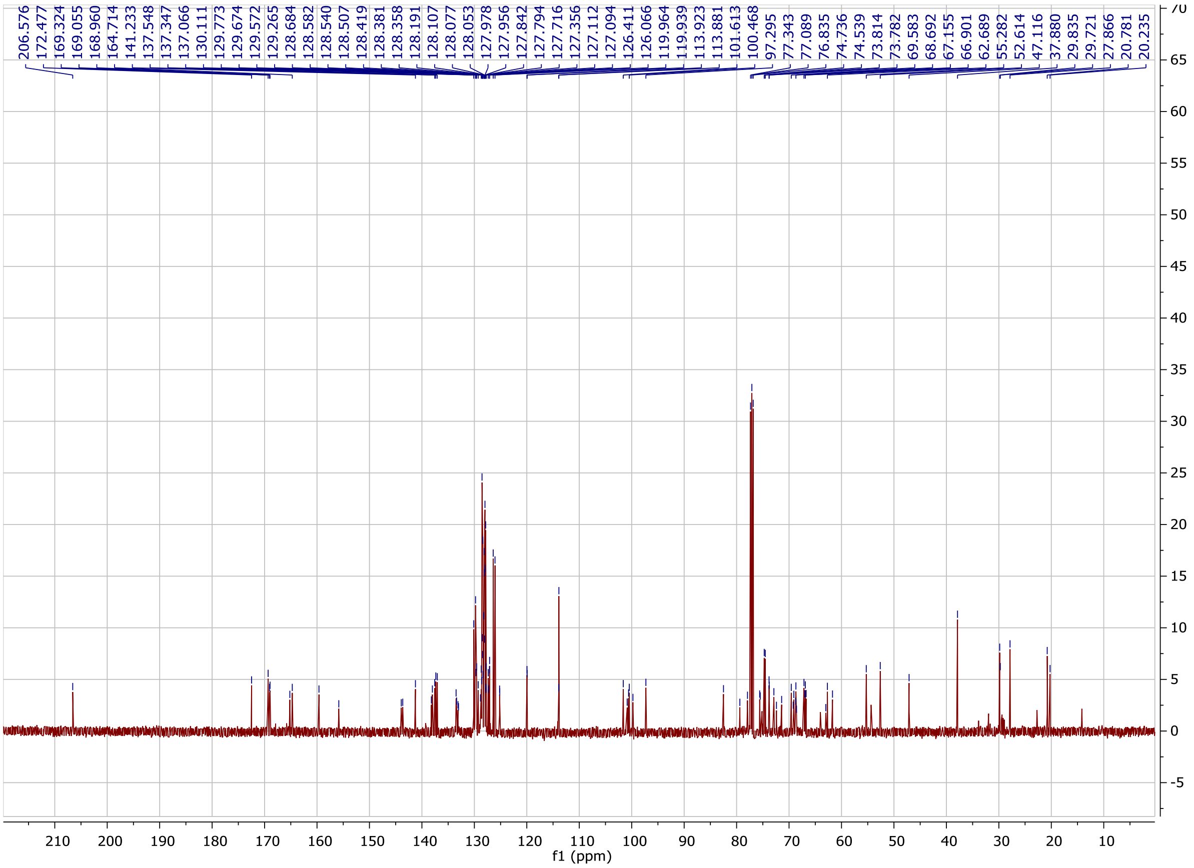

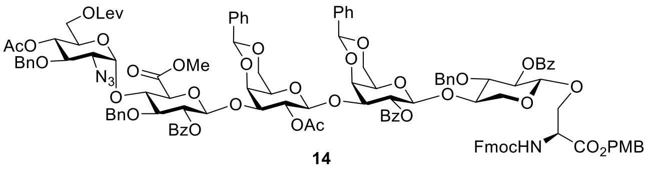

14

gHSQC ($CDCl_3$, 500 MHz) of **14**

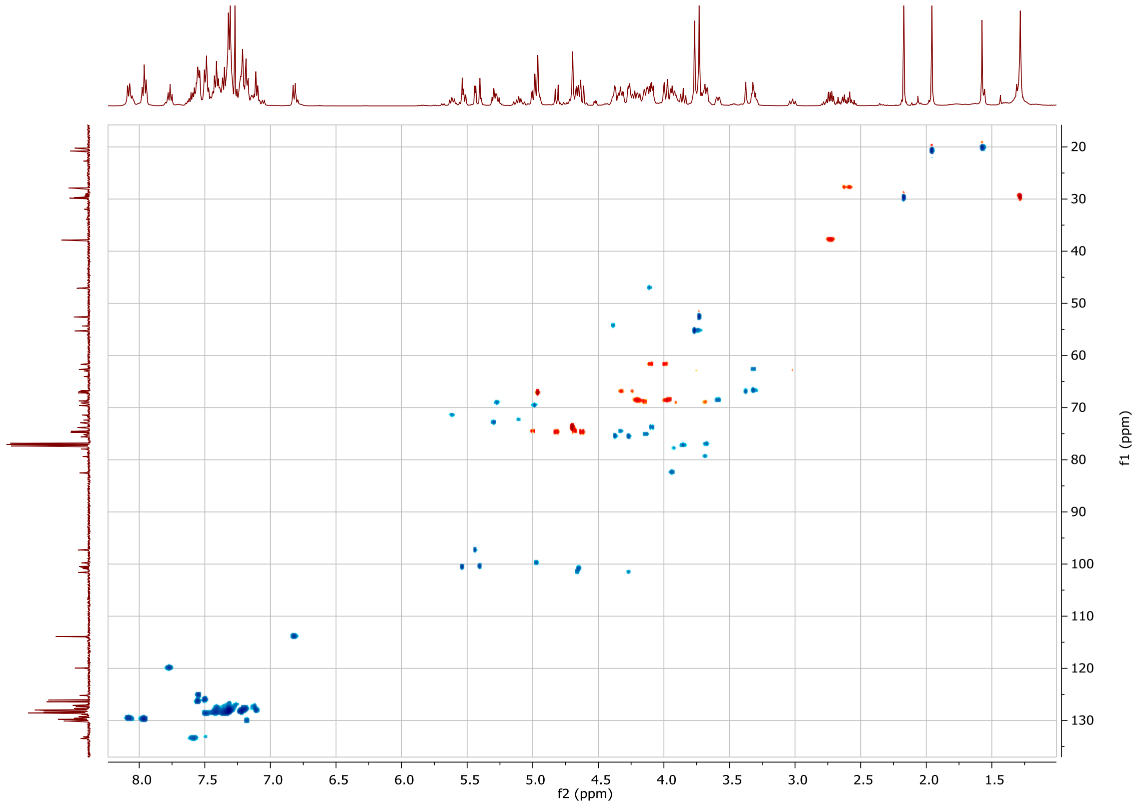

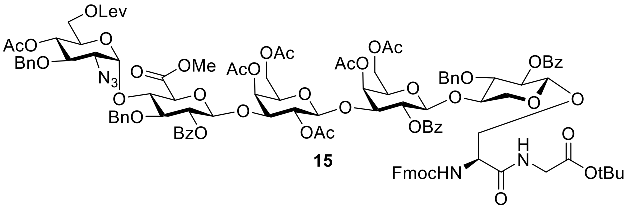

15

^{1}H-NMR ($CDCl_3$, 500 MHz) of **15**

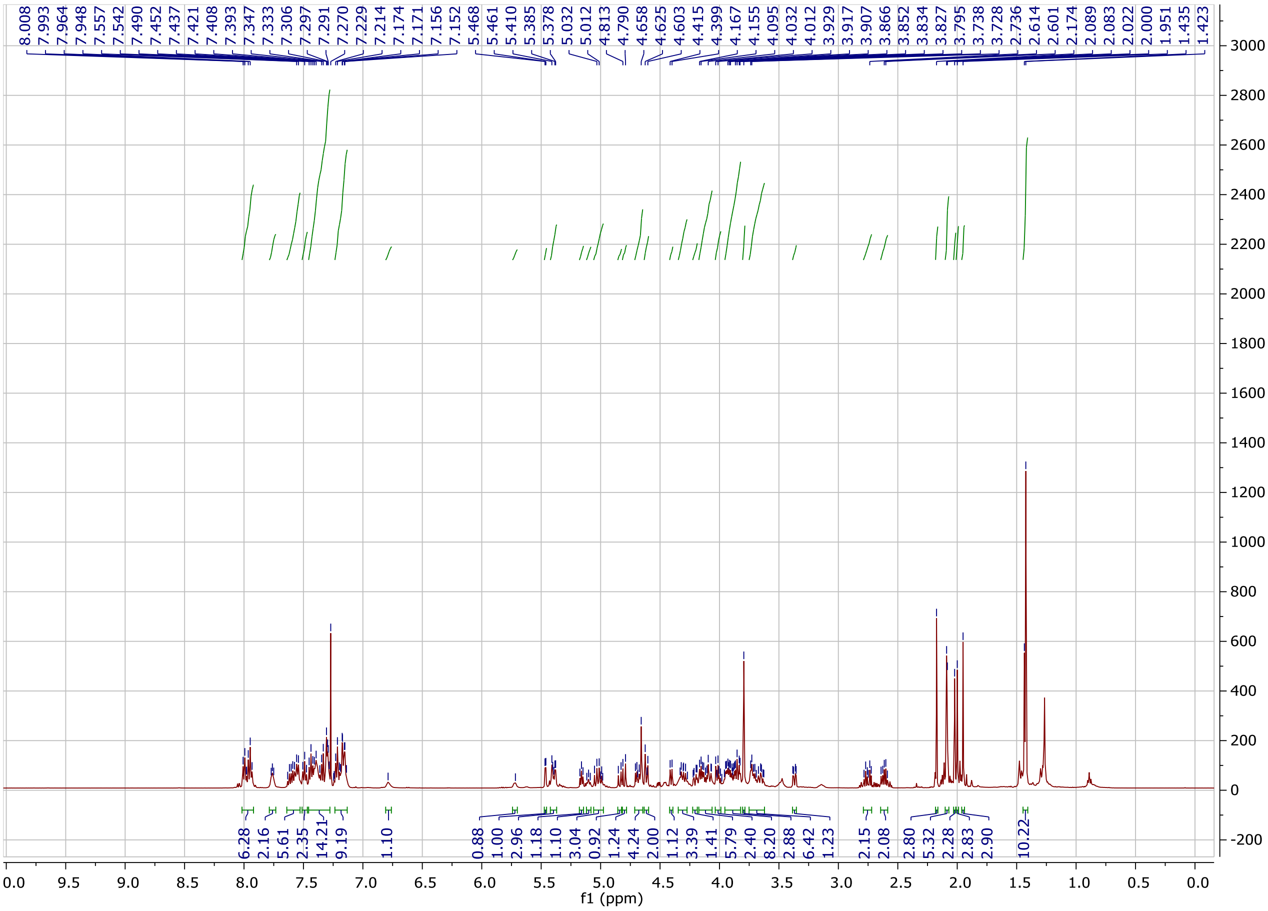

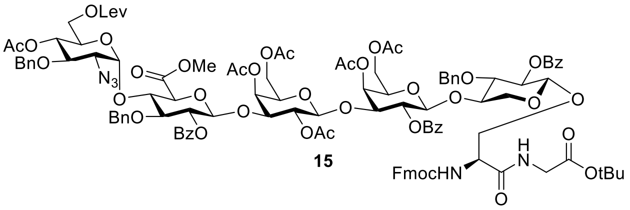

15

^{13}C-NMR ($CDCl_3$, 125 MHz) of **15**

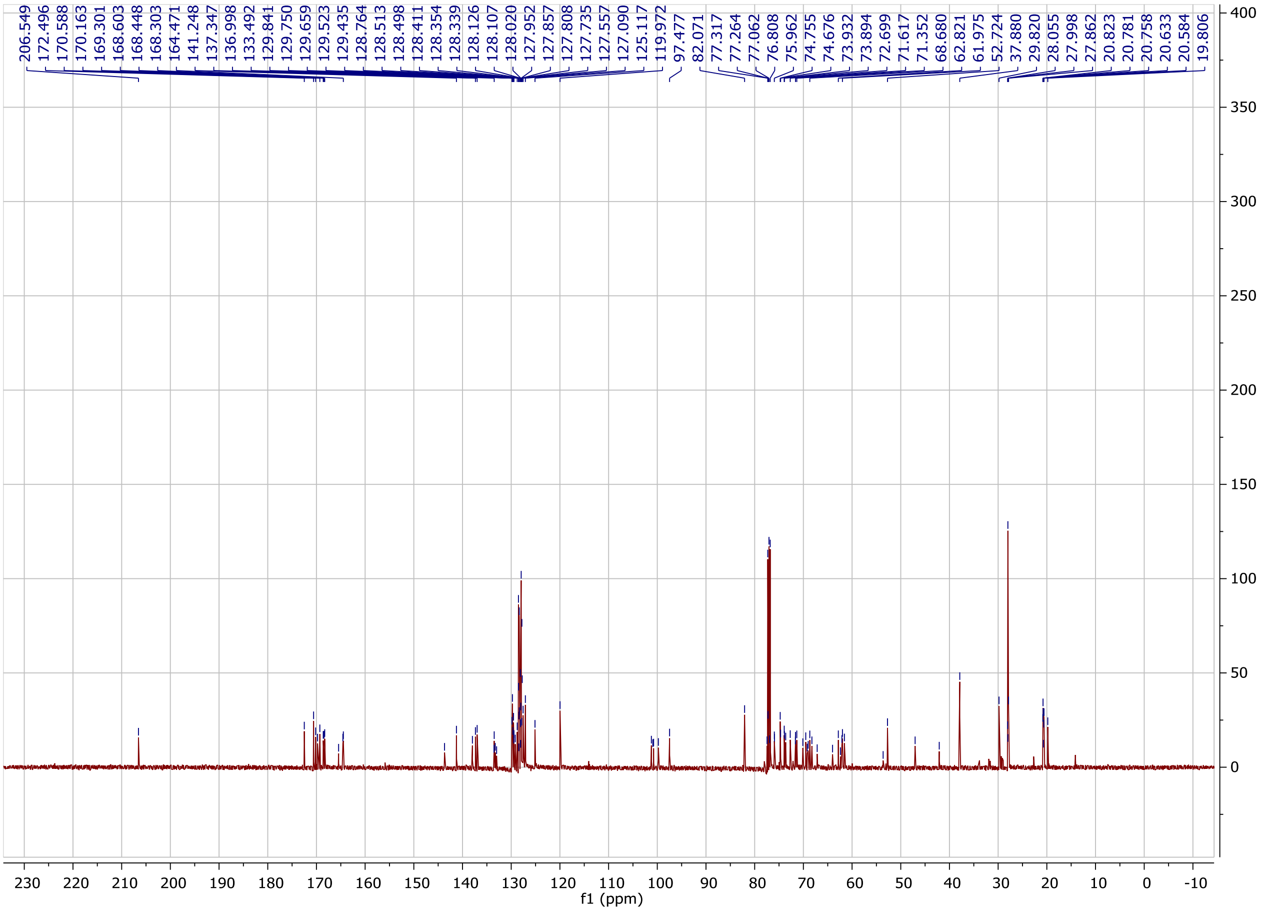

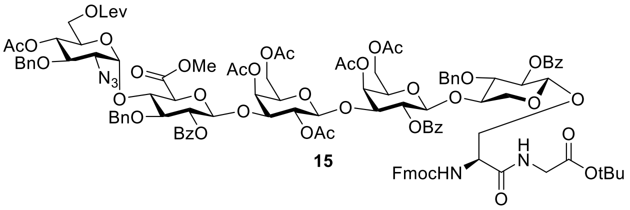

15

gHSQC ($CDCl_3$, 500 MHz) of **15**

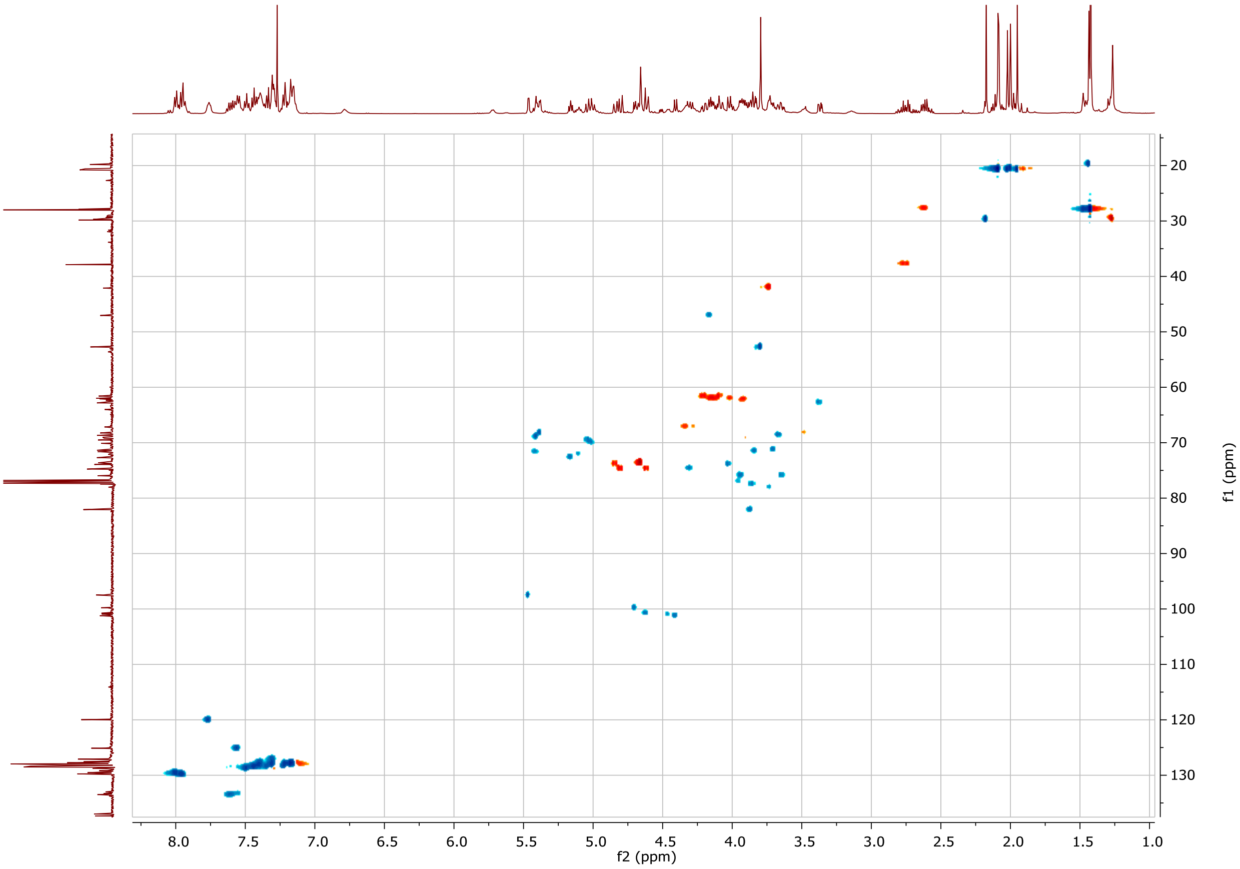

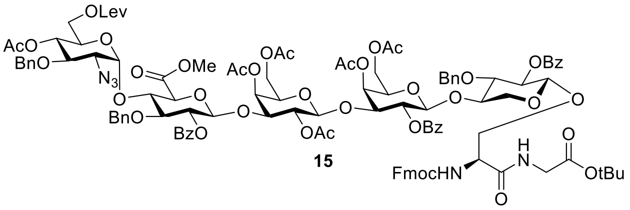

gCOSY ($CDCl_3$, 500 MHz) of **15**

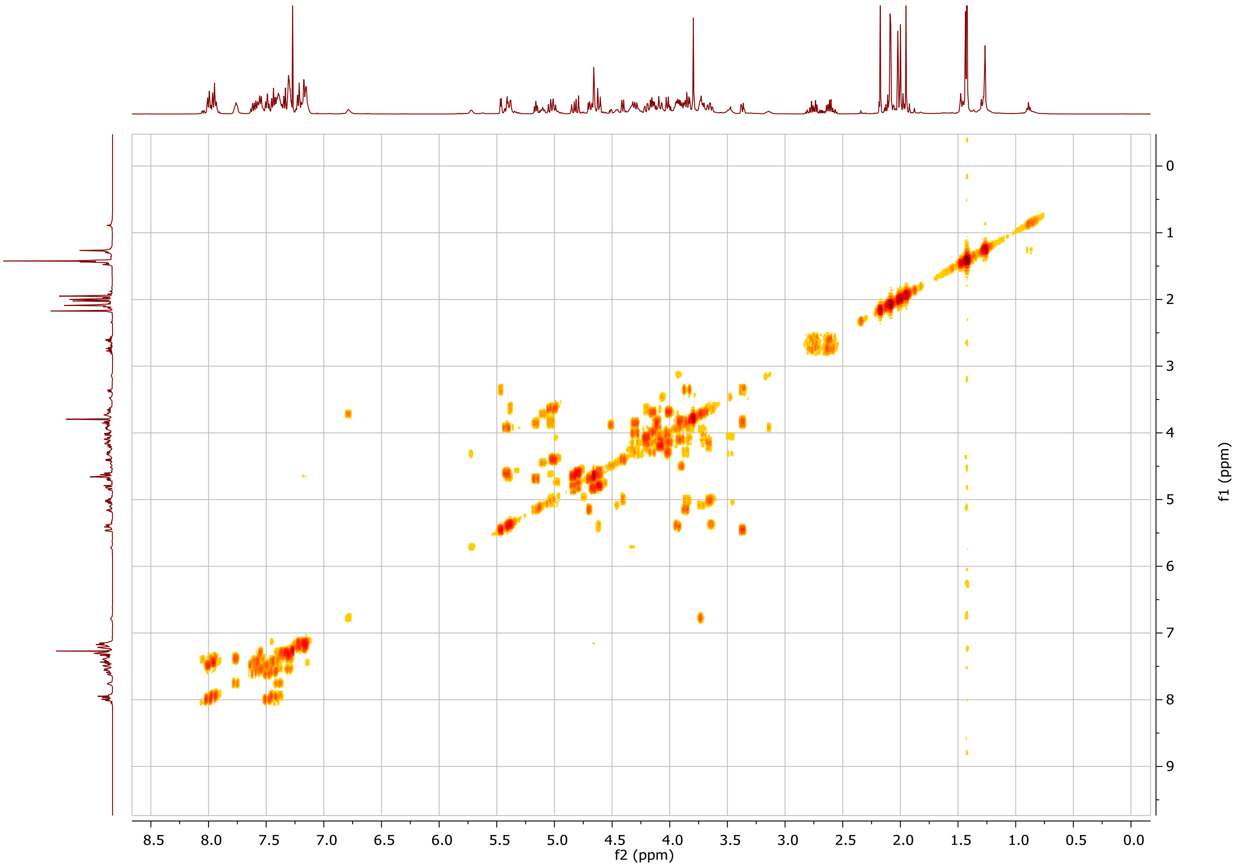

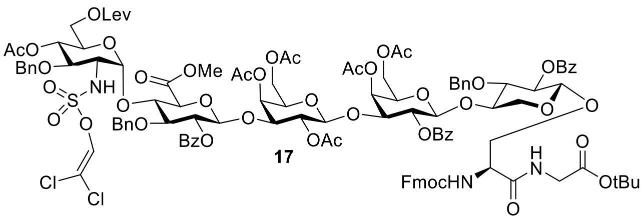

^{1}H-NMR ($CDCl_3$, 500 MHz) of **17**

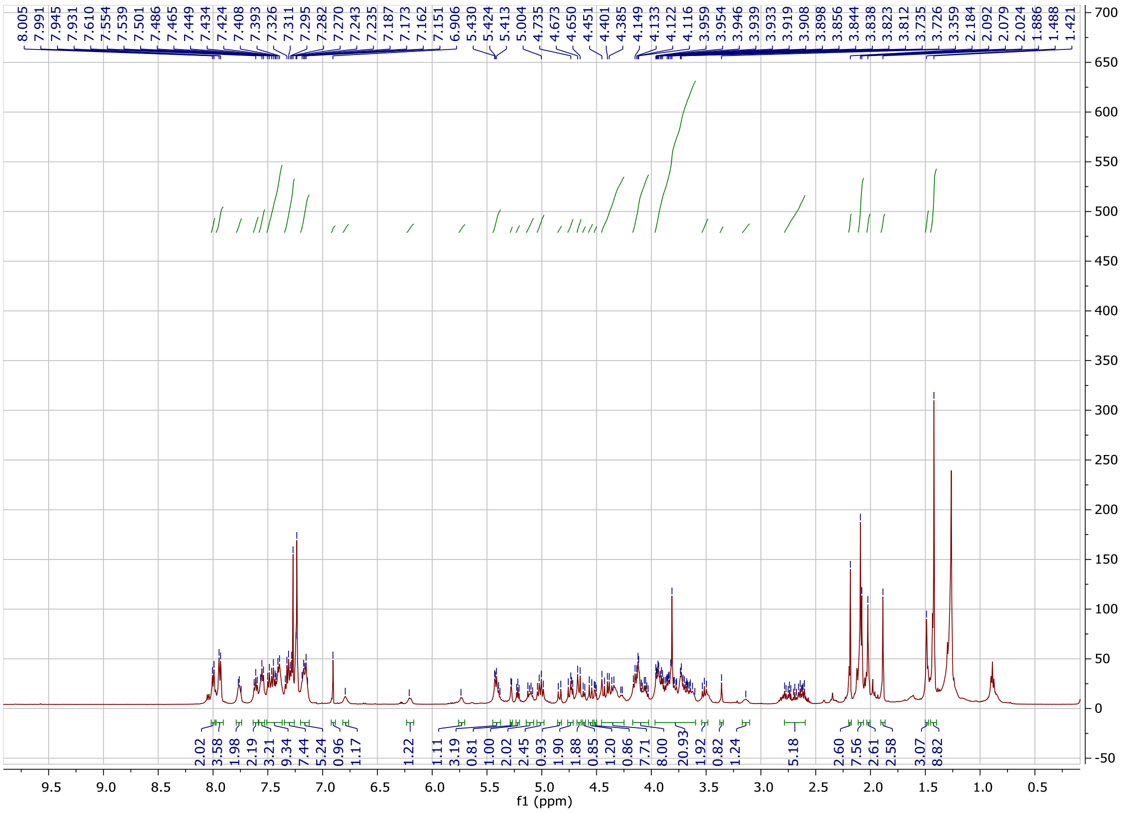

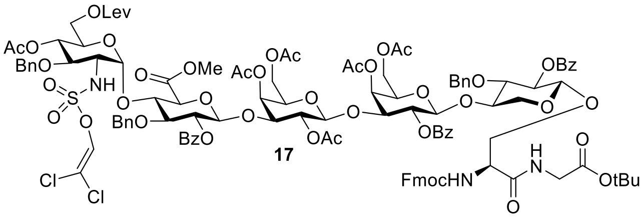

^{13}C-NMR ($CDCl_3$, 125 MHz) of **17**

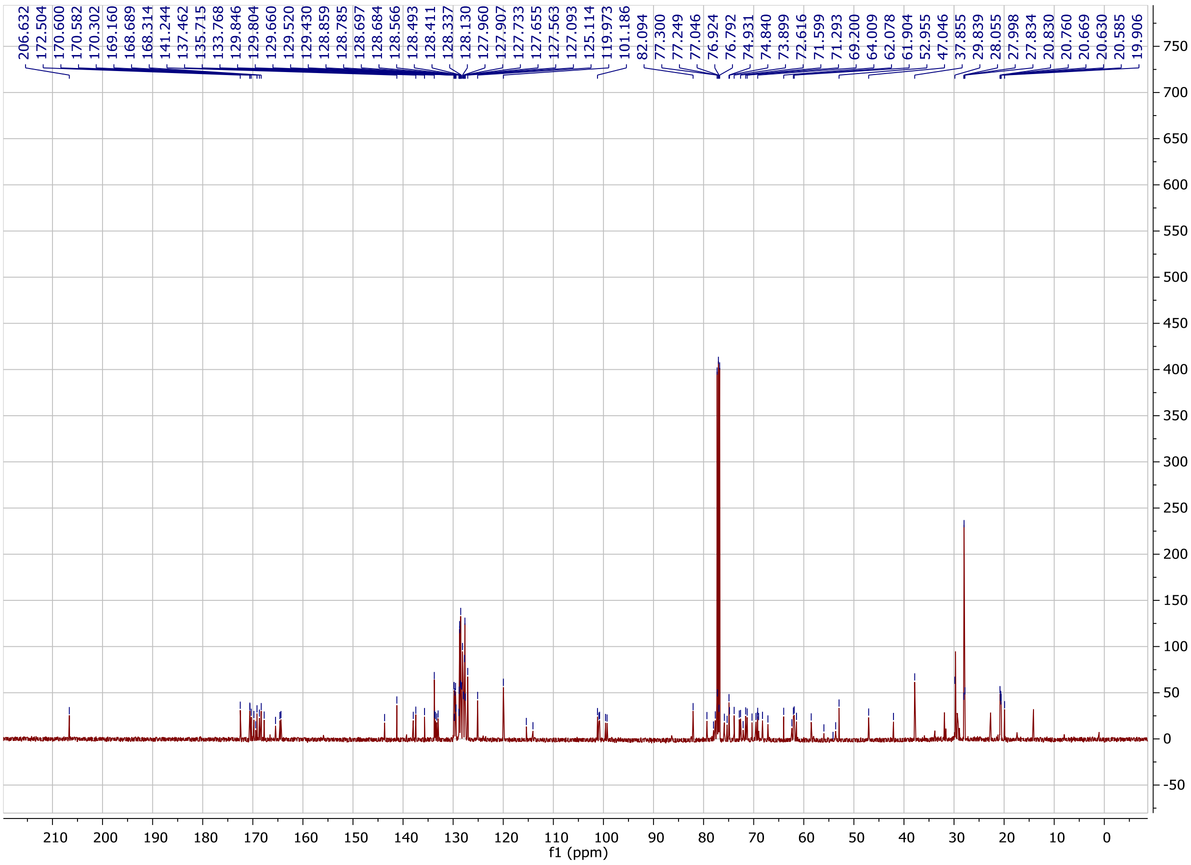

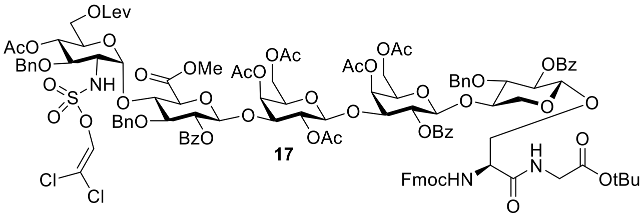

gHSQC ($CDCl_3$, 500 MHz) of **17**

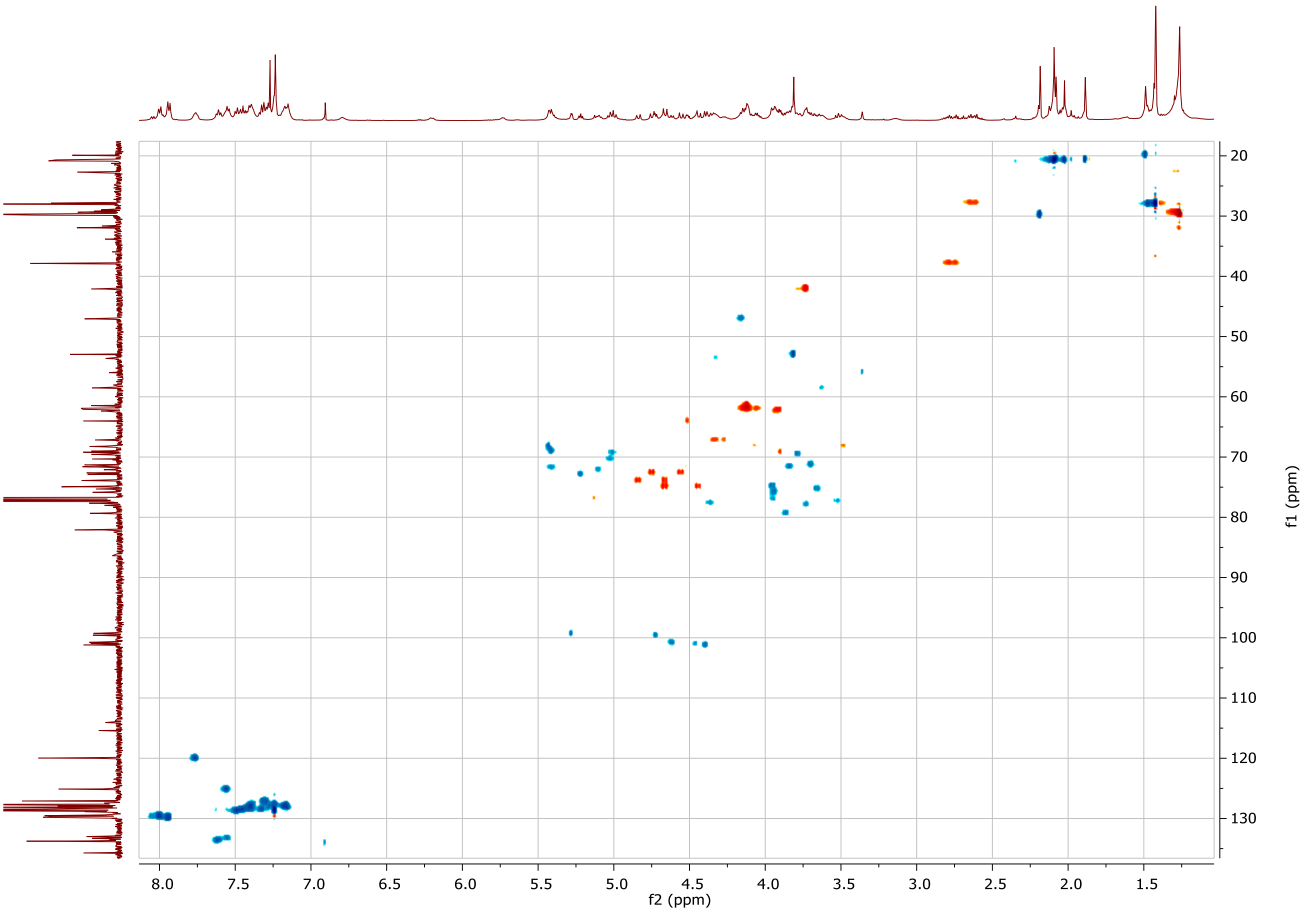

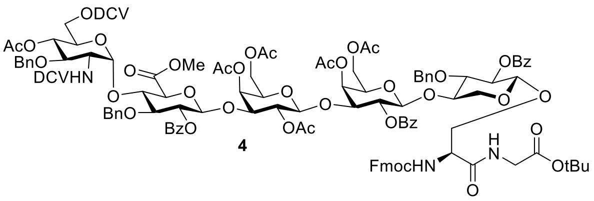

^{1}H-NMR ($CDCl_3$, 500 MHz) of **4**

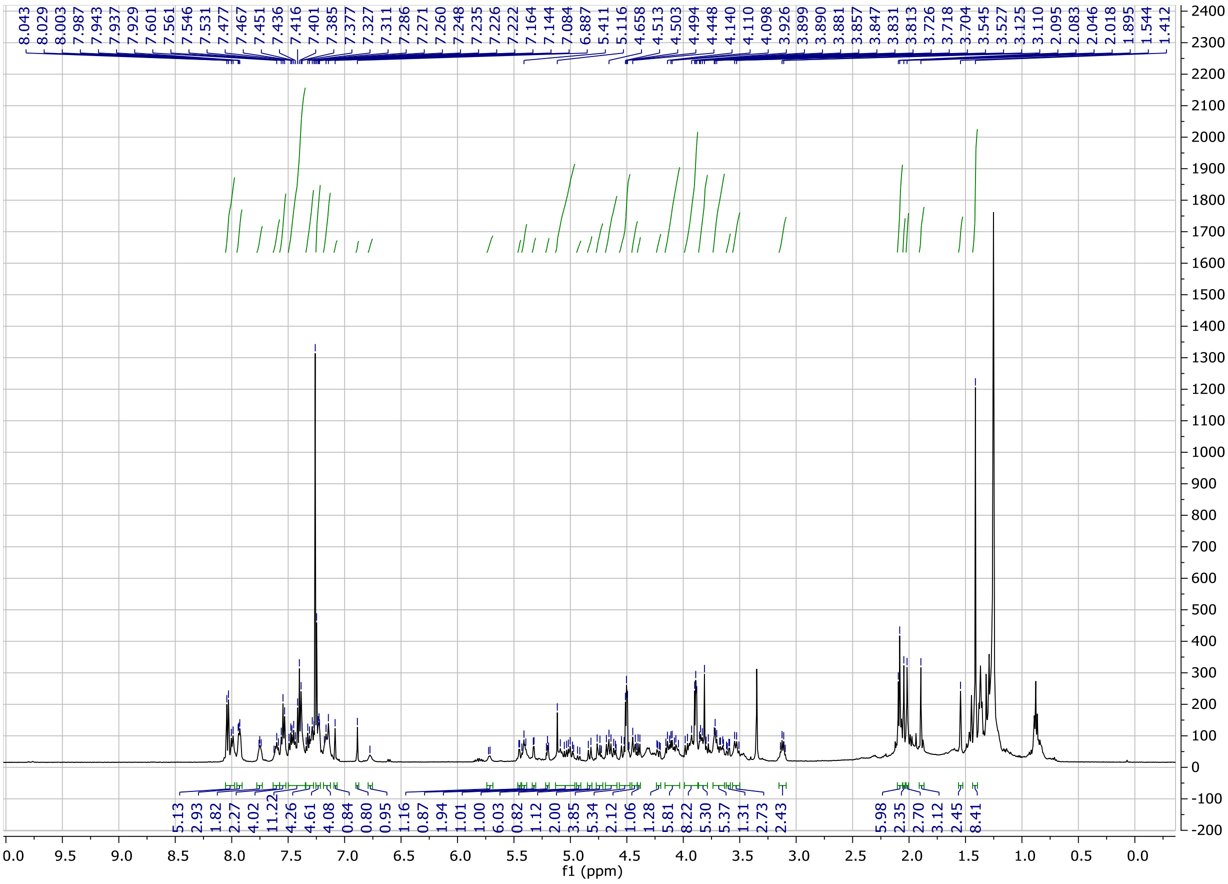

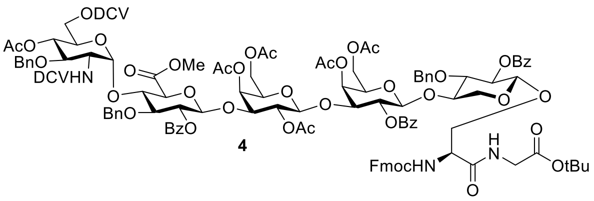

4

^{13}C-NMR ($CDCl_3$, 125 MHz) of **4**

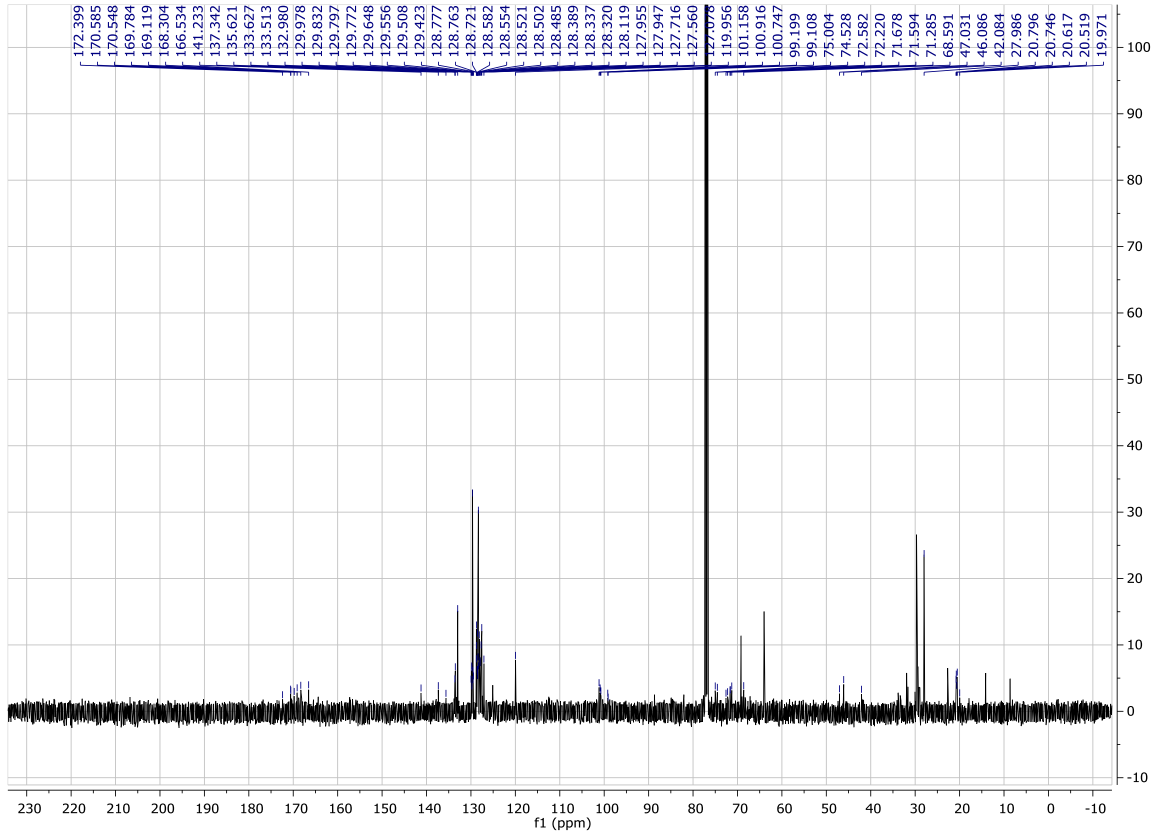

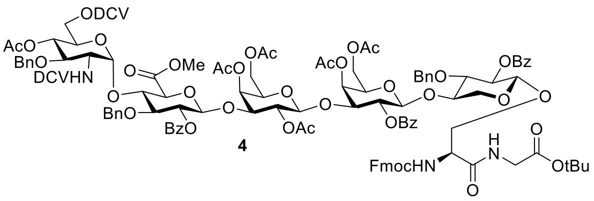

4

gHSQC ($CDCl_3$, 500 MHz) of **4**

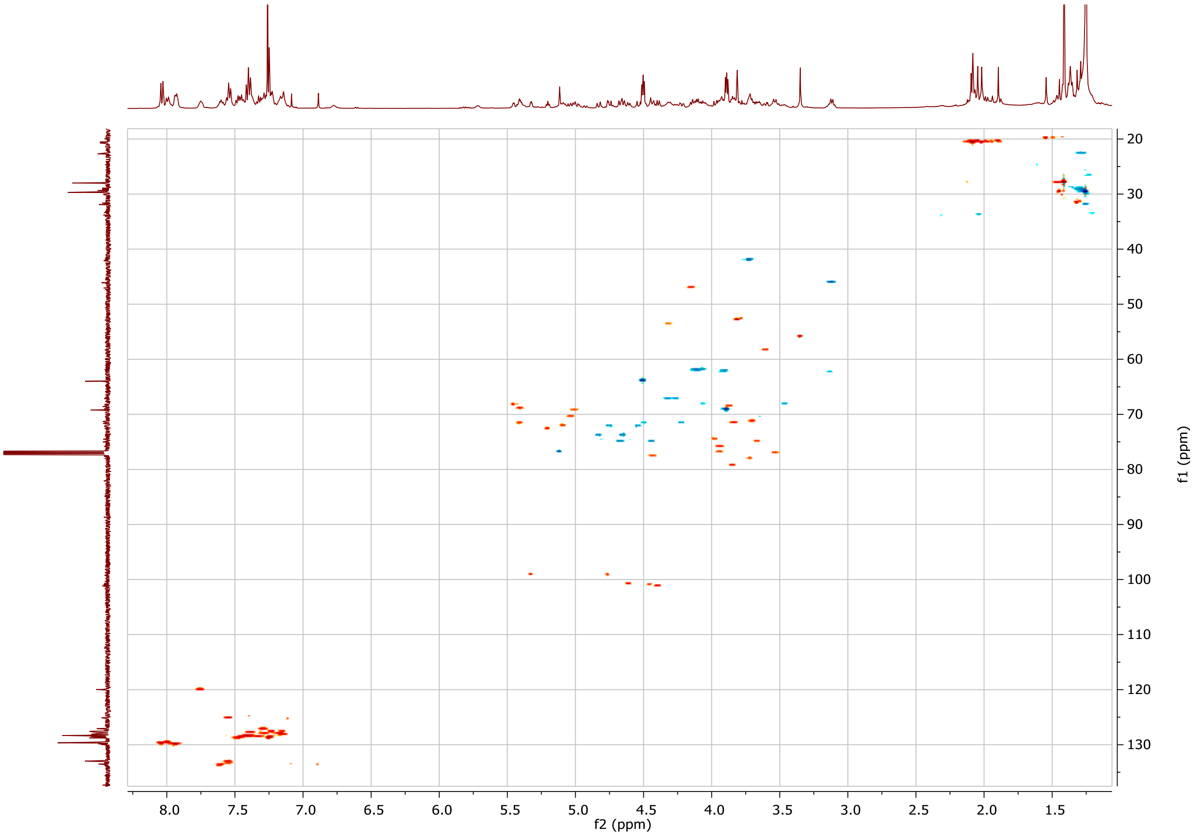

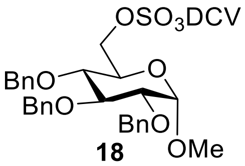

18

^{1}H-NMR ($CDCl_3$, 500 MHz) of **18**

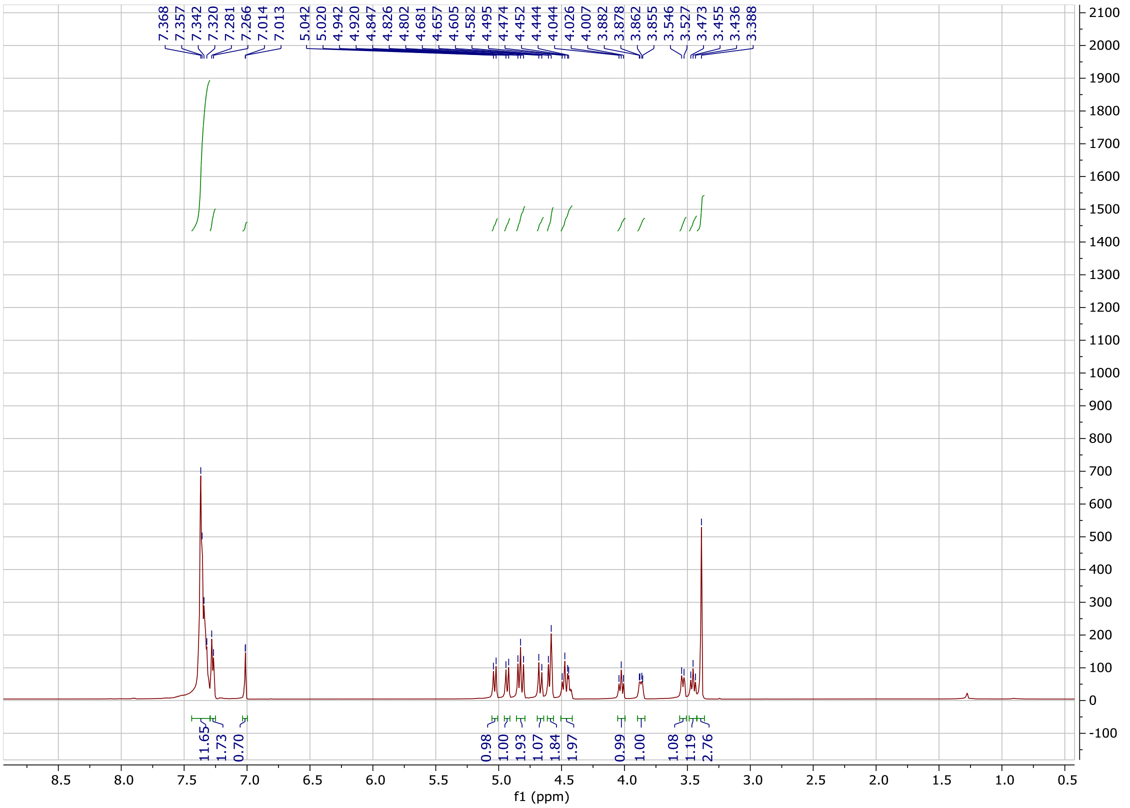

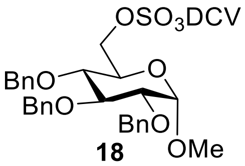

18

^{13}C-NMR ($CDCl_3$, 125 MHz) of **18**

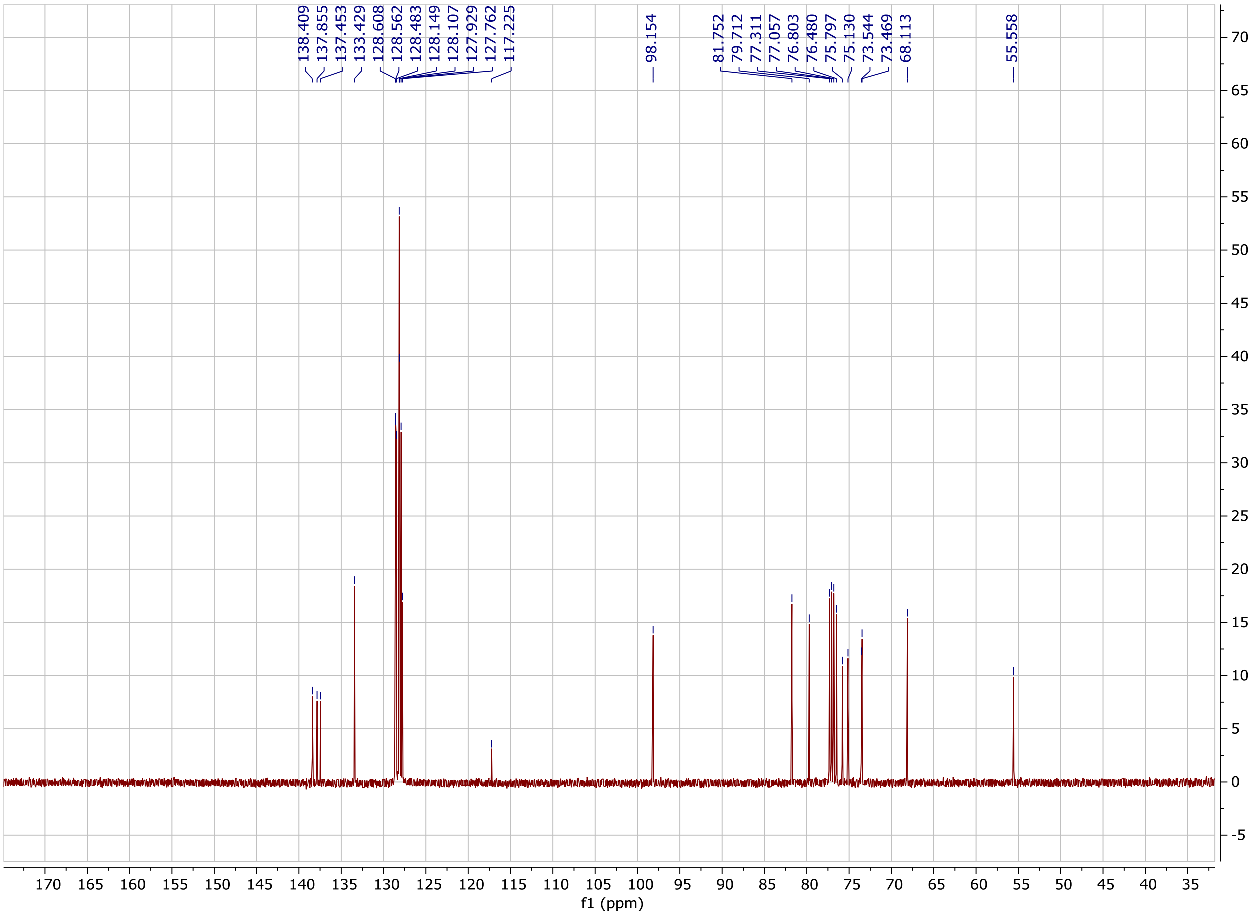

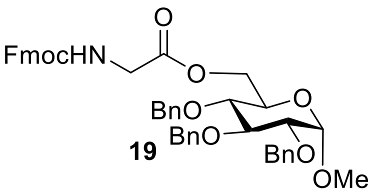

19

^{1}H-NMR ($CDCl_3$, 500 MHz) of **19**

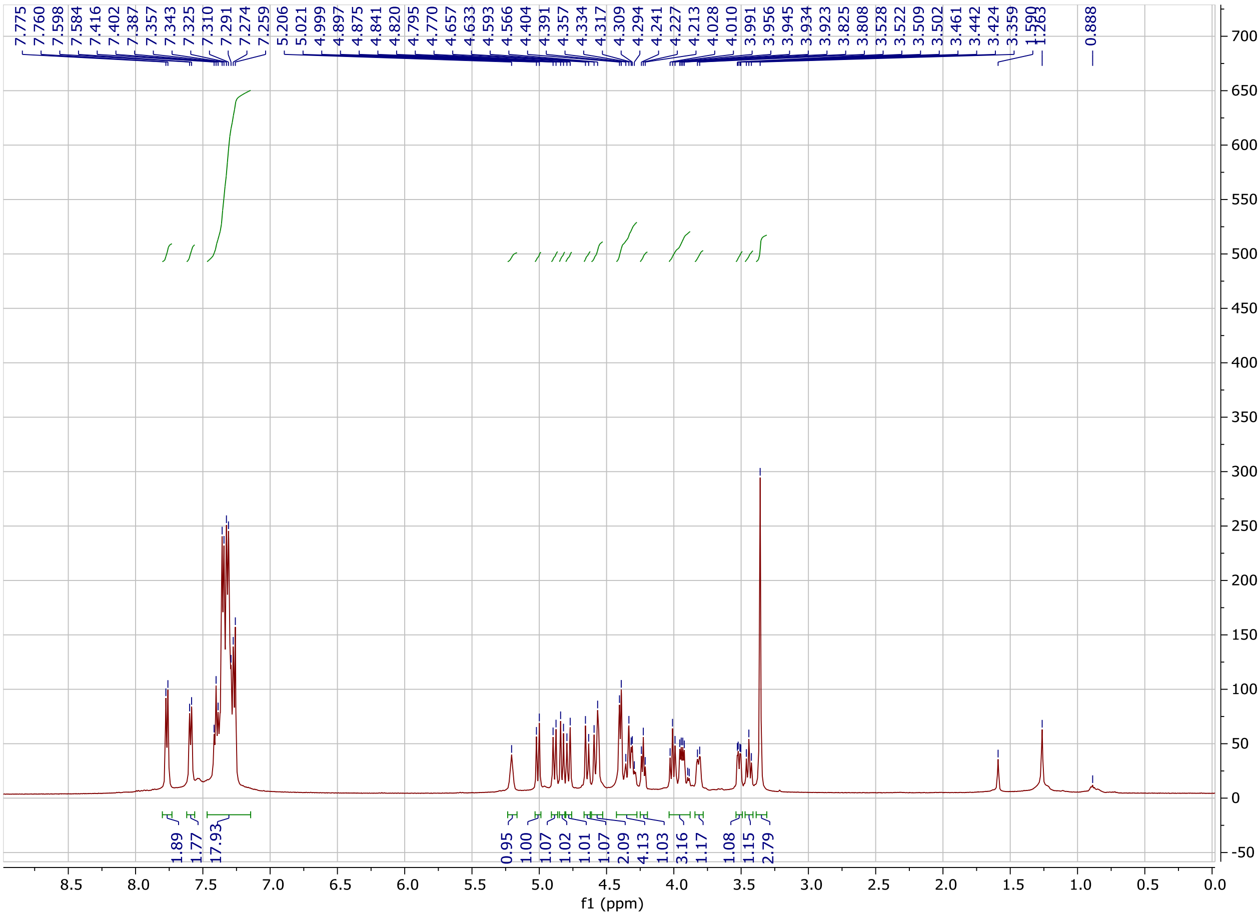

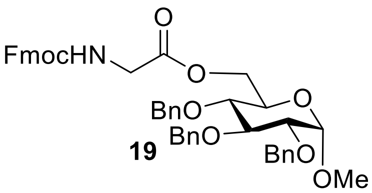

19

^{13}C-NMR ($CDCl_3$, 125 MHz) of **19**

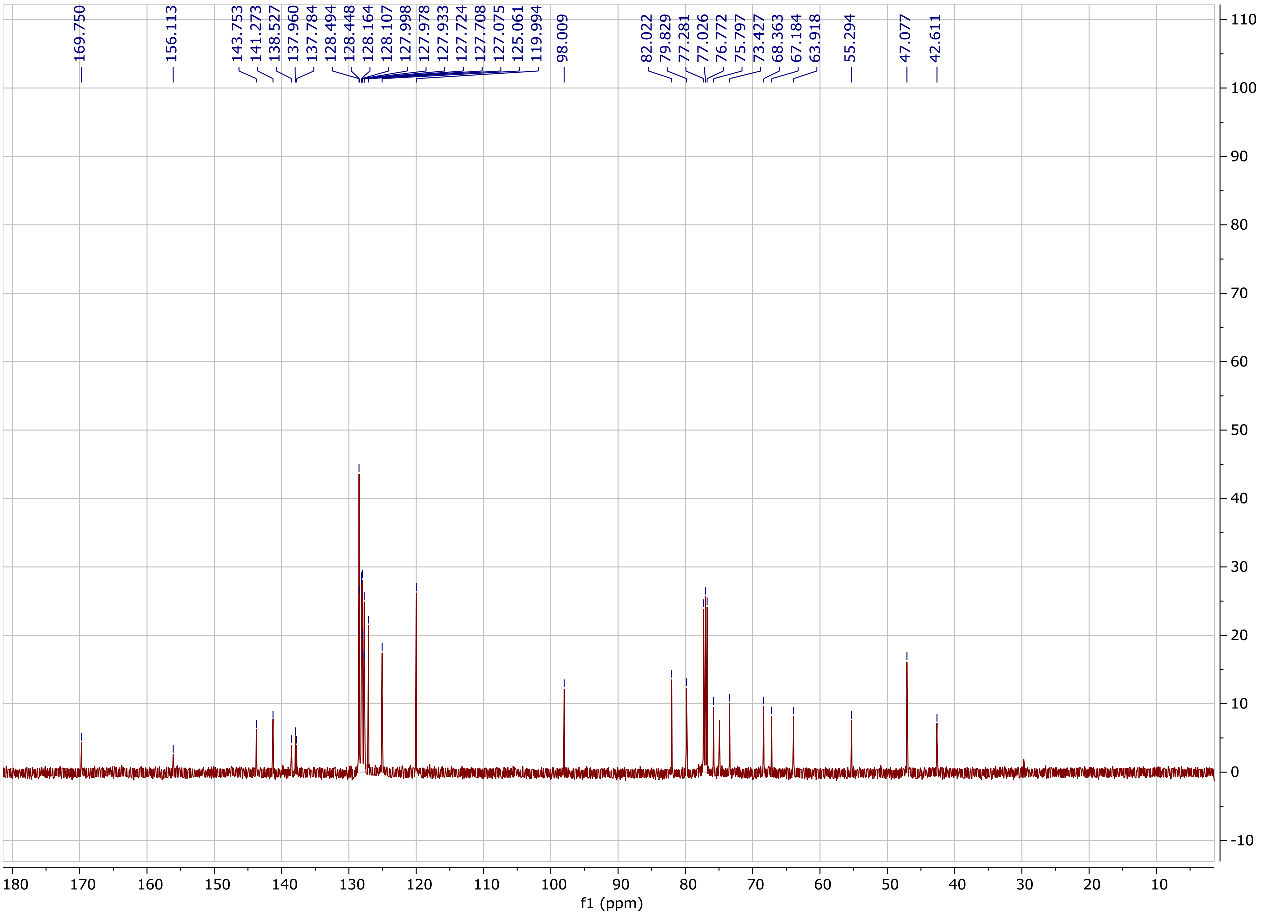

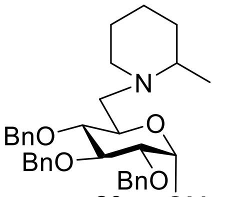

20

^{1}H-NMR ($CDCl_3$, 500 MHz) of **20**

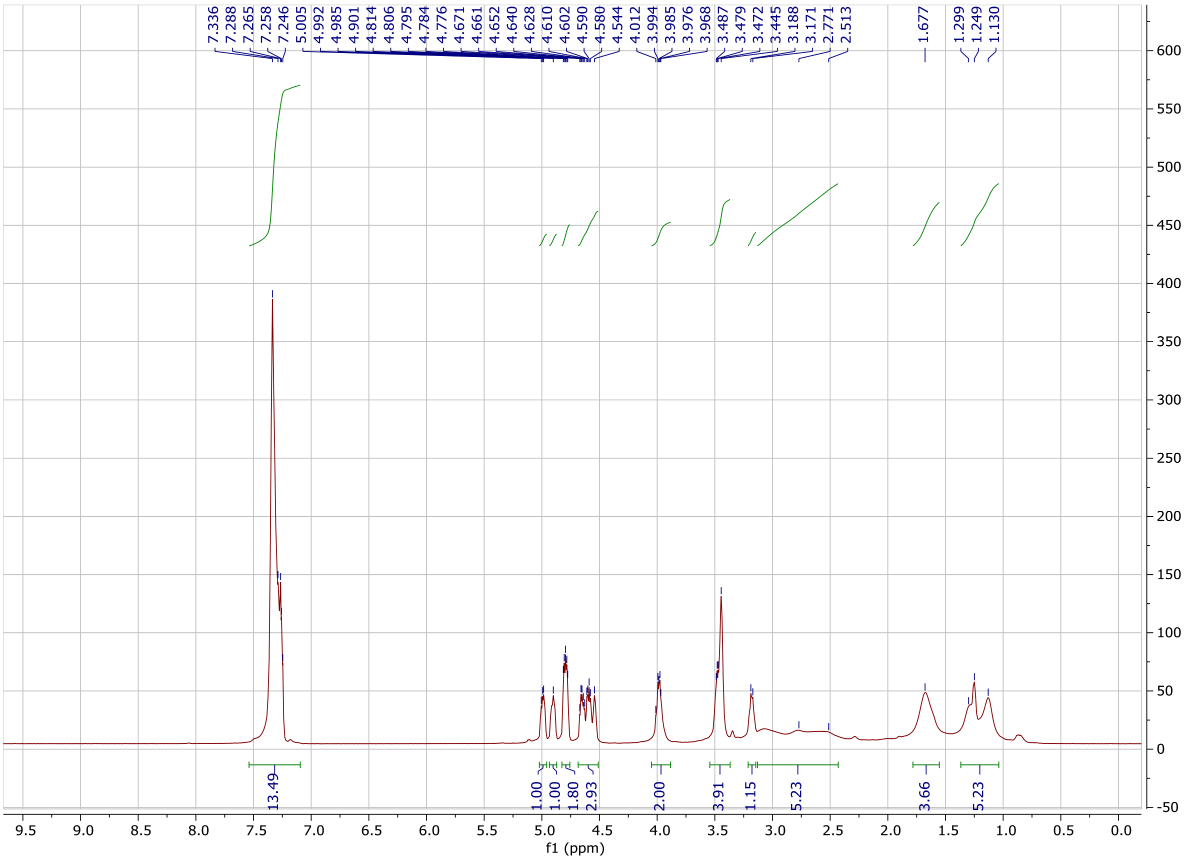

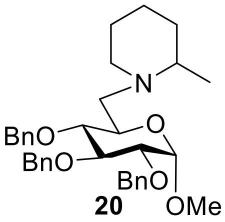

^{13}C-NMR ($CDCl_3$, 125 MHz) of **20**

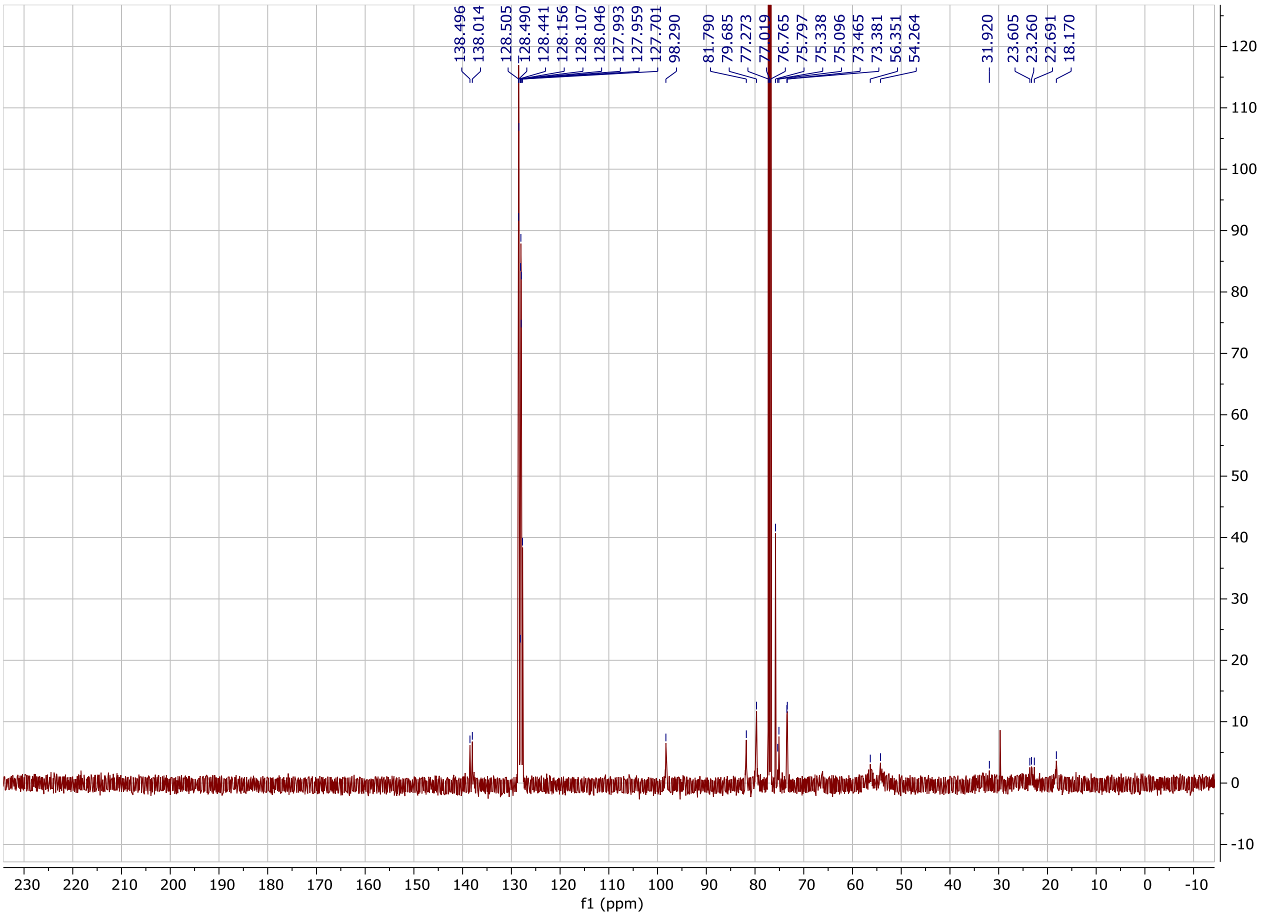

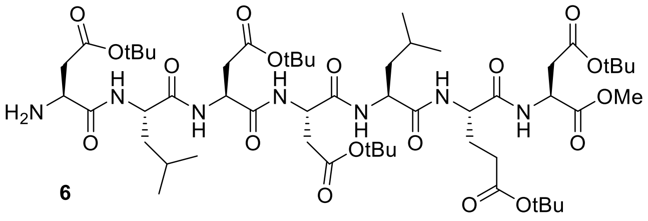

6

^{1}H-NMR ($CDCl_3$, 500 MHz) of **6**

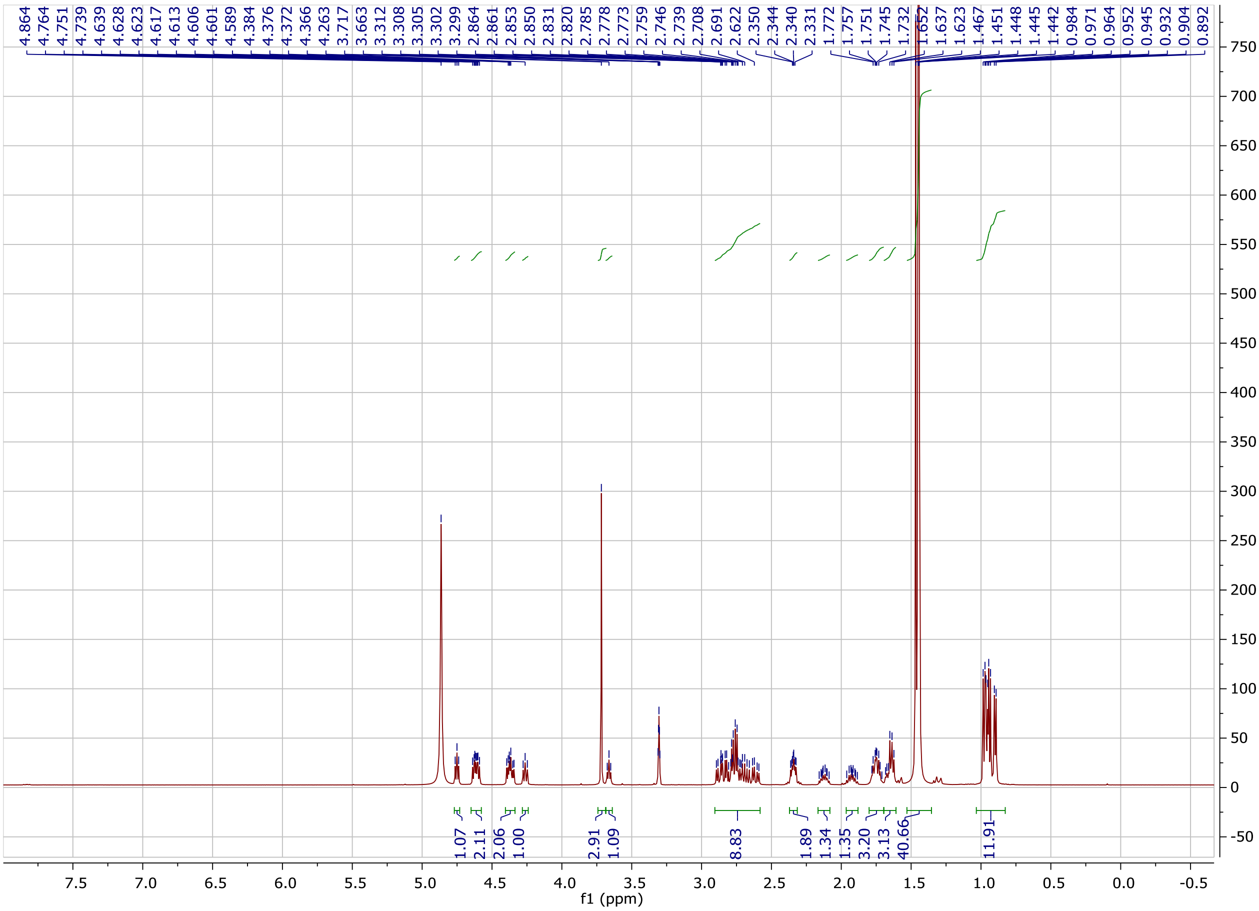

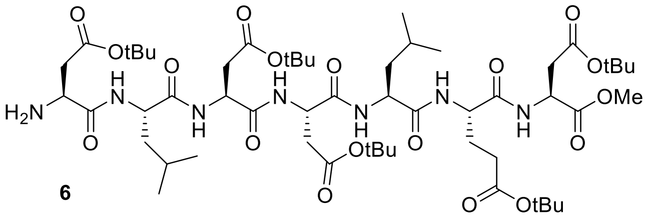

6

^{13}C-NMR ($CDCl_3$, 125 MHz) of **6**

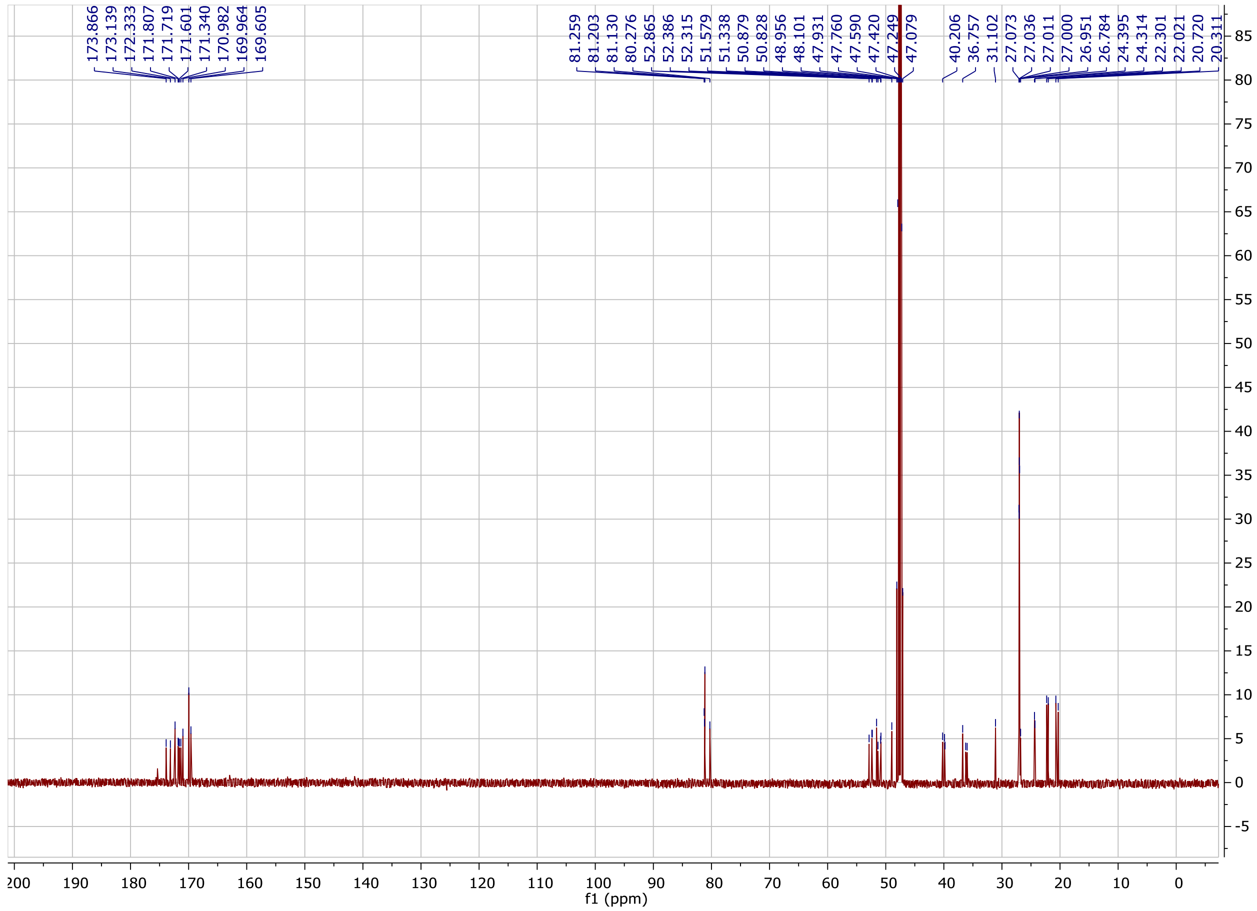

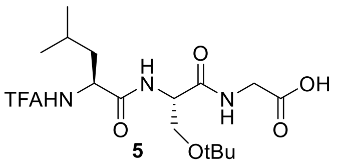

5

^{1}H-NMR ($CDCl_3$, 500 MHz) of **5**

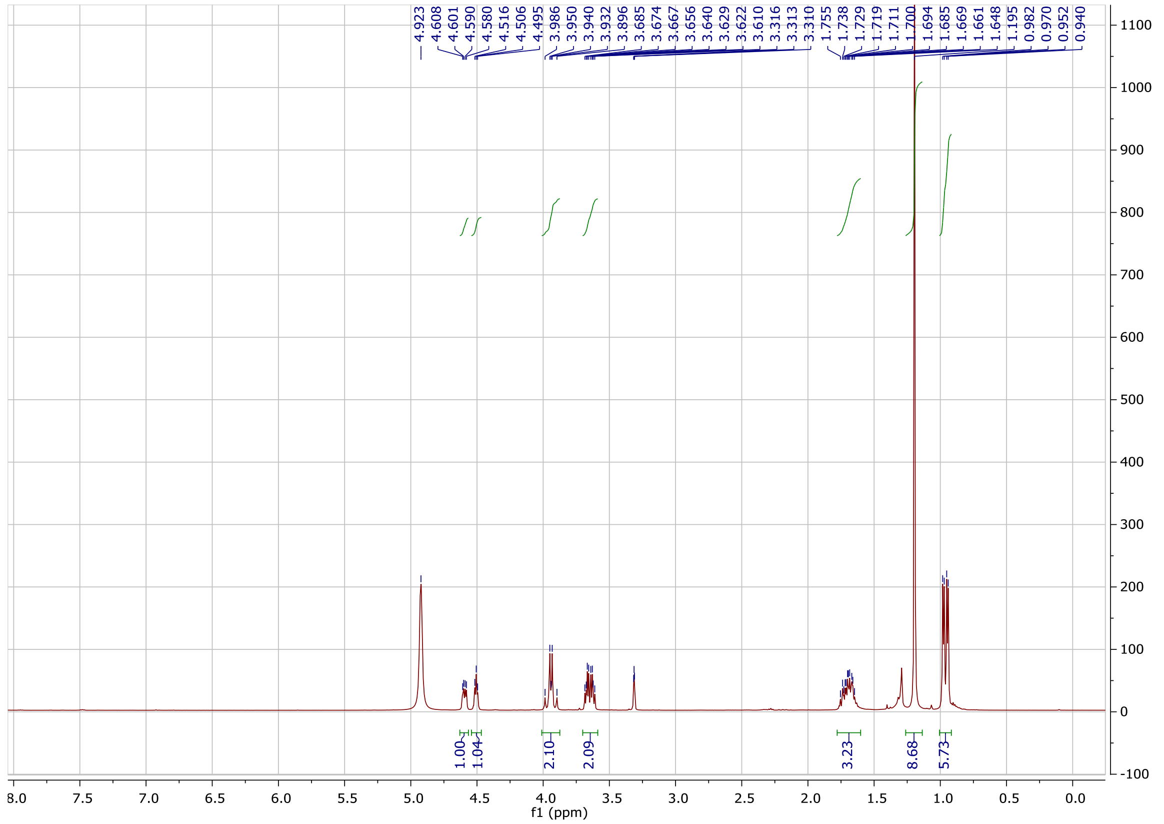

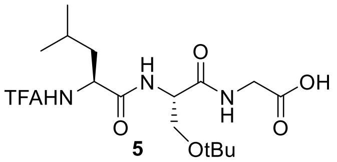

5

^{13}C-NMR ($CDCl_3$, 125 MHz) of **5**

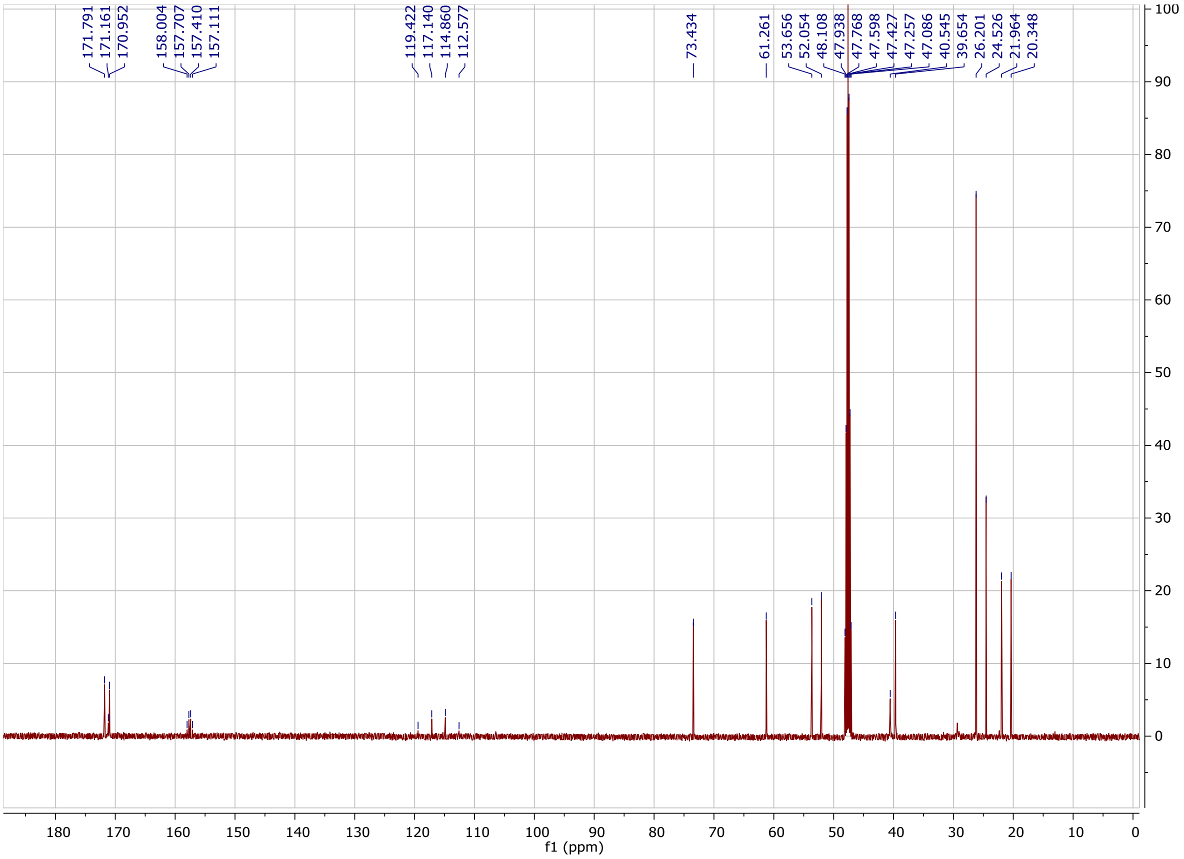

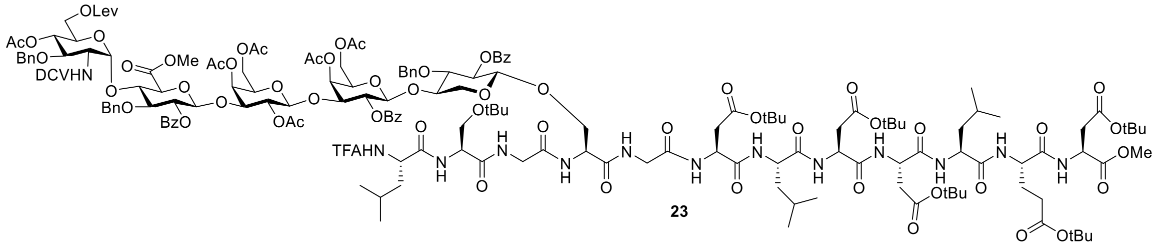

^{1}H-NMR ($CDCl_3$, 500 MHz) of **23**

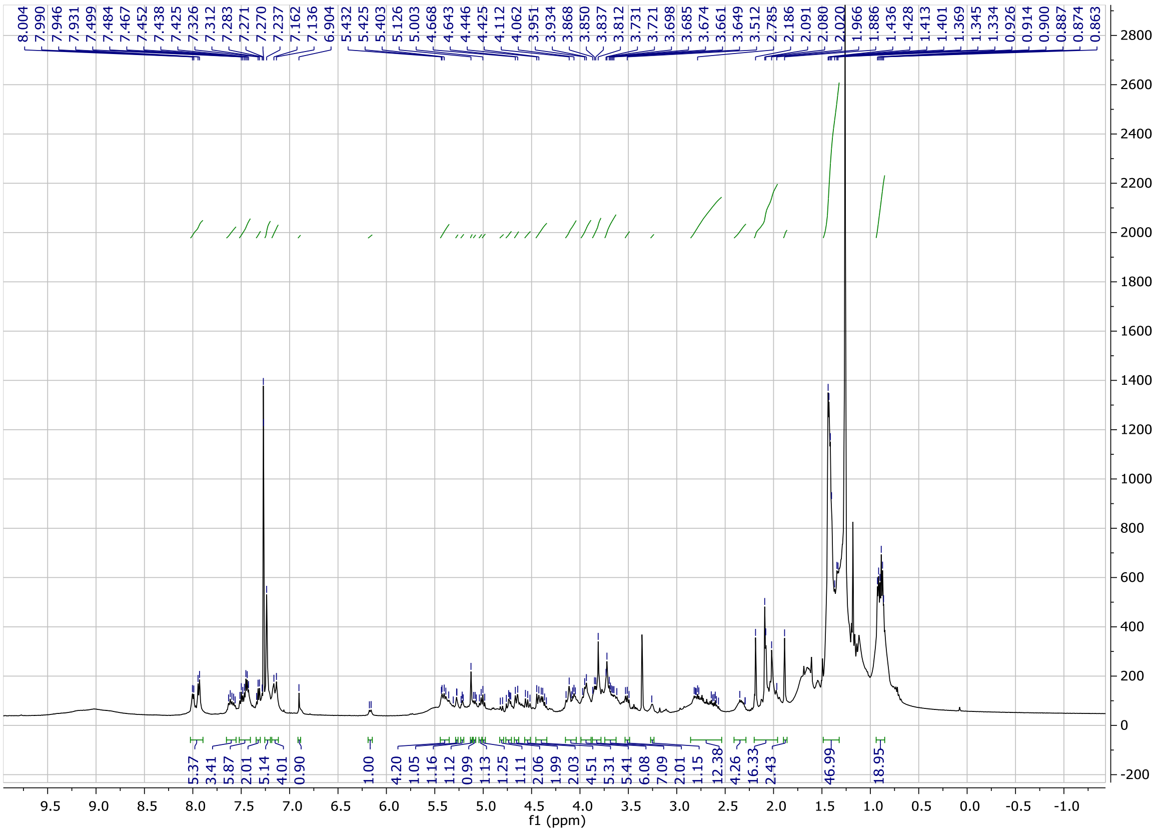

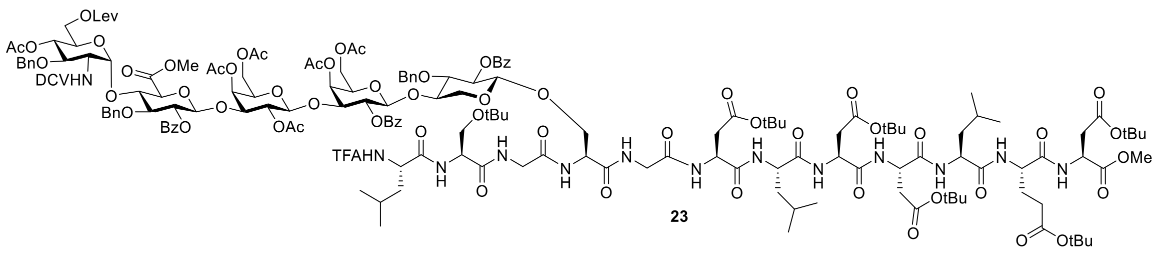

^{13}C-NMR ($CDCl_3$, 125 MHz) of **23**

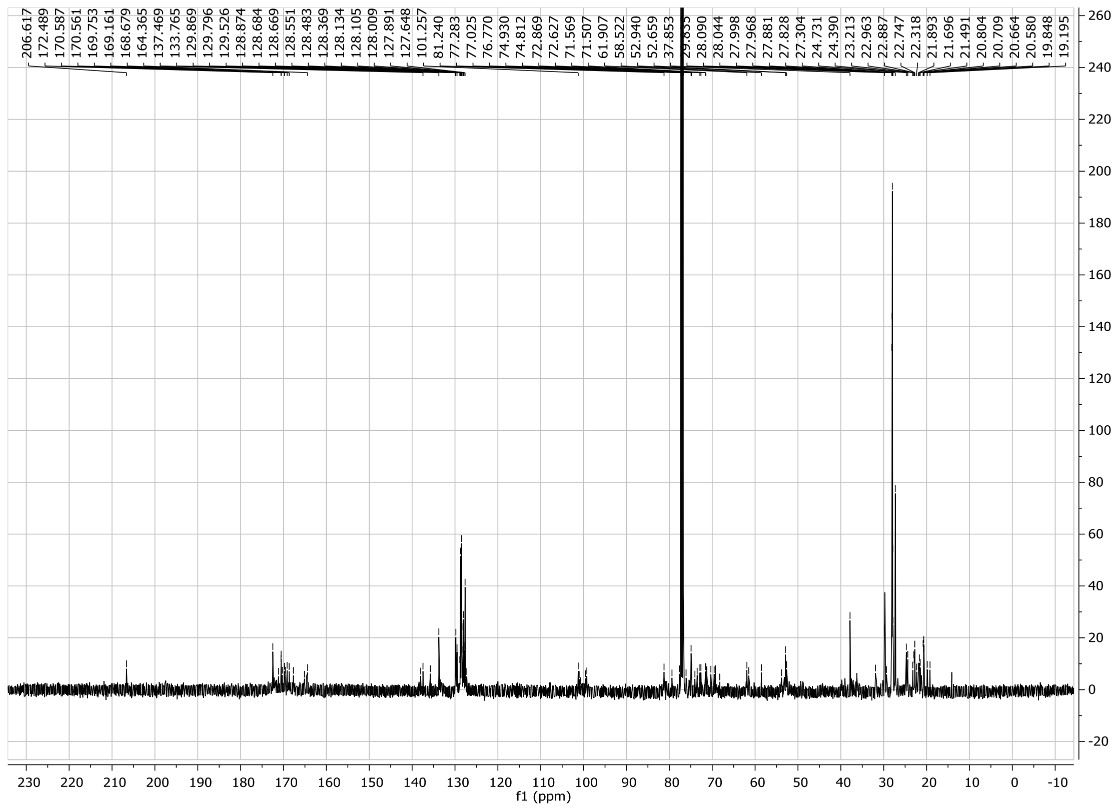

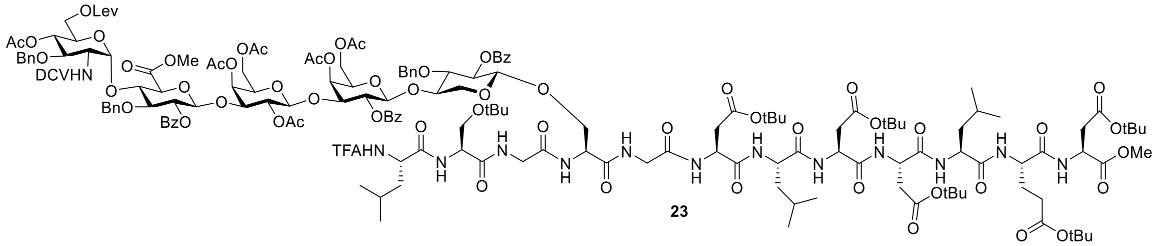

23

gHSQC ($CDCl_3$, 500 MHz) of **23**

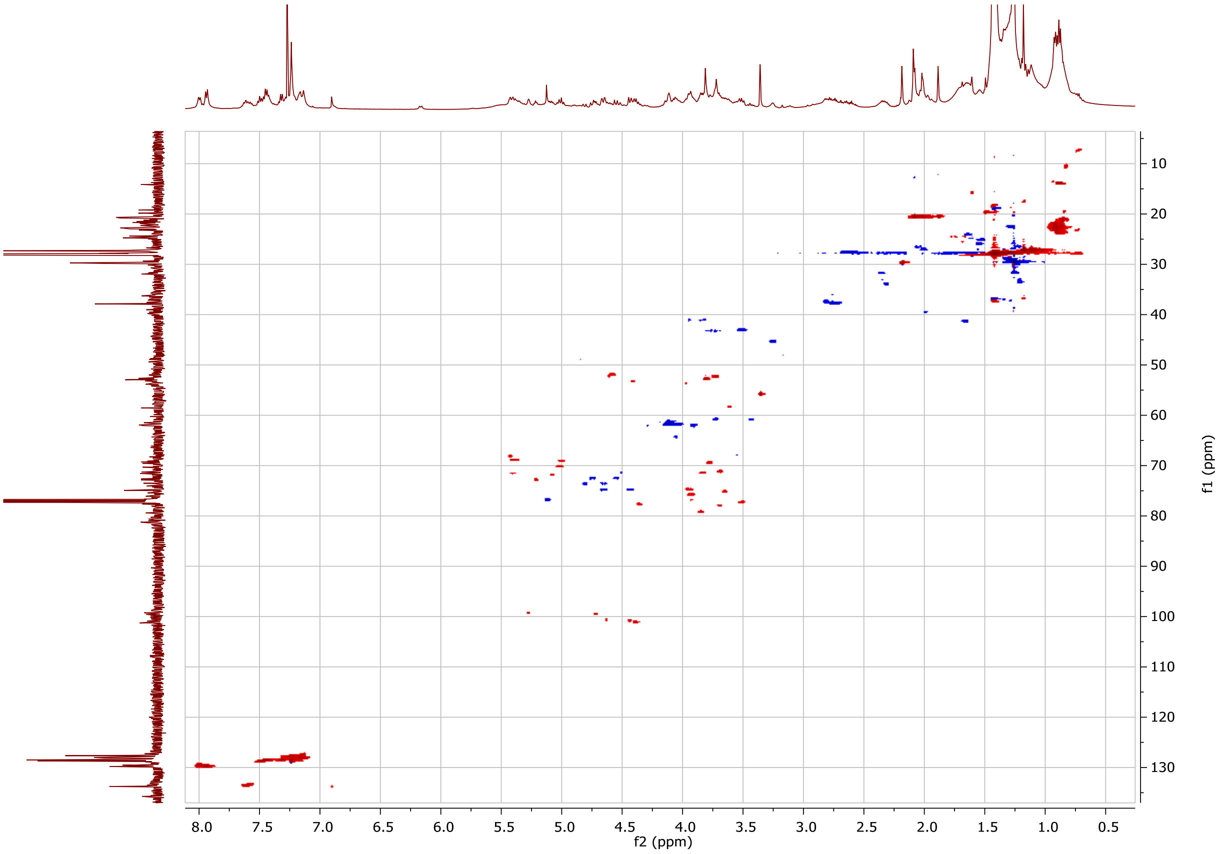

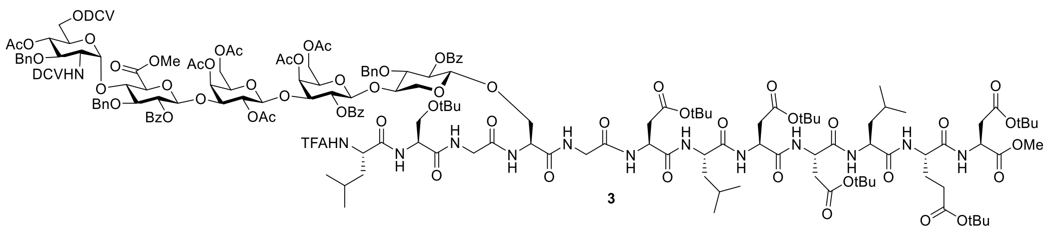

3

^{1}H-NMR ($CDCl_3$, 500 MHz) of **3**

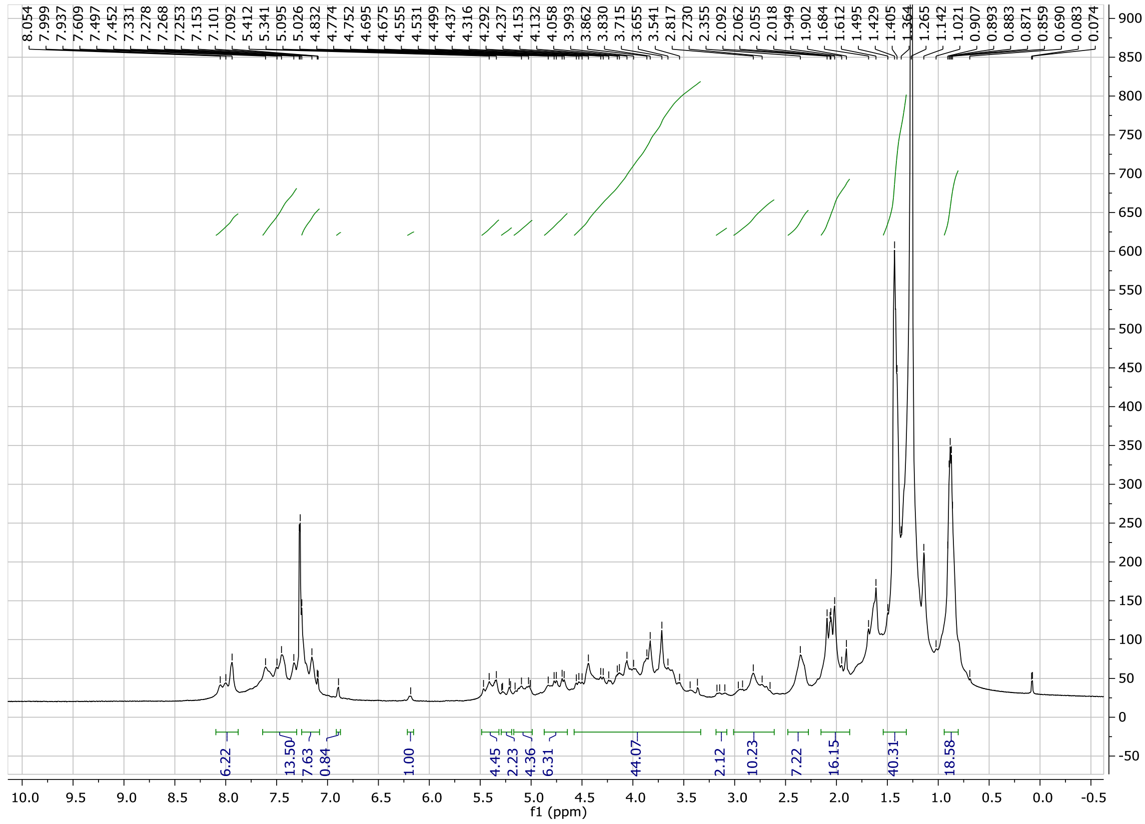

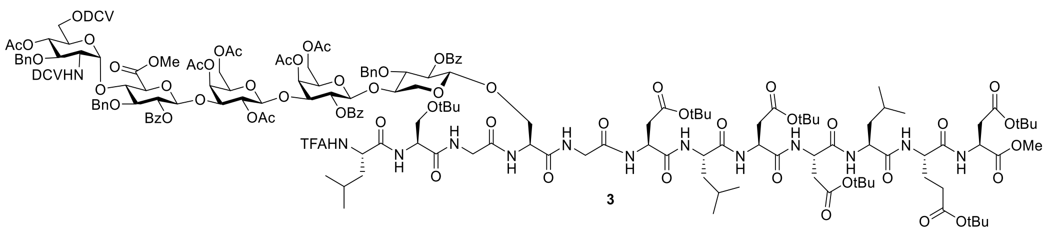

3

^{13}C-NMR ($CDCl_3$, 125 MHz) of **3**

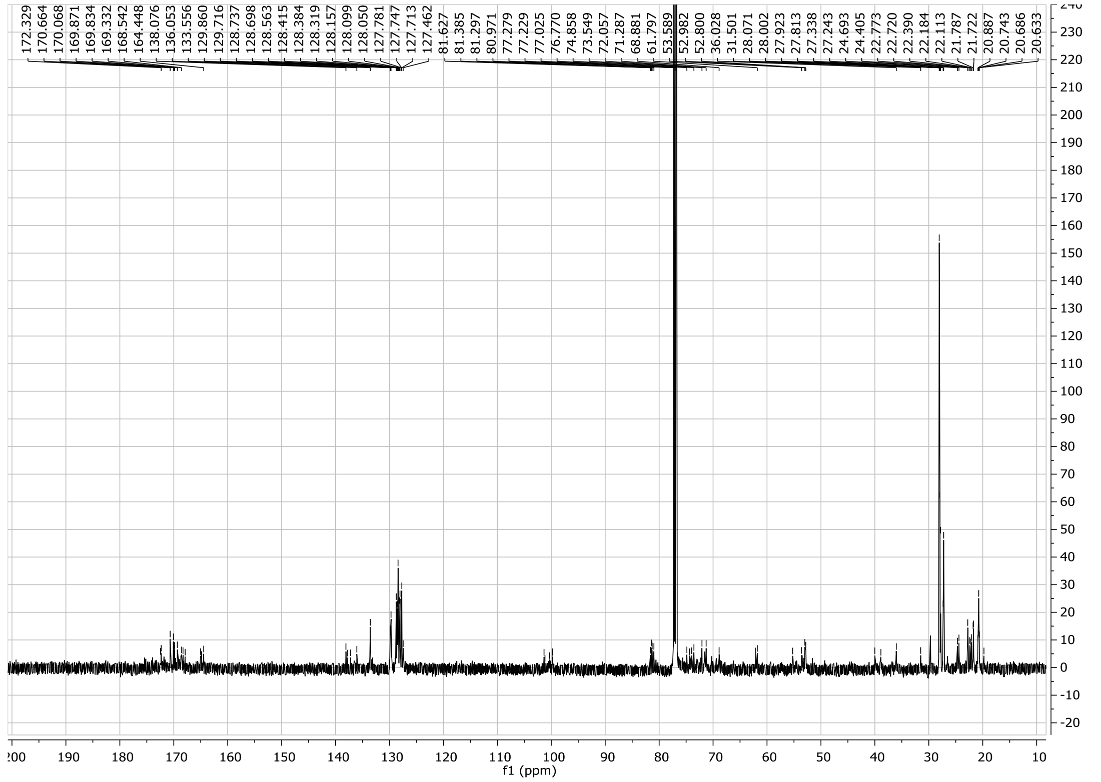

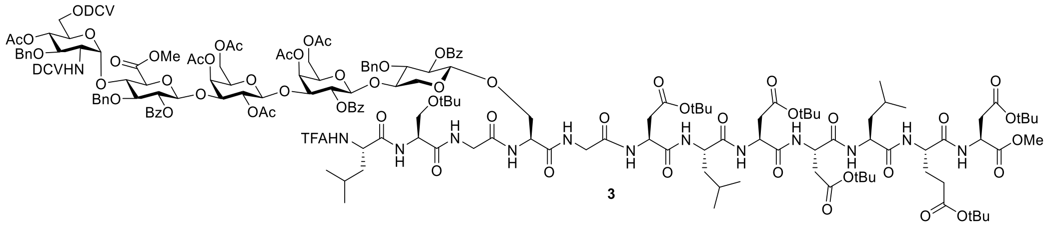

3

gHSQC ($CDCl_3$, 500 MHz) of **3**

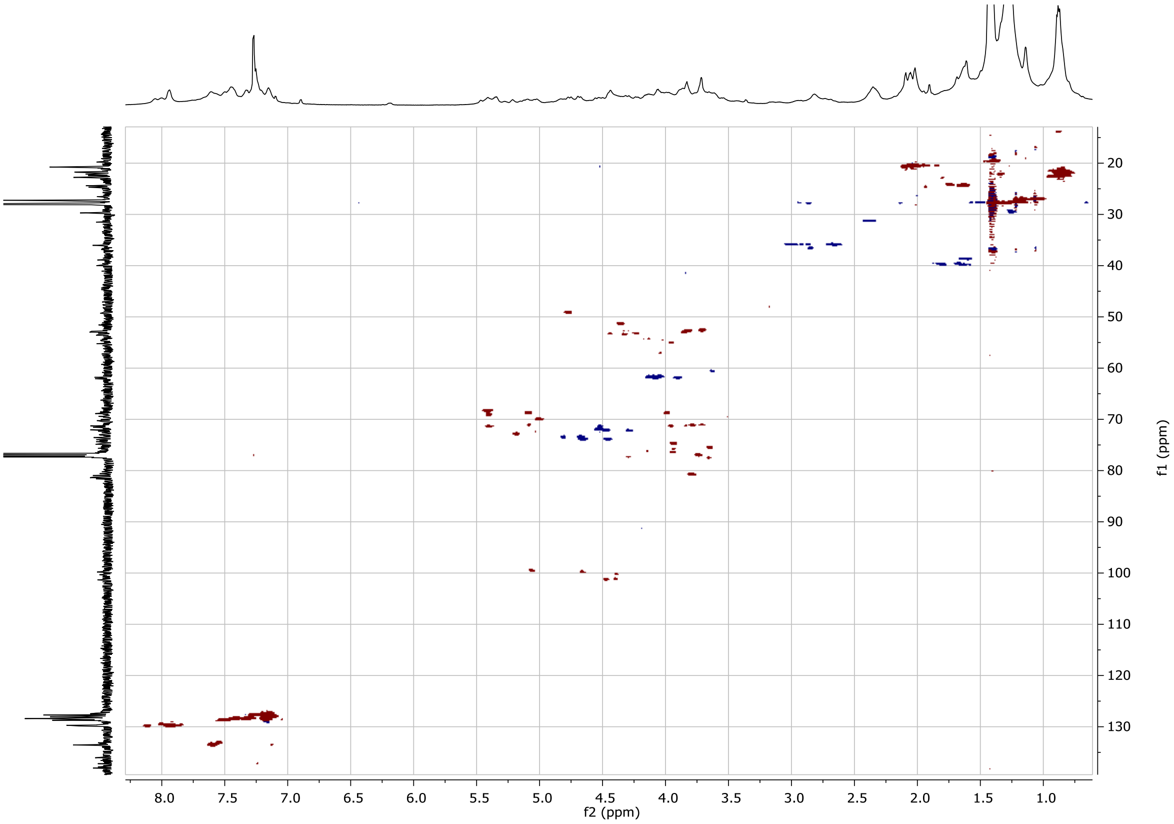

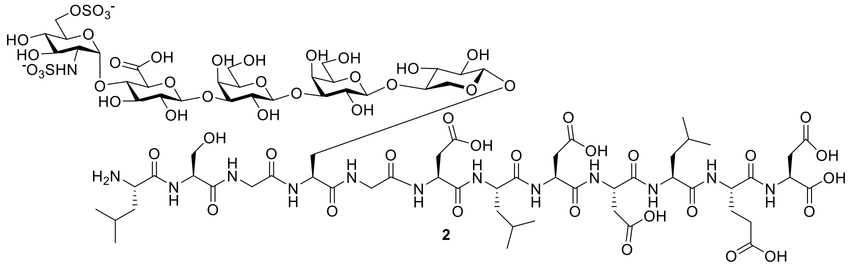

2

^{1}H-NMR (D_2O, 500 MHz) of **2**

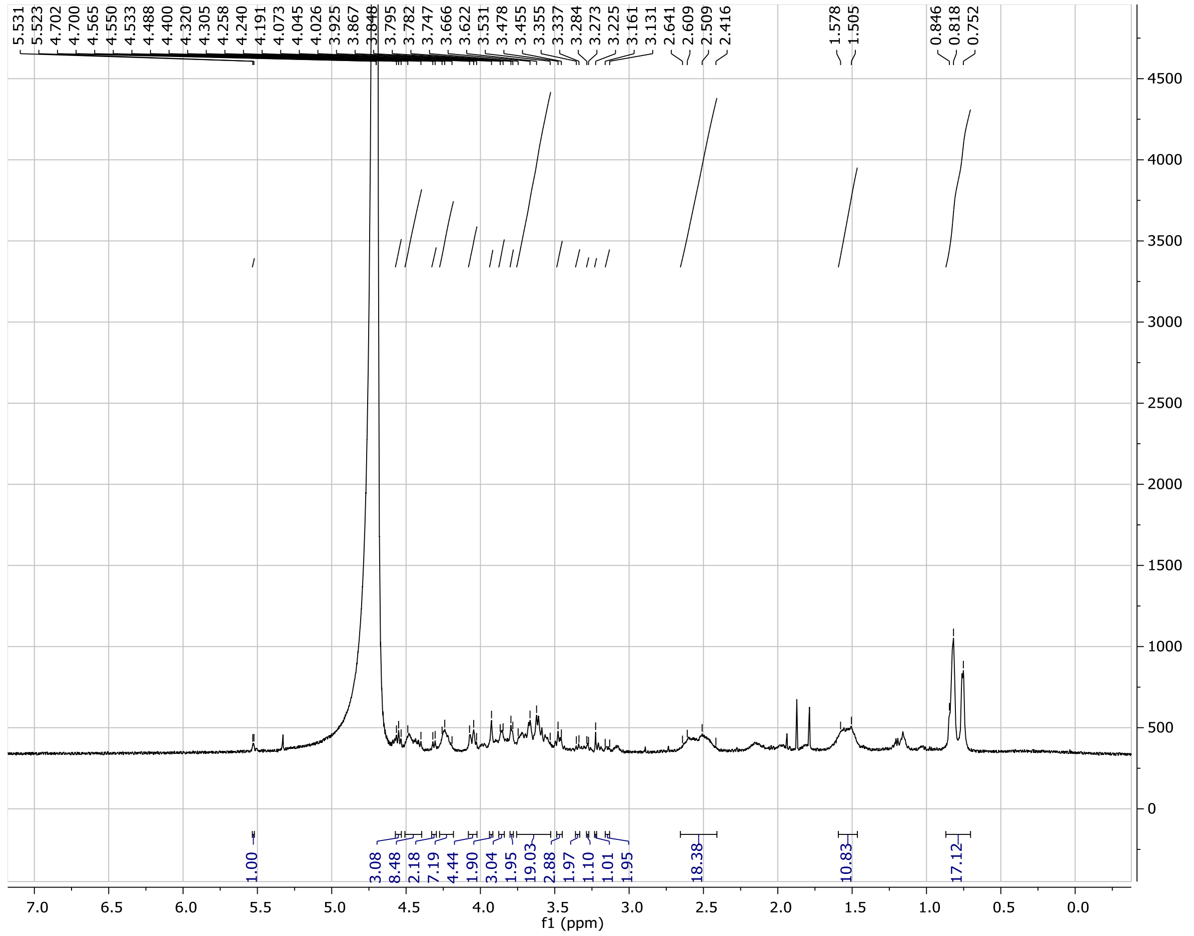

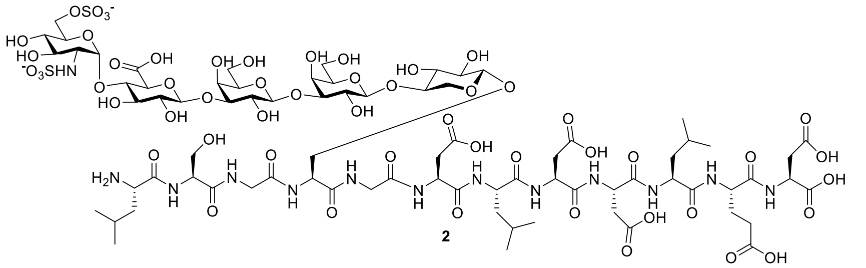

2

gHSQC ($CDCl_3$, 500 MHz) of **2**

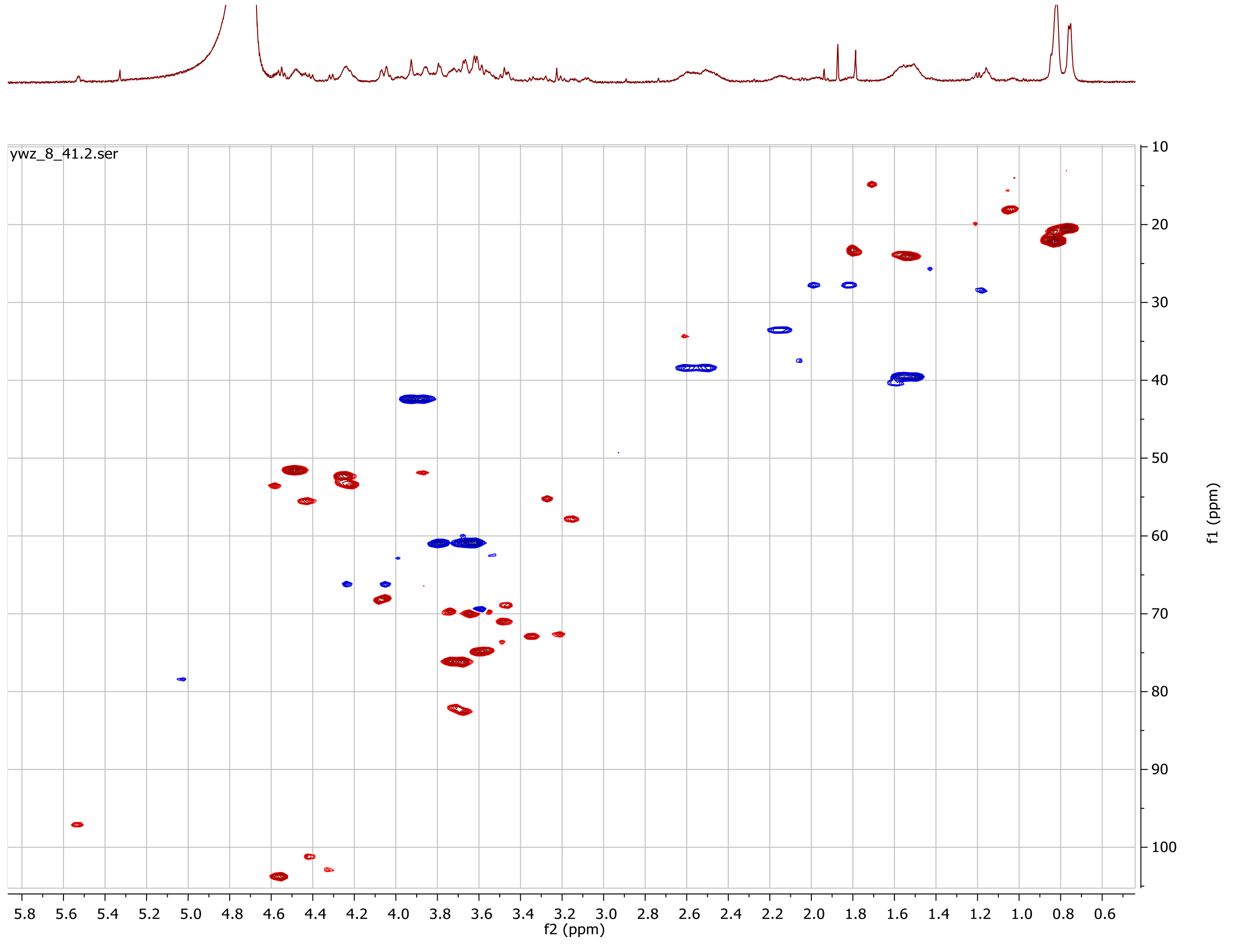

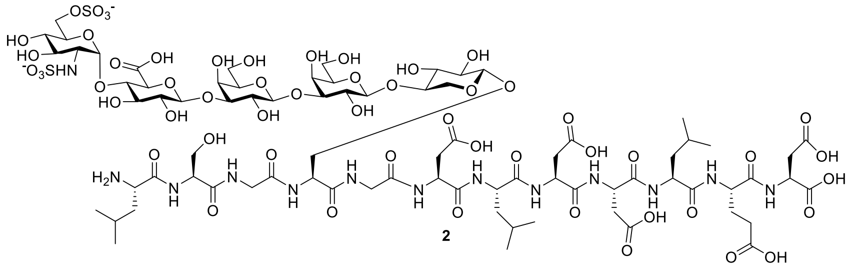

2

HRMS of **2**

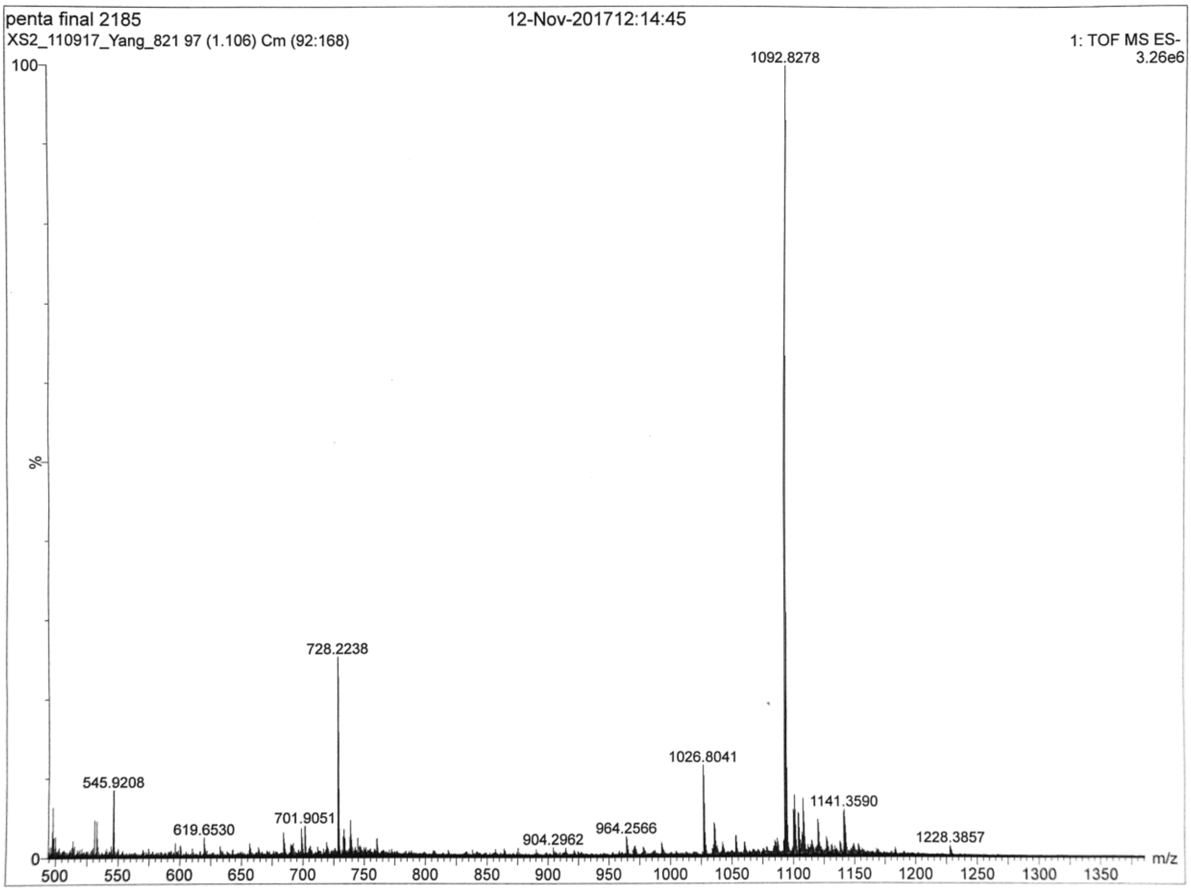

penta final 2185
12-Nov-201712:14:45
XS2_110917_Yang_821 97 (1.106) Cm (92:168)
1: TOF MS ES-
3.26e6
1092.8278
728.2238
545.9208
619.6530
701.9051
904.2962
964.2566
1026.8041
1141.3590
1228.3857
%
m/z

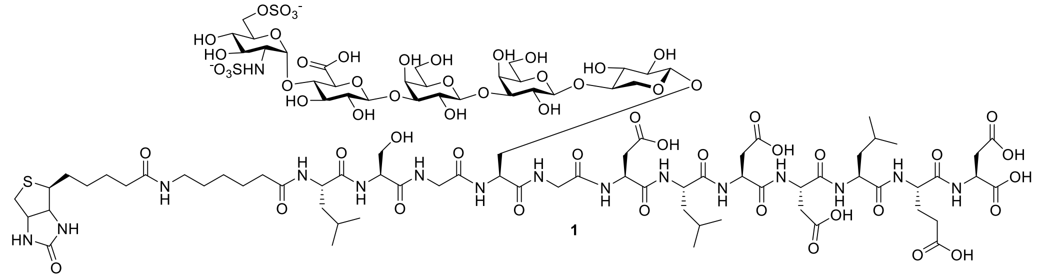

1

^{1}H-NMR (D_2O, 500 MHz) of **1**

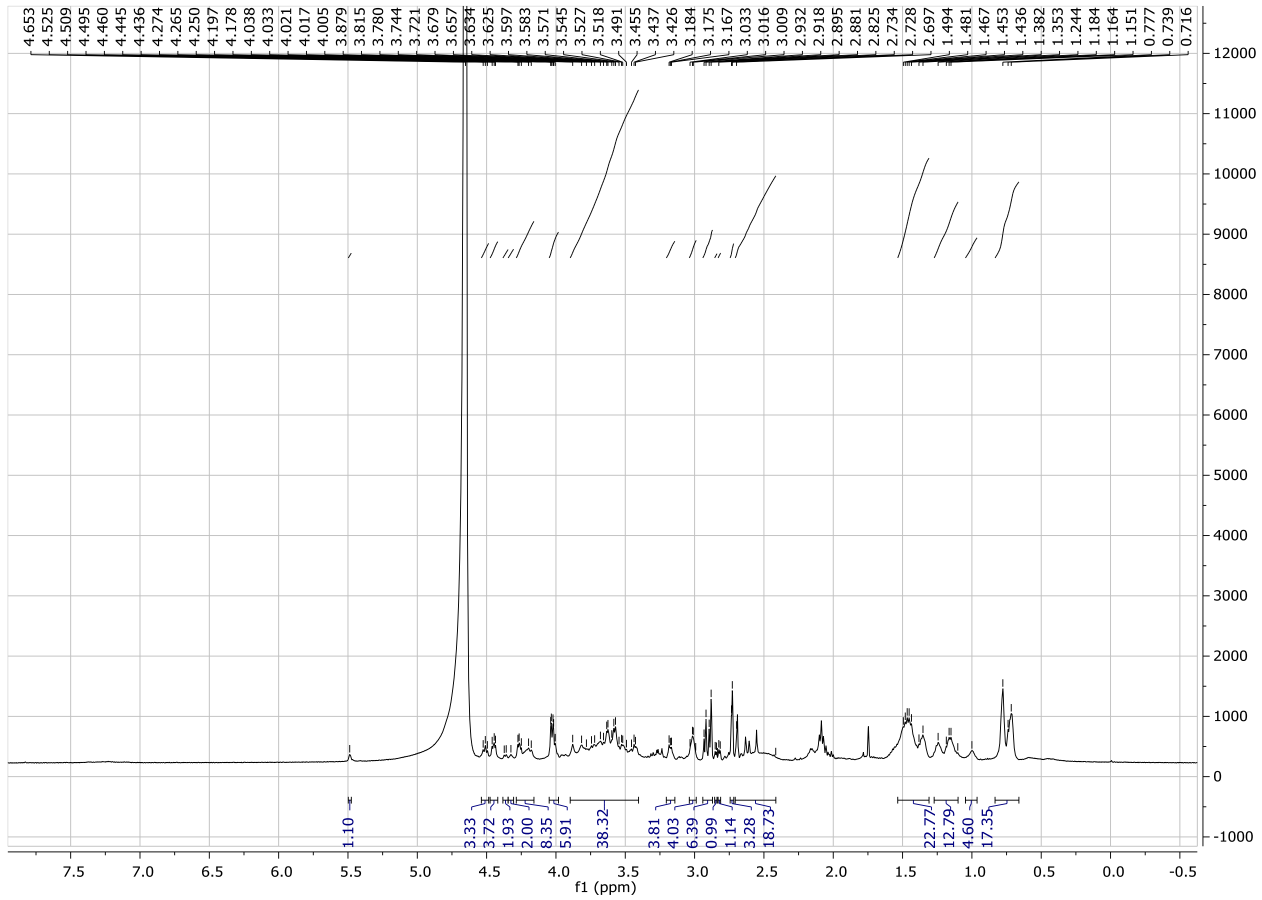

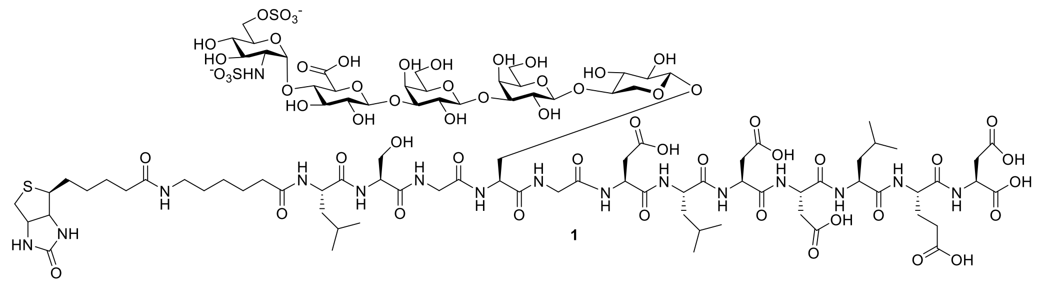

1

MS of **1**

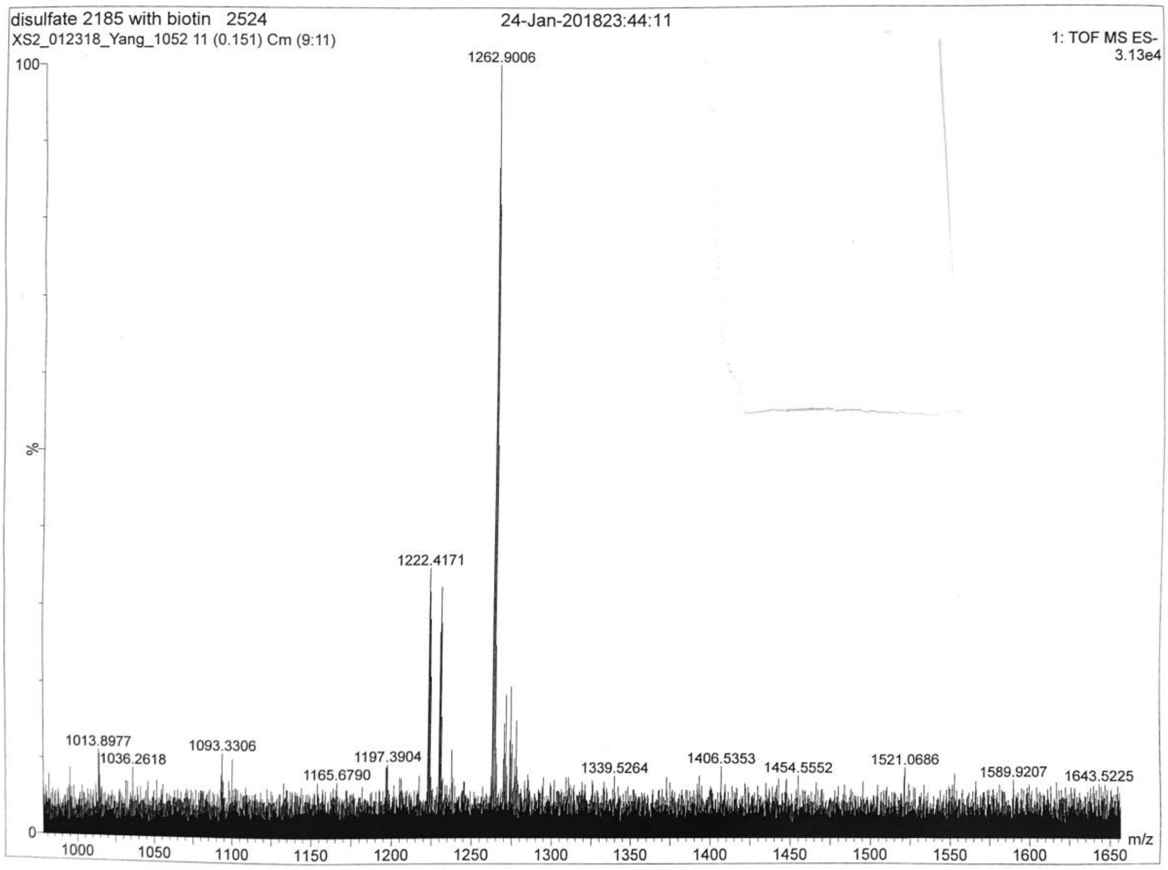

disulfate 2185 with biotin 2524
24-Jan-201823:44:11
XS2_012318_Yang_1052 11 (0.151) Cm (9:11)
1: TOF MS ES-
3.13e4
1262.9006
1222.4171
1013.8977
1036.2618
1093.3306
1165.6790
1197.3904
1339.5264
1406.5353
1454.5552
1521.0686
1589.9207
1643.5225
%
m/z

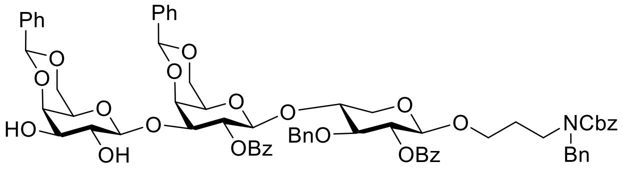

25

^{1}H-NMR ($CDCl_3$, 500 MHz) of **25**

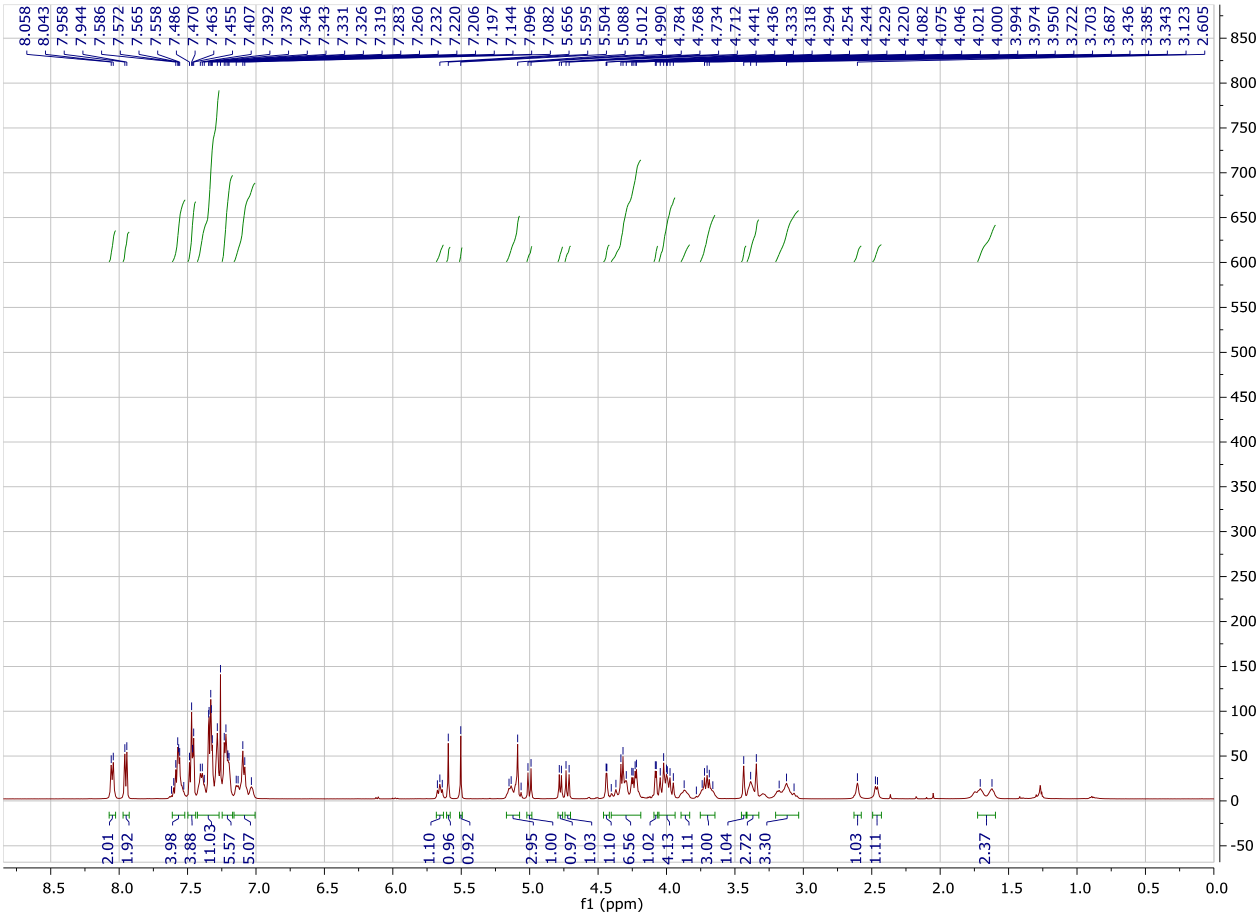

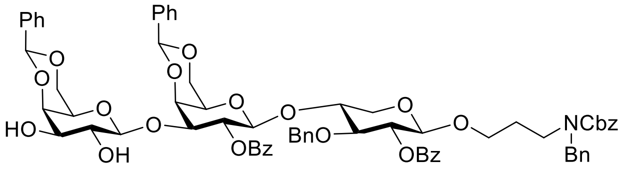

25

^{13}C-NMR ($CDCl_3$, 125 MHz) of **25**

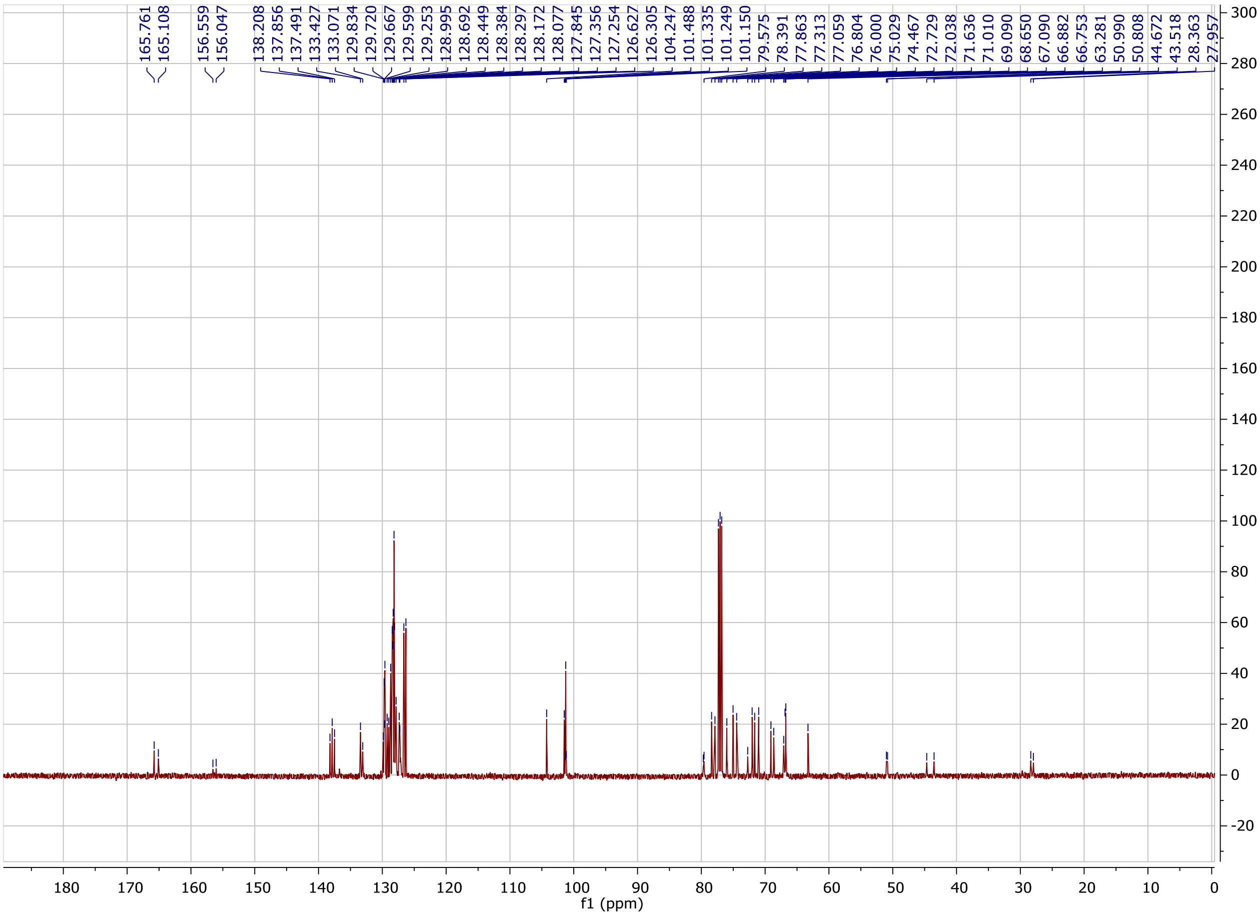

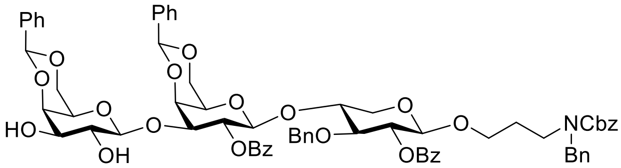

25

Coupled gHSQC (CDCl$_3$, 500 MHz) of **25**

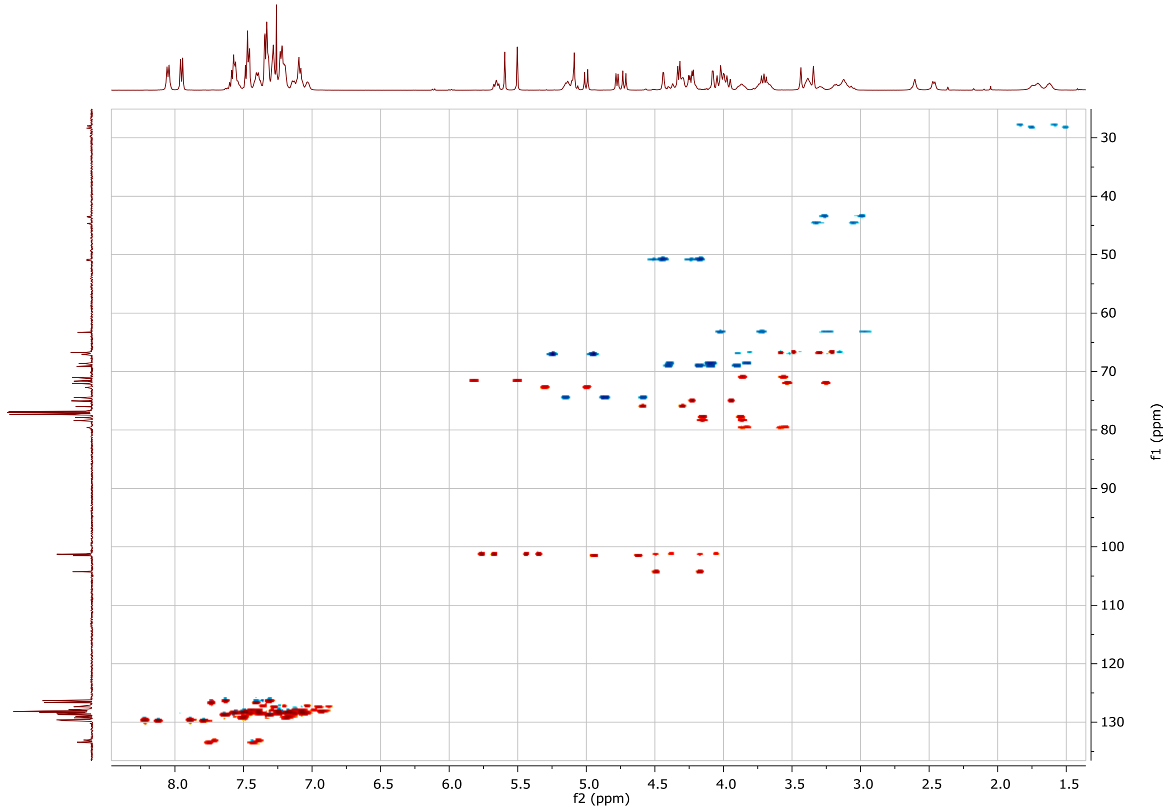

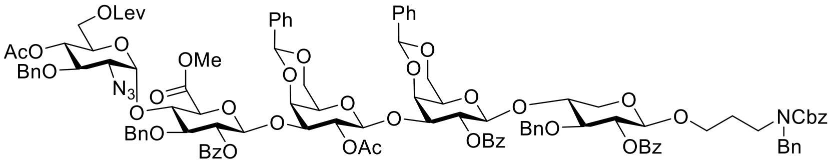

26

^{1}H-NMR ($CDCl_3$, 500 MHz) of **26**

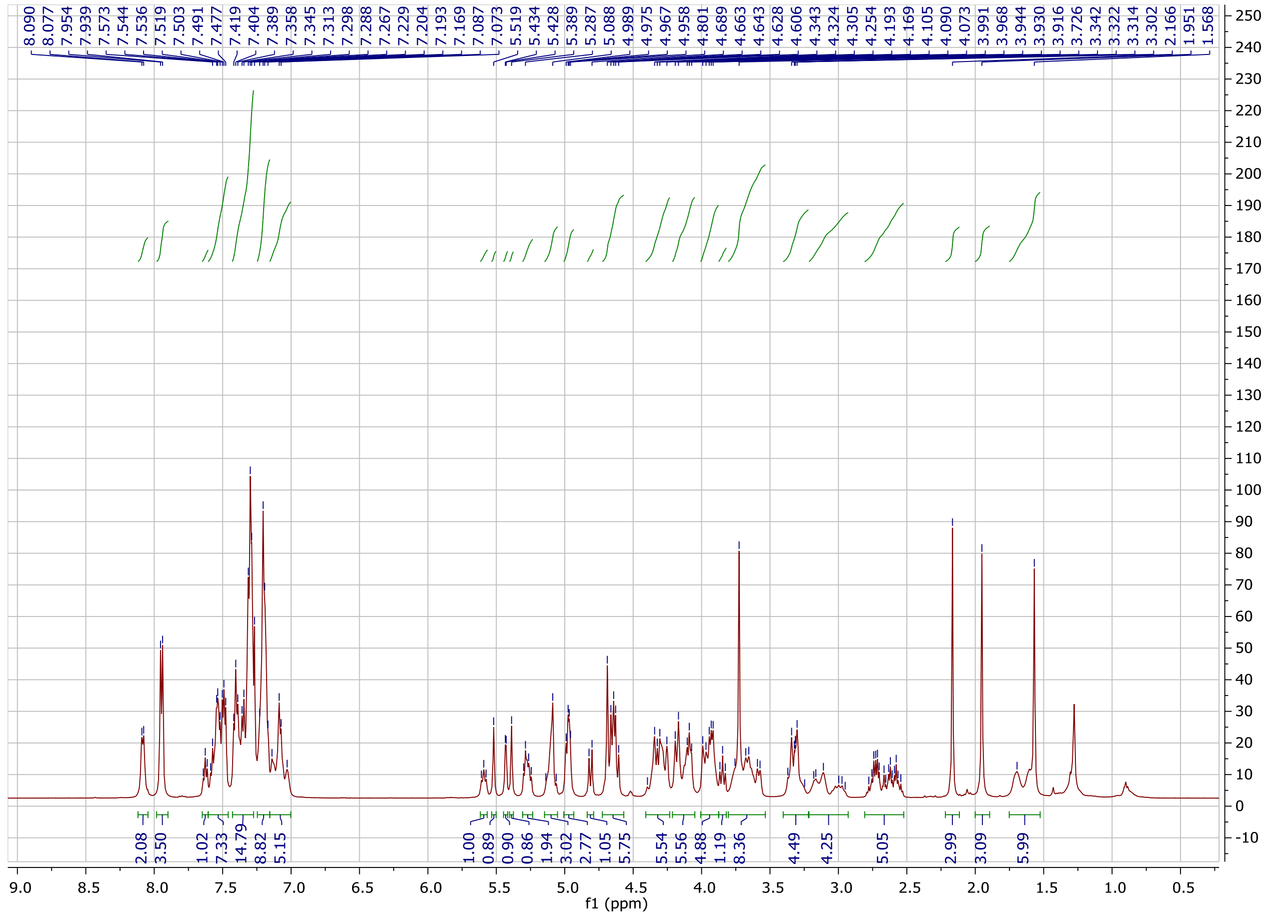

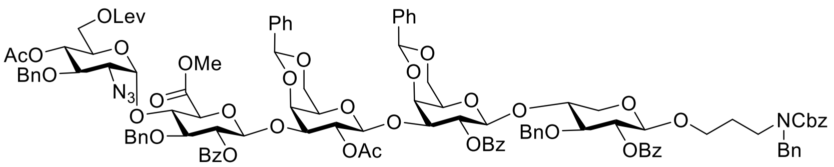

26

^{13}C-NMR ($CDCl_3$, 125 MHz) of **26**

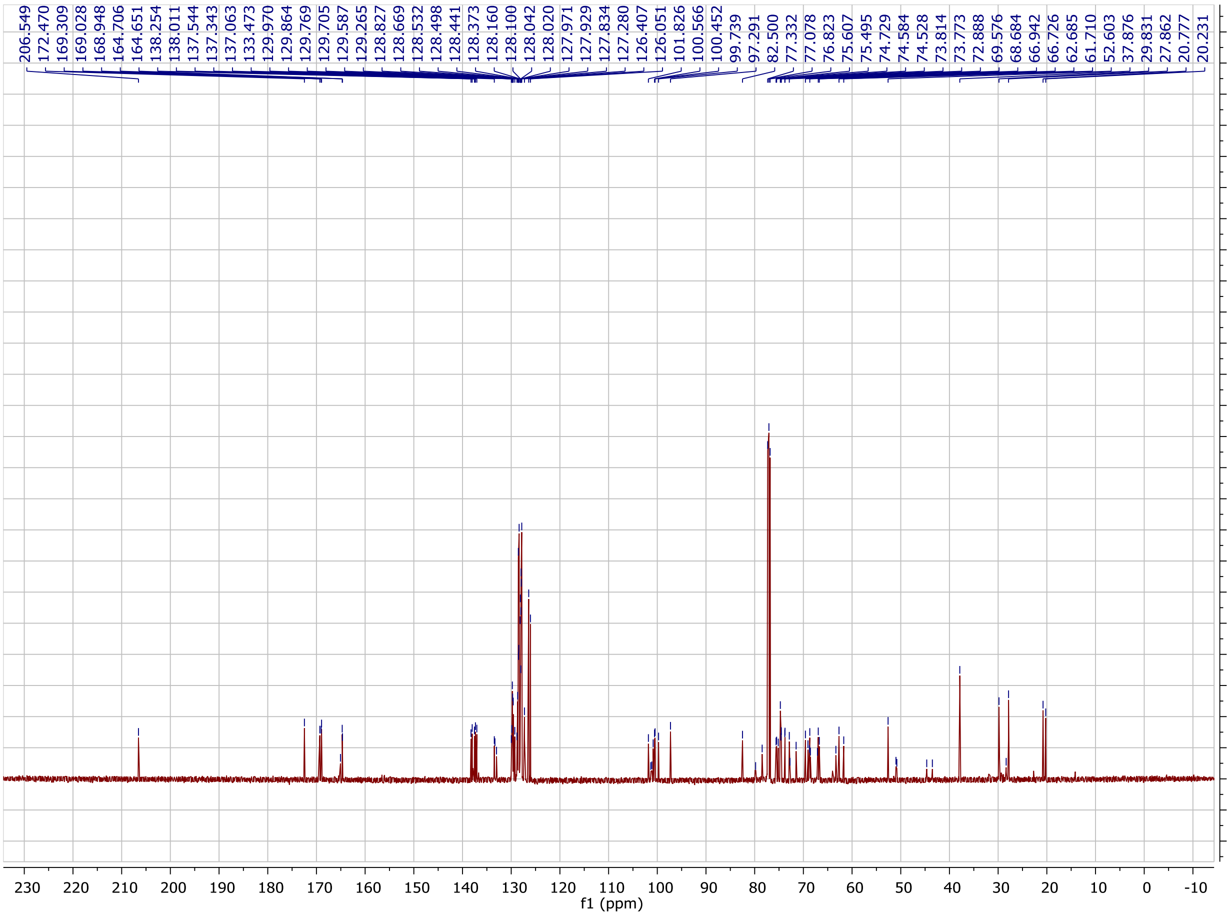

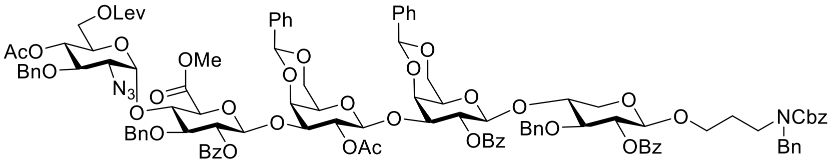

26

gHSQC ($CDCl_3$, 500 MHz) of **26**

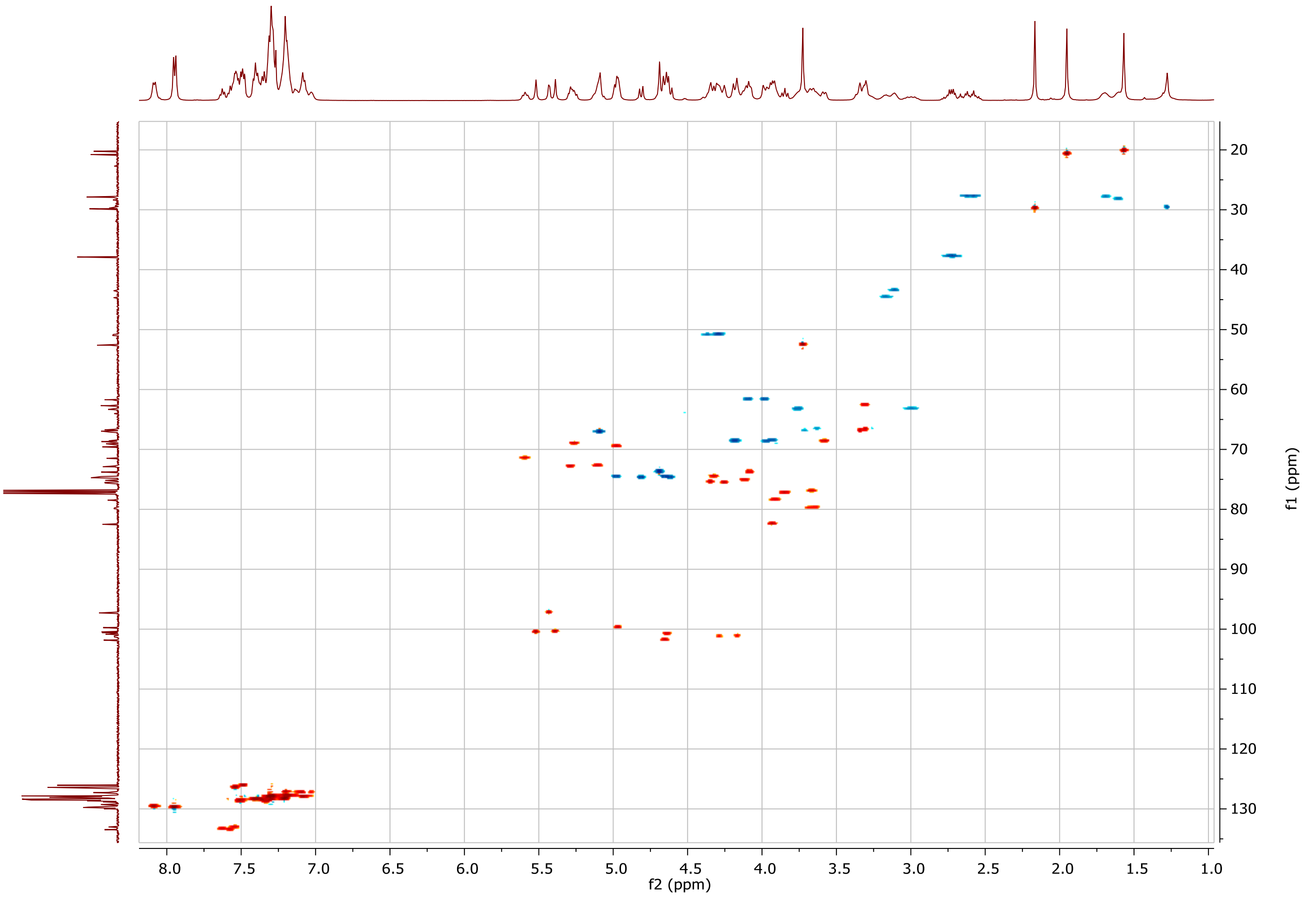

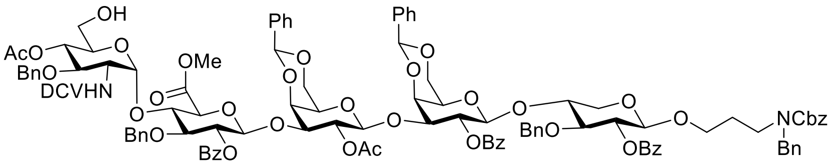

27

^{1}H-NMR ($CDCl_3$, 500 MHz) of **27**

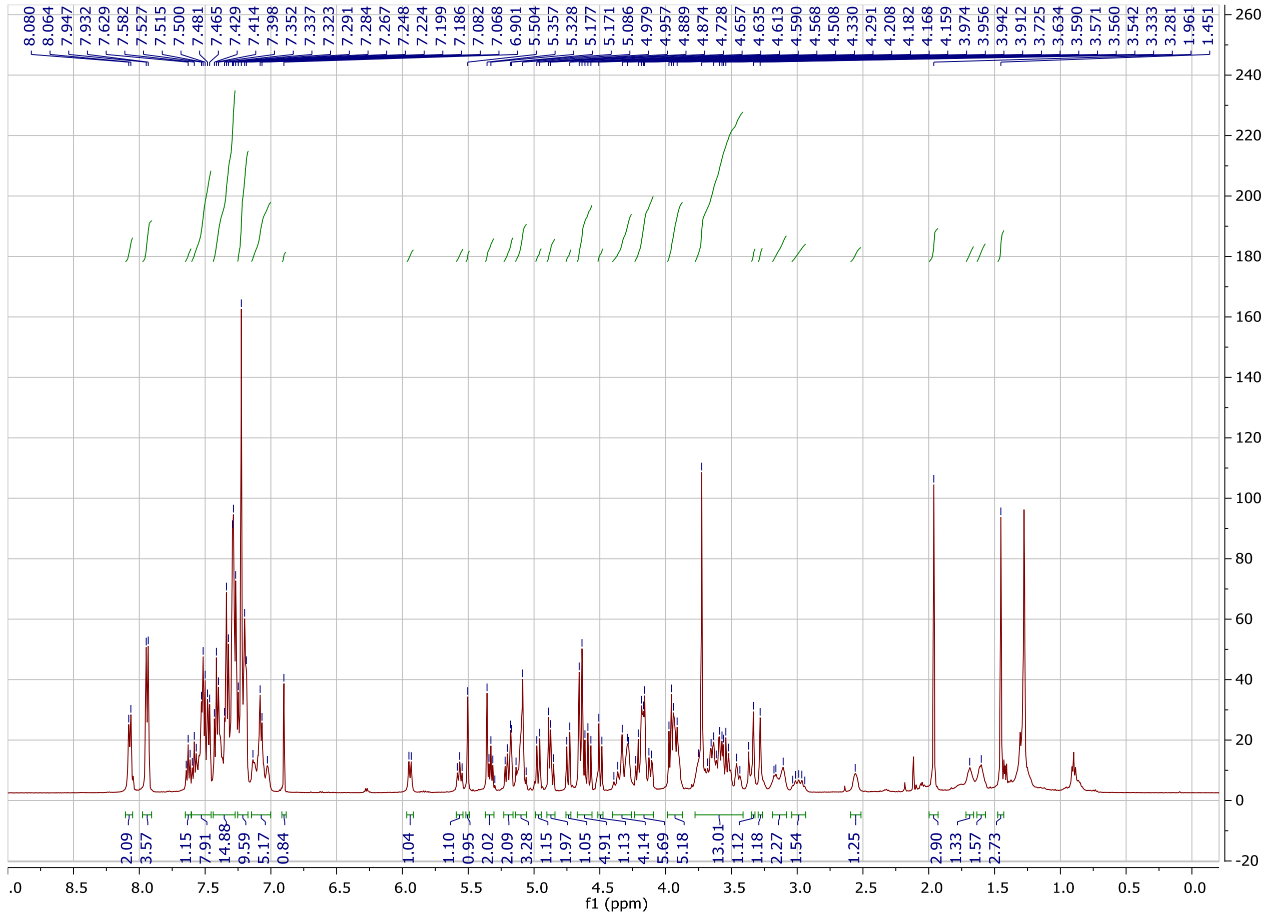

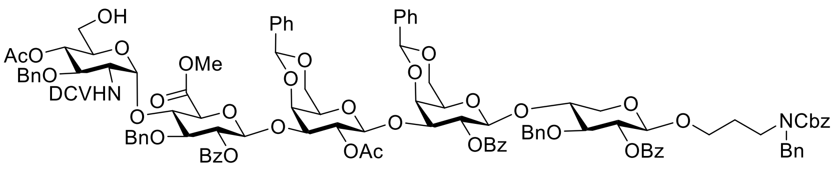

27

^{13}C-NMR ($CDCl_3$, 125 MHz) of **27**

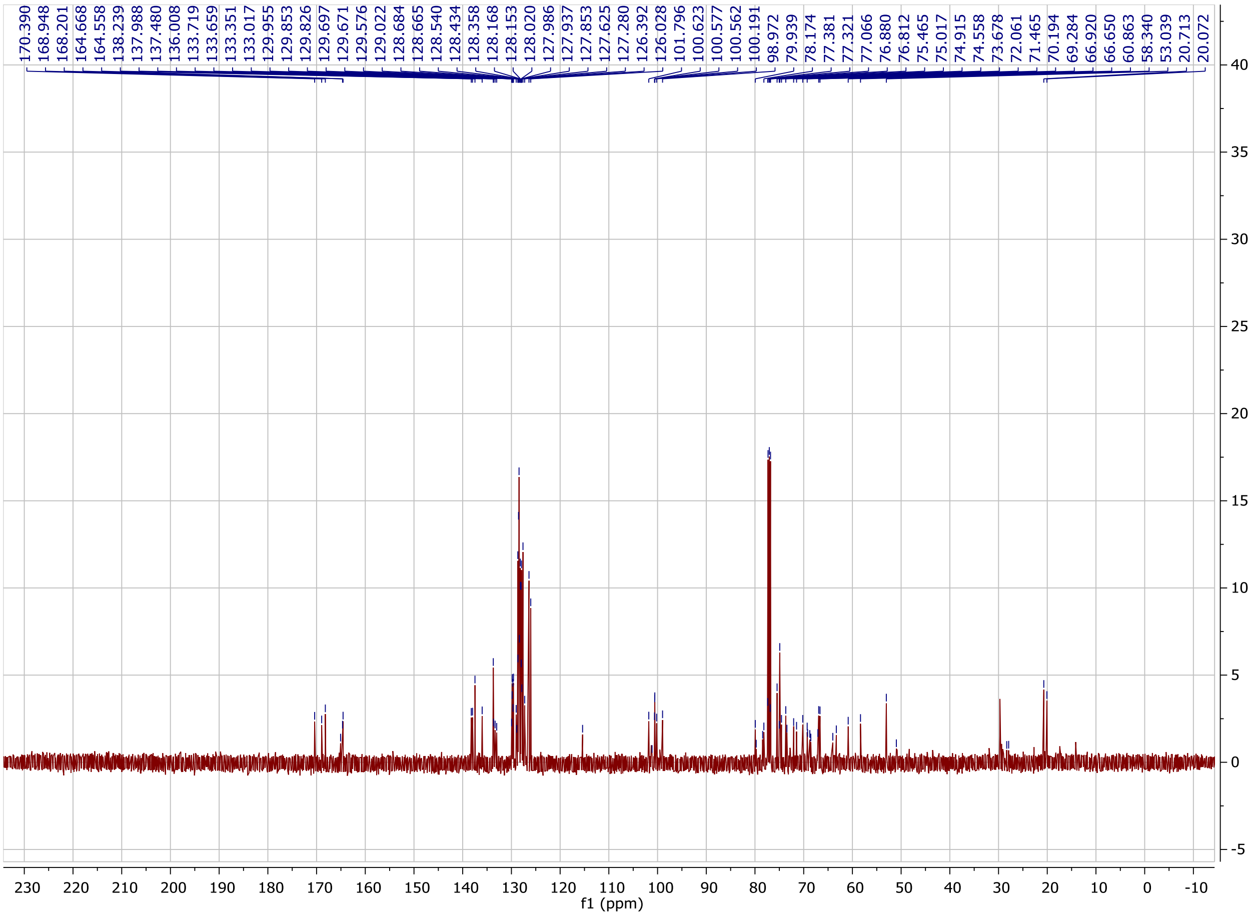

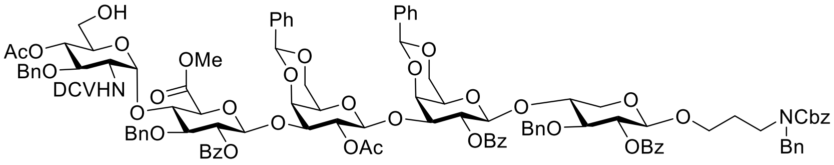

27

gHSQC ($CDCl_3$, 500 MHz) of **27**

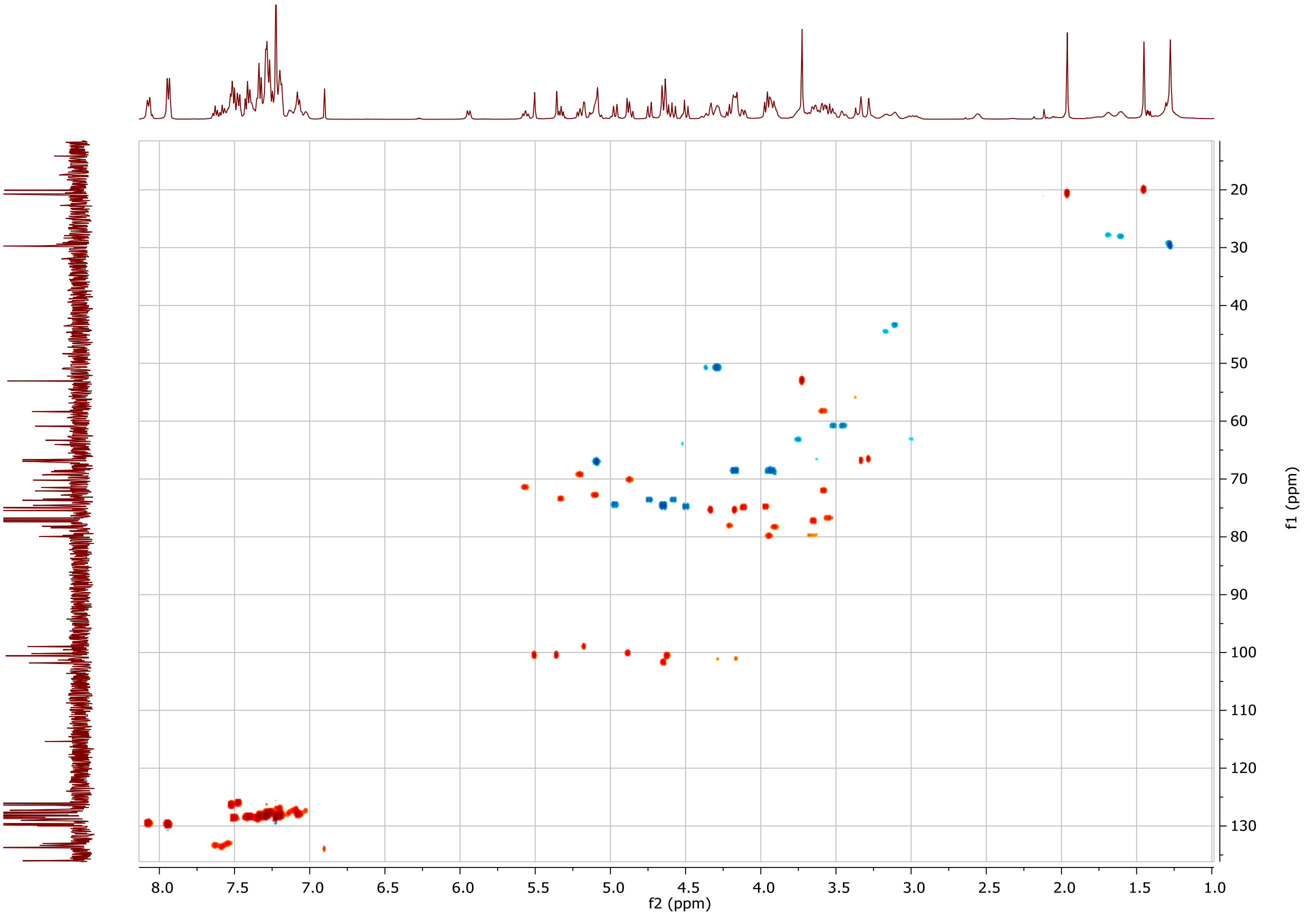

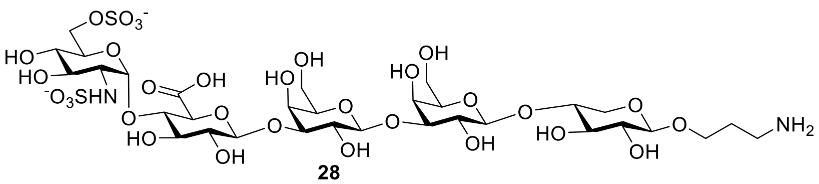

^{1}H-NMR (D_2O, 500 MHz) of **28**

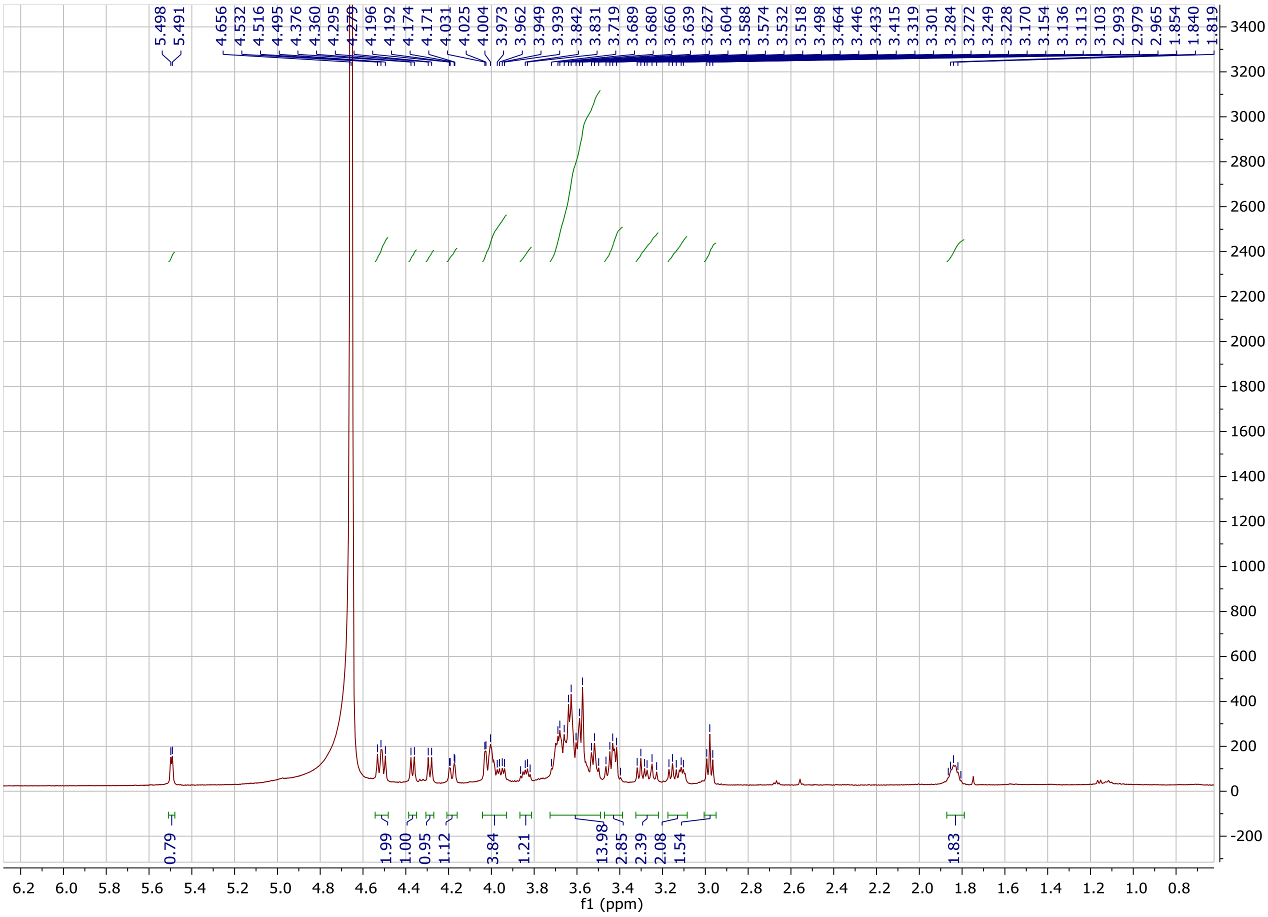

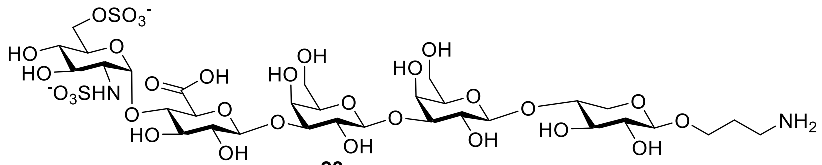

28

gHSQC (D_2O, 500 MHz) of **28**

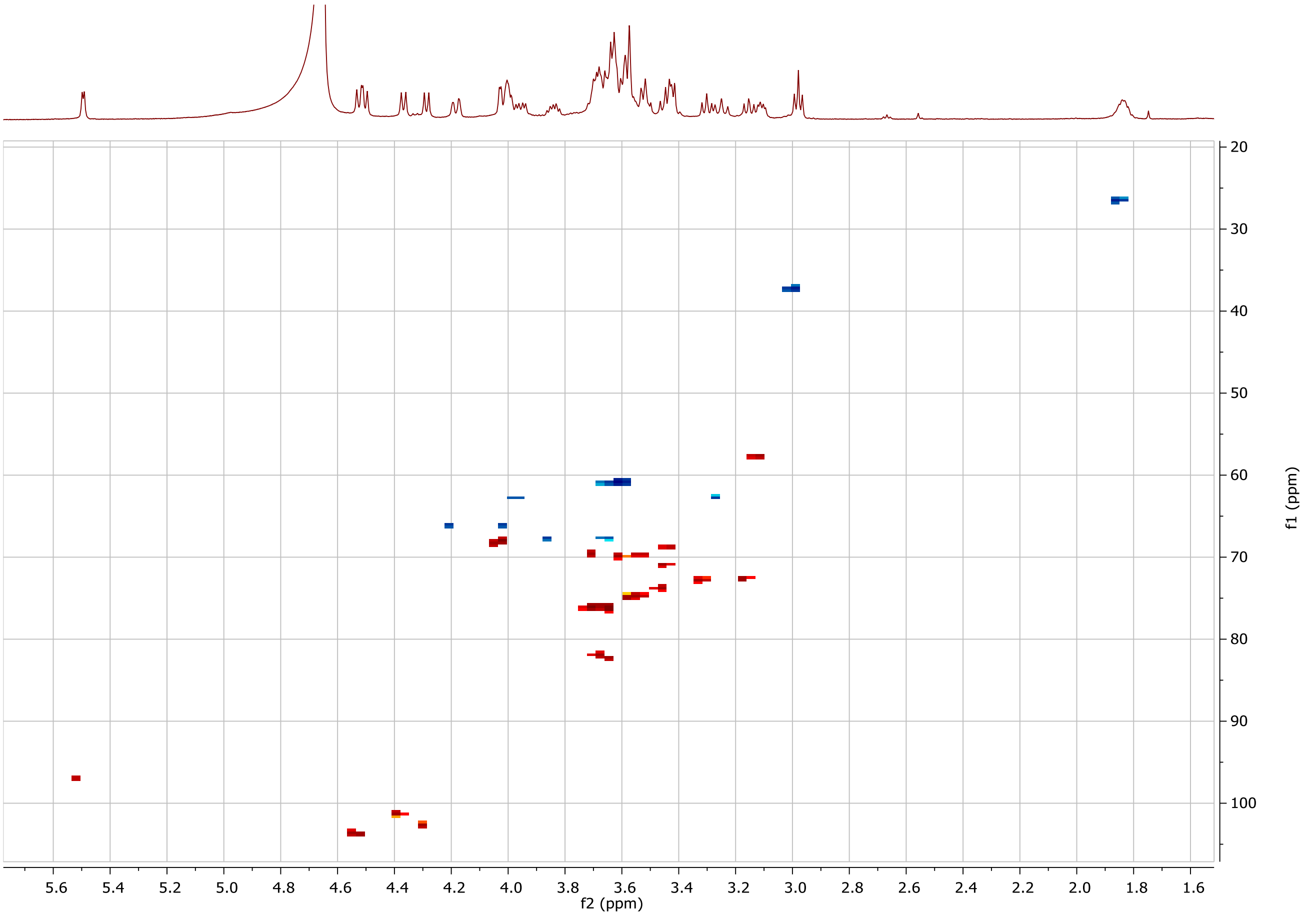

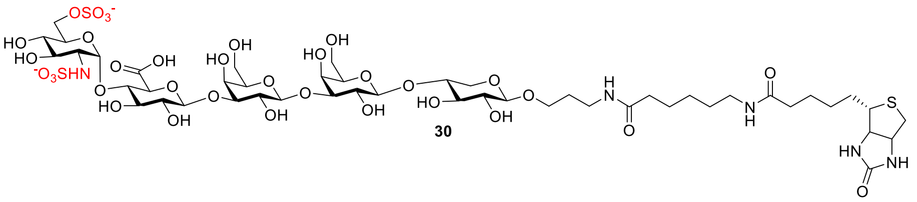

30

^{1}H-NMR (D_2O, 500 MHz) of **30**

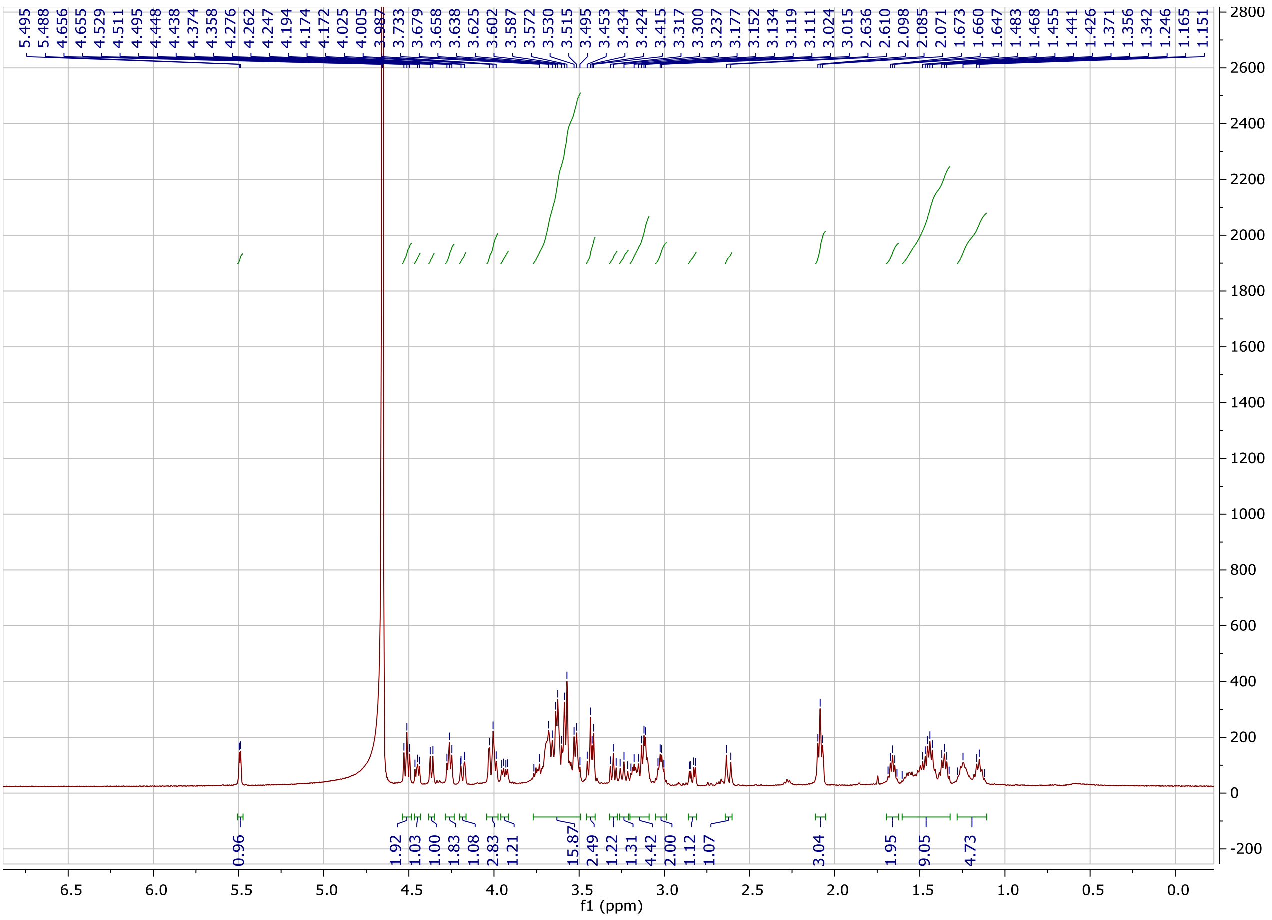

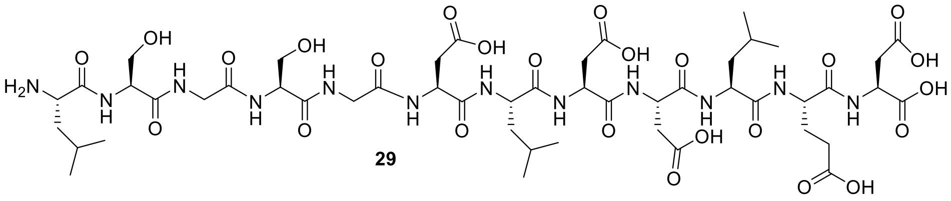

29

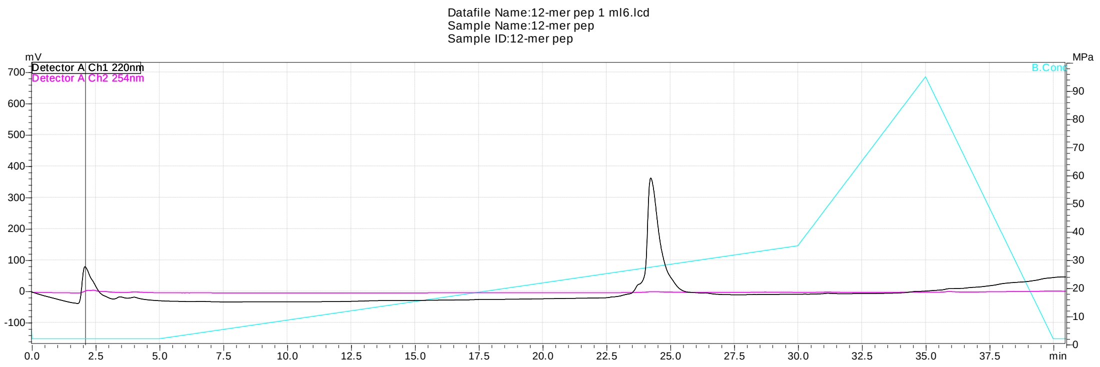
Datafile Name:12-mer pep 1 ml6.lcd
Sample Name:12-mer pep
Sample ID:12-mer pep
Detector A Ch1 220nm
Detector A Ch2 254nm
B.Conc
mV
MPa
min

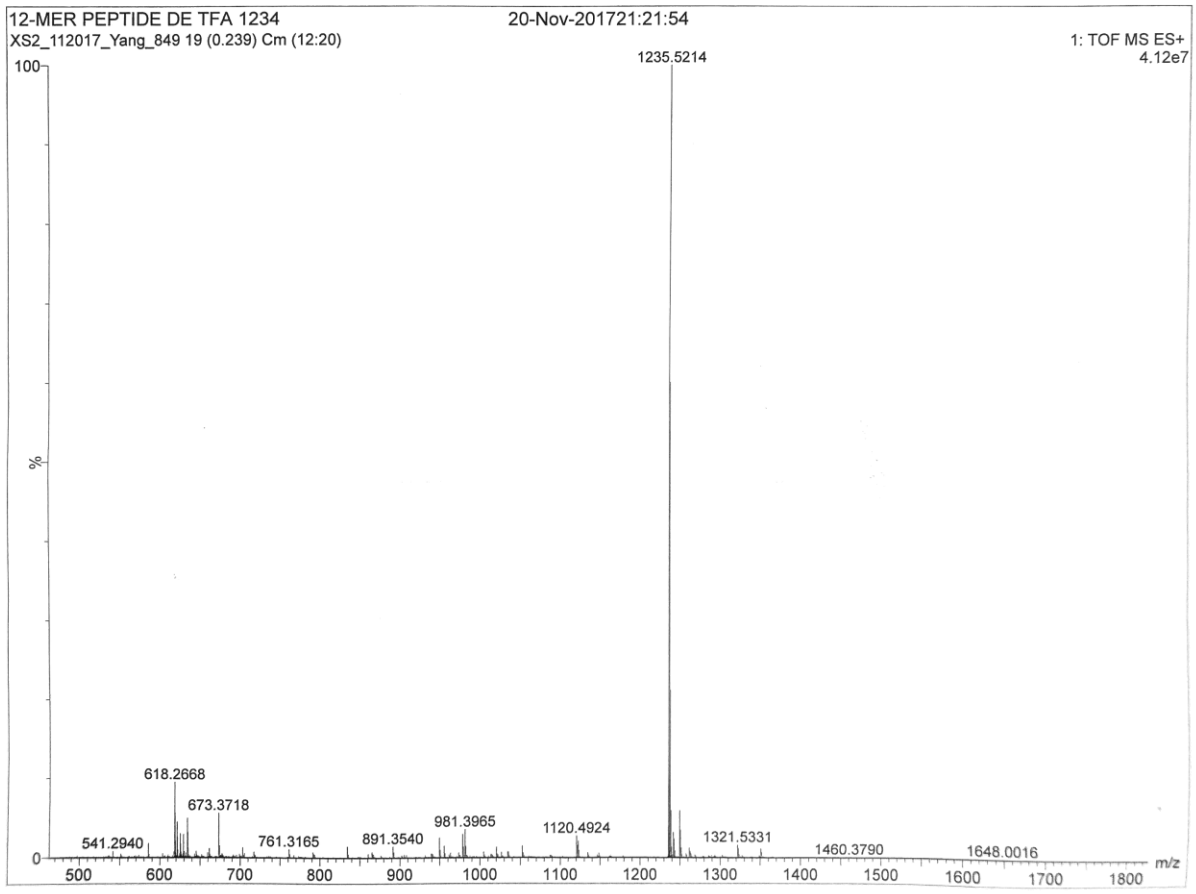
12-MER PEPTIDE DE TFA 1234
20-Nov-201721:21:54
XS2_112017_Yang_849 19 (0.239) Cm (12:20)
1: TOF MS ES+
4.12e7
1235.5214
618.2668
673.3718
541.2940
761.3165
891.3540
981.3965
1120.4924
1321.5331
1460.3790
1648.0016
%
m/z

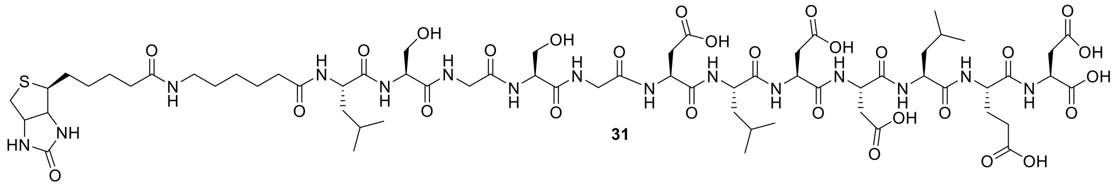

31

Datafile Name:1-15-2018-3.lcd
Sample Name:12-mer pep with biotin
Sample ID:12-mer pep with biotin

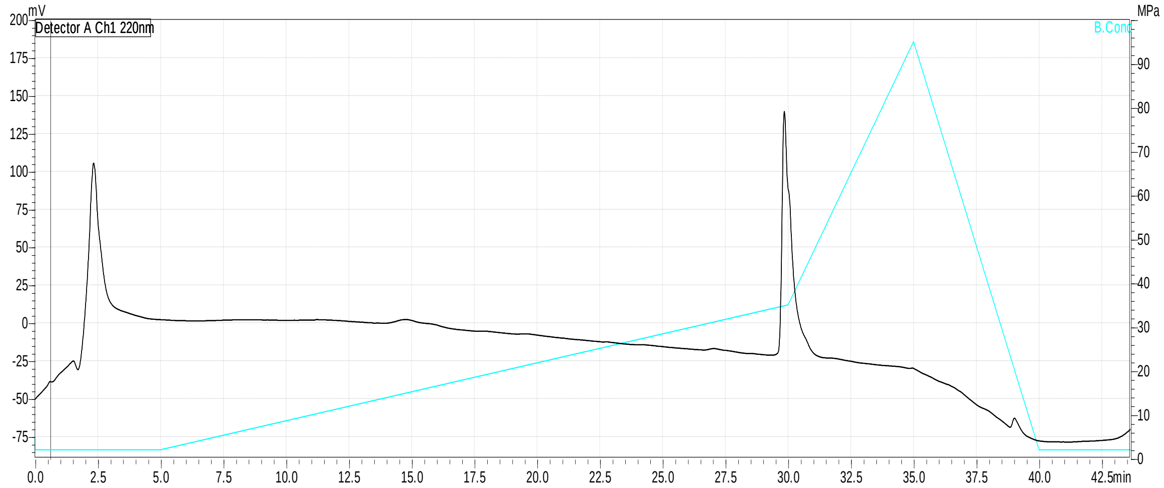

Supplement: SC-011-D0SC01140A-s001 [file SC-011-D0SC01140A-s001.pdf]
